# Highly Electrophilic, Catalytically Active and Redox‐Responsive Cobaltoceniumyl and Ferrocenyl Triazolylidene Coinage Metal Complexes

**DOI:** 10.1002/chem.201705051

**Published:** 2018-01-17

**Authors:** Stefan Vanicek, Maren Podewitz, Jessica Stubbe, Dennis Schulze, Holger Kopacka, Klaus Wurst, Thomas Müller, Petra Lippmann, Simone Haslinger, Herwig Schottenberger, Klaus R. Liedl, Ingo Ott, Biprajit Sarkar, Benno Bildstein

**Affiliations:** ^1^ Institute of General, Inorganic and Theoretical Chemistry University of Innsbruck, Center for Chemistry and Biomedicine Innrain 80–82 6020 Innsbruck Austria; ^2^ Institute of Chemistry and Biochemistry, Inorganic Chemistry Freie Universität Berlin Fabeckstraße 34–36 14195 Berlin Germany; ^3^ Institute of Organic Chemistry University of Innsbruck, Center for Chemistry and Biomedicine Innrain 80–82 6020 Innsbruck Austria; ^4^ Institute of Medicinal and Pharmaceutical Chemistry Technische Universität Braunschweig Beethovenstr. 55 38106 Braunschweig Germany

**Keywords:** catalysis, cytotoxicity, density functional calculations, mesoionic carbenes, sandwich complexes

## Abstract

A convenient access to a triad of triazoles with ferrocenyl and cobaltoceniumyl substituents is reported. N‐Alkylation, deprotonation and metalation with Cu^I^/Ag^I^/Au^I^ synthons affords the heteroleptic triazolylidene complexes. Due to the combination of neutral, electron‐donating ferrocenyl substituents and cationic, strongly electron‐withdrawing cobaltocenium substituents, the mesoionic carbene (MIC) ligands of these complexes are electronically interesting “push–pull”, “pull–push” and “pull–pull” metalloligands with further switchable redox states based on their fully reversible Fe^II^/Fe^III^, (ferrocene/ferrocenium) and Co^III^/Co^II^, (cobaltocenium/cobaltocene) redox couples. These are the first examples of metal complexes of (di)cationic NHC ligands based on cobaltoceniumyl substituents. DFT calculated Tolman electronic parameter (TEP) of the new MIC ligands, show these metalloligands to be extremely electron‐poor NHCs with properties unmatched in other carbene chemistry. Utilization of these multimetallic electronically tunable compounds in catalytic oxazoline synthesis and in antitumor studies are presented. Remarkably, 1 mol % of the Au^I^ complex with the dicationic MIC ligand displays full catalytic conversion, without the need for any other additives, in less than 2 hours at ambient temperatures. These results thus firmly establish these new classes of cobaltoceniumyl based (di)cationic MIC ligands as prominent players in several branches of chemistry.

## Introduction

In current organometallic chemistry, N‐heterocyclic carbene ligands (NHC) constitute one of the most important ligand classes for coordination chemistry with almost all elements of the periodic table.^1^ The high interest in NHC chemistry is clearly motivated by the many useful applications of their complexes in catalysis, materials science and medicinal chemistry.^1^ Among the various structural varieties possible, mesoionic carbenes (MICs) based on the 1,2,3‐triazol‐5‐ylidene core structure^2^ are increasingly popular, because they are conveniently synthesized by the Cu^I^ catalyzed azide‐alkyne [3+2] cycloaddition (CuAAC‐click‐chemistry) reaction.^3^ Whereas most NHCs are purely organic compounds in an apparently endless variation of stereoelectronic properties, functionalized NHCs with organometallic substituents are only scarcely known. Within this small subclass of NHCs, representatives containing electroactive substituents are especially interesting, because they allow electrochemical switching of their ligand properties with potentially beneficial effects in catalytic or sensoric applications. Until now such systems are mostly limited to some ferrocenyl NHCs/MICs and their complexes,^4^ due to easily available alkyne and azide ferrocene starting materials and due to the fully reversible Fe^II^/Fe^III^ ferrocene/ferrocenium redox couple, without doubt one of the most useful redox processes in electrochemistry.

In recent publications on mono‐ and bis‐ferrocenylated triazolylidene gold complexes,4e,4f we could show that redox‐switchable catalysis and reversible control of donor properties is possible. In general, catalysis by electrophilic gold species is a hot topic of current research in organic chemistry.^5^ In other recent work, we could develop reliable syntheses of isoelectronic cobaltocenium alkyne and azide starting materials,^6^ thereby enabling now an access to cobaltoceniumyl triazolylidene mesoionic carbene complexes via [3+2] cycloaddition reactions (vide infra). To the best of our knowledge, there are no reports of any NHC or MIC complexes containing cobaltoceniumyl substituents in the literature. In comparison to ferrocenyl donor substituents, cobaltoceniumyl acceptor substituents will cause opposite electronic properties due to their cationic charge and reversible Co^III^/Co^II^ cobaltocenium/cobaltocene redox couple. Compared to ferrocenyl‐substituted ligands, increased electrophilicity of the coordinated metal center can be expected. Furthermore, cobaltoceniumyl‐substituted complexes are intrinsically cationic and therefore highly soluble in polar solvents including water, an advantageous feature for catalytic applications with polar substrates under environmentally benign conditions or in medicinal applications. In recent work, we could show that cobaltoceniumyl‐substituted metal complexes^7^ exhibit significant antitumor properties in various cancer cell lines, therefore, cobaltoceniumyl triazolylidene copper/silver/gold complexes are of obvious interest as potential metallodrugs in bioorganometallic chemistry. Currently, mostly standard NHC metal complexes are investigated in this area,^8^ only very few triazolylidene complexes have been studied so far.^9^ Below we present the first synthetic report of metal complexes based on cationic MIC (or NHC) ligands containing cobaltoceniumyl substituents. Furthermore, we thoroughly investigate their electrochemical and electronic properties, and show their utility as ligands in gold(I) catalysis and in bioorganometallic chemistry.

## Results and Discussion

### Synthesis

**Scheme 1:**
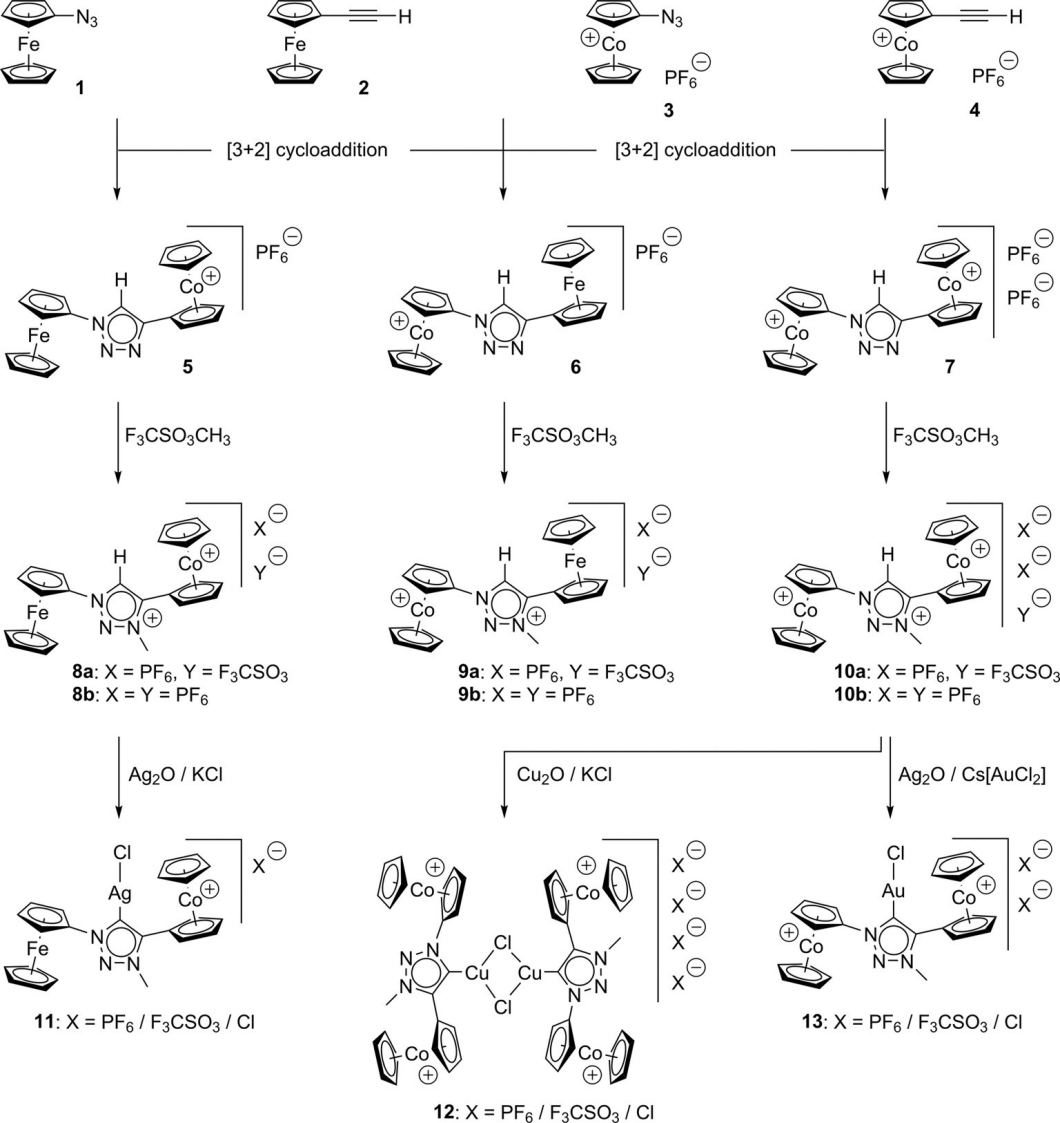
Synthesis of triazoles **5**–**7**, triazolium compounds **8 a**/**b**–**10 a**/**b** and triazolylidene complexes **11**–**13**.

Standard Cu^I^ catalyzed azide‐alkyne [3+2] cycloaddition reactions (CuAAC‐click‐chemistry)^3^ of azidoferrocene (**1**),^10^ ethynylferrocene (**2**),^11^ azidocobaltocenium hexafluoridophosphate (**3**)6b and ethynylcobaltocenium hexafluoridophosphate (**4**)6a afforded triazoles **5**–**7** in 63–84 % isolated yield (Scheme 1). Note that a diferrocenylated triazole, made similarly by cycloaddition of azidoferrocene with ethynylferrocene, is not included, because its synthesis and further chemistry has already recently been published.4f We also note that only one related example of a heterobimetallic cobaltoceniumyl/ferrocenyl triazole and triazolium containing a methylene‐spacered ferrocenyl substituent has been synthesized and electrochemically investigated,^12^ however, no metal complexes have been prepared therefrom.

Ferrocenyl/cobaltoceniumyl‐triazoles **5**/**6** are regioisomeric monocationic compounds, whereas dicobaltoceniumyltriazole **7** is a highly polar dicationic material. Due to the electron‐withdrawing cobaltoceniumyl substituents, all three triazoles **5**–**7** are quite poor nucleophiles, therefore, rather harsh conditions in the next alkylation step proved necessary. N‐Methylation with an excess of methyl triflate in refluxing dichloroethane afforded the desired dicationic (**8 a**, **9 a**) or tricationic (**10 a**) triazolium hexafluoridophosphates/triflates in 80–96 % yield, assuming that the additional anion is triflate as one would expect with methyl triflate as the methylating agent. Exchange of the counterions on a hexafluoridophosphate‐loaded ion‐exchange column afforded the pure triazolium hexafluoridophosphates (**8 b**, **9 b**, **10 b**) in 94–96 % yield.

Formation of triazolylidene ligands by deprotonation of **8 a**–**10 a** and concomitant complex formation was investigated under a wide variety of experimental conditions using various prospective bases and metal precursors commonly used in NHC chemistry.^1^ Although **8 a**–**10 a** represent quite acidic triazolium salts due to their strongly electron‐withdrawing cobaltocenium substituents (vide infra), it proved surprisingly difficult to get access to the desired heteroleptic coinage metal complexes. One obstacle is the known common nucleophilic addition of nucleophiles (e.g. carbanions or hydride) at a cationic cobaltocenium moiety, another obstacle is the steric hindrance imposed by the two α‐metallocenyl substituents, preventing deprotonation by bulky bases (e.g. LiN(SiMe_3_)_2_ or Schlosser base KO*t*Bu/*n*BuLi) as well as preventing transmetalation to Au^I^ using common synthons such as (triphenylphosphine)AuCl. In addition, strongly reducing reaction conditions are not compatible with cobaltocenium compounds and Au^I^ synthons.

However, in situ deprotonation by Cu_2_O and Ag_2_O in acetonitrile solution in the presence of an excess of KCl with molecular sieve as water scavenger did serve the purpose for triazolium salts **8 a** and **10 a**, whereas the least acidic triazolium precursor **9 a** (vide infra) proved unreactive (Scheme 1), thereby affording triazolylidene complexes **11** and **12** in reasonable yields. An excess of KCl was used to generate the desired heteroleptic triazolylidene/chlorido complexes intended for catalytic applications and anti‐cancer bioorganometallic studies (vide infra), otherwise mixtures containing heteroleptic and catalytically and biologically inactive homoleptic triazolylidene complexes were observed. However, copper complex **12** could only be obtained as a mixture with its precursor **10 a** (60 %/40 %) according to NMR and X‐ray single crystal structure analysis (vide infra) and unfortunately all attempts to separate this mixture proved so far unsuccessful. Gold complex **13** was obtained by a sequential reaction of triazolium salt **10 a** with Ag_2_O followed by transmetalation with caesium dichlorideaurate, Cs[ClAuCl]. This precursor has never been used for this kind of gold(I) chemistry and might also be useful for other workers, due to its high solubility in polar solvents and due to its easily displaced chlorido ligands attached to a sterically unhindered gold center. The relative ratio of the counterions of complexes **11**–**13** is difficult to specify due to three potential anions in these syntheses. Mass spectrometry and IR spectroscopy show analytical evidence of hexafluoridophosphate and triflate (compare Experimental Section). From a chemical point of view these two anions are also the most likely counterions because of increased solubility of their salts in an apolar solvent like acetonitrile; chloride salts are in general much less soluble. However, the main focus of complexes **11**–**13** is their coordination chemistry based on their cationic triazolylidene ligands, therefore we consider various innocent counteranions as tolerable.

### Physical, spectroscopic and structural properties

Triazoles and triazolium compounds **5**–**10** are air‐stable yellow to red powders with melting points in the range of 173–240 °C. As expected, higher melting points are observed for the higher charged dicationic and tricationic salts **7**–**10 a/b** in comparison to monocationic systems **5** and **6**. In the IR‐spectra of **5**–**10 a/b,** strong ν_P−F_ absorptions at approximately 820 and 557 cm^−1^ are present due to the hexafluoridophosphate counterions. NMR spectra are in line with regular triazole or triazolium heterocycles containing monosubstituted ferrocenyl or cobaltoceniumyl groups displaying the common pattern of singlets for the unsubstituted Cp rings and pseudo‐triplets for the substituted Cp ring. As expected, cobaltoceniumyl‐related signals are observed at lower field in comparison to those of the neutral ferrocenyl groups. The NMR signals of the C−H functionality of triazoles **5**–**7** (^1^H NMR: δ_C−H_=8.71–9.15, ^13^C NMR: δ_C−H_=120.0–125.4) are shielded in comparison to those of the C−H functionality of *N*‐methyl triazolium salts **8 a/b**–**10 a/b** (^1^H NMR: δ_C−H_=9.67–10.14, δ_N−CH3_=4.60–4.71, ^13^C NMR: δ_C−H_=128.8–147.0, δ_N−CH3_=40.7–41.5). These spectroscopic data indicate considerable acidic character of the C−H functionality of these di‐ and tricationic triazolium heterocycles (vide infra), a precondition for facile deprotonation and concomitant triazolylidene complex formation.

Triazolylidene complexes **11**–**13** are air‐stable yellow to orange salts with melting points in the range of 150–214 °C. Spectral features (NMR, IR, MS) are in line with expectations. Most significant, ^13^C NMR signals of the carbene carbons are observed in a narrow range at 166.4 ppm (**11**), 160.3 ppm (**12**), and 162.2 ppm (**13**).

**Figure 1:**
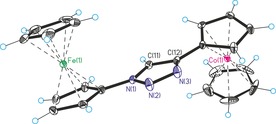
Molecular structure of the cation of **5** (counteranion hexafluoridophosphate omitted for clarity). Selected bond lengths (Å): N(1)−N(2)=1.342(7), N(2)−N(3)=1.320(7), N(1)−C(11)=1.345(7), C(11)−C(12)=1.361(8), N(3)−C(12)=1.348(7).

**Figure 2:**
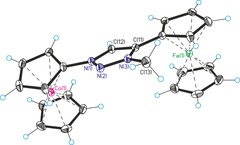
Molecular structure of the dication of **9 a** (counteranions hexafluoridophosphate and triflate omitted for clarity). Selected bond lengths (Å): N(1)‐N(2)=1.323(3), N(2)−N(3)=1.323(3), N(1)−C(12)=1.353(3), C(11)−C(12)=1.369(4), N(3)−C(11)=1.366(3), N(3)−C(13)=1.465(3).

**Figure 3:**
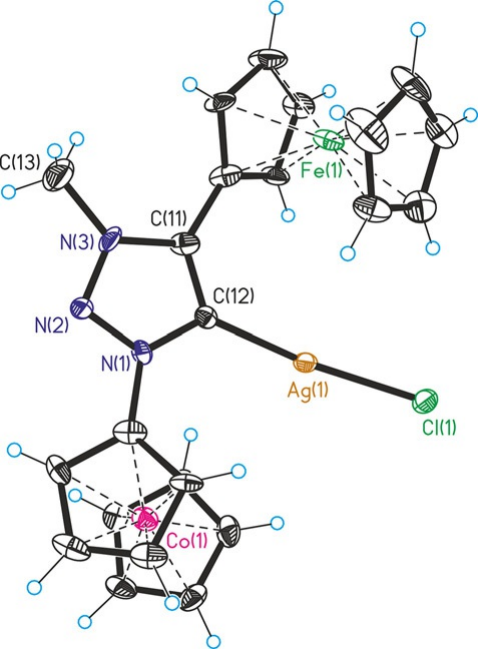
Molecular structure of the cation of **11** (counteranion hexafluoridophosphate omitted for clarity). Selected bond lengths (Å): Ag(1)−Cl(1)=2.345(3), Ag(1)−C(12)=2.064(12), N(1)−N(2)=1.324(13), N(2)−N(3)=1.328(14), N(1)−C(12)=1.364(14), C(11)−C(12)=1.372(17), N(3)−C(11)=1.375(16), N(3)−C(13)=1.454(17). Selected angle (°): C(12)‐Ag(1)‐Cl(1)=169.6(3).

**Figure 4:**
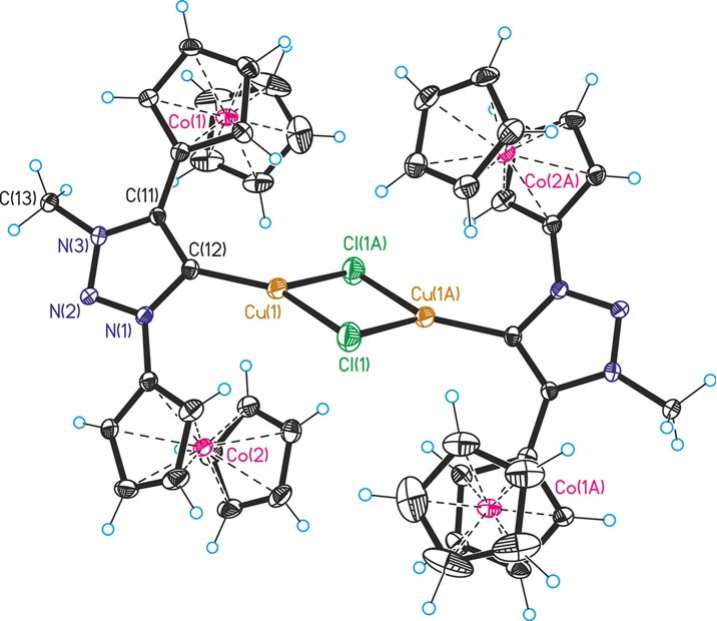
Molecular structure of the tetracation of **12** (counteranions hexafluoridophosphate and triflate omitted for clarity). Selected bond lengths (Å): Cu(1)−Cl(1)=2.2670(19), Cu(1)−C(12)=1.900(5), N(1)−N(2)=1.343(5), N(2)−N(3)=1.314(6), N(1)−C(12)=1.376(6), C(11)−C(12)=1.402(7), N(3)−C(11)=1.364(6), N(3)−C(13)=1.470(6). Selected angle (°): C(12)‐Cu(1)‐Cl(1)=144.75(16).

**Figure 5:**
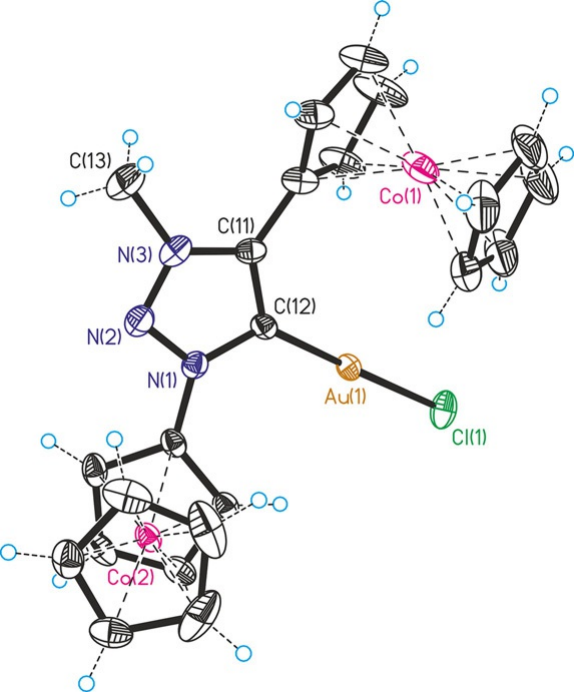
Molecular structure of the dication of **13** (counteranions hexafluoridophosphate and triflate are omitted for clarity). Selected bond lengths (Å): Au(1)−Cl(1)=2.2836(12), Au(1)−C(12)=1.982(4), N(1)−N(2)=1.332(5), N(2)−N(3)=1.301(5), N(1)− (12)=1.373(5), C(11)−C(12)=1.385(6), N(3)−C(11)=1.357(6), N(3)−C(13)=1.480(6). Selected angle (°): C(12)‐Au(1)‐Cl(1)=178.18(12).

Single crystal structure analyses are available for nine compounds in this study (see Supporting Information). Exemplary, two representative carbene metal complex precursors will be discussed. Figures 1 and Figure 2 show the molecular structures of triazole **5** and triazolium **9 a** with counterions omitted for clarity. Regular aromatic triazole/triazolium heterocycles containing undistorted ferrocenyl/cobaltoceniumyl substituents are observed; selected bond lengths are given in the Figure captions. Overall, these single crystal X‐ray structures, together with those in the Supporting Information, prove the identity of all compounds in this paper. Figures 3, 4 and 5 show the molecular structures of the cations of triazolylidene complexes **11**, **12** and **13**. All three structures exhibit regular triazole heterocycles with variably twisted ferrocenyl or cobaltoceniumyl substituents, most likely due to packing effects depending on charge (monopositive or dipositive) and counterions (hexafluoridophosphate and/or triflate). The metallocenyl groups are again regular sandwich moieties with structural metrics in line with expectations.

Silver and gold complexes **11** and **13** show the anticipated structures of simple heteroleptic complexes with linear coordination geometry at the metal centers. In contrast, copper complex **12** is a dimer formed by bridging chlorido ligands resulting in a distorted trigonal‐planar coordination at copper. The occurrence of a dimeric structure in the case of copper complex **12**, but not for silver complex **11** and gold complex **13**, might be explained by a weaker copper‐carbene bond compared to stronger bonding in Ag/Au‐carbene complexes necessitating additional electron‐donation by a bridging chlorido ligand. The carbene‐metal bond lengths [**11**: Ag(1)−C(12)=2.064(12) Å, **12**: Cu(1)−C(12)=1.900(5) Å, **13**: Au(1)−C(12)=1.982(4) Å] are also unexceptional and similar to those of other Cu/Ag/Au NHC complexes.^13^

### DFT studies of triazolylidene ligands

**Figure 6:**
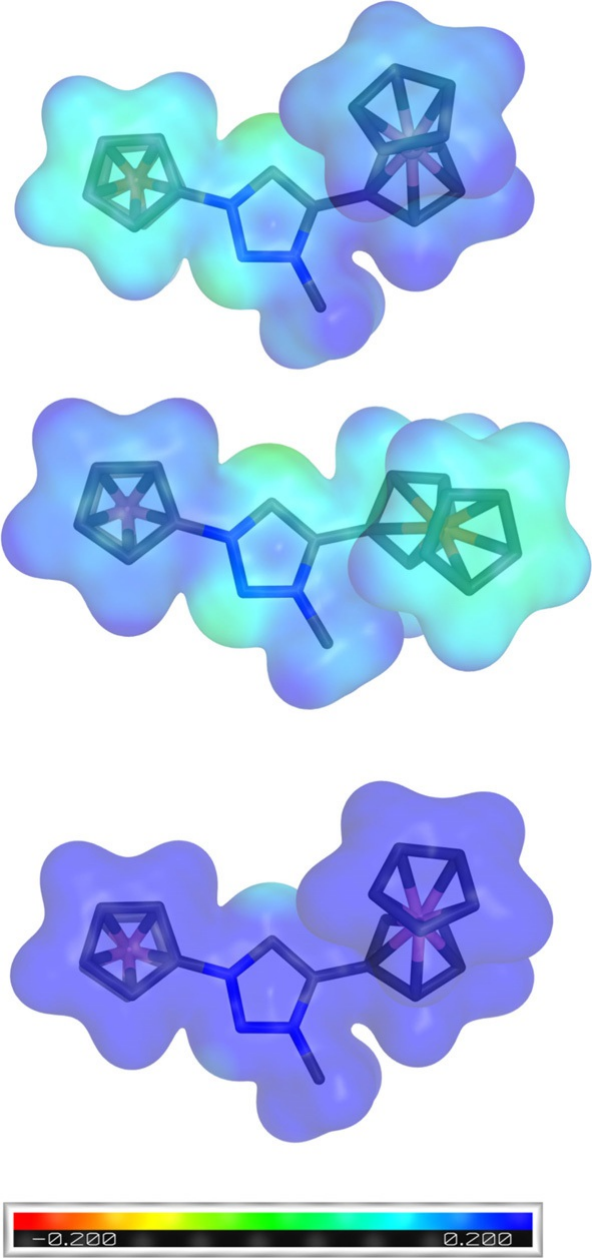
Molecular electrostatic potential (in a.u.) of the triazolylidene ligands **8′** (top), **9′** (middle) and **10′** (bottom) derived from triazolium salts **8 a**, **9 a** and **10 a**, calculated with BP86/def2‐TZVP/BJ. An isodensity of 0.1 a.u. was chosen. Red areas indicate a negative MEP, blue areas a positive MEP.

To evaluate the ligand properties of these metallocenyl triazolylidene ligands, to explore their electronic structure, and to determine their reactivity, density functional theory (DFT) calculations of **8 a**–**10 a** were performed (see computational methodology and Supporting Information for details).^14^ While the conformation of the ferrocenyl and cobaltoceniumyl groups differ in their X‐ray crystal structures, pointing in opposite directions (*anti* conformation) in **8 a** and **9 a** and the same direction (*syn* conformation) in **10 a**, all quantum chemical structure optimizations predicted the *anti* conformations to be more stable (see SI Table S55). Structural parameters of the triazole ring are very similar for all conformers and in good agreement with experimental data from single crystal diffraction (see Supporting Information Table S56). Calculations of the molecular electrostatic potential (MEP) of the deprotonated species **8′** to **10′**, were performed to determine the reactivity of the three metallocenyl triazolylidene ligands.^15^ The MEPs, depicted in Figure 6, revealed similarities and differences between the three ligands. Overall, all three structures have rather positive MEPs indicated by the absence of red colored areas in Figure 6 and show a more negative MEP at the carbene atom (illustrated by a greenish coloring) compared to the rest of the molecule. However, the MEP of the cobaltoceniumyl substituent is significantly more positive as indicated by the blue color than that of ferrocenyl making the triazole ring of the disubstituted cobaltoceniumyl species **10′** more electron deficient than those of **8′** and **9′**.

To explore the consequences of these electronic differences, we calculated p*K*_a_ values in water (treated as implicit solvent)^16^ according to the thermodynamic cycle depicted in Scheme S1 (Supporting Information). The p*K*_a_ values further confirmed the different character of **8 a** and **9 a** vs. **10 a**: while for **8 a** and **9 a** very similar p*K*_a_ values of around 23 were found with **9 a** having a slightly higher p*K*_a_ value, a p*K*_a_ of approx. 18 was determined for **10 a**. Thus, the tricationic dicobaltoceniumyl‐substituted triazolium salt **10 a** is significantly more acidic than dicationic **8 a** and **9 a**.

**Figure 7:**
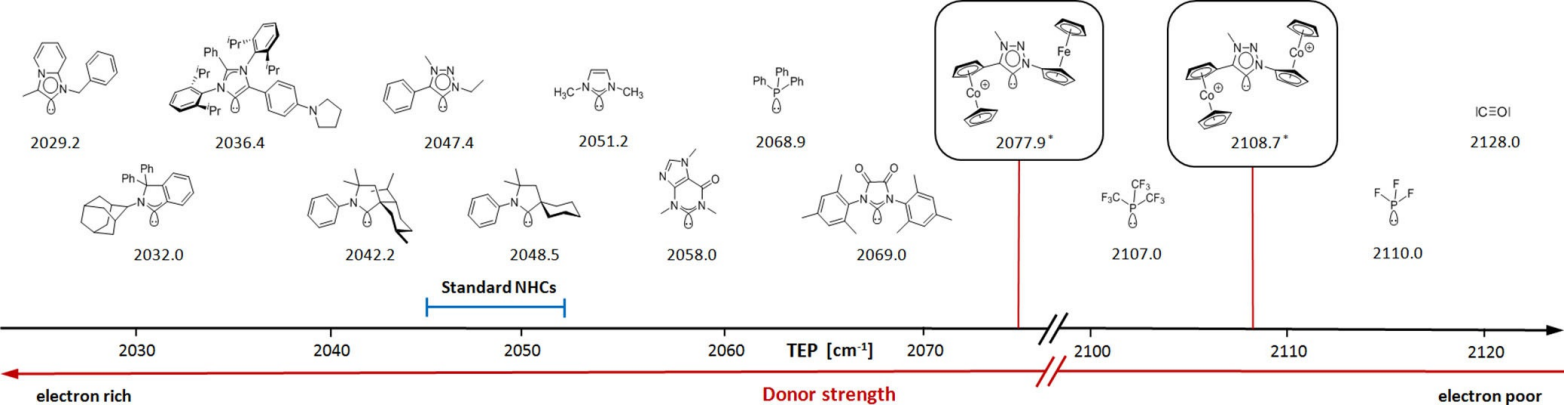
Tolman electronic parameter (TEP) of metallocenyl triazolylidene ligands in comparison to those of NHCs and other two‐electron ligands. *TEP values calculated (see text and Supporting Information).

To compare these metallocenyl triazolylidenes in their σ‐donor/π‐acceptor properties with NHCs and other common two‐electron ligands, their Tolman electronic parameter (TEP)^17^ was calculated and values of 2077.9 cm^−1^ (**8′**), 2077.1 cm^−1^ (**9′**) and 2108.7 cm^−1^ (**10′**) were obtained (Figure 7) using B3LYP/6–311++G(d,p).^17^ Overall, these are by far the most electrophilic NHCs reported to date. There are only very few mono‐cationic NHCs known^18^ and their TEP values are well below 2077 cm^−1^. Quite remarkably, the TEP value of the dicationic triazolylidene **10′** (2108.7 cm^−1^) is very close to that of PF_3_ (2110.0 cm^−1^) which is the most π‐acidic phosphine^19^ with strong back‐bonding similar to carbon monoxide. Therefore, we have to consider these metallocenyl triazolylidene, **8′**, **9′** and especially **10′,** as extremely electron‐poor, highly π‐acidic NHCs with very strong back‐bonding properties.

Further characterization of the electronic properties of these unusual carbenes by experimental approaches,^17^ for example, TEP measurement via IR absorptions of their Rh(CO)_2_Cl complexes and π‐acidity evaluation via ^77^Se‐NMR chemical shifts of their selenium derivatives would be highly desirable. However, we experienced major difficulties in attempted synthesis of these compounds under a variety of conditions commonly used in NHC chemistry,^1^ most likely due to instability of the “free” mono‐ or dicationic carbenes in combination with steric hindrance in the case of Rh(CO)_2_Cl complexes and heterogeneous reaction conditions in the case of Se derivatives, respectively. Therefore an experimental proof of the σ‐donor/π‐acceptor properties is unfortunately not easily possible as yet.

### Electrochemistry and redox‐switchable catalysis

**Figure 8:**
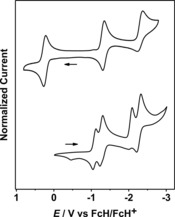
Cyclic voltammograms of **5** (top) and **7** (bottom) in CH_3_CN/Bu_4_NPF_6_.

The redox‐active triazoles, triazolylidenes, and the coinage metal complexes were investigated by cyclic voltammetry in CH_3_CN with either Bu_4_NPF_6_ or with Bu_4_NBAr^F^ as supporting electrolytes. The triazoles **5** and **6** display one oxidation and two reduction steps (Figures 8 and S62). In comparison to related compounds,4f, 6b the oxidation step is assigned to a ferrocenyl‐based Fe^II^ to Fe^III^ process, and the reduction steps to the cobaltoceniumyl‐based successive reduction of Co^III^, via Co^II^ to Co^I^. Whereas the first reduction is reversible, the second reduction is quasi‐reversible, a fact that is likely related to the lability of the cobalt−Cp bond on reduction. As expected, the N‐bound ferrocenyl unit in **5** is more difficult to oxidize than the C‐bound one in **6**. In keeping with the same trends, the N‐bound cobaltoceniumyl unit in **5** is easier to reduce compared to the C‐bound counterpart in **6** (Table S59).

For **7**, that contains only cobaltoceniumyl substituents, no oxidation steps are observed. However, a total of four reduction waves are seen (Figure 8) which correspond to the stepwise reduction of each of the Co^III^ centers via Co^II^ to Co^I^. The difference between the first two reduction waves (Table S59) delivers a comproportionation constant *K*_c_ of 1.1×10^3^ for the Co^III^‐Co^II^ mixed‐valent species. Similarly, the difference between the third and fourth reduction potentials delivers a *K*_c_ of 7.9×10^3^ for the Co^II^−Co^I^ mixed‐valent species (Table S59). For the methylated triazolium salts **8 a**, **9 a** and **10 a**, an extra reduction wave is observed compared to the corresponding triazoles (Figures S64–S66). This process is assigned to the reduction of the triazolium ring.4f As expected, all redox potentials for the triazolium salts are anodically shifted compared to their triazole counterpart (Table S59). For compound **10 a**, the use of Bu_4_NBAr^F^ instead of Bu_4_NPF_6_ led to an increase of the separation between the first two reduction potentials from almost negligible to 120 mV, thus delivering a *K*_c_ of 1.07×10^2^, a value that is an order of magnitude smaller compared to that of **7** (Figures S69 and S70). The electrochemistry of the Ag^I^ complex **11** was marred by the deposition of elemental silver on the electrode surface following redox processes (Figure S71). Only one reversible oxidation step could be detected for this compound (Figure S72). Just like **7** and **10 a**, the Au^I^ complex **13** displays four reduction steps, the first two of which are completely reversible (Figures S73 and S74). The use of different electrolytes did not have any influence on the separation between the first two reduction waves in this case (Figures S74 and S75). The difference of 120 mV delivers a *K*_c_ of 1.07×10^2^ for the Co^III^−Co^II^ mixed valent Au^I^ heterotrimetallic complex.

**Figure 9:**
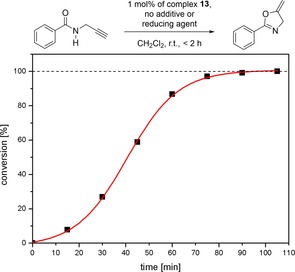
Synthesis of oxazoline catalyzed by complex **13** (top), and the time versus conversion plot for the same reaction (bottom).

Owing to the cationic nature of complexes **11**–**13**, and the extremely electron‐poor nature of the carbenes, we decided to use them as precatalysts for the formation of oxazoline **B** from substrate **A** (Figure 9). As anticipated, the silver complex **11** and the copper complex **12** did not show any conversion in this reaction. However, the use of 1 mol % of the Au^I^ complex **13** at ambient temperatures delivered quantitative conversion in less than 2 hours. Remarkably, no additives were needed for this reaction, showing the potency of the dicationic MIC ligands in Au^I^ catalysis. This is a unique example of an extremely potent, additive‐free Au^I^ catalyst. The catalytic results thus corroborate the extremely electron‐poor nature of these carbenes as postulated by calculation of the TEP parameters. We also tested the potential redox‐switchability of this reaction by the addition of slightly more than two equivalents of cobaltocene as a reducing agent. The addition of the reducing agent completely stops the catalysis, and no further conversion to the product is observed (Figure S76). However, further addition of ferrocenium hexafluoridophosphate as an oxidizing agent did not restore back the catalytic activity of the system (Figure S77). Thus, it is seen that even though the activity of these cobaltoceniumyl containing Au^I^ complexes are much higher than their recently published ferrocenyl analogues (in the oxidized form), their redox stability during catalysis is poorer compared to their ferrocenyl counterparts.4f,4g The exact reason for these differences are part of ongoing research.

### Bioorganometallic chemistry

Organometallic complexes have recently attracted major attention in the fields of inorganic medicinal chemistry and bioorganometallic chemistry.8a Heteropolynuclear organometallics are of interest as they can combine several metal‐related biological properties in a single molecule. The effects of **11**–**13** against the breast cancer lines MCF‐7 and MDA‐MB‐231, the colon carcinoma cell line HT‐29 and RC‐124 non‐tumorigenic kidney primary cells were investigated (Table 1). Whereas **12** was inactive (IC_50_ values >100 μm) against all investigated cell lines, complexes **11** and **13** showed moderate effects in the range of 9–50 μm (with the exception of **9** with HT‐29 cells). A tumor selectivity was not noted, as RC‐124 cells were affected with comparable potency to the tumor cell lines. The inactivity of **12** might be related to the dimeric nature of the compound or to decomposition after a possible oxidation of the Cu^I^ center. The anticancer potential of organometallic silver and gold species is well established, and can be considered as the driving force behind the observed antiproliferative effects of **11** and **13**.8b,8c,8d

**Table 1:** Cytotoxic effects of **11**–**13** in cell lines as IC_50_ values (μm) with experimental errors between brackets.

	**HT‐29**	**MCF‐7**	**MDA‐MB‐231**	**RC‐124**
**11**	45.6(6.6)	27.0(6.4)	28.6(2.3)	9.0(3.0)
**12** ^[a]^	>100	>100	>100	>100
**13**	>100	48.4(8.0)	49.6(4.2)	34.3(6.7)

[a] The concentration was calculated from the monomeric form.

## Conclusion

CuAAC‐click‐chemistry starting from iron/cobalt metallocene azides and alkynes gave access to three triazoles containing directly attached ferrocenyl and cobaltoceniumyl substituents. N‐Methylation with methyl triflate afforded the corresponding di‐ or tricationic triazolium salts as NHC precursors of triazolylidene metal complexes. Heteroleptic Cu^I^, Ag^I^ and Au^I^ triazolylidene complexes containing chlorido coligands were synthesized by deprotonation and complex formation with Cu_2_O and Ag_2_O or by transmetalation with caesium dichloridoaurate from in situ formed Ag^I^ complexes. Full spectroscopic (NMR, IR, MS) characterization as well as single crystal X‐ray structure analyses for all compounds are reported. These are the first examples of NHC complexes containing cobaltoceniumyl substituents. DFT calculations showed triazolylidene ligands with cobaltoceniumyl substituents to be extremely electron‐poor NHC ligands with Tolman electronic parameters up to 2109 cm^−1^, almost identical to that of the most electrophilic phosphine ligand PF_3_. The more cobaltoceniumyl substituents the electron poorer and the more acidic the metallocenyl triazolylidene ligands are. The ligand precursors and metal complexes display ferrocenyl‐based oxidation and two successive cobaltoceniumyl‐based reductions. Owing to the extremely electron‐poor nature of the dicationic carbene, its Au^I^ complex was used as a precatalyst for oxazoline synthesis. Under ambient conditions, 1 mol % of the complex delivered quantitative conversion within less than 2 h without the need of any additional additive. A complete redox‐switchablity could unfortunately not be achieved for this catalytic system. Moderate cytotoxicity was observed for the heteroleptic triazolylidene chloride coinage metal complexes related to the respective silver or gold centers.

Overall, the use of cobaltoceniumyl substituents as very strong acceptor substituents and electrochemically switchable units significantly expands standard NHC chemistry. In future, we will explore the high electrophilic reactivity of these cobaltoceniumyl/ferrocenyl triazolylidenes, both in transition metal coordination chemistry and catalysis as well as in small molecule fixation and/or activation.

## Experimental Section

**General Procedures**. Synthetic methods and spectroscopic characterization as well as single‐crystal structure analysis were performed as described recently.^6^, ^7^, ^20^ Chemicals were obtained commercially and used as received. Starting materials azidoferrocene (**1**),^10^ ethynylferrocene (**2**),^11^ azidocobaltocenium hexafluoridophosphate (**3**)^6^ and ethynylcobaltocenium hexafluoridophosphate (**4**)^6^ were synthesized as published.

**Triazole (5)**. A 250 mL Schlenk tube was charged under an atmosphere of Ar with 60 mL of dry THF and 40 mL of H_2_O. The mixture was protected from light by wrapping with aluminum foil and cooled in an ice bath to 0 °C. Ethinylcobaltocenium hexafluoridophosphate (1.00 g, 2.79 mmol, 1 equiv), azidoferrocene (0.643 g, 2.79 mmol, 1 equiv) and CuSO_4_⋅5 H_2_O (0.697 g, 2.79 mmol, 1 equiv) was added. After 3 min of stirring, an aqueous solution of sodium ascorbate (1.383 g, 6.98 mmol, 2.5 equiv, dissolved in 5 mL of degassed H_2_O) was added, the cooling bath was removed and stirring was continued overnight, resulting in a deep red heterogeneous mixture. Workup under ambient conditions: Red solid Cu_2_O was filtered off, the precipitate was washed with three portions of dichloromethane and the organic solvents of the combined solutions were removed on a rotary evaporator, resulting in an orange oil with a yellow aqueous phase. This mixture was extracted with three portions of dichloromethane, the combined organic phases were washed with one portion of H_2_O and dried over Na_2_SO_4_. The dichloromethane solution was reduced in volume on a rotary evaporator to approximately 15 mL and 400 mL of Et_2_O was added to precipitate the product at −20 °C in a freezer. The yellow precipitate was filtered off, washed with two small portions of Et_2_O and dried in vacuo, affording 1.364 g (2.33 mmol) of **5** in 83.6 % yield. Compound **5** is air‐stable and soluble in dichloromethane, acetone and acetonitrile. M.p: 177 °C; ^1^H‐NMR (300 MHz, C_3_D_6_O): *δ*=4.27 (s, 5 H, Fc‐Cp), 4.40 (pseudo‐t, 2 H, *J*=2.0 Hz, C3/C4 of subst. Fc‐Cp), 5.04 (pseudo‐t, 2 H, *J*=2.0 Hz, C2/C5 of subst. Fc‐Cp), 5.80 (s, 5 H, Cc‐Cp), 6.05 (pseudo‐t, 2 H, *J*=2.1 Hz, C3/C4 of subst. Cc‐Cp), 6.46 (pseudo‐t, 2 H, *J*=2.1 Hz, C2/C5 of subst. Cc‐Cp), 8.93 (s, 1 H, CH of triazole) ppm; ^13^C‐NMR (75 MHz, C_3_D_6_O): *δ*=62.9 (C3/C4 of subst. Fc‐Cp), 67.9 (C2/C5 of subst. Fc‐Cp), 71.1 (Fc‐Cp), 81.8 (C3/C4 of subst. Cc‐Cp), 85.5 (C2/C5 of subst. Cc‐Cp), 86.9 (Cc‐Cp), 94.3 (quart. carbon of subst. Fc‐Cp), 96.7 (quart. carbon of subst. Cc‐Cp), 124.3 (CH of triazole), 139.1 (quart. carbon of triazole) ppm; IR (ATR): 3117 (ν_C−H_), 1590, 1525, 1452, 1420 (ν_C=C_), 1228, 1185, 1106, 1050, 1004, 808 (ν_P−F_), 557 (ν_P−F_), 508, 484, 454 cm^−1^; MS (ESI pos): *m*/*z* 440.02 (M^+^−PF_6_^−^). Single crystals of **5** were obtained from an acetone solution at room temperature. Single crystal analysis (Figure 1), Supporting Information: Spectra and crystallographic details.

**Triazole (6)**. Compound **6** was prepared in a similar manner as described above for **5**. Starting materials: Azidocobaltocenium hexafluoridophosphate (0.200 g, 0.53 mmol, 1 equiv), ethinylferrocene (0.112 g, 0.53 mmol, 1 equiv), CuSO_4_⋅5 H_2_O (0.133 g, 0.53 mmol, 1 equiv), sodium ascorbate (0.264 g, 1.33 mmol, 2.5 equiv). Product **6**: burgundy red powder, 0.196 g (0.33 mmol), 62.8 % yield. Compound **6** is air‐stable and soluble in dichloromethane, acetone and acetonitrile. M.p: 173 °C; ^1^H‐NMR (300 MHz, C_3_D_6_O): *δ*=4.09 (s, 5 H, Fc‐Cp), 4.39 (pseudo‐t, 2 H, *J=*1.8 Hz, C3/C4 of subst. Fc‐Cp), 4.83 (pseudo‐t, 2 H, *J=*1.8 Hz, C2/C5 of subst. Fc‐Cp), 5.97 (s, 5 H, Cc‐Cp), 6.09 (pseudo‐t, 2 H, *J=*2.3 Hz, C3/C4 of subst. Cc‐Cp), 6.70 (pseudo‐t, 2 H, *J*=2.3 Hz, C2/C5 of subst. Cc‐Cp), 8.71 (s, 1 H, CH of triazole) ppm; ^13^C‐NMR (75 MHz, C_3_D_6_O): *δ*=67.7 (C3/C4 of subst. Fc‐Cp), 69.9 (C2/C5 of subst. Fc‐Cp), 70.4 (Fc‐Cp), 75.2 (quart. carbon of subst. Fc‐Cp), 76,1 (C3/C4 of subst. Cc‐Cp), 83.3 (C2/C5 of subst. Cc‐Cp), 87.3 (Cc‐Cp), 108.6 (quart. carbon of subst. Cc‐Cp), 120.0 (CH of triazole), 149.4 (quart. carbon of triazole) ppm. IR (ATR): 3133 (ν_C‐H_), 1593, 1539, 1419 (ν_C=C_), 1259, 1125, 1028, 816 (ν_P−F_), 557 (ν_P−F_), 490, 446 cm^−1^; MS (ESI pos): *m*/*z* 440.03 (M^+^‐PF_6_. Single crystals of **6** were obtained from an acetone solution at room temperature. Supporting Information: Single crystal analysis, spectra and crystallographic details.

**Triazole (7)**. Compound **7** was prepared in a similar manner as described above for **5**. Starting materials: Azidocobaltocenium hexafluoridophosphate (0.200 g, 0.53 mmol, 1 equiv), ethinylcobaltocenium hexafluoridophosphate (0.191 g, 0.53 mmol, 1 equiv), CuSO_4_⋅5 H_2_O (0.133 g, 0.53 mmol, 1 equiv), sodium ascorbate (0.264 g, 1.33 mmol, 2.5 equiv). Workup under ambient conditions: Red solid Cu_2_O was filtered off, the precipitate was washed with three portions of acetonitrile and the organic solvents of the combined solutions were removed on a rotary evaporator, resulting in a yellow solid. This material was extracted with six portions of CH_2_Cl_2_ to remove dehydroascorbate by filtration. Solvent of the combined CH_2_Cl_2_ fractions was removed on a rotary evaporator, the residue was dissolved in acetonitrile and the product was crystallized from this solution by diffusion of diethyl ether at room temperature, affording orange crystalline **7** (0.287 g, 0.39 mmol) in 73.4 % yield. Compound **7** is air‐stable and soluble in dichloromethane, acetone, acetonitrile and dimethylsulfoxide. M.p: 230 °C; ^1^H‐NMR (300 MHz, C_3_D_6_O): *δ*=5.84 (s, 5 H, 4‐Cc‐Cp), 6.01 (s, 5 H, 1‐Cc‐Cp), 6.12 (pseudo‐t, 2 H, *J=*2.1 Hz, C3/C4 of subst. 4‐Cc‐Cp), 6.15 (pseudo‐t, 2 H, *J=*2.3 Hz, C3/C4 of subst. 1‐Cc‐Cp), 6.48 (pseudo‐t, 2 H, *J=*2.1 Hz, C2/C5 of subst. 4‐Cc‐Cp), 6.70 (pseudo‐t, 2 H, *J*=2.3 Hz, C2/C5 of subst. 1‐Cc‐Cp), 9.15 (s, 1 H, CH of triazole) ppm; ^13^C‐NMR (75 MHz, C_3_D_6_O): *δ*=77.0 (C3/C4 of subst. 4‐Cc‐Cp), 82.3 (C2/C5 of subst. 4‐Cc‐Cp), 83.8 (C3/C4 of subst. 1‐Cc‐Cp), 85.9 (C2/C5 of subst. 1‐Cc‐Cp), 87.1 (4‐Cc‐Cp), 87.7 (1‐Cc‐Cp), 90.0 (quart. carbon of subst. 4‐Cc‐Cp), 95.0 (quart. carbon of subst. 1‐Cc‐Cp), 125.4 (CH of triazole), 140.8 (quart. carbon of triazole) ppm; IR (ATR): 3121 (ν_C−H_), 1603, 1540, 1459, 1418 (ν_C=C_), 1208, 1048, 1024, 816 (ν_P−F_), 555 (ν_P−F_), 494, 479, 443 cm^−1^; MS (MALDI pos): *m*/*z* 588.01 (M^+^‐PF_6_). Single crystals of **7** were obtained from an acetonitrile/diethyl ether solution at room temperature. Supporting Information: Single crystal analysis, spectra and crystallographic details.

**Triazolium salt (8 a)**. A Schlenk tube was charged under an atmosphere of Ar with **5** (0.737 g, 1.26 mmol, 1 equiv), dry 1,2‐dichloroethane (30 mL) and methyl triflate (1.47 mL, 12.6 mmol, 10 equiv). The mixture was refluxed overnight resulting in a red solution. Workup: After cooling to 0 °C in an ice bath, dry diethyl ether (250 mL) was added and the mixture was placed in a freezer to precipitate the product. The red precipitate was filtered off under ambient conditions, washed three times with small portions of diethyl ether and dried in vacuo, affording 0.909 g (1.21 mmol) of **8 a** as a red powder in 95.8 % yield. Compound **8 a** is air‐stable and soluble in acetone and acetonitrile. M.p.: 231 °C; ^1^H‐NMR (300 MHz, C_3_D_6_O): *δ*=4.43 (s, 5 H, Fc‐Cp), 4.61 (m, 5 H, CH_3_ of triazolium and C3/C4 of subst. Fc‐Cp), 5.33 (pseudo‐t, 2 H, *J=*2.0 Hz, C2/C5 of subst. Fc‐Cp), 6.15 (s, 5 H, Cc‐Cp), 6.31 (pseudo‐t, 2 H, *J=*2.1 Hz, C3/C4 of subst. Cc‐Cp), 6.77 (pseudo‐t, 2 H, *J*=2.1 Hz, C2/C5 of subst. Cc‐Cp), 9.90 (s, 1 H, CH of triazolium) ppm; ^13^C‐NMR (75 MHz, C_3_D_6_O): *δ*=40.8 (CH_3_ of triazolium), 64.0 (C3/C4 of subst. Fc‐Cp), 69.7 (C2/C5 of subst. Fc‐Cp), 72.2 (Fc‐Cp), 85.9 (C3/C4 of subst. Cc‐Cp), 86.6 (quart. carbon of subst. Fc‐Cp), 87.3 (C2/C5 of subst. Cc‐Cp), 88.3 (Cc‐Cp), 92.7 (quart. carbon of subst. Cc‐Cp), 130.5 (CH of triazolium), 137.6 (quart. carbon of triazolium) ppm; IR (ATR): 3116 (ν_C‐H_), 1604, 1440, 1419 (ν_C=C_), 1255 (ν_SO3_), 1226 (ν_CF3_), 1154 (ν_CF3_), 1028 (ν_SO3_), 835 (ν_P‐F_), 636, 558 (ν_P‐F_), 508, 446 cm^−1^; MS (ESI pos): *m*/*z* 604.01 ([M(CF_3_SO_3_)]^+^). Single crystals of **8 a** were obtained from an acetone/diethyl ether solution at 4 °C. Supporting Information: Single crystal analysis, spectra and crystallographic details.

**Triazolium salt (8 b)**. Triazolium salt **8 a** (0.050 g, 0.07 mmol) was dissolved in a beaker in 5 mL of acetonitrile and the anions were converted to hexafluoridophosphate over a previously conditioned DOWEX Marathon A^TM^ anionic exchange column (conditioning terms: 200 mL of deionized water for swelling, followed by 200 mL acetonitrile). The column was slowly eluted with additional 50 mL of solvent, the red fractions were combined and concentrated on a rotary evaporator until a total volume of 3 mL was reached. The product was precipitated by adding 100 mL of diethyl ether and the precipitation process was facilitated at 0 °C. The resulting red powder was filtered off on a Büchner funnel, thoroughly washed three times with diethyl ether and dried in vacuo, yielding pure red powdery **8 b** in 96.1 % (0.047 g, 0.06 mmol). **8 b** is air‐stable and soluble in acetone and acetonitrile. M.p.: 275 °C; ^1^H‐NMR (300 MHz, C_3_D_6_O): *δ*=4.47 (s, 5 H, Fc‐Cp), 4.59 (s, 3 H, CH_3_ of triazolium), 4.63 (s, broad, 2 H, C3/C4 of substituted Fc‐Cp), 5.35 (s, broad, 2 H, *J=*2.0 Hz, C2/C5 of substituted Fc‐Cp), 6.14 (s, 5 H, Cc‐Cp), 6.29 (pseudo‐t, 2 H, *J=*1.7 Hz, C3/C4 of substituted Cc‐Cp), 6.73 (pseudo‐t, 2 H, *J*=1.7 Hz, C2/C5 of substituted Cc‐Cp), 9.83 (s, 1 H, CH of triazolium) ppm; ^13^C‐NMR (75 MHz, C_3_D_6_O): *δ*=40.7 (CH_3_ of triazolium), 64.0 (C3/C4 of substituted Fc‐Cp), 69.7 (C2/C5 of substituted Fc‐Cp), 72.2 (Fc‐Cp), 85.9 (C3/C4 of substituted Cc‐Cp), 86.6 (quart. carbon of substituted Fc‐Cp), 87.3 (C2/C5 of substituted Cc‐Cp), 88.3 (Cc‐Cp), 92.7 (quart. carbon of substituted Cc‐Cp), 130.4 (CH of triazolium), 137.6 (quart. carbon of triazolium) ppm; IR (ATR): 3160 (ν_C‐H_), 3127 (ν_C‐H_), 1600, 1441, 1418 (ν_C=C_), 812 (ν_P‐F_), 555 (ν_P‐F_), 500, 444, 427 cm^−1^; MS (ESI pos): *m*/*z* 600.01 ([M(PF_6_)]^+^). Supporting Information: spectra.

**Triazolium salt (9 a)**. Compound **9 a** was prepared in a similar manner as described above for **8 a**. Starting materials: **6** (0.140 g, 0.24 mmol, 1 equiv), dry dichloroethane (15 mL), methyl triflate (0.28 mL, 2.40 mmol, 10 equiv). Product: red powder, 0.161 g (0.22 mmol), 89.3 % yield. Compound **9 a** is air‐stable and soluble in acetone and acetonitrile. M.p.: 236 °C; ^1^H‐NMR (300 MHz, C_3_D_6_O): *δ*=4.39 (s, 5 H, Fc‐Cp), 4.60 (s, 3 H, CH_3_ of triazolium), 4.70 (pseudo‐t, 2 H, *J=*1.7 Hz, C3/C4 of subst. Fc‐Cp), 5.10 (pseudo‐t, 2 H, *J=*1.8 Hz, C2/C5 of subst. Fc‐Cp), 6.18 (s, 5 H, Cc‐Cp), 6.24 (pseudo‐t, 2 H, *J=*2.3 Hz, C3/C4 of subst. Cc‐Cp), 6.94 (pseudo‐t, 2 H, *J*=2.3 Hz, C2/C5 of subst. Cc‐Cp), 9.67 (s, 1 H, CH of triazole) ppm; ^13^C‐NMR (75 MHz, C_3_D_6_O): *δ* 40.8 (CH_3_ of triazole), 66.1 (quart. carbon of subst. Fc‐Cp), 70.3 (C3/C4 of subst. Fc‐Cp), 71.4 (Fc‐Cp), 72.5 (C2/C5 of subst. Fc‐Cp), 79.6 (C3/C4 of subst. Cc‐Cp), 84.9 (C2/C5 of subst. Cc‐Cp), 88.7 (Cc‐Cp), 105.3 (quart. carbon of subst. Cc‐Cp), 128.8 (CH of triazolium), 147.0 (quart. carbon of triazolium) ppm; IR (ATR): 3127 (ν_C−H_), 1601, 1521, 1420 (ν_C=C_), 1257 (ν_SO3_), 1224 (ν_CF3_), 1152 (ν_CF3_), 1031 (ν_SO3_), 827 (ν_P−F_), 636, 556 (ν_P−F_), 510, 443 cm^−1^; MS (MALDI pos): *m*/*z* 604.03 (M^+^‐PF_6_^−^). Single crystals of **9 a** were obtained from an acetone/diethyl ether solution at 4 °C. Single crystal analysis (Figure 2), Supporting Information: spectra and crystallographic details.

**Triazolium salt (9 b)**. Compound **9 b** was prepared in an analogous manner to **8 b**. Starting materials: Triazolium salt **9 a** (0.040 g, 0.05 mmol), 3 mL of acetonitrile for dissolving, 50 mL of acetonitrile for eluting the target compound. Product: red powder, 0.037 g (0.05 mmol), 94.2 % yield. Compound **9 b** is air‐stable and soluble in acetone and acetonitrile. M.p.: 247 °C; ^1^H‐NMR (300 MHz, C_3_D_6_O): *δ*=4.38 (s, 5 H, Fc‐Cp), 4.62 (s, 3 H, CH_3_ of triazolium), 4.74 (pseudo‐t, 2 H, *J=*1.8 Hz, C3/C4 of substituted Fc‐Cp), 5.05 (pseudo‐t, 2 H, *J=*1.8 Hz, C2/C5 of substituted Fc‐Cp), 6.15 (s, 5 H, Cc‐Cp), 6.25 (pseudo‐t, 2 H, *J=*2.3 Hz, C3/C4 of substituted Cc‐Cp), 6.87 (pseudo‐t, 2 H, *J*=2.3 Hz, C2/C5 of substituted Cc‐Cp), 9.49 (s, 1 H, CH of triazole) ppm; ^13^C‐NMR (75 MHz, C_3_D_6_O): *δ*=40.8 (CH_3_ of triazole), 66.0 (quart. carbon of substituted Fc‐Cp), 70.2 (C3/C4 of substituted Fc‐Cp), 71.3 (Fc‐Cp), 72.7 (C2/C5 of substituted Fc‐Cp), 79.6 (C3/C4 of substituted Cc‐Cp), 85.0 (C2/C5 of substituted Cc‐Cp), 88.8 (Cc‐Cp), 105.2 (quart. carbon of substituted Cc‐Cp), 128.7 (CH of triazolium), 147.0 (quart. carbon of triazolium) ppm; IR (ATR): 3156 (ν_C−H_), 3130 (ν_C−H_), 1600, 1519, 1434, 1419 (ν_C=C_), 824 (ν_P−F_), 555 (ν_P−F_), 510, 442 cm^−1^; MS (ESI pos): *m*/*z* 600.01 ([M(PF_6_)]^+^). Supporting Information: spectra.

**Triazolium salt (10 a)**. Compound **10 a** was prepared in a similar manner as described above for **8 a**. Starting materials: **7** (0.174 g, 0.24 mmol, 1 equiv), dry dichloroethane (20 mL), methyl triflate (0.69 mL, 5.93 mmol, 25 equiv); reflux period: 48 h. Product: yellow powder, 0.171 g (0.19 mmol), 79.5 % yield. Compound **10 a** is air‐stable and soluble in acetone, acetonitrile and dimethylsulfoxide. M.p.: 240 °C; ^1^H‐NMR (300 MHz, C_3_D_6_O): *δ*=4.68 (s, 3 H, CH_3_ of triazolium), 6.18 (s, 5 H, 4‐Cc‐Cp), 6.23 (s, 5 H, 1‐Cc‐Cp), 6.28 (pseudo‐t, 2 H, *J=*2.3 Hz, C3/C4 of subst. 1‐Cc‐Cp), 6.34 (pseudo‐t, 2 H, *J=*2.1 Hz, C3/C4 of subst. 4‐Cc‐Cp), 6.70 (pseudo‐t, 2 H, *J=*2.1 Hz, C2/C5 of subst. 4‐Cc‐Cp), 6.93 (pseudo‐t, 2 H, *J*=2.3 Hz, C2/C5 of subst. 1‐Cc‐Cp), 10.14 (s, 1 H, CH of triazolium) ppm; ^13^C‐NMR (75 MHz, C_3_D_6_O): *δ*=41.5 (CH_3_ of triazolium), 79.9 (C3/C4 of subst. 4‐Cc‐Cp), 85.0 (C3/C4 of subst. 1‐Cc‐Cp), 85.9 (quart. carbon of subst. 4‐Cc‐Cp), 86.5 (C2/C5 of subst. 4‐Cc‐Cp), 87.4 (C2/C5 of subst. 1‐Cc‐Cp), 88.5 (4‐Cc‐Cp), 89.0 (1‐Cc‐Cp), 105.0 (quart. carbon of subst. 1‐Cc‐Cp), 119.9 (CF_3_ of triflate), 124.1 (CF_3_ of triflate), 132.6 (CH of triazolium), 138.8 (quart. carbon of triazolium) ppm; IR (ATR): 3115 (ν_C‐H_), 1525, 1466, 1417 (ν_C=C_), 1254 (ν_SO3_), 1224 (ν_CF3_), 1151 (ν_CF3_), 1028 (ν_SO3_), 835 (ν_P−F_), 634, 572 (ν_P−F_), 516, 500, 473, 442 cm^−1^; MS (MALDI pos): *m*/*z* 755.96 ([M(CF_3_SO_3_)_2_]^+^). Single crystals of **10 a** were obtained from an acetone/diethyl ether solution at 4 °C. Supporting Information: Single crystal analysis, spectra and crystallographic details.

**Triazolium salt (10 b)**. Compound **10 b** was prepared in an analogous manner to **8 b**. Starting materials: Triazolium salt **10 a** (0.040 g, 0.04 mmol), 3 mL of acetonitrile for dissolving, 50 mL of acetonitrile for eluting the target compound. Product: yellow powder, 0.038 g (0.04 mmol), 95.6 % yield. Compound **10 b** is air‐stable and soluble in acetone, acetonitrile and dimethoxysulfoxide. M.p.: 218 °C; ^1^H‐NMR (300 MHz, C_3_D_6_O): *δ*=4.71 (s, 3 H, CH_3_ of triazolium), 6.12 (s, 5 H, 4‐Cc‐Cp), 6.18 (s, 5 H, 1‐Cc‐Cp), 6.29 (pseudo‐t, 2 H, *J=*2.3 Hz, C3/C4 of substituted 1‐Cc‐Cp), 6.36 (pseudo‐t, 2 H, *J=*2.3 Hz, C3/C4 of substituted 4‐Cc‐Cp), 6.61 (pseudo‐t, 2 H, *J=*2.3 Hz, C2/C5 of substituted 4‐Cc‐Cp), 6.83 (pseudo‐t, 2 H, *J*=2.4 Hz, C2/C5 of substituted 1‐Cc‐Cp), 9.86 (s, 1 H, CH of triazolium) ppm; ^13^C‐NMR (75 MHz, C_3_D_6_O): *δ*=41.5 (CH_3_ of triazolium), 80.0 (C3/C4 of substituted 4‐Cc‐Cp), 85.2 (C3/C4 of substituted 1‐Cc‐Cp), 85.5 (quart. carbon of substituted 4‐Cc‐Cp), 86.1 (C2/C5 of substituted 4‐Cc‐Cp), 87.8 (C2/C5 of substituted 1‐Cc‐Cp), 88.4 (4‐Cc‐Cp), 88.8 (1‐Cc‐Cp), 104.9 (quart. carbon of substituted 1‐Cc‐Cp), 132.5 (CH of triazolium), 138.6 (quart. carbon of triazolium) ppm; IR (ATR): 3127 (ν_C‐H_), 2921, 2851, 1611, 1529, 1469, 1418 (ν_C=C_), 817 (ν_P‐F_), 554 (ν_P‐F_), 499, 473, 437 cm^−1^; MS (ESI pos): *m*/*z* 747.97 ([M(PF_6_)_2_]^+^). Supporting Information: spectra.

**Triazolylidene complex (11)**. A Schlenk flask equipped with a reflux condenser was charged with 15 mL of dry acetonitrile, 0.100 g of **8 a** (0.13 mmol, 1 equiv), 0.154 g of Ag_2_O (0.66 mmol, 5 equiv), 0.099 g of KCl (1.33 mmol, 10 equiv) and 0.170 g of molecular sieve (4 Å) under exclusion of light and under protection from air by an argon atmosphere. After stirring for 72 h at 50 °C, the black solid was filtered off by syringe filtration under argon atmosphere and the product was precipitated by adding 130 mL of diethyl ether. Precipitation was facilitated by placing the flask in an ice bath. The resulting ochre powder was filtered off on a Büchner funnel, thoroughly washed three times with diethyl ether and dried in vacuo, affording 0.075 g of ochre powdery **11** in 75.1–88.5 % yield. Note: Yield is reported in a range, depending on relative ratio of counterions PF_6_^−^, F_3_CSO_3_^−^, Cl^−^). The solid product can be handled under air but was stored under argon atmosphere and exclusion of light. To remove traces of KCl, the product was dissolved in 12 mL of dry acetone at 0 °C and the inorganic residues were filtered off via syringe filtration. **11** is soluble in DMSO, methanol, acetonitrile and slightly in dichloromethane. M.p.: 150 °C; ^1^H‐NMR (300 MHz, C_3_D_6_O): *δ*=4.42 (s, 5 H, Fc‐Cp), 4.46 (s, 3 H, CH_3_ of triazolium), 4.49 (pseudo‐t, 2 H, *J*=2.0 Hz, C3/C4 of subst. Fc‐Cp), 5.25 (pseudo‐t, 2 H, *J*=2.0 Hz, C2/C5 of subst. Fc‐Cp), 6.07 (s, 5 H, Cc‐Cp), 6.22 (pseudo‐t, 2 H, *J*=1.7 Hz, C3/C4 of subst. Cc‐Cp), 6.72 (pseudo‐t, 2 H, *J*=2.0 Hz, C2/C5 of subst. Cc‐Cp) ppm; ^13^C‐NMR (75 MHz, C_3_D_6_O): *δ*=39.4 (CH_3_ of triazolium), 64.7 (C3/C4 of subst. Fc‐Cp), 68.8 (C2/C5 of subst. Fc‐Cp), 71.9 (Fc‐Cp), 72.3 (quart. carbon of subst. Fc‐Cp), 85.0 (C3/C4 of subst. Cc‐Cp), 86.1 (C2/C5 of subst. Cc‐Cp), 87.9 (Cc‐Cp), 88.2 (quart. carbon of subst. Fc‐Cp), 104.2 (quart. carbon of subst. Cc‐Cp), 131.0 (quart. carbon of triazolium), 166.4 (carbene carbon of triazolium) ppm; IR (ATR): 3114 (ν_C−H_), 1625, 1451, 1418 (ν_C=C_), 1253 (ν_SO3_), 1228 (νCF3), 1163 (ν_CF3_), 1030 (νSO3), 840 (ν_P−F_), 639, 575 (ν_P−F_), 515, 485, 448 cm^−1^; MS (ESI pos): *m*/*z* 597.91 (M^+^‐CF_3_SO_3_^−^). Single crystals of **11** were obtained by diffusion‐crystallization from acetonitrile/diethyl ether at 4 °C. Single crystal analysis (Figure 3), Supporting Information: spectra and crystallographic details.

**Triazolylidene complex (12)**. A Schlenk flask equipped with a reflux condenser was charged with 10 mL of dry acetonitrile, 0.050 g of **10 a** (0.06 mmol, 1 equiv), 0.040 g of Cu_2_O (0.28 mmol, 5 equiv), 0.042 g of KCl (0.56 mmol, 10 equiv) and 0.085 g of molecular sieve (4 Å) under exclusion of light and under protection from air by an argon atmosphere. The reaction mixture was stirred for 72 h at 50 °C. The solvent was removed in vacuo and the product was extracted with cold dry acetone. Red Cu_2_O and inorganic salts were removed by syringe filtration. The solvent was removed and the orange‐yellow product was dried in vacuo. To remove inorganic salts the product was crystallized by diffusion‐crystallization from acetone/diethyl ether at 4 °C, leading to large rhombohedral orange crystrals accompanied by small colorless inorganic crystals. The inorganic salts were removed by size‐exclusion filtration on a #3 Büchner funnel and the product was dried in vacuo, leading to a yield of 26.6–36.2 % (0.013 g). Note 1: Yield is reported in a range, depending on relative ratio of counterions PF_6_^−^, F_3_CSO_3_^−^, Cl^−^). Note 2: NMR spectroscopy showed that **12** occurred in a product/educt ratio of 60/40. The solid product can be handled under air but was stored under argon atmosphere and exclusion of light. **12** is soluble in DMSO, methanol, acetonitrile and slightly in dichloromethane. M.p.: 214 °C; ^1^H‐NMR (300 MHz, C_3_D_6_O): *δ*=4.49 (s, 3 H, CH_3_ of triazolium), 6.13 (s, 5 H, 4‐Cc‐Cp), 6.17 (s, 5 H, 1‐Cc‐Cp), 6.19 (shoulder, 2 H, C3/C4 of subst. 1‐Cc‐Cp), 6.23 (shoulder, 2 H, C3/C4 of subst. 4‐Cc‐Cp), 6.64 (pseudo‐t, 2 H, *J*=1.8 Hz, C2/C5 of subst. 4‐Cc‐Cp), 6.87 (pseudo‐t, 2 H, *J*=1.8 Hz, C2/C5 of subst. 1‐Cc‐Cp) ppm; ^13^C‐NMR (75 MHz, C_3_D_6_O): *δ*=41.4 (CH_3_ of triazolium), 76.3 (C3/C4 of subst. 4‐Cc‐Cp), 80.7 (C3/C4 of subst. 1‐Cc‐Cp), 83.0 (C2/C5 of subst. 4‐Cc‐Cp), 85.1 (C2/C5 of subst. 1‐Cc‐Cp), 88.3 (4‐Cc‐Cp), 88.6 (1‐Cc‐Cp), not observed: (quart. carbon of subst. 4‐Cc‐Cp), 105.3 (quart. carbon of subst. 1‐Cc‐Cp), 120.0 (CF_3_ of triflate), 124.3 (CF_3_ of triflate), 138.9 (quart. carbon of triazolium), 160.3 (carbene carbon of triazolium) ppm; IR (ATR): 3119 (ν_C−H_), 2961, 2925, 2854, 1705, 1668, 1620, 1554, 1419 (ν_C=C_), 1248 (ν_SO3_), 1228 (ν_CF3_), 1161 (ν_CF3_), 1030 (ν_SO3_), 841 (ν_P−F_), 766, 635, 577 (ν_P−F_), 559, 518, 476 cm^−1^; MS (ESI pos): *m*/*z* 703.88 ([M(CF_3_SO_3_)]^+^). Single crystals of **12** were obtained from acetone/diethyl ether at 4 °C. Single crystal analysis (Figure 4), Supporting Information: spectra and crystallographic details.

**Triazolylidene complex (13)**. A Schlenk flask equipped with a reflux condenser was charged with 15 mL of dry acetonitrile, 0.041 g of AuCl (0.18 mmol, 2 equiv), 0.030 g of CsCl (0.18 mmol, 2 equiv) and the reaction mixture was refluxed overnight under exclusion of light and under protection from air by an argon atmosphere. Parallel to this reaction, a 25 mL round bottom Schlenk flask also equipped with a reflux condenser was charged under argon atmosphere with 10 mL of dry acetonitrile, 0.080 g of **10 a** (0.09 mmol, 1 equiv) and 0.102 g of Ag_2_O (0.44 mmol, 5 equiv). The reaction mixture was stirred at 50 °C overnight under exclusion of light. Both solutions were allowed to cool to room temperature and the silver complex solution was added by syringe filtration to the Schlenk flask containing Cs[AuCl_2_], retaining thereby the excess of Ag_2_O. Immediately a precipitation of white AgCl was observed and the yellow reaction mixture was stirred under exclusion of light at room temperature overnight. The precipitated AgCl was filtered off by syringe filtration and the solvent was removed in vacuo. The yellow product was extracted with cold dry acetone and further precipitated with diethyl ether, leading to a sticky yellow product. The solvent was decanted, the product washed two times with diethyl ether and dried in vacuo, yielding 0.067 g of **13** as a yellow oily foam in 75.6–98.1 % yield. Note: Yield is reported in a range, depending on relative ratio of counterions PF_6_^−^, F_3_CSO_3_^−^, Cl^−^). The solid product can be handled under air but was stored under argon atmosphere and exclusion of light. **13** is soluble in DMSO, methanol, acetonitrile and slightly in dichloromethane. M.p.: 161 °C; ^1^H‐NMR (300 MHz, C_3_D_6_O): *δ*=4.50 (s, 3 H, CH_3_ of triazolium), 6.12 (s, 5 H, 4‐Cc‐Cp), 6.18 (pseudo‐t, 2 H, *J*=2.1 Hz, C3/C4 of subst. 1‐Cc‐Cp), 6.19 (s, 5 H, 1‐Cc‐Cp), 6.22 (pseudo‐t, 2 H, *J*=1.8 Hz, C3/C4 of subst. 4‐Cc‐Cp), 6.66 (pseudo‐t, 2 H, *J*=2.0 Hz, C2/C5 of subst. 4‐Cc‐Cp), 6.91 (pseudo‐t, 2 H, *J*=2.3 Hz, C2/C5 of subst. 1‐Cc‐Cp) ppm; ^13^C‐NMR (75 MHz, C_3_D_6_O): *δ*=40.7 (CH_3_ of triazolium), 81.0 (C3/C4 of subst. 4‐Cc‐Cp), 84.1 (C3/C4 of subst. 1‐Cc‐Cp), 86.0 (C2/C5 of subst. 4‐Cc‐Cp), 86.0 (C2/C5 of subst. 1‐Cc‐Cp), 88.4 (4‐Cc‐Cp), 88.7 (1‐Cc‐Cp), 92.2 (quart. carbon of subst. 4‐Cc‐Cp), 109.5 (quart. carbon of subst. 1‐Cc‐Cp), 120.0 (CF_3_ of triflate), 124.3 (CF_3_ of triflate), 141.0 (quart. carbon of triazolium), 162.2 (carbene carbon of triazolium) ppm; IR (ATR): 3099 (ν_C−H_), 1706, 1415 (ν_C=C_), 1362, 1255 (ν_SO3_), 1224 (ν_CF3_), 1149 (ν_CF3_), 1027 (ν_SO3_), 865 (ν_P−F_), 757, 635, 573 (ν_P−F_), 506, 480, 434 cm^−1^; MS (ESI pos): *m*/*z* 837.93 ([M(CF_3_SO_3_)]^+^). Single crystals of **13** were obtained from acetone/diethyl ether at 4 °C. Single crystal analysis (Figure 5), Supporting Information: spectra and crystallographic details.

### DFT calculations

X‐ray crystal structures of **8 a**–**10 a** were subject to structure optimization. Density functional theory calculations were performed with the quantum chemical program package Turbomole.14a Schäfer et al.“s triple‐zeta basis14b was used in combination with two density functionals, the pure BP8614c,14d as well as the hybrid PBE014e functional with 25 % Hartree–Fock exchange. For BP86 calculations, the resolution‐of‐identity technique was invoked to reduce computational costs.14f The effect of Grimme's empirical dispersion corrections of the Becke‐Johnson type was also tested.14g,14h Solvation was treated implicitly by the conductor‐like‐screening model (COSMO) as implemented in Turbomole,^15^ where the solvent is described by a dielectric continuum modelled by a dielectric constant *ϵ* and the solute is placed in a cavity in this continuum. All structures were fully optimized and verified as (local) energy minima by calculation of the Hesse matrix, i.e., the second derivatives of energy with respect to coordinates of each molecule, which yielded only positive eigenvalues. Electronic energies were converted to Gibb's free energies Δ*G* by adding zero‐point energy and thermal corrections via the rigid‐rotator harmonic oscillator approximation. Scaling factors had little effect on the obtained results (p*K*_a_ values) and where, thus, not used. For the calculations of the molecular electrostatic potential (MEP) of **8′**–**10′**, a density isovalue of 0.01 a.u. was chosen. Tolman electronic parameters (TEPs) were obtained according to a parametrization Scheme by Mathew and Sureh,^17^ for which the TEP is determined by the quantum chemically derived molecular electrostatic potential at the carbene (V_c_) and does not require the sometimes cumbersome experimental determination of the TEP value.17a TEP values of two recently reported reference carbenes (with known experimental TEP values)^21^ were calculated to prove the validity of this approach (compare Supporting Information). TEPs were obtained as single point calculations on the BP86/def2‐TZVP optimized structures using the density functionals B3LYP,22a in combination with the Pople type basis set 6–311++G(d,p).22b For comparison, Gaussian09 was used.^23^ pK_a_ values were calculated in water, treated as implicit solvent, for both density functionals (BP86 and PBE0) in combination with empirical dispersion corrections and without dispersion corrections according to the thermodynamic cycle depicted in Scheme S1. For the free energy of solvation, Δ*G*(solv) (H^+^), the literature value of −259.80 kcal mol^−1^ was used^24^ whereas the gas phase free energy of H^+^, G(g) (H^+^)=−6.29 kcal mol^−1^, was obtained from the Sackur‐Tetrode equation and translational energy at 298 K.^25^

### Electrochemical measurements

Cyclic voltammograms were recorded with a PAR VersaStat 4 potentiostat (Ametek) by working in anhydrous and degassed CH_3_CN with either 0.1 m NBu_4_PF_6_ or 0.1 m NBu_4_BAr^F^ as supporting electrolyte. A three‐electrode setup was used with a glassy carbon working electrode, a coiled platinum wire as counter electrode, and a coiled silver wire as a pseudoreference electrode. The ferrocene/ferrocenium couple was used as internal reference.

**Catalytic studies**. The complex **13** (1 mol %), *N*‐(2‐propyn‐1‐yl)benzamide (0.2 mmol), and hexadecane (10 mol %) were dissolved in absolute dichloromethane (2 mL) and stirred at room temperature under an inert atmosphere. Samples were taken after different reaction times. For that, 0.05 mL of the reaction solution was taken with a syringe, the solvent evaporated in a stream of argon, and the conversions to the product 5‐methylene‐2‐phenyl‐4,5‐dihydrooxazole were detected by ^1^H‐NMR spectroscopy with hexadecane as internal standard. To test redox‐switchability, the reducing agent, cobaltocene (2.6 mol %) was added before the addition of the substrate and the reaction solution was stirred for an hour. With the reduced catalyst, no conversion to product 5‐methylene‐2‐phenyl‐4,5‐dihydrooxazole was detected after 30 hours.

**Cytotoxicity studies**. The cytotoxic effects were determined according to a standard procedure, which had also been applied for other cobaltocenium compounds.^**7**^ In short: Cells were grown in 96‐well flat bottom microtiter plates, the cell cultu**r**e media were replaced with media containing the compounds in graded concentrations (0.1 % DMF v/v) and incubated for 72 h at 37 °C/5 % CO_2_. Afterwards the cell biomass was determined by crystal violet staining and the IC_50_ values were calculated as the concentration reducing cell growth by 50 % compared to an untreated control.

https://summary.ccdc.cam.ac.uk/structure-summary?doi=10.1002/chem.201705051 1580972, 1580973, 1580974, 1580975, 1580976, 1580977, 1580978, 1580979, 1580980 contain the supplementary crystallographic data for this paper. These data are provided free of charge by The Cambridge Crystallographic Data Centre.

## Conflict of interest

The authors declare no conflict of interest.

## Supporting information

As a service to our authors and readers, this journal provides supporting information supplied by the authors. Such materials are peer reviewed and may be re‐organized for online delivery, but are not copy‐edited or typeset. Technical support issues arising from supporting information (other than missing files) should be addressed to the authors.

Supplementary
