# Supplementary material for: Highly Electrophilic, Catalytically Active and Redox‐Responsive Cobaltoceniumyl and Ferrocenyl Triazolylidene Coinage Metal Complexes
Source: Chemistry. 2018 Jan 17;24(15):3742–53. doi: 10.1002/chem.201705051 (PMC6100101; doi:10.1002/chem.201705051)
Supplement: Supplementary file 1 — Supplementary [file CHEM-24-3742-s001.pdf]

CHEMISTRY
A European Journal

Supporting Information

Highly Electrophilic, Catalytically Active and Redox-Responsive Cobaltoceniumyl and Ferrocenyl Triazolylidene Coinage Metal Complexes

Stefan Vanicek,[a] Maren Podewitz,[a] Jessica Stubbe,[b] Dennis Schulze,[b] Holger Kopacka,[a] Klaus Wurst,[a] Thomas Müller,[c] Petra Lippmann,[d] Simone Haslinger,[a] Herwig Schottenberger,[a] Klaus R. Liedl,[a] Ingo Ott,[d] Biprajit Sarkar,*[b] and Benno Bildstein*[a]

chem_201705051_sm_miscellaneous_information.pdf

Supporting Information

Analytical Section (S1 – S167)

Triazole (5) S1

Triazole (6) S20

Triazole (7) S32

Triazolium salt (8a) S45

Triazolium salt (8b) S58

Triazolium salt (9a) S62

Triazolium salt (9b) S89

Triazolium salt (10a) S93

Triazolium salt (10b) S116

Triazolylidene complexe (11) S120

Triazolylidene complexe (12) S140

Triazolylidene complexe (13) S154

Computational Section (S168 – S174)

Electrochemical Section (S175 – S183)

Catalytical Section (S184)

References (S185)

Analytical Section

Triazole (5) – "4-cobaltoceniumyl-1-ferrocenyltriazole hexafluoridophosphate"

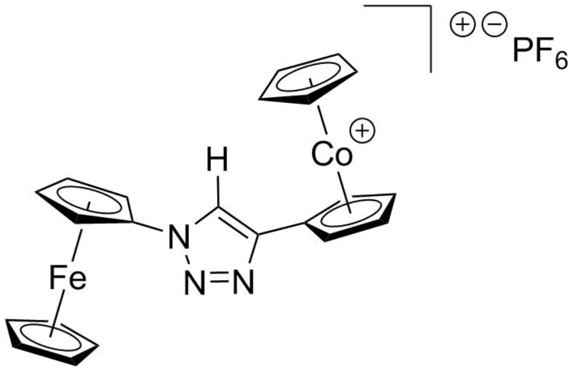

[585.15]

$C_{22}H_{19}N_3CoFe(PF_6)$

Analytical data:

IR (ATR [cm^{-1}]): 3117 (ν_{C-H}), 1590, 1525, 1452, 1420 ($\nu_{C=C}$), 1228, 1185, 1106, 1050, 1004, 808 (ν_{P-F}), 557 (ν_{P-F}), 508, 484, 454.

^{1}H-NMR (300 MHz, C_3D_6O, [ppm]): δ 4.27 (s, 5H, Fc-Cp), 4.40 (pseudo-t, 2H, *J* = 2.0 Hz, C3/C4 of substituted Fc-Cp), 5.04 (pseudo-t, 2H, *J* = 2.0 Hz, C2/C5 of substituted Fc-Cp), 5.80 (s, 5H, Cc-Cp), 6.05 (pseudo-t, 2H, *J* = 2.1 Hz, C3/C4 of substituted Cc-Cp), 6.46 (pseudo-t, 2H, J = 2.1 Hz, C2/C5 of substituted Cc-Cp), 8.93 (s, 1H, CH of triazole).

^{13}C-NMR (75 MHz, C_3D_6O, [ppm]): δ 62.9 (C3/C4 of substituted Fc-Cp), 67.9 (C2/C5 of substituted Fc-Cp), 71.1 (Fc-Cp), 81.8 (C3/C4 of substituted Cc-Cp), 85.5 (C2/C5 of substituted Cc-Cp), 86.9 (Cc-Cp), 94.3 (quart. carbon of substituted Fc-Cp), 96.7 (quart. carbon of substituted Cc-Cp), 124.3 (CH of triazole), 139.1 (quart. carbon of triazole).

MS (ESI pos, [m/z]): 440.02 (M^+ - PF_6^-).

Melting point [°C]: 177.

Spectra

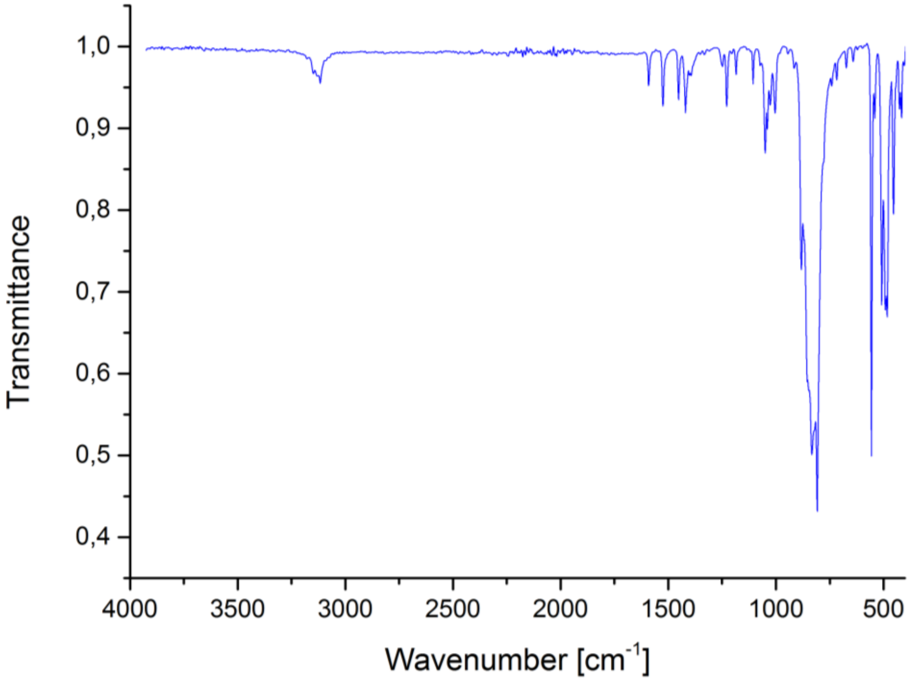

Figure S1. IR-spectrum (ATR, [cm^{-1}]) of "4-cobaltoceniumyl-1-ferrocenyltriazole hexafluoridophosphate".

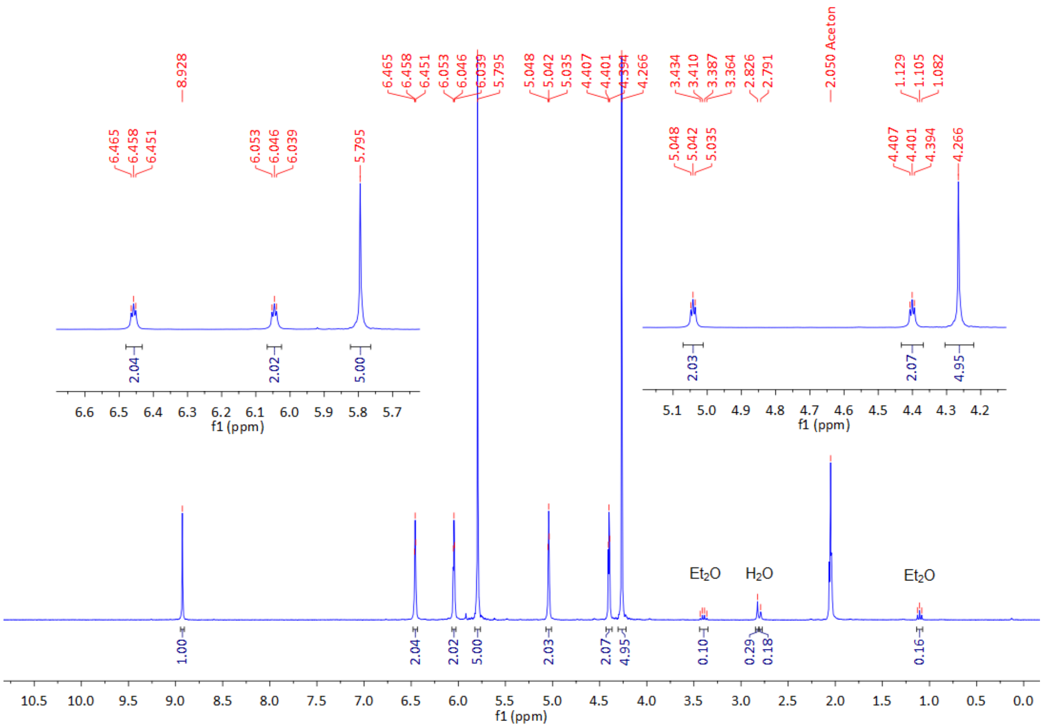

Figure S2. ^{1}H-NMR (300 MHz, C_3D_6O, [ppm]) of "4-cobaltoceniumyl-1-ferrocenyltriazole hexafluoridophosphate".

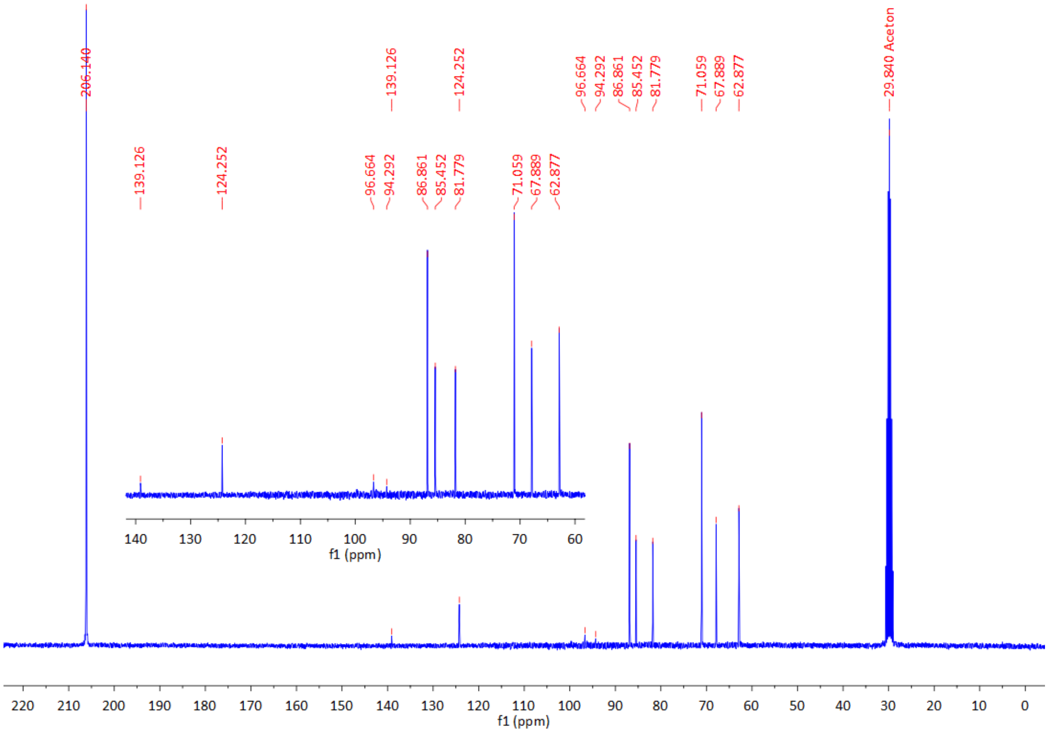

Figure S3. ^{13}C-NMR (75 MHz, C_3D_6O, [ppm]) of "4-cobaltoceniumyl-1-ferrocenyltriazole hexafluoridophosphate".

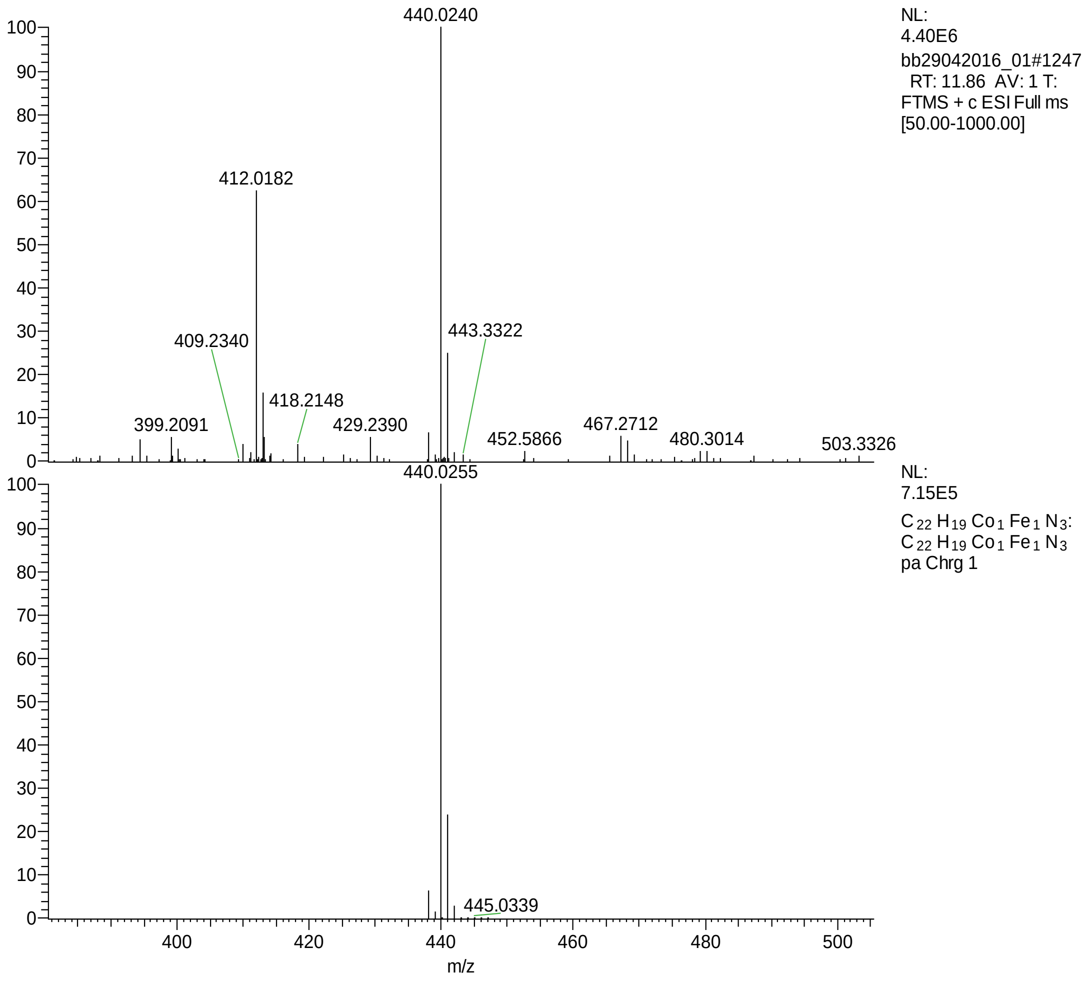

Figure S4. MS (ESI pos, [m/z]; *top*: experimental, *bottom*: simulated) of “4-cobaltoceniumyl-1-ferrocenyltriazole hexafluoridophosphate“.

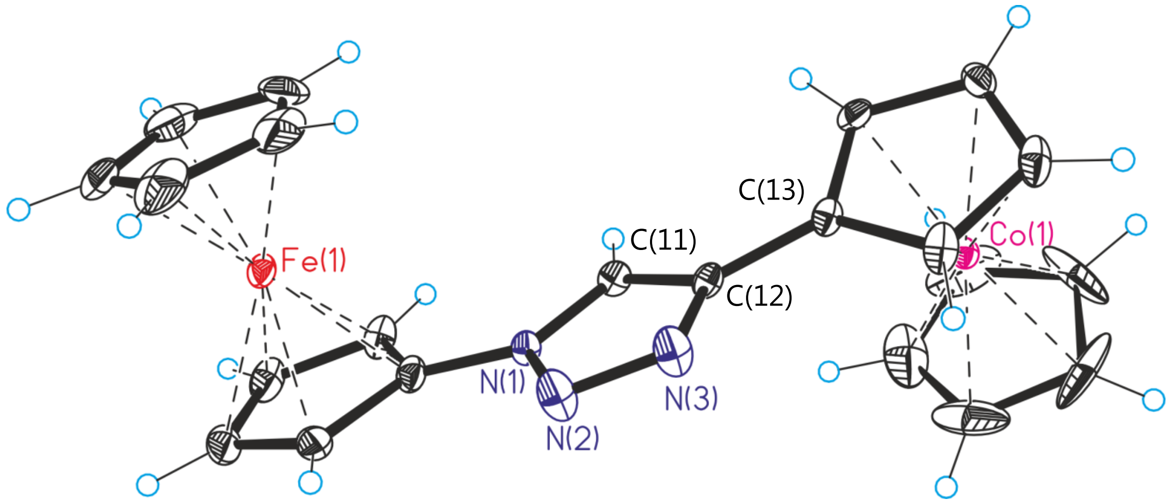

Figure S5. X-ray single crystal structure analysis of “4-cobaltoceniumyl-1-ferrocenyltriazole hexafluoridophosphate“ (counterion omitted for clarity).

X-Ray single crystal structure analysis data

Table S1. Crystal data and structure refinement for **5**.

Empirical formula	C22 H19 Co F6 Fe N3 P x CH2CL2	
Formula weight	670.08	
Temperature	173(2) K	
Wavelength	0.71073 Å	
Crystal system	Monoclinic	
Space group	Cc (no. 9)	
Unit cell dimensions	a = 14.8887(6) Å	α = 90°.
	b = 14.7993(6) Å	ß = 106.7920(10)°.
	c = 24.3468(9) Å	γ = 90°.
Volume	5135.9(4) Å^3	
Z	8	
Density (calculated)	1.733 Mg/m^3	
Absorption coefficient	1.544 mm^{-1}	
F(000)	2688	
Crystal size	0.150 x 0.090 x 0.060 mm^3	
Theta range for data collection	2.328 to 24.998°.	
Index ranges	-17<h<17, -17<k<17, -28<l<28	
Reflections collected	42432	
Independent reflections	8992 [R(int) = 0.0404]	
Completeness to theta = 24.998°	99.9 %	
Absorption correction	Semi-empirical from equivalents	
Max. and min. transmission	0.798 and 0.687	
Refinement method	Full-matrix least-squares on F^2	
Data / restraints / parameters	8992 / 2 / 730	
Goodness-of-fit on F^2	1.096	
Final R indices [I>2sigma(I)]	R1 = 0.0346, wR2 = 0.0688	
R indices (all data)	R1 = 0.0380, wR2 = 0.0700	
Absolute structure parameter	0.017(6)	
Extinction coefficient	n/a	
Largest diff. peak and hole	0.384 and -0.484 e.Å^{-3}	

Table S2. Atomic coordinates (x 10^4) and equivalent isotropic displacement parameters ($Å^2$x 10^3) for **5**. U(eq) is defined as one third of the trace of the orthogonalized U^{ij} tensor.

	x	y	z	U(eq)
Co(1)	1865(1)	2398(1)	2343(1)	19(1)
Co(2)	1686(1)	7328(1)	2008(1)	19(1)
Fe(1)	-240(1)	109(1)	4605(1)	24(1)
Fe(2)	5748(1)	10049(1)	4387(1)	23(1)
N(1)	635(3)	308(3)	3610(2)	20(1)
N(2)	1340(4)	-253(3)	3605(3)	38(1)
N(3)	1945(4)	226(3)	3423(2)	35(1)
N(4)	4429(3)	9121(3)	3322(2)	21(1)
N(5)	4899(3)	8401(3)	3208(2)	34(1)
N(6)	4275(3)	7766(3)	3005(2)	35(1)
C(1)	320(8)	1208(6)	5062(3)	67(3)
C(2)	-418(6)	854(6)	5267(3)	53(2)
C(3)	-157(7)	-10(6)	5456(3)	48(2)
C(4)	688(7)	-216(7)	5378(3)	59(2)
C(5)	999(6)	518(7)	5139(3)	61(3)
C(6)	-929(4)	518(5)	3797(2)	29(1)
C(7)	-1516(5)	-41(5)	4018(3)	36(2)
C(8)	-1084(5)	-894(5)	4152(3)	38(2)
C(9)	-204(5)	-880(4)	4022(2)	31(1)
C(10)	-137(4)	-3(4)	3801(2)	25(1)
C(11)	788(4)	1141(4)	3435(2)	22(1)
C(12)	1619(4)	1077(4)	3310(2)	21(1)
C(13)	2166(4)	1770(4)	3123(2)	22(1)
C(14)	2098(4)	2725(4)	3181(2)	27(1)
C(15)	2774(4)	3145(4)	2957(2)	30(1)
C(16)	3255(4)	2454(5)	2755(3)	34(2)
C(17)	2885(4)	1613(4)	2853(2)	29(1)
C(18)	525(6)	2171(9)	1890(4)	71(3)
C(19)	720(7)	3034(7)	1862(4)	74(3)
C(20)	1449(8)	3109(7)	1618(4)	85(4)
C(21)	1682(6)	2231(10)	1503(3)	82(4)
C(22)	1073(9)	1683(6)	1687(4)	75(4)
C(31)	5433(6)	9522(6)	5077(3)	53(2)
C(32)	6126(7)	10169(6)	5259(3)	61(3)
C(33)	6892(6)	9891(7)	5080(4)	65(3)
C(34)	6670(6)	9069(7)	4780(4)	62(2)
C(35)	5761(7)	8848(5)	4774(4)	59(2)
C(36)	4529(4)	10576(4)	3864(2)	27(1)
C(37)	5225(5)	11260(4)	4037(3)	35(2)
C(38)	6009(5)	11022(4)	3857(3)	32(2)
C(39)	5822(4)	10178(4)	3564(3)	26(1)
C(40)	4904(4)	9916(4)	3573(2)	23(1)

C(41)	3510(4)	8948(3)	3191(2)	20(1)
C(42)	3408(4)	8079(3)	2989(2)	19(1)
C(43)	2575(4)	7514(4)	2815(2)	22(1)
C(44)	1642(4)	7769(4)	2791(3)	27(1)
C(45)	1062(4)	6996(5)	2611(2)	34(2)
C(46)	1614(5)	6281(4)	2525(3)	36(2)
C(47)	2552(4)	6589(4)	2645(2)	26(1)
C(48)	2249(6)	7838(8)	1421(3)	71(3)
C(49)	1548(9)	8383(6)	1469(3)	72(3)
C(50)	739(7)	7910(7)	1337(3)	63(3)
C(51)	904(5)	7049(5)	1200(3)	43(2)
C(52)	1852(6)	6958(6)	1249(3)	54(2)
P(1)	-1004(1)	-266(1)	1922(1)	32(1)
F(1)	1(3)	-260(3)	1818(2)	51(1)
F(2)	-2014(3)	-261(3)	2028(2)	60(1)
F(3)	-689(3)	555(3)	2355(2)	60(1)
F(4)	-1307(4)	-1090(4)	1501(3)	97(2)
F(5)	-626(4)	-950(4)	2440(3)	92(2)
F(6)	-1368(4)	431(4)	1413(2)	73(2)
P(2)	3886(1)	10254(1)	1573(1)	30(1)
F(11)	3903(4)	9204(3)	1613(4)	103(2)
F(12)	3874(5)	11306(3)	1514(4)	125(3)
C(23)	2616(8)	8430(6)	4857(5)	77(3)
Cl(1)	3216(2)	7995(3)	4408(1)	112(1)
Cl(2)	1977(2)	7595(2)	5079(1)	79(1)
F(7)	2885(6)	10311(6)	1665(7)	81(4)
F(8)	4883(6)	10204(6)	1476(5)	63(3)
F(9)	4349(9)	10494(9)	2203(4)	103(3)
F(10)	3410(11)	10042(10)	924(5)	114(5)
C(24)	-1512(16)	3018(17)	329(7)	97(7)
Cl(3)	-387(3)	2596(3)	341(2)	82(1)
Cl(4)	-2138(7)	3260(5)	-386(4)	65(2)
F(7A)	2950(20)	10323(19)	1094(15)	105(11)
F(8A)	4836(16)	10128(15)	2074(15)	106(11)
F(9A)	3350(30)	10104(15)	2025(12)	107(12)
F(10A)	4410(30)	10400(20)	1122(17)	151(16)
C(24A)	-1150(30)	3400(30)	291(18)	68(11)
Cl(3A)	-974(9)	2291(7)	534(4)	102(4)
Cl(4A)	-2008(18)	3574(15)	-347(10)	91(7)

Table S3. Bond lengths [Å] and angles [°] for **5**.

Co(1)-C(22)	1.994(8)	Co(1)-C(19)	2.002(8)
Co(1)-C(20)	1.995(7)	Co(1)-C(18)	2.009(8)
Co(1)-C(21)	1.999(7)	Co(1)-C(16)	2.024(6)

Co(1)-C(14)	2.026(5)	C(3)-H(3)	0.9500
Co(1)-C(17)	2.028(5)	C(4)-C(5)	1.375(13)
Co(1)-C(15)	2.030(6)	C(4)-H(4)	0.9500
Co(1)-C(13)	2.044(5)	C(5)-H(5)	0.9500
Co(2)-C(48)	2.001(7)	C(6)-C(10)	1.406(9)
Co(2)-C(52)	2.009(6)	C(6)-C(7)	1.418(9)
Co(2)-C(49)	2.011(7)	C(6)-H(6)	0.9500
Co(2)-C(45)	2.012(6)	C(7)-C(8)	1.412(10)
Co(2)-C(50)	2.016(7)	C(7)-H(7)	0.9500
Co(2)-C(51)	2.017(6)	C(8)-C(9)	1.435(9)
Co(2)-C(46)	2.019(6)	C(8)-H(8)	0.9500
Co(2)-C(47)	2.027(6)	C(9)-C(10)	1.419(8)
Co(2)-C(44)	2.036(6)	C(9)-H(9)	0.9500
Co(2)-C(43)	2.046(5)	C(11)-C(12)	1.361(8)
Fe(1)-C(1)	2.011(7)	C(11)-H(11)	0.9500
Fe(1)-C(10)	2.014(6)	C(12)-C(13)	1.462(8)
Fe(1)-C(5)	2.019(7)	C(13)-C(14)	1.426(8)
Fe(1)-C(6)	2.031(6)	C(13)-C(17)	1.428(8)
Fe(1)-C(2)	2.032(7)	C(14)-C(15)	1.418(8)
Fe(1)-C(7)	2.033(7)	C(14)-H(14)	0.9500
Fe(1)-C(4)	2.044(8)	C(15)-C(16)	1.417(9)
Fe(1)-C(3)	2.046(7)	C(15)-H(15)	0.9500
Fe(1)-C(8)	2.049(7)	C(16)-C(17)	1.409(9)
Fe(1)-C(9)	2.051(6)	C(16)-H(16)	0.9500
Fe(2)-C(35)	2.009(7)	C(17)-H(17)	0.9500
Fe(2)-C(40)	2.025(6)	C(18)-C(22)	1.290(14)
Fe(2)-C(31)	2.028(6)	C(18)-C(19)	1.315(14)
Fe(2)-C(33)	2.035(7)	C(18)-H(18)	0.9500
Fe(2)-C(34)	2.037(7)	C(19)-C(20)	1.385(15)
Fe(2)-C(37)	2.039(6)	C(19)-H(19)	0.9500
Fe(2)-C(32)	2.041(7)	C(20)-C(21)	1.393(15)
Fe(2)-C(38)	2.044(6)	C(20)-H(20)	0.9500
Fe(2)-C(36)	2.046(6)	C(21)-C(22)	1.383(15)
Fe(2)-C(39)	2.047(6)	C(21)-H(21)	0.9500
N(1)-N(2)	1.342(7)	C(22)-H(22)	0.9500
N(1)-C(11)	1.345(7)	C(31)-C(32)	1.383(12)
N(1)-C(10)	1.433(7)	C(31)-C(35)	1.411(12)
N(2)-N(3)	1.320(7)	C(31)-H(31)	0.9500
N(3)-C(12)	1.348(7)	C(32)-C(33)	1.395(13)
N(4)-C(41)	1.336(7)	C(32)-H(32)	0.9500
N(4)-N(5)	1.347(6)	C(33)-C(34)	1.409(13)
N(4)-C(40)	1.417(7)	C(33)-H(33)	0.9500
N(5)-N(6)	1.312(7)	C(34)-C(35)	1.389(13)
N(6)-C(42)	1.361(7)	C(34)-H(34)	0.9500
C(1)-C(5)	1.411(13)	C(35)-H(35)	0.9500
C(1)-C(2)	1.432(12)	C(36)-C(40)	1.414(8)
C(1)-H(1)	0.9500	C(36)-C(37)	1.423(9)
C(2)-C(3)	1.377(12)	C(36)-H(36)	0.9500
C(2)-H(2)	0.9500	C(37)-C(38)	1.405(10)
C(3)-C(4)	1.359(12)	C(37)-H(37)	0.9500

C(38)-C(39)	1.427(8)	P(1)-F(6)	1.583(5)
C(38)-H(38)	0.9500	P(1)-F(5)	1.587(5)
C(39)-C(40)	1.427(9)	P(1)-F(1)	1.588(4)
C(39)-H(39)	0.9500	P(1)-F(3)	1.588(4)
C(41)-C(42)	1.370(7)	P(1)-F(2)	1.598(4)
C(41)-H(41)	0.9500	P(2)-F(9)	1.531(9)
C(42)-C(43)	1.454(8)	P(2)-F(7A)	1.54(2)
C(43)-C(44)	1.425(8)	P(2)-F(10A)	1.54(2)
C(43)-C(47)	1.427(8)	P(2)-F(9A)	1.556(18)
C(44)-C(45)	1.424(9)	P(2)-F(11)	1.556(5)
C(44)-H(44)	0.9500	P(2)-F(12)	1.564(5)
C(45)-C(46)	1.393(10)	P(2)-F(10)	1.566(11)
C(45)-H(45)	0.9500	P(2)-F(8)	1.570(8)
C(46)-C(47)	1.416(9)	P(2)-F(7)	1.573(8)
C(46)-H(46)	0.9500	P(2)-F(8A)	1.590(19)
C(47)-H(47)	0.9500	C(23)-Cl(1)	1.724(10)
C(48)-C(49)	1.349(14)	C(23)-Cl(2)	1.739(9)
C(48)-C(52)	1.442(12)	C(23)-H(23A)	0.9900
C(48)-H(48)	0.9500	C(23)-H(23B)	0.9900
C(49)-C(50)	1.349(14)	C(24)-Cl(4)	1.76(2)
C(49)-H(49)	0.9500	C(24)-Cl(3)	1.78(2)
C(50)-C(51)	1.357(11)	C(24)-H(24A)	0.9900
C(50)-H(50)	0.9500	C(24)-H(24B)	0.9900
C(51)-C(52)	1.389(11)	C(24A)-Cl(4A)	1.72(5)
C(51)-H(51)	0.9500	C(24A)-Cl(3A)	1.74(4)
C(52)-H(52)	0.9500	C(24A)-H(24C)	0.9900
P(1)-F(4)	1.573(5)	C(24A)-H(24D)	0.9900

C(22)-Co(1)-C(20)	67.0(4)	C(16)-Co(1)-C(14)	68.8(3)
C(22)-Co(1)-C(21)	40.5(4)	C(22)-Co(1)-C(17)	110.6(3)
C(20)-Co(1)-C(21)	40.8(5)	C(20)-Co(1)-C(17)	146.1(5)
C(22)-Co(1)-C(19)	64.8(4)	C(21)-Co(1)-C(17)	114.3(4)
C(20)-Co(1)-C(19)	40.5(5)	C(19)-Co(1)-C(17)	171.2(4)
C(21)-Co(1)-C(19)	67.5(4)	C(18)-Co(1)-C(17)	133.6(4)
C(22)-Co(1)-C(18)	37.6(4)	C(16)-Co(1)-C(17)	40.7(2)
C(20)-Co(1)-C(18)	66.2(4)	C(14)-Co(1)-C(17)	68.9(2)
C(21)-Co(1)-C(18)	66.2(4)	C(22)-Co(1)-C(15)	173.5(5)
C(19)-Co(1)-C(18)	38.3(4)	C(20)-Co(1)-C(15)	109.6(3)
C(22)-Co(1)-C(16)	134.6(4)	C(21)-Co(1)-C(15)	133.3(4)
C(20)-Co(1)-C(16)	115.4(3)	C(19)-Co(1)-C(15)	116.7(4)
C(21)-Co(1)-C(16)	109.3(3)	C(18)-Co(1)-C(15)	147.2(4)
C(19)-Co(1)-C(16)	147.8(4)	C(16)-Co(1)-C(15)	40.9(3)
C(18)-Co(1)-C(16)	171.8(4)	C(14)-Co(1)-C(15)	40.9(2)
C(22)-Co(1)-C(14)	145.4(4)	C(17)-Co(1)-C(15)	68.8(3)
C(20)-Co(1)-C(14)	133.2(4)	C(22)-Co(1)-C(13)	115.1(3)
C(21)-Co(1)-C(14)	173.0(5)	C(20)-Co(1)-C(13)	172.4(5)
C(19)-Co(1)-C(14)	110.3(3)	C(21)-Co(1)-C(13)	145.3(5)
C(18)-Co(1)-C(14)	116.6(4)	C(19)-Co(1)-C(13)	132.7(4)

C(18)-Co(1)-C(13)	110.5(3)	C(1)-Fe(1)-C(10)	117.9(3)
C(16)-Co(1)-C(13)	68.9(2)	C(1)-Fe(1)-C(5)	41.0(4)
C(14)-Co(1)-C(13)	41.0(2)	C(10)-Fe(1)-C(5)	109.7(3)
C(17)-Co(1)-C(13)	41.1(2)	C(1)-Fe(1)-C(6)	107.9(3)
C(15)-Co(1)-C(13)	69.1(2)	C(10)-Fe(1)-C(6)	40.7(2)
C(48)-Co(2)-C(52)	42.2(4)	C(5)-Fe(1)-C(6)	130.2(3)
C(48)-Co(2)-C(49)	39.3(4)	C(1)-Fe(1)-C(2)	41.5(4)
C(52)-Co(2)-C(49)	68.2(4)	C(10)-Fe(1)-C(2)	151.5(3)
C(48)-Co(2)-C(45)	171.8(4)	C(5)-Fe(1)-C(2)	68.4(4)
C(52)-Co(2)-C(45)	144.1(3)	C(6)-Fe(1)-C(2)	117.5(3)
C(49)-Co(2)-C(45)	133.1(4)	C(1)-Fe(1)-C(7)	129.1(4)
C(48)-Co(2)-C(50)	66.4(4)	C(10)-Fe(1)-C(7)	68.1(3)
C(52)-Co(2)-C(50)	67.4(3)	C(5)-Fe(1)-C(7)	168.6(4)
C(49)-Co(2)-C(50)	39.1(4)	C(6)-Fe(1)-C(7)	40.8(3)
C(45)-Co(2)-C(50)	109.4(4)	C(2)-Fe(1)-C(7)	107.9(3)
C(48)-Co(2)-C(51)	67.9(3)	C(1)-Fe(1)-C(4)	67.6(4)
C(52)-Co(2)-C(51)	40.4(3)	C(10)-Fe(1)-C(4)	130.9(3)
C(49)-Co(2)-C(51)	66.5(3)	C(5)-Fe(1)-C(4)	39.6(4)
C(45)-Co(2)-C(51)	113.8(3)	C(6)-Fe(1)-C(4)	168.5(3)
C(50)-Co(2)-C(51)	39.3(3)	C(2)-Fe(1)-C(4)	66.8(4)
C(48)-Co(2)-C(46)	147.5(4)	C(7)-Fe(1)-C(4)	150.2(3)
C(52)-Co(2)-C(46)	114.0(3)	C(1)-Fe(1)-C(3)	67.4(3)
C(49)-Co(2)-C(46)	171.4(4)	C(10)-Fe(1)-C(3)	167.8(3)
C(45)-Co(2)-C(46)	40.4(3)	C(5)-Fe(1)-C(3)	66.3(3)
C(50)-Co(2)-C(46)	133.1(4)	C(6)-Fe(1)-C(3)	150.7(3)
C(51)-Co(2)-C(46)	109.4(3)	C(2)-Fe(1)-C(3)	39.5(3)
C(48)-Co(2)-C(47)	116.5(4)	C(7)-Fe(1)-C(3)	118.3(3)
C(52)-Co(2)-C(47)	109.7(3)	C(4)-Fe(1)-C(3)	38.8(3)
C(49)-Co(2)-C(47)	147.2(4)	C(1)-Fe(1)-C(8)	167.4(4)
C(45)-Co(2)-C(47)	68.6(3)	C(10)-Fe(1)-C(8)	68.0(2)
C(50)-Co(2)-C(47)	172.6(4)	C(5)-Fe(1)-C(8)	150.3(4)
C(51)-Co(2)-C(47)	134.2(3)	C(6)-Fe(1)-C(8)	68.6(3)
C(46)-Co(2)-C(47)	41.0(3)	C(2)-Fe(1)-C(8)	128.3(3)
C(48)-Co(2)-C(44)	132.9(3)	C(7)-Fe(1)-C(8)	40.5(3)
C(52)-Co(2)-C(44)	174.3(3)	C(4)-Fe(1)-C(8)	118.1(3)
C(49)-Co(2)-C(44)	109.7(3)	C(3)-Fe(1)-C(8)	109.3(3)
C(45)-Co(2)-C(44)	41.2(2)	C(1)-Fe(1)-C(9)	150.5(4)
C(50)-Co(2)-C(44)	114.6(3)	C(10)-Fe(1)-C(9)	40.9(2)
C(51)-Co(2)-C(44)	144.4(3)	C(5)-Fe(1)-C(9)	117.2(3)
C(46)-Co(2)-C(44)	68.9(3)	C(6)-Fe(1)-C(9)	69.4(3)
C(47)-Co(2)-C(44)	69.0(2)	C(2)-Fe(1)-C(9)	166.4(3)
C(48)-Co(2)-C(43)	110.3(3)	C(7)-Fe(1)-C(9)	69.0(3)
C(52)-Co(2)-C(43)	134.6(3)	C(4)-Fe(1)-C(9)	108.9(3)
C(49)-Co(2)-C(43)	116.0(3)	C(3)-Fe(1)-C(9)	129.3(3)
C(45)-Co(2)-C(43)	68.8(2)	C(8)-Fe(1)-C(9)	41.0(3)
C(50)-Co(2)-C(43)	145.8(3)	C(35)-Fe(2)-C(40)	107.0(3)
C(51)-Co(2)-C(43)	174.2(3)	C(35)-Fe(2)-C(31)	40.9(3)
C(46)-Co(2)-C(43)	68.9(2)	C(40)-Fe(2)-C(31)	123.2(3)
C(47)-Co(2)-C(43)	41.0(2)	C(35)-Fe(2)-C(33)	67.5(4)
C(44)-Co(2)-C(43)	40.9(2)	C(40)-Fe(2)-C(33)	159.0(4)

C(31)-Fe(2)-C(33)	66.9(3)	C(5)-C(1)-C(2)	106.4(7)
C(35)-Fe(2)-C(34)	40.1(4)	C(5)-C(1)-Fe(1)	69.8(4)
C(40)-Fe(2)-C(34)	122.2(3)	C(2)-C(1)-Fe(1)	70.0(4)
C(31)-Fe(2)-C(34)	67.8(3)	C(5)-C(1)-H(1)	126.8
C(33)-Fe(2)-C(34)	40.5(4)	C(2)-C(1)-H(1)	126.8
C(35)-Fe(2)-C(37)	157.5(4)	Fe(1)-C(1)-H(1)	125.0
C(40)-Fe(2)-C(37)	67.9(2)	C(3)-C(2)-C(1)	106.5(8)
C(31)-Fe(2)-C(37)	122.2(3)	C(3)-C(2)-Fe(1)	70.8(4)
C(33)-Fe(2)-C(37)	125.0(3)	C(1)-C(2)-Fe(1)	68.5(4)
C(34)-Fe(2)-C(37)	161.1(4)	C(3)-C(2)-H(2)	126.7
C(35)-Fe(2)-C(32)	68.1(3)	C(1)-C(2)-H(2)	126.7
C(40)-Fe(2)-C(32)	158.8(4)	Fe(1)-C(2)-H(2)	125.5
C(31)-Fe(2)-C(32)	39.7(3)	C(4)-C(3)-C(2)	110.2(8)
C(33)-Fe(2)-C(32)	40.0(4)	C(4)-C(3)-Fe(1)	70.5(5)
C(34)-Fe(2)-C(32)	68.1(4)	C(2)-C(3)-Fe(1)	69.7(4)
C(37)-Fe(2)-C(32)	108.4(3)	C(4)-C(3)-H(3)	124.9
C(35)-Fe(2)-C(38)	160.3(4)	C(2)-C(3)-H(3)	124.9
C(40)-Fe(2)-C(38)	68.2(2)	Fe(1)-C(3)-H(3)	126.5
C(31)-Fe(2)-C(38)	157.8(3)	C(3)-C(4)-C(5)	108.7(9)
C(33)-Fe(2)-C(38)	109.7(3)	C(3)-C(4)-Fe(1)	70.7(5)
C(34)-Fe(2)-C(38)	124.9(3)	C(5)-C(4)-Fe(1)	69.2(5)
C(37)-Fe(2)-C(38)	40.2(3)	C(3)-C(4)-H(4)	125.6
C(32)-Fe(2)-C(38)	123.3(3)	C(5)-C(4)-H(4)	125.6
C(35)-Fe(2)-C(36)	121.0(3)	Fe(1)-C(4)-H(4)	126.1
C(40)-Fe(2)-C(36)	40.6(2)	C(4)-C(5)-C(1)	108.1(8)
C(31)-Fe(2)-C(36)	106.6(3)	C(4)-C(5)-Fe(1)	71.2(5)
C(33)-Fe(2)-C(36)	159.9(4)	C(1)-C(5)-Fe(1)	69.2(4)
C(34)-Fe(2)-C(36)	157.0(4)	C(4)-C(5)-H(5)	125.9
C(37)-Fe(2)-C(36)	40.8(2)	C(1)-C(5)-H(5)	125.9
C(32)-Fe(2)-C(36)	122.8(3)	Fe(1)-C(5)-H(5)	125.2
C(38)-Fe(2)-C(36)	68.6(3)	C(10)-C(6)-C(7)	106.6(6)
C(35)-Fe(2)-C(39)	123.0(3)	C(10)-C(6)-Fe(1)	69.0(3)
C(40)-Fe(2)-C(39)	41.0(2)	C(7)-C(6)-Fe(1)	69.7(4)
C(31)-Fe(2)-C(39)	159.8(3)	C(10)-C(6)-H(6)	126.7
C(33)-Fe(2)-C(39)	123.6(3)	C(7)-C(6)-H(6)	126.7
C(34)-Fe(2)-C(39)	107.8(3)	Fe(1)-C(6)-H(6)	126.2
C(37)-Fe(2)-C(39)	68.4(3)	C(8)-C(7)-C(6)	108.6(6)
C(32)-Fe(2)-C(39)	159.0(3)	C(8)-C(7)-Fe(1)	70.3(4)
C(38)-Fe(2)-C(39)	40.8(2)	C(6)-C(7)-Fe(1)	69.5(4)
C(36)-Fe(2)-C(39)	69.2(3)	C(8)-C(7)-H(7)	125.7
N(2)-N(1)-C(11)	111.1(5)	C(6)-C(7)-H(7)	125.7
N(2)-N(1)-C(10)	120.5(4)	Fe(1)-C(7)-H(7)	126.1
C(11)-N(1)-C(10)	128.4(5)	C(7)-C(8)-C(9)	108.7(6)
N(3)-N(2)-N(1)	106.4(4)	C(7)-C(8)-Fe(1)	69.2(4)
N(2)-N(3)-C(12)	109.3(5)	C(9)-C(8)-Fe(1)	69.6(3)
C(41)-N(4)-N(5)	110.9(4)	C(7)-C(8)-H(8)	125.7
C(41)-N(4)-C(40)	127.7(5)	C(9)-C(8)-H(8)	125.7
N(5)-N(4)-C(40)	121.4(5)	Fe(1)-C(8)-H(8)	127.1
N(6)-N(5)-N(4)	106.9(4)	C(10)-C(9)-C(8)	105.4(6)
N(5)-N(6)-C(42)	109.2(4)	C(10)-C(9)-Fe(1)	68.2(3)

C(8)-C(9)-Fe(1)	69.4(4)
C(10)-C(9)-H(9)	127.3
C(8)-C(9)-H(9)	127.3
Fe(1)-C(9)-H(9)	126.7
C(6)-C(10)-C(9)	110.7(6)
C(6)-C(10)-N(1)	124.8(5)
C(9)-C(10)-N(1)	124.5(6)
C(6)-C(10)-Fe(1)	70.3(3)
C(9)-C(10)-Fe(1)	71.0(3)
N(1)-C(10)-Fe(1)	125.6(4)
N(1)-C(11)-C(12)	104.8(5)
N(1)-C(11)-H(11)	127.6
C(12)-C(11)-H(11)	127.6
N(3)-C(12)-C(11)	108.5(5)
N(3)-C(12)-C(13)	121.2(5)
C(11)-C(12)-C(13)	130.2(5)
C(14)-C(13)-C(17)	106.9(5)
C(14)-C(13)-C(12)	127.1(5)
C(17)-C(13)-C(12)	126.0(5)
C(14)-C(13)-Co(1)	68.8(3)
C(17)-C(13)-Co(1)	68.8(3)
C(12)-C(13)-Co(1)	128.0(4)
C(15)-C(14)-C(13)	108.6(5)
C(15)-C(14)-Co(1)	69.7(3)
C(13)-C(14)-Co(1)	70.2(3)
C(15)-C(14)-H(14)	125.7
C(13)-C(14)-H(14)	125.7
Co(1)-C(14)-H(14)	126.0
C(16)-C(15)-C(14)	107.6(5)
C(16)-C(15)-Co(1)	69.3(4)
C(14)-C(15)-Co(1)	69.4(3)
C(16)-C(15)-H(15)	126.2
C(14)-C(15)-H(15)	126.2
Co(1)-C(15)-H(15)	126.7
C(17)-C(16)-C(15)	108.5(5)
C(17)-C(16)-Co(1)	69.8(3)
C(15)-C(16)-Co(1)	69.8(4)
C(17)-C(16)-H(16)	125.7
C(15)-C(16)-H(16)	125.7
Co(1)-C(16)-H(16)	126.3
C(16)-C(17)-C(13)	108.4(6)
C(16)-C(17)-Co(1)	69.5(3)
C(13)-C(17)-Co(1)	70.1(3)
C(16)-C(17)-H(17)	125.8
C(13)-C(17)-H(17)	125.8
Co(1)-C(17)-H(17)	126.1
C(22)-C(18)-C(19)	110.5(9)
C(22)-C(18)-Co(1)	70.6(5)
C(19)-C(18)-Co(1)	70.6(5)
C(22)-C(18)-H(18)	124.8
C(19)-C(18)-H(18)	124.8
Co(1)-C(18)-H(18)	125.7
C(18)-C(19)-C(20)	108.2(9)
C(18)-C(19)-Co(1)	71.2(5)
C(20)-C(19)-Co(1)	69.4(5)
C(18)-C(19)-H(19)	125.9
C(20)-C(19)-H(19)	125.9
Co(1)-C(19)-H(19)	125.1
C(19)-C(20)-C(21)	106.4(8)
C(19)-C(20)-Co(1)	70.0(5)
C(21)-C(20)-Co(1)	69.7(5)
C(19)-C(20)-H(20)	126.8
C(21)-C(20)-H(20)	126.8
Co(1)-C(20)-H(20)	125.0
C(22)-C(21)-C(20)	105.0(8)
C(22)-C(21)-Co(1)	69.6(5)
C(20)-C(21)-Co(1)	69.4(5)
C(22)-C(21)-H(21)	127.5
C(20)-C(21)-H(21)	127.5
Co(1)-C(21)-H(21)	125.1
C(18)-C(22)-C(21)	110.0(9)
C(18)-C(22)-Co(1)	71.8(5)
C(21)-C(22)-Co(1)	69.9(5)
C(18)-C(22)-H(22)	125.0
C(21)-C(22)-H(22)	125.0
Co(1)-C(22)-H(22)	124.8
C(32)-C(31)-C(35)	108.5(8)
C(32)-C(31)-Fe(2)	70.6(4)
C(35)-C(31)-Fe(2)	68.8(4)
C(32)-C(31)-H(31)	125.8
C(35)-C(31)-H(31)	125.8
Fe(2)-C(31)-H(31)	126.3
C(31)-C(32)-C(33)	107.4(8)
C(31)-C(32)-Fe(2)	69.6(4)
C(33)-C(32)-Fe(2)	69.8(5)
C(31)-C(32)-H(32)	126.3
C(33)-C(32)-H(32)	126.3
Fe(2)-C(32)-H(32)	125.9
C(32)-C(33)-C(34)	109.1(8)
C(32)-C(33)-Fe(2)	70.2(4)
C(34)-C(33)-Fe(2)	69.8(4)
C(32)-C(33)-H(33)	125.5
C(34)-C(33)-H(33)	125.5
Fe(2)-C(33)-H(33)	126.1
C(35)-C(34)-C(33)	106.9(8)
C(35)-C(34)-Fe(2)	68.9(4)
C(33)-C(34)-Fe(2)	69.7(5)
C(35)-C(34)-H(34)	126.6
C(33)-C(34)-H(34)	126.6
Fe(2)-C(34)-H(34)	126.4

C(34)-C(35)-C(31)	108.2(8)	C(43)-C(44)-Co(2)	70.0(3)
C(34)-C(35)-Fe(2)	71.0(4)	C(45)-C(44)-H(44)	126.4
C(31)-C(35)-Fe(2)	70.2(4)	C(43)-C(44)-H(44)	126.4
C(34)-C(35)-H(35)	125.9	Co(2)-C(44)-H(44)	126.7
C(31)-C(35)-H(35)	125.9	C(46)-C(45)-C(44)	109.0(5)
Fe(2)-C(35)-H(35)	124.5	C(46)-C(45)-Co(2)	70.1(3)
C(40)-C(36)-C(37)	106.2(6)	C(44)-C(45)-Co(2)	70.3(3)
C(40)-C(36)-Fe(2)	68.9(3)	C(46)-C(45)-H(45)	125.5
C(37)-C(36)-Fe(2)	69.4(3)	C(44)-C(45)-H(45)	125.5
C(40)-C(36)-H(36)	126.9	Co(2)-C(45)-H(45)	125.7
C(37)-C(36)-H(36)	126.9	C(45)-C(46)-C(47)	108.3(5)
Fe(2)-C(36)-H(36)	126.4	C(45)-C(46)-Co(2)	69.5(4)
C(38)-C(37)-C(36)	109.3(5)	C(47)-C(46)-Co(2)	69.8(3)
C(38)-C(37)-Fe(2)	70.1(3)	C(45)-C(46)-H(46)	125.9
C(36)-C(37)-Fe(2)	69.9(3)	C(47)-C(46)-H(46)	125.9
C(38)-C(37)-H(37)	125.3	Co(2)-C(46)-H(46)	126.4
C(36)-C(37)-H(37)	125.3	C(46)-C(47)-C(43)	108.0(6)
Fe(2)-C(37)-H(37)	126.3	C(46)-C(47)-Co(2)	69.2(3)
C(37)-C(38)-C(39)	108.4(5)	C(43)-C(47)-Co(2)	70.2(3)
C(37)-C(38)-Fe(2)	69.7(3)	C(46)-C(47)-H(47)	126.0
C(39)-C(38)-Fe(2)	69.7(3)	C(43)-C(47)-H(47)	126.0
C(37)-C(38)-H(38)	125.8	Co(2)-C(47)-H(47)	126.2
C(39)-C(38)-H(38)	125.8	C(49)-C(48)-C(52)	107.6(7)
Fe(2)-C(38)-H(38)	126.3	C(49)-C(48)-Co(2)	70.8(5)
C(38)-C(39)-C(40)	106.2(5)	C(52)-C(48)-Co(2)	69.2(4)
C(38)-C(39)-Fe(2)	69.5(3)	C(49)-C(48)-H(48)	126.2
C(40)-C(39)-Fe(2)	68.7(3)	C(52)-C(48)-H(48)	126.2
C(38)-C(39)-H(39)	126.9	Co(2)-C(48)-H(48)	125.4
C(40)-C(39)-H(39)	126.9	C(50)-C(49)-C(48)	109.2(8)
Fe(2)-C(39)-H(39)	126.5	C(50)-C(49)-Co(2)	70.6(5)
C(36)-C(40)-N(4)	124.7(6)	C(48)-C(49)-Co(2)	69.9(5)
C(36)-C(40)-C(39)	109.8(5)	C(50)-C(49)-H(49)	125.4
N(4)-C(40)-C(39)	125.5(5)	C(48)-C(49)-H(49)	125.4
C(36)-C(40)-Fe(2)	70.5(3)	Co(2)-C(49)-H(49)	125.6
N(4)-C(40)-Fe(2)	126.4(4)	C(49)-C(50)-C(51)	109.4(9)
C(39)-C(40)-Fe(2)	70.3(3)	C(49)-C(50)-Co(2)	70.2(5)
N(4)-C(41)-C(42)	105.4(4)	C(51)-C(50)-Co(2)	70.3(4)
N(4)-C(41)-H(41)	127.3	C(49)-C(50)-H(50)	125.3
C(42)-C(41)-H(41)	127.3	C(51)-C(50)-H(50)	125.3
N(6)-C(42)-C(41)	107.6(5)	Co(2)-C(50)-H(50)	125.7
N(6)-C(42)-C(43)	122.4(5)	C(50)-C(51)-C(52)	108.8(7)
C(41)-C(42)-C(43)	129.9(5)	C(50)-C(51)-Co(2)	70.3(4)
C(44)-C(43)-C(47)	107.5(5)	C(52)-C(51)-Co(2)	69.5(4)
C(44)-C(43)-C(42)	126.8(5)	C(50)-C(51)-H(51)	125.6
C(47)-C(43)-C(42)	125.7(5)	C(52)-C(51)-H(51)	125.6
C(44)-C(43)-Co(2)	69.2(3)	Co(2)-C(51)-H(51)	126.1
C(47)-C(43)-Co(2)	68.8(3)	C(51)-C(52)-C(48)	104.9(7)
C(42)-C(43)-Co(2)	128.2(4)	C(51)-C(52)-Co(2)	70.1(4)
C(45)-C(44)-C(43)	107.2(6)	C(48)-C(52)-Co(2)	68.6(4)
C(45)-C(44)-Co(2)	68.5(3)	C(51)-C(52)-H(52)	127.5

C(48)-C(52)-H(52)	127.5
Co(2)-C(52)-H(52)	125.3
F(4)-P(1)-F(6)	91.5(4)
F(4)-P(1)-F(5)	89.5(4)
F(6)-P(1)-F(5)	178.9(3)
F(4)-P(1)-F(1)	90.4(3)
F(6)-P(1)-F(1)	89.9(3)
F(5)-P(1)-F(1)	89.7(3)
F(4)-P(1)-F(3)	179.1(4)
F(6)-P(1)-F(3)	89.4(3)
F(5)-P(1)-F(3)	89.5(3)
F(1)-P(1)-F(3)	89.4(2)
F(4)-P(1)-F(2)	90.1(3)
F(6)-P(1)-F(2)	89.9(3)
F(5)-P(1)-F(2)	90.5(3)
F(1)-P(1)-F(2)	179.4(2)
F(3)-P(1)-F(2)	90.1(2)
F(7A)-P(2)-F(10A)	89(2)
F(7A)-P(2)-F(9A)	90.4(19)
F(10A)-P(2)-F(9A)	179(2)
F(9)-P(2)-F(11)	100.1(6)
F(7A)-P(2)-F(11)	96.2(11)
F(10A)-P(2)-F(11)	100.5(14)
F(9A)-P(2)-F(11)	79.4(9)
F(9)-P(2)-F(12)	81.4(6)
F(7A)-P(2)-F(12)	83.1(11)
F(10A)-P(2)-F(12)	77.8(14)
F(9A)-P(2)-F(12)	102.3(9)
F(11)-P(2)-F(12)	178.2(5)
F(9)-P(2)-F(10)	178.1(8)
F(11)-P(2)-F(10)	81.8(6)
F(12)-P(2)-F(10)	96.7(7)
F(9)-P(2)-F(8)	89.5(6)
F(11)-P(2)-F(8)	87.9(4)
F(12)-P(2)-F(8)	91.1(4)
F(10)-P(2)-F(8)	90.6(7)
F(9)-P(2)-F(7)	90.8(6)
F(11)-P(2)-F(7)	92.5(4)
F(12)-P(2)-F(7)	88.5(4)
F(10)-P(2)-F(7)	89.1(7)
F(8)-P(2)-F(7)	179.4(7)
F(7A)-P(2)-F(8A)	176.9(14)
F(10A)-P(2)-F(8A)	92(2)
F(9A)-P(2)-F(8A)	88.2(19)
F(11)-P(2)-F(8A)	80.8(8)
F(12)-P(2)-F(8A)	99.9(9)
Cl(1)-C(23)-Cl(2)	111.2(5)
Cl(1)-C(23)-H(23A)	109.4
Cl(2)-C(23)-H(23A)	109.4
Cl(1)-C(23)-H(23B)	109.4
Cl(2)-C(23)-H(23B)	109.4
H(23A)-C(23)-H(23B)	108.0
Cl(4)-C(24)-Cl(3)	108.3(10)
Cl(4)-C(24)-H(24A)	110.0
Cl(3)-C(24)-H(24A)	110.0
Cl(4)-C(24)-H(24B)	110.0
Cl(3)-C(24)-H(24B)	110.0
H(24A)-C(24)-H(24B)	108.4
Cl(4A)-C(24A)-Cl(3A)	117(2)
Cl(4A)-C(24A)-H(24C)	108.1
Cl(3A)-C(24A)-H(24C)	108.1
Cl(4A)-C(24A)-H(24D)	108.1
Cl(3A)-C(24A)-H(24D)	108.1
H(24C)-C(24A)-H(24D)	107.3

Table S4. Anisotropic displacement parameters (Å^{2}x 10^3) for **5**. The anisotropic displacement factor exponent takes the form: $-2p^2 [h^2 a^{*2}U^{11} + ... + 2 h k a^* b^* U^{12}]$.

	U^{11}	U^{22}	U^{33}	U^{23}	U^{13}	U^{12}
Co(1)	16(1)	21(1)	19(1)	1(1)	5(1)	0(1)
Co(2)	20(1)	20(1)	16(1)	1(1)	3(1)	-4(1)
Fe(1)	22(1)	34(1)	15(1)	1(1)	4(1)	-8(1)
Fe(2)	24(1)	25(1)	18(1)	5(1)	2(1)	-5(1)
N(1)	20(2)	18(2)	23(2)	3(2)	10(2)	6(2)

N(2)	33(3)	27(3)	61(4)	10(2)	26(3)	9(2)
N(3)	32(3)	26(3)	55(3)	9(2)	25(3)	12(2)
N(4)	18(2)	21(2)	22(2)	-5(2)	4(2)	2(2)
N(5)	15(2)	29(3)	55(3)	-16(2)	6(2)	3(2)
N(6)	20(3)	29(3)	51(3)	-14(2)	2(2)	6(2)
C(1)	114(9)	43(4)	31(4)	-9(3)	-1(5)	-44(5)
C(2)	53(5)	67(5)	38(4)	-26(4)	13(4)	-4(4)
C(3)	62(5)	64(5)	19(3)	-8(3)	13(3)	-20(4)
C(4)	56(6)	87(6)	24(4)	2(4)	-5(4)	1(5)
C(5)	38(5)	110(8)	31(4)	-23(5)	1(3)	-37(5)
C(6)	19(3)	46(4)	22(3)	7(3)	5(2)	5(3)
C(7)	20(3)	64(5)	25(3)	0(3)	6(3)	-6(3)
C(8)	35(4)	51(4)	27(3)	-3(3)	8(3)	-23(3)
C(9)	40(4)	29(3)	21(3)	-1(2)	5(3)	-8(3)
C(10)	26(3)	32(3)	17(3)	4(2)	6(2)	-2(2)
C(11)	24(3)	21(3)	23(3)	3(2)	7(2)	7(2)
C(12)	20(3)	24(3)	19(3)	2(2)	7(2)	6(2)
C(13)	15(3)	30(3)	20(3)	3(2)	5(2)	4(2)
C(14)	31(3)	33(3)	16(3)	-6(2)	7(2)	-2(3)
C(15)	31(3)	33(3)	23(3)	2(3)	4(3)	-13(3)
C(16)	18(3)	60(4)	24(3)	11(3)	5(2)	-1(3)
C(17)	18(3)	39(4)	30(3)	15(3)	8(3)	14(3)
C(18)	33(5)	127(10)	41(5)	16(6)	-7(4)	-28(5)
C(19)	65(6)	86(7)	47(5)	-14(5)	-21(4)	54(6)
C(20)	88(8)	73(6)	51(5)	50(5)	-48(5)	-64(6)
C(21)	44(5)	190(13)	10(4)	-7(5)	6(3)	40(7)
C(22)	118(10)	36(5)	37(5)	-3(4)	-33(6)	-5(5)
C(31)	46(4)	80(6)	36(4)	32(4)	20(4)	2(4)
C(32)	95(8)	59(5)	16(3)	6(3)	-3(4)	-2(5)
C(33)	36(5)	100(7)	43(5)	38(5)	-16(4)	-18(5)
C(34)	55(5)	79(6)	54(5)	40(5)	19(4)	34(5)
C(35)	78(6)	40(4)	45(5)	23(4)	-3(4)	-9(4)
C(36)	28(3)	25(3)	25(3)	-3(2)	0(3)	6(3)
C(37)	50(4)	19(3)	30(3)	1(2)	2(3)	-1(3)
C(38)	39(4)	29(3)	26(3)	7(2)	2(3)	-19(3)
C(39)	27(3)	31(3)	21(3)	6(2)	7(3)	-8(3)
C(40)	27(3)	22(3)	15(3)	2(2)	-2(2)	1(2)
C(41)	17(3)	20(3)	22(3)	2(2)	7(2)	5(2)
C(42)	17(3)	22(3)	17(3)	0(2)	2(2)	6(2)
C(43)	25(3)	26(3)	12(3)	-2(2)	1(2)	-3(3)
C(44)	23(3)	39(4)	21(3)	-6(3)	7(3)	-5(3)
C(45)	26(3)	58(4)	22(3)	-2(3)	11(3)	-16(3)
C(46)	46(4)	35(4)	23(3)	5(3)	5(3)	-15(3)
C(47)	33(3)	18(3)	20(3)	2(2)	-2(3)	4(3)
C(48)	53(5)	135(9)	22(4)	19(5)	4(4)	-62(6)
C(49)	130(10)	47(5)	26(4)	16(4)	3(5)	-15(6)
C(50)	72(6)	80(6)	26(4)	10(4)	-5(4)	41(5)
C(51)	48(5)	60(5)	15(3)	3(3)	1(3)	-33(4)
C(52)	81(6)	68(5)	20(3)	9(3)	25(4)	33(5)
P(1)	35(1)	29(1)	38(1)	-8(1)	20(1)	-11(1)

F(1)	41(2)	48(2)	74(3)	-4(2)	32(2)	-3(2)
F(2)	48(3)	59(3)	90(3)	-25(3)	44(3)	-22(2)
F(3)	57(3)	63(3)	67(3)	-37(2)	31(3)	-22(2)
F(4)	71(4)	83(4)	148(5)	-87(4)	48(4)	-30(3)
F(5)	88(4)	83(4)	111(5)	61(3)	37(4)	-5(3)
F(6)	62(3)	96(4)	62(3)	33(3)	20(3)	18(3)
P(2)	25(1)	24(1)	40(1)	-5(1)	9(1)	-3(1)
F(11)	51(3)	32(2)	226(8)	18(3)	40(4)	-2(2)
F(12)	91(5)	27(2)	271(9)	14(4)	76(5)	6(3)
C(23)	85(7)	68(6)	87(7)	-15(5)	42(6)	-26(5)
Cl(1)	93(2)	178(3)	84(2)	5(2)	57(2)	-13(2)
Cl(2)	92(2)	94(2)	63(1)	-13(1)	40(1)	-43(2)
F(7)	37(4)	60(6)	161(12)	-28(7)	53(6)	-12(4)
F(8)	44(4)	46(4)	118(9)	5(5)	52(5)	1(3)
F(9)	83(7)	180(11)	44(4)	-28(7)	14(5)	-40(9)
F(10)	113(12)	155(13)	53(5)	-28(6)	-12(7)	4(9)
C(24)	120(20)	140(20)	26(7)	12(11)	20(10)	-4(14)
Cl(3)	77(3)	91(3)	56(2)	2(2)	-13(2)	18(2)
Cl(4)	68(3)	74(3)	48(3)	7(2)	7(2)	24(3)
F(7A)	63(17)	94(18)	110(30)	24(17)	-59(16)	-4(13)
F(8A)	48(13)	79(14)	140(30)	-26(15)	-52(14)	11(11)
F(9A)	220(40)	59(13)	95(17)	-7(13)	140(20)	-3(19)
F(10A)	230(40)	140(30)	160(30)	-40(20)	170(30)	-90(30)
C(24A)	100(30)	60(20)	50(20)	-11(17)	20(20)	-1(19)
Cl(3A)	123(9)	86(7)	63(6)	-20(5)	-29(6)	63(7)
Cl(4A)	82(11)	148(19)	62(10)	50(11)	52(9)	43(12)

Table S5. Hydrogen coordinates (x 10^4) and isotropic displacement parameters ($Å^2$x 10^3) for **5**.

	x	y	z	U(eq)
H(1)	348	1792	4905	81
H(2)	-979	1156	5271	63
H(3)	-515	-405	5617	58
H(4)	1010	-774	5474	71
H(5)	1572	554	5042	74
H(6)	-1047	1125	3671	35
H(7)	-2103	130	4067	44
H(8)	-1335	-1394	4304	46
H(9)	239	-1357	4072	37
H(11)	401	1660	3406	27
H(14)	1672	3028	3342	32

H(15)	2884	3776	2945	36
H(16)	3745	2542	2583	41
H(17)	3081	1038	2756	34
H(18)	48	1940	2039	85
H(19)	414	3521	1988	88
H(20)	1734	3651	1543	102
H(21)	2155	2048	1336	98
H(22)	1059	1042	1666	90
H(31)	4836	9531	5146	63
H(32)	6087	10704	5466	73
H(33)	7470	10206	5149	79
H(34)	7067	8730	4614	74
H(35)	5419	8332	4596	70
H(36)	3931	10565	3931	33
H(37)	5169	11793	4241	42
H(38)	6569	11365	3921	39
H(39)	6227	9855	3396	31
H(41)	3030	9344	3230	23
H(44)	1443	8348	2879	33
H(45)	404	6973	2557	41
H(46)	1399	5688	2407	43
H(47)	3073	6241	2618	31
H(48)	2887	8005	1489	86
H(49)	1614	9001	1578	86
H(50)	146	8144	1340	76
H(51)	446	6586	1089	51
H(52)	2170	6430	1183	65
H(23A)	2185	8911	4654	92
H(23B)	3068	8704	5197	92
H(24A)	-1853	2562	489	116
H(24B)	-1443	3572	565	116
H(24C)	-1313	3764	591	81
H(24D)	-553	3634	251	81

Table S6. Torsion angles [°] for **5**.

C(11)-N(1)-N(2)-N(3)	-0.1(7)	C(10)-N(1)-C(11)-C(12)	179.2(5)
C(10)-N(1)-N(2)-N(3)	-178.6(5)	N(2)-N(3)-C(12)-C(11)	1.3(7)
N(1)-N(2)-N(3)-C(12)	-0.7(7)	N(2)-N(3)-C(12)-C(13)	177.5(5)
C(41)-N(4)-N(5)-N(6)	-0.3(6)	N(1)-C(11)-C(12)-N(3)	-1.3(6)
C(40)-N(4)-N(5)-N(6)	177.1(5)	N(1)-C(11)-C(12)-C(13)	-177.0(5)
N(4)-N(5)-N(6)-C(42)	0.1(7)	N(3)-C(12)-C(13)-C(14)	-156.0(6)
C(5)-C(1)-C(2)-C(3)	0.3(8)	C(11)-C(12)-C(13)-C(14)	19.2(10)
Fe(1)-C(1)-C(2)-C(3)	61.0(5)	N(3)-C(12)-C(13)-C(17)	23.1(9)
C(5)-C(1)-C(2)-Fe(1)	-60.7(5)	C(11)-C(12)-C(13)-C(17)	-161.7(6)
C(1)-C(2)-C(3)-C(4)	-0.4(9)	N(3)-C(12)-C(13)-Co(1)	113.1(6)
Fe(1)-C(2)-C(3)-C(4)	59.0(6)	C(11)-C(12)-C(13)-Co(1)	-71.6(8)
C(1)-C(2)-C(3)-Fe(1)	-59.4(5)	C(17)-C(13)-C(14)-C(15)	-0.8(6)
C(2)-C(3)-C(4)-C(5)	0.4(9)	C(12)-C(13)-C(14)-C(15)	178.4(5)
Fe(1)-C(3)-C(4)-C(5)	58.9(5)	Co(1)-C(13)-C(14)-C(15)	-59.3(4)
C(2)-C(3)-C(4)-Fe(1)	-58.5(6)	C(17)-C(13)-C(14)-Co(1)	58.5(4)
C(3)-C(4)-C(5)-C(1)	-0.2(9)	C(12)-C(13)-C(14)-Co(1)	-122.3(6)
Fe(1)-C(4)-C(5)-C(1)	59.6(5)	C(13)-C(14)-C(15)-C(16)	0.6(7)
C(3)-C(4)-C(5)-Fe(1)	-59.8(6)	Co(1)-C(14)-C(15)-C(16)	-59.0(4)
C(2)-C(1)-C(5)-C(4)	-0.1(8)	C(13)-C(14)-C(15)-Co(1)	59.6(4)
Fe(1)-C(1)-C(5)-C(4)	-60.9(5)	C(14)-C(15)-C(16)-C(17)	-0.2(7)
C(2)-C(1)-C(5)-Fe(1)	60.8(5)	Co(1)-C(15)-C(16)-C(17)	-59.2(4)
C(10)-C(6)-C(7)-C(8)	-0.3(7)	C(14)-C(15)-C(16)-Co(1)	59.0(4)
Fe(1)-C(6)-C(7)-C(8)	-59.7(5)	C(15)-C(16)-C(17)-C(13)	-0.4(7)
C(10)-C(6)-C(7)-Fe(1)	59.4(4)	Co(1)-C(16)-C(17)-C(13)	-59.5(4)
C(6)-C(7)-C(8)-C(9)	0.6(7)	C(15)-C(16)-C(17)-Co(1)	59.2(4)
Fe(1)-C(7)-C(8)-C(9)	-58.5(4)	C(14)-C(13)-C(17)-C(16)	0.7(6)
C(6)-C(7)-C(8)-Fe(1)	59.1(4)	C(12)-C(13)-C(17)-C(16)	-178.5(5)
C(7)-C(8)-C(9)-C(10)	-0.7(7)	Co(1)-C(13)-C(17)-C(16)	59.2(4)
Fe(1)-C(8)-C(9)-C(10)	-59.0(4)	C(14)-C(13)-C(17)-Co(1)	-58.5(4)
C(7)-C(8)-C(9)-Fe(1)	58.3(5)	C(12)-C(13)-C(17)-Co(1)	122.3(6)
C(7)-C(6)-C(10)-C(9)	-0.2(7)	C(22)-C(18)-C(19)-C(20)	0.4(10)
Fe(1)-C(6)-C(10)-C(9)	59.6(4)	Co(1)-C(18)-C(19)-C(20)	59.8(6)
C(7)-C(6)-C(10)-N(1)	179.9(5)	C(22)-C(18)-C(19)-Co(1)	-59.4(7)
Fe(1)-C(6)-C(10)-N(1)	-120.3(6)	C(18)-C(19)-C(20)-C(21)	-0.3(10)
C(7)-C(6)-C(10)-Fe(1)	-59.8(4)	Co(1)-C(19)-C(20)-C(21)	60.6(6)
C(8)-C(9)-C(10)-C(6)	0.6(7)	C(18)-C(19)-C(20)-Co(1)	-60.9(6)
Fe(1)-C(9)-C(10)-C(6)	-59.2(4)	C(19)-C(20)-C(21)-C(22)	0.1(9)
C(8)-C(9)-C(10)-N(1)	-179.6(5)	Co(1)-C(20)-C(21)-C(22)	60.9(6)
Fe(1)-C(9)-C(10)-N(1)	120.7(6)	C(19)-C(20)-C(21)-Co(1)	-60.8(6)
C(8)-C(9)-C(10)-Fe(1)	59.8(4)	C(19)-C(18)-C(22)-C(21)	-0.3(10)
N(2)-N(1)-C(10)-C(6)	-176.7(6)	Co(1)-C(18)-C(22)-C(21)	-59.8(6)
C(11)-N(1)-C(10)-C(6)	5.1(9)	C(19)-C(18)-C(22)-Co(1)	59.4(6)
N(2)-N(1)-C(10)-C(9)	3.4(9)	C(20)-C(21)-C(22)-C(18)	0.1(10)
C(11)-N(1)-C(10)-C(9)	-174.8(6)	Co(1)-C(21)-C(22)-C(18)	60.9(6)
N(2)-N(1)-C(10)-Fe(1)	93.7(6)	C(20)-C(21)-C(22)-Co(1)	-60.8(6)
C(11)-N(1)-C(10)-Fe(1)	-84.5(7)	C(35)-C(31)-C(32)-C(33)	-1.2(8)
N(2)-N(1)-C(11)-C(12)	0.9(6)	Fe(2)-C(31)-C(32)-C(33)	-59.8(5)

C(35)-C(31)-C(32)-Fe(2)	58.6(5)	N(4)-C(41)-C(42)-N(6)	-0.2(6)
C(31)-C(32)-C(33)-C(34)	0.6(8)	N(4)-C(41)-C(42)-C(43)	175.9(5)
Fe(2)-C(32)-C(33)-C(34)	-59.2(5)	N(6)-C(42)-C(43)-C(44)	179.0(6)
C(31)-C(32)-C(33)-Fe(2)	59.7(5)	C(41)-C(42)-C(43)-C(44)	3.4(9)
C(32)-C(33)-C(34)-C(35)	0.3(8)	N(6)-C(42)-C(43)-C(47)	0.2(9)
Fe(2)-C(33)-C(34)-C(35)	-59.1(5)	C(41)-C(42)-C(43)-C(47)	-175.4(6)
C(32)-C(33)-C(34)-Fe(2)	59.4(5)	N(6)-C(42)-C(43)-Co(2)	-89.7(7)
C(33)-C(34)-C(35)-C(31)	-1.1(8)	C(41)-C(42)-C(43)-Co(2)	94.7(7)
Fe(2)-C(34)-C(35)-C(31)	-60.7(5)	C(47)-C(43)-C(44)-C(45)	0.3(6)
C(33)-C(34)-C(35)-Fe(2)	59.6(5)	C(42)-C(43)-C(44)-C(45)	-178.7(5)
C(32)-C(31)-C(35)-C(34)	1.4(8)	Co(2)-C(43)-C(44)-C(45)	58.6(4)
Fe(2)-C(31)-C(35)-C(34)	61.2(5)	C(47)-C(43)-C(44)-Co(2)	-58.3(4)
C(32)-C(31)-C(35)-Fe(2)	-59.7(5)	C(42)-C(43)-C(44)-Co(2)	122.8(6)
C(40)-C(36)-C(37)-C(38)	-0.2(7)	C(43)-C(44)-C(45)-C(46)	0.1(7)
Fe(2)-C(36)-C(37)-C(38)	59.1(4)	Co(2)-C(44)-C(45)-C(46)	59.6(4)
C(40)-C(36)-C(37)-Fe(2)	-59.3(4)	C(43)-C(44)-C(45)-Co(2)	-59.5(4)
C(36)-C(37)-C(38)-C(39)	0.2(7)	C(44)-C(45)-C(46)-C(47)	-0.5(7)
Fe(2)-C(37)-C(38)-C(39)	59.2(4)	Co(2)-C(45)-C(46)-C(47)	59.3(4)
C(36)-C(37)-C(38)-Fe(2)	-59.0(4)	C(44)-C(45)-C(46)-Co(2)	-59.7(4)
C(37)-C(38)-C(39)-C(40)	-0.1(7)	C(45)-C(46)-C(47)-C(43)	0.7(6)
Fe(2)-C(38)-C(39)-C(40)	59.1(4)	Co(2)-C(46)-C(47)-C(43)	59.7(4)
C(37)-C(38)-C(39)-Fe(2)	-59.2(4)	C(45)-C(46)-C(47)-Co(2)	-59.1(4)
C(37)-C(36)-C(40)-N(4)	-179.0(5)	C(44)-C(43)-C(47)-C(46)	-0.6(6)
Fe(2)-C(36)-C(40)-N(4)	121.4(5)	C(42)-C(43)-C(47)-C(46)	178.4(5)
C(37)-C(36)-C(40)-C(39)	0.1(7)	Co(2)-C(43)-C(47)-C(46)	-59.1(4)
Fe(2)-C(36)-C(40)-C(39)	-59.5(4)	C(44)-C(43)-C(47)-Co(2)	58.5(4)
C(37)-C(36)-C(40)-Fe(2)	59.6(4)	C(42)-C(43)-C(47)-Co(2)	-122.5(5)
C(41)-N(4)-C(40)-C(36)	17.2(9)	C(52)-C(48)-C(49)-C(50)	-0.2(9)
N(5)-N(4)-C(40)-C(36)	-159.7(6)	Co(2)-C(48)-C(49)-C(50)	-59.9(6)
C(41)-N(4)-C(40)-C(39)	-161.8(6)	C(52)-C(48)-C(49)-Co(2)	59.7(5)
N(5)-N(4)-C(40)-C(39)	21.3(8)	C(48)-C(49)-C(50)-C(51)	-0.2(9)
C(41)-N(4)-C(40)-Fe(2)	107.5(6)	Co(2)-C(49)-C(50)-C(51)	-59.6(5)
N(5)-N(4)-C(40)-Fe(2)	-69.4(7)	C(48)-C(49)-C(50)-Co(2)	59.5(6)
C(38)-C(39)-C(40)-C(36)	0.0(7)	C(49)-C(50)-C(51)-C(52)	0.5(8)
Fe(2)-C(39)-C(40)-C(36)	59.6(4)	Co(2)-C(50)-C(51)-C(52)	-59.1(5)
C(38)-C(39)-C(40)-N(4)	179.1(5)	C(49)-C(50)-C(51)-Co(2)	59.5(5)
Fe(2)-C(39)-C(40)-N(4)	-121.3(6)	C(50)-C(51)-C(52)-C(48)	-0.6(8)
C(38)-C(39)-C(40)-Fe(2)	-59.6(4)	Co(2)-C(51)-C(52)-C(48)	-60.2(5)
N(5)-N(4)-C(41)-C(42)	0.3(6)	C(50)-C(51)-C(52)-Co(2)	59.6(5)
C(40)-N(4)-C(41)-C(42)	-176.9(5)	C(49)-C(48)-C(52)-C(51)	0.5(8)
N(5)-N(6)-C(42)-C(41)	0.0(7)	Co(2)-C(48)-C(52)-C(51)	61.2(5)
N(5)-N(6)-C(42)-C(43)	-176.4(5)	C(49)-C(48)-C(52)-Co(2)	-60.7(5)

Triazole (6) – "1-cobaltoceniumyl-4-ferrocenyltriazole hexafluoridophosphate"

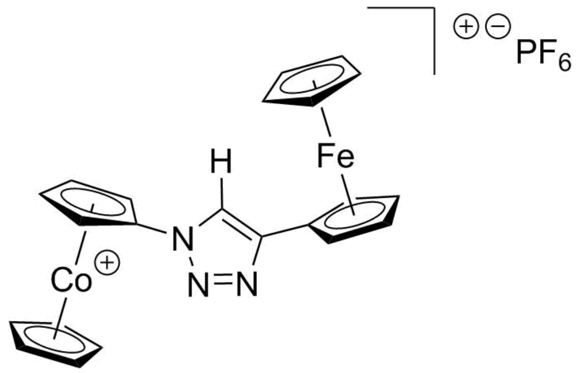

[585.15]

$C_{22}H_{19}N_3CoFe(PF_6)$

Analytical data:

IR (ATR [cm^{-1}]): 3133 (ν_{C-H}), 1593, 1539, 1419 ($\nu_{C=C}$), 1259, 1125, 1028, 816 (ν_{P-F}), 557 (ν_{P-F}), 490,446.

^{1}H-NMR (300 MHz, C_3D_6O, [ppm]): δ 4.09 (s, 5H, Fc-Cp), 4.39 (pseudo-t, 2H, J = 1.8 Hz, C3/C4 of substituted Fc-Cp), 4.83 (pseudo-t, 2H, J = 1.8 Hz, C2/C5 of substituted Fc-Cp), 5.97 (s, 5H, Cc-Cp), 6.09 (pseudo-t, 2H, J = 2.3 Hz, C3/C4 of substituted Cc-Cp), 6.70 (pseudo-t, 2H, J = 2.3 Hz, C2/C5 of substituted Cc-Cp), 8.71 (s, 1H, CH of triazole).

^{13}C-NMR (75 MHz, C_3D_6O, [ppm]): δ 67.7 (C3/C4 of substituted Fc-Cp), 69.9 (C2/C5 of substituted Fc-Cp), 70.4 (Fc-Cp), 75.2 (quart. carbon of substituted Fc-Cp), 76,1 (C3/C4 of substituted Cc-Cp), 83.3 (C2/C5 of substituted Cc-Cp), 87.3 (Cc-Cp), 108.6 (quart. carbon of substituted Cc-Cp), 120.0 (CH of triazole), 149.4 (quart. carbon of triazole).

MS (ESI pos, [m/z]): 440.03 (M^+ - PF_6^-).

Melting point [°C]: 173.

Spectra

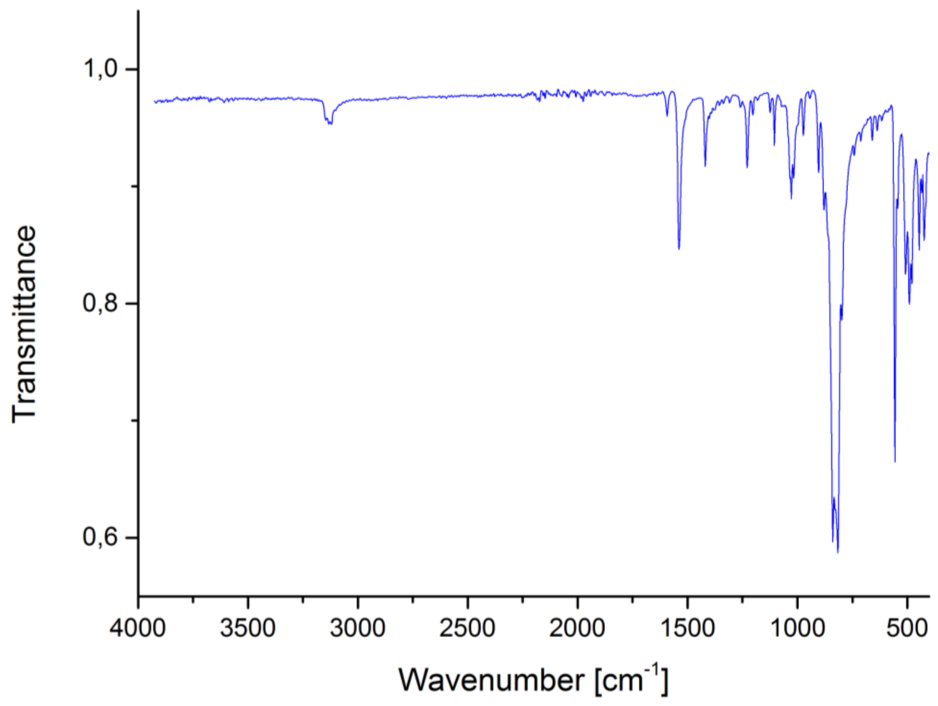

Figure S6. IR-spectrum (ATR, [cm^{-1}]) of “1-cobaltoceniumyl-4-ferrocenyltriazole hexafluoridophosphate“.

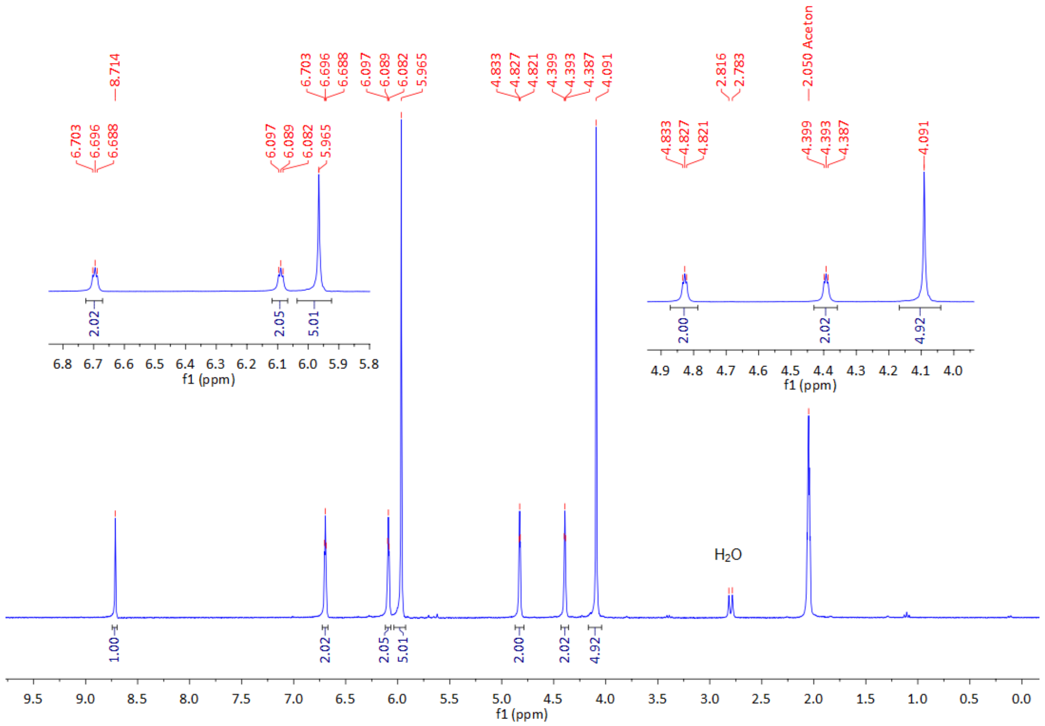

Figure S7. ^{1}H-NMR (300 MHz, C_3D_6O, [ppm]) of "1-cobaltoceniumyl-4-ferrocenyltriazole hexafluoridophosphate".

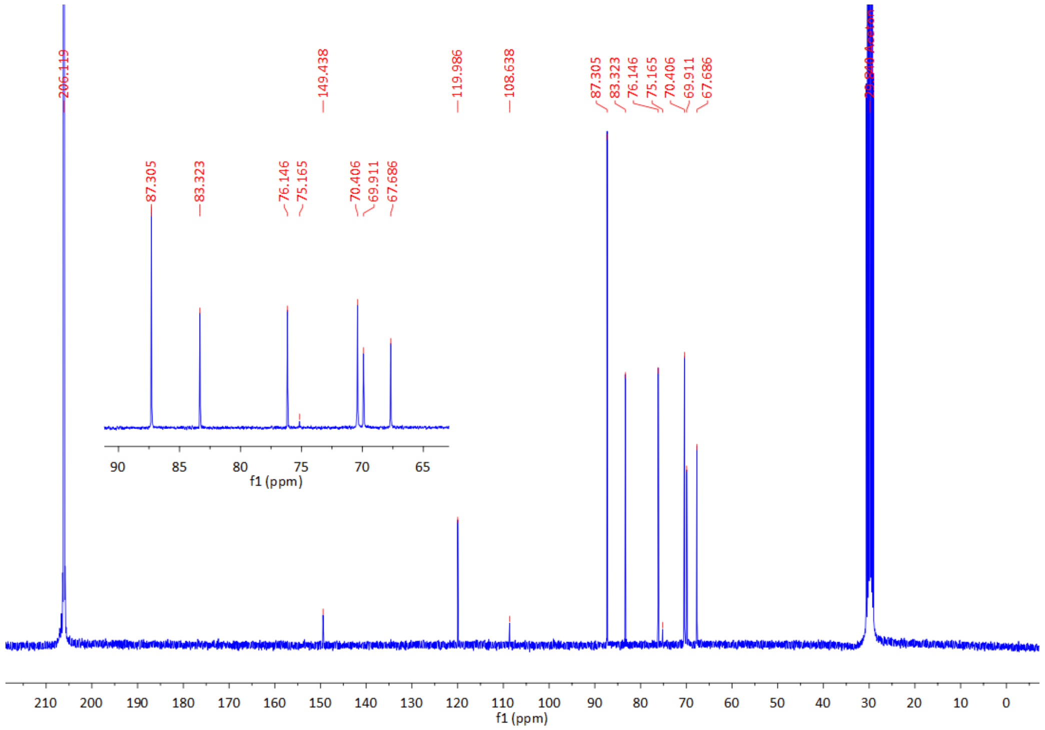

Figure S8. ^{13}C-NMR (75 MHz, C_3D_6O, [ppm]) of "1-cobaltoceniumyl-4-ferrocenyltriazole hexafluoridophosphate".

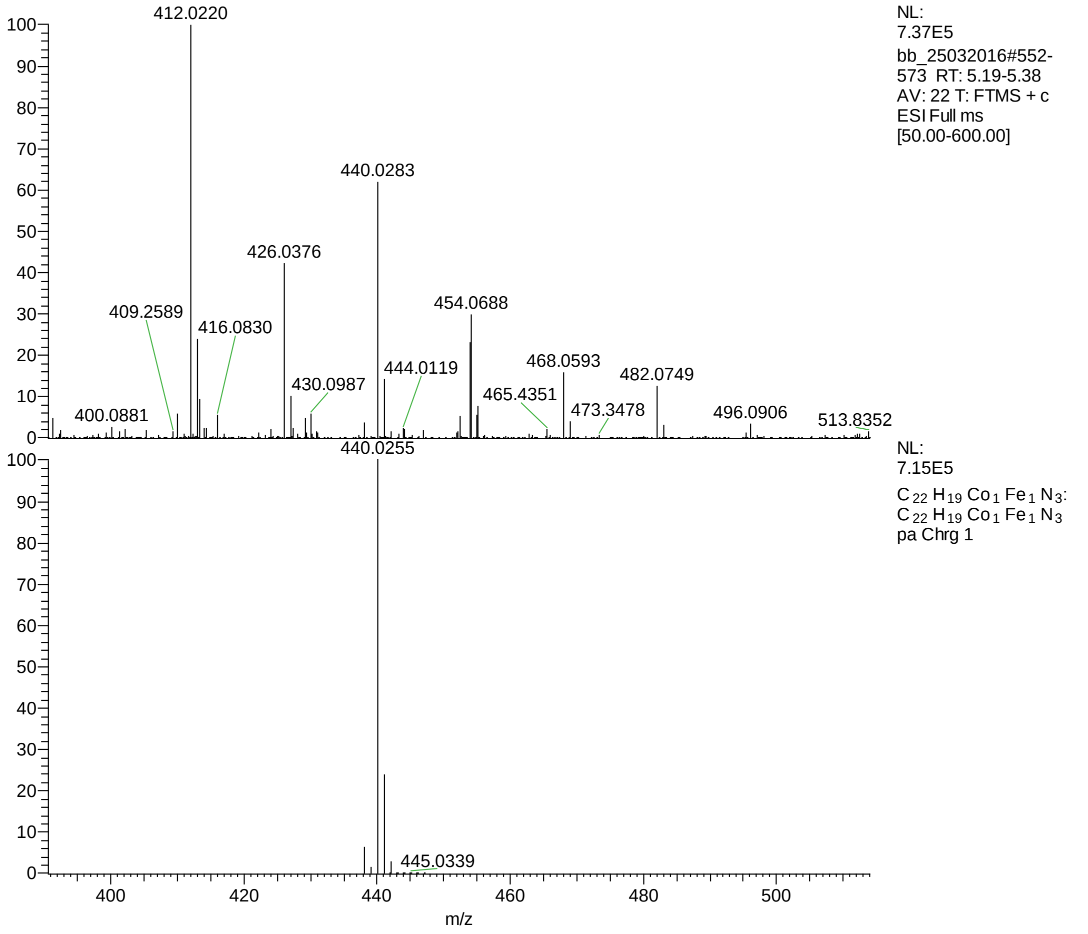

Figure S9. MS (ESI pos, [m/z]; *top*: experimental, *bottom*: simulated) of "1-cobaltoceniumyl-4-ferrocenyltriazole hexafluoridophosphate".

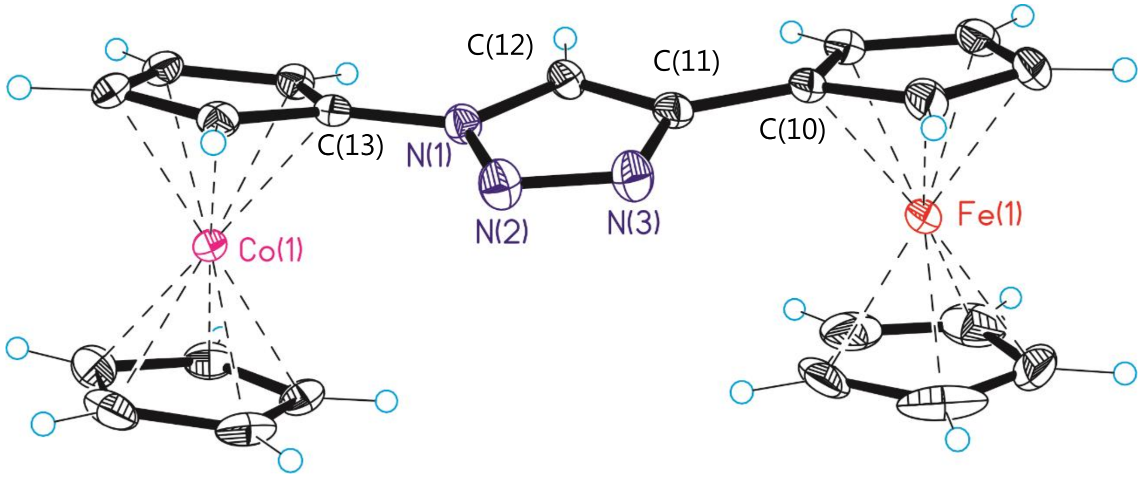

Figure S10. X-ray single crystal structure analysis of "1-cobaltoceniumyl-4-ferrocenyltriazole hexafluoridophosphate" (counterion omitted for clarity).

X-Ray single crystal structure analysis data

Table S7. Crystal data and structure refinement for **6**.

Empirical formula	C22 H19 Co F6 Fe N3 P	
Formula weight	585.15	
Temperature	193(2) K	
Wavelength	0.71073 Å	
Crystal system	Monoclinic	
Space group	$P2_1/n$ (no. 14)	
Unit cell dimensions	a = 5.9289(3) Å	*α* = 90°.
	b = 38.898(2) Å	*ß* = 95.634(2)°.
	c = 9.3684(5) Å	*γ* = 90°.
Volume	2150.1(2) $Å^3$	
Z	4	
Density (calculated)	1.808 Mg/m^3	
Absorption coefficient	1.589 mm^{-1}	
F(000)	1176	
Crystal size	0.180 x 0.120 x 0.050 mm^3	
Theta range for data collection	2.094 to 25.999°.	
Index ranges	-7<h<7, -47<k<47, -9<l<11	
Reflections collected	31310	
Independent reflections	4232 [R(int) = 0.0351]	
Completeness to theta = 25.242°	100.0 %	
Absorption correction	Semi-empirical from equivalents	
Max. and min. transmission	0.891 and 0.807	
Refinement method	Full-matrix least-squares on F^2	
Data / restraints / parameters	4232 / 0 / 308	
Goodness-of-fit on F^2	1.055	
Final R indices [I>2sigma(I)]	R1 = 0.0292, wR2 = 0.0593	
R indices (all data)	R1 = 0.0380, wR2 = 0.0619	
Extinction coefficient	0.00166(13)	
Largest diff. peak and hole	0.365 and -0.279 e.$Å^{-3}$	

Table S8. Atomic coordinates (x 10^4) and equivalent isotropic displacement parameters ($Å^2$x 10^3) for **6**. U(eq) is defined as one third of the trace of the orthogonalized U^{ij} tensor.

	x	y	z	U(eq)
Co(1)	5206(1)	4341(1)	2571(1)	17(1)
Fe(1)	5640(1)	2973(1)	8351(1)	22(1)
N(1)	4906(3)	3572(1)	3551(2)	21(1)
N(2)	2711(3)	3495(1)	3728(2)	26(1)
N(3)	2758(3)	3252(1)	4690(2)	28(1)
C(1)	3148(5)	3212(1)	9311(4)	58(1)
C(2)	4632(6)	3044(1)	10347(3)	51(1)
C(3)	6759(5)	3179(1)	10286(3)	50(1)
C(4)	6663(5)	3427(1)	9251(3)	50(1)
C(5)	4452(6)	3453(1)	8624(3)	56(1)
C(6)	4174(4)	2658(1)	6787(2)	28(1)
C(7)	5533(4)	2463(1)	7826(3)	33(1)
C(8)	7782(4)	2586(1)	7891(3)	32(1)
C(9)	7853(4)	2859(1)	6890(2)	27(1)
C(10)	5613(4)	2905(1)	6202(2)	22(1)
C(11)	4968(4)	3168(1)	5135(2)	22(1)
C(12)	6337(4)	3372(1)	4408(2)	22(1)
C(13)	5445(3)	3814(1)	2529(2)	20(1)
C(14)	3975(4)	3934(1)	1350(2)	21(1)
C(15)	5264(4)	4156(1)	548(2)	24(1)
C(16)	7492(4)	4176(1)	1244(2)	25(1)
C(17)	7612(4)	3966(1)	2490(2)	22(1)
C(18)	4940(4)	4526(1)	4582(2)	29(1)
C(19)	2793(4)	4521(1)	3774(3)	32(1)
C(20)	2920(4)	4730(1)	2551(3)	35(1)
C(21)	5144(4)	4864(1)	2610(3)	35(1)
C(22)	6380(4)	4737(1)	3858(3)	29(1)
P(1)	-150(1)	5644(1)	2425(1)	24(1)
F(1)	16(2)	5321(1)	3487(2)	41(1)
F(2)	-338(2)	5961(1)	1346(2)	43(1)
F(3)	-892(3)	5392(1)	1104(2)	50(1)
F(4)	587(3)	5889(1)	3743(2)	53(1)
F(5)	2425(2)	5588(1)	2152(2)	47(1)
F(6)	-2739(2)	5692(1)	2684(2)	48(1)

Table S9. Bond lengths [Å] and angles [°] for **6**.

Co(1)-C(20)	2.030(2)	C(6)-C(10)	1.429(3)
Co(1)-C(19)	2.030(2)	C(6)-H(6)	0.9500
Co(1)-C(16)	2.031(2)	C(7)-C(8)	1.412(4)
Co(1)-C(15)	2.032(2)	C(7)-H(7)	0.9500
Co(1)-C(22)	2.036(2)	C(8)-C(9)	1.420(3)
Co(1)-C(18)	2.036(2)	C(8)-H(8)	0.9500
Co(1)-C(21)	2.036(2)	C(9)-C(10)	1.430(3)
Co(1)-C(14)	2.046(2)	C(9)-H(9)	0.9500
Co(1)-C(17)	2.046(2)	C(10)-C(11)	1.454(3)
Co(1)-C(13)	2.057(2)	C(11)-C(12)	1.365(3)
Fe(1)-C(5)	2.020(2)	C(12)-H(12)	0.9500
Fe(1)-C(4)	2.022(3)	C(13)-C(14)	1.418(3)
Fe(1)-C(1)	2.028(3)	C(13)-C(17)	1.419(3)
Fe(1)-C(10)	2.030(2)	C(14)-C(15)	1.416(3)
Fe(1)-C(3)	2.032(3)	C(14)-H(14)	0.9500
Fe(1)-C(9)	2.036(2)	C(15)-C(16)	1.417(3)
Fe(1)-C(2)	2.037(3)	C(15)-H(15)	0.9500
Fe(1)-C(6)	2.039(2)	C(16)-C(17)	1.421(3)
Fe(1)-C(8)	2.043(2)	C(16)-H(16)	0.9500
Fe(1)-C(7)	2.045(2)	C(17)-H(17)	0.9500
N(1)-C(12)	1.354(3)	C(18)-C(22)	1.406(3)
N(1)-N(2)	1.362(2)	C(18)-C(19)	1.415(3)
N(1)-C(13)	1.400(3)	C(18)-H(18)	0.9500
N(2)-N(3)	1.306(3)	C(19)-C(20)	1.411(4)
N(3)-C(11)	1.374(3)	C(19)-H(19)	0.9500
C(1)-C(2)	1.404(5)	C(20)-C(21)	1.415(4)
C(1)-C(5)	1.411(5)	C(20)-H(20)	0.9500
C(1)-H(1)	0.9500	C(21)-C(22)	1.408(4)
C(2)-C(3)	1.371(4)	C(21)-H(21)	0.9500
C(2)-H(2)	0.9500	C(22)-H(22)	0.9500
C(3)-C(4)	1.366(4)	P(1)-F(4)	1.5859(16)
C(3)-H(3)	0.9500	P(1)-F(5)	1.5871(14)
C(4)-C(5)	1.387(4)	P(1)-F(6)	1.5884(14)
C(4)-H(4)	0.9500	P(1)-F(2)	1.5903(14)
C(5)-H(5)	0.9500	P(1)-F(1)	1.5999(14)
C(6)-C(7)	1.421(3)	P(1)-F(3)	1.6066(16)

C(20)-Co(1)-C(19)	40.68(10)	C(20)-Co(1)-C(18)	68.49(10)
C(20)-Co(1)-C(16)	135.60(10)	C(19)-Co(1)-C(18)	40.74(10)
C(19)-Co(1)-C(16)	175.88(10)	C(16)-Co(1)-C(18)	142.61(10)
C(20)-Co(1)-C(15)	109.16(10)	C(15)-Co(1)-C(18)	176.53(9)
C(19)-Co(1)-C(15)	135.84(10)	C(22)-Co(1)-C(18)	40.41(9)
C(16)-Co(1)-C(15)	40.84(9)	C(20)-Co(1)-C(21)	40.73(10)
C(20)-Co(1)-C(22)	68.35(10)	C(19)-Co(1)-C(21)	68.30(10)
C(19)-Co(1)-C(22)	68.22(9)	C(16)-Co(1)-C(21)	109.89(10)
C(16)-Co(1)-C(22)	113.01(9)	C(15)-Co(1)-C(21)	111.92(10)
C(15)-Co(1)-C(22)	141.71(9)	C(22)-Co(1)-C(21)	40.44(10)

C(18)-Co(1)-C(21)	68.14(10)
C(20)-Co(1)-C(14)	111.73(9)
C(19)-Co(1)-C(14)	110.20(9)
C(16)-Co(1)-C(14)	68.69(9)
C(15)-Co(1)-C(14)	40.64(9)
C(22)-Co(1)-C(14)	177.64(9)
C(18)-Co(1)-C(14)	137.27(9)
C(21)-Co(1)-C(14)	141.04(10)
C(20)-Co(1)-C(17)	176.22(10)
C(19)-Co(1)-C(17)	142.96(10)
C(16)-Co(1)-C(17)	40.80(9)
C(15)-Co(1)-C(17)	68.71(9)
C(22)-Co(1)-C(17)	111.21(9)
C(18)-Co(1)-C(17)	113.79(9)
C(21)-Co(1)-C(17)	136.60(10)
C(14)-Co(1)-C(17)	68.88(9)
C(20)-Co(1)-C(13)	142.19(10)
C(19)-Co(1)-C(13)	114.10(9)
C(16)-Co(1)-C(13)	67.84(8)
C(15)-Co(1)-C(13)	67.72(9)
C(22)-Co(1)-C(13)	138.21(9)
C(18)-Co(1)-C(13)	112.41(9)
C(21)-Co(1)-C(13)	177.08(10)
C(14)-Co(1)-C(13)	40.43(8)
C(17)-Co(1)-C(13)	40.48(8)
C(5)-Fe(1)-C(4)	40.13(13)
C(5)-Fe(1)-C(1)	40.81(13)
C(4)-Fe(1)-C(1)	67.54(12)
C(5)-Fe(1)-C(10)	106.09(10)
C(4)-Fe(1)-C(10)	120.02(11)
C(1)-Fe(1)-C(10)	124.36(12)
C(5)-Fe(1)-C(3)	67.14(12)
C(4)-Fe(1)-C(3)	39.39(12)
C(1)-Fe(1)-C(3)	67.23(12)
C(10)-Fe(1)-C(3)	155.06(12)
C(5)-Fe(1)-C(9)	122.83(12)
C(4)-Fe(1)-C(9)	106.49(11)
C(1)-Fe(1)-C(9)	160.65(13)
C(10)-Fe(1)-C(9)	41.17(9)
C(3)-Fe(1)-C(9)	120.68(12)
C(5)-Fe(1)-C(2)	67.74(12)
C(4)-Fe(1)-C(2)	66.55(12)
C(1)-Fe(1)-C(2)	40.42(13)
C(10)-Fe(1)-C(2)	162.55(13)
C(3)-Fe(1)-C(2)	39.38(13)
C(9)-Fe(1)-C(2)	155.98(12)
C(5)-Fe(1)-C(6)	121.14(12)
C(4)-Fe(1)-C(6)	155.97(12)
C(1)-Fe(1)-C(6)	108.43(11)
C(10)-Fe(1)-C(6)	41.13(9)
C(3)-Fe(1)-C(6)	163.06(12)
C(9)-Fe(1)-C(6)	69.07(9)
C(2)-Fe(1)-C(6)	126.78(12)
C(5)-Fe(1)-C(8)	159.97(13)
C(4)-Fe(1)-C(8)	124.32(12)
C(1)-Fe(1)-C(8)	157.77(13)
C(10)-Fe(1)-C(8)	68.84(9)
C(3)-Fe(1)-C(8)	108.82(11)
C(9)-Fe(1)-C(8)	40.76(9)
C(2)-Fe(1)-C(8)	122.56(11)
C(6)-Fe(1)-C(8)	68.60(10)
C(5)-Fe(1)-C(7)	157.59(14)
C(4)-Fe(1)-C(7)	161.47(13)
C(1)-Fe(1)-C(7)	122.96(12)
C(10)-Fe(1)-C(7)	68.73(9)
C(3)-Fe(1)-C(7)	126.58(11)
C(9)-Fe(1)-C(7)	68.48(9)
C(2)-Fe(1)-C(7)	110.36(10)
C(6)-Fe(1)-C(7)	40.73(9)
C(8)-Fe(1)-C(7)	40.43(10)
C(12)-N(1)-N(2)	110.62(17)
C(12)-N(1)-C(13)	128.23(18)
N(2)-N(1)-C(13)	121.04(17)
N(3)-N(2)-N(1)	106.72(17)
N(2)-N(3)-C(11)	109.61(18)
C(2)-C(1)-C(5)	106.8(3)
C(2)-C(1)-Fe(1)	70.14(16)
C(5)-C(1)-Fe(1)	69.29(15)
C(2)-C(1)-H(1)	126.6
C(5)-C(1)-H(1)	126.6
Fe(1)-C(1)-H(1)	125.6
C(3)-C(2)-C(1)	108.1(3)
C(3)-C(2)-Fe(1)	70.09(16)
C(1)-C(2)-Fe(1)	69.44(16)
C(3)-C(2)-H(2)	125.9
C(1)-C(2)-H(2)	125.9
Fe(1)-C(2)-H(2)	126.1
C(4)-C(3)-C(2)	108.9(3)
C(4)-C(3)-Fe(1)	69.94(16)
C(2)-C(3)-Fe(1)	70.53(17)
C(4)-C(3)-H(3)	125.5
C(2)-C(3)-H(3)	125.5
Fe(1)-C(3)-H(3)	125.6
C(3)-C(4)-C(5)	109.0(3)
C(3)-C(4)-Fe(1)	70.68(16)
C(5)-C(4)-Fe(1)	69.85(16)
C(3)-C(4)-H(4)	125.5
C(5)-C(4)-H(4)	125.5
Fe(1)-C(4)-H(4)	125.5
C(4)-C(5)-C(1)	107.2(3)
C(4)-C(5)-Fe(1)	70.03(15)
C(1)-C(5)-Fe(1)	69.91(15)
C(4)-C(5)-H(5)	126.4
C(1)-C(5)-H(5)	126.4
Fe(1)-C(5)-H(5)	125.2
C(7)-C(6)-C(10)	107.6(2)

C(7)-C(6)-Fe(1)	69.86(13)	C(15)-C(16)-C(17)	108.33(19)
C(10)-C(6)-Fe(1)	69.10(12)	C(15)-C(16)-Co(1)	69.62(12)
C(7)-C(6)-H(6)	126.2	C(17)-C(16)-Co(1)	70.18(12)
C(10)-C(6)-H(6)	126.2	C(15)-C(16)-H(16)	125.8
Fe(1)-C(6)-H(6)	126.4	C(17)-C(16)-H(16)	125.8
C(8)-C(7)-C(6)	108.5(2)	Co(1)-C(16)-H(16)	125.9
C(8)-C(7)-Fe(1)	69.73(13)	C(13)-C(17)-C(16)	106.84(19)
C(6)-C(7)-Fe(1)	69.42(12)	C(13)-C(17)-Co(1)	70.17(12)
C(8)-C(7)-H(7)	125.7	C(16)-C(17)-Co(1)	69.02(12)
C(6)-C(7)-H(7)	125.7	C(13)-C(17)-H(17)	126.6
Fe(1)-C(7)-H(7)	126.7	C(16)-C(17)-H(17)	126.6
C(7)-C(8)-C(9)	108.3(2)	Co(1)-C(17)-H(17)	125.8
C(7)-C(8)-Fe(1)	69.85(13)	C(22)-C(18)-C(19)	107.8(2)
C(9)-C(8)-Fe(1)	69.37(13)	C(22)-C(18)-Co(1)	69.78(13)
C(7)-C(8)-H(8)	125.9	C(19)-C(18)-Co(1)	69.40(13)
C(9)-C(8)-H(8)	125.9	C(22)-C(18)-H(18)	126.1
Fe(1)-C(8)-H(8)	126.5	C(19)-C(18)-H(18)	126.1
C(8)-C(9)-C(10)	107.8(2)	Co(1)-C(18)-H(18)	126.3
C(8)-C(9)-Fe(1)	69.88(13)	C(20)-C(19)-C(18)	108.1(2)
C(10)-C(9)-Fe(1)	69.17(12)	C(20)-C(19)-Co(1)	69.64(13)
C(8)-C(9)-H(9)	126.1	C(18)-C(19)-Co(1)	69.86(12)
C(10)-C(9)-H(9)	126.1	C(20)-C(19)-H(19)	126.0
Fe(1)-C(9)-H(9)	126.4	C(18)-C(19)-H(19)	126.0
C(6)-C(10)-C(9)	107.81(19)	Co(1)-C(19)-H(19)	126.1
C(6)-C(10)-C(11)	127.6(2)	C(19)-C(20)-C(21)	107.7(2)
C(9)-C(10)-C(11)	124.6(2)	C(19)-C(20)-Co(1)	69.67(13)
C(6)-C(10)-Fe(1)	69.77(12)	C(21)-C(20)-Co(1)	69.89(13)
C(9)-C(10)-Fe(1)	69.66(12)	C(19)-C(20)-H(20)	126.1
C(11)-C(10)-Fe(1)	124.44(15)	C(21)-C(20)-H(20)	126.1
C(12)-C(11)-N(3)	107.89(19)	Co(1)-C(20)-H(20)	125.9
C(12)-C(11)-C(10)	128.6(2)	C(22)-C(21)-C(20)	108.0(2)
N(3)-C(11)-C(10)	123.55(19)	C(22)-C(21)-Co(1)	69.76(13)
N(1)-C(12)-C(11)	105.17(19)	C(20)-C(21)-Co(1)	69.38(13)
N(1)-C(12)-H(12)	127.4	C(22)-C(21)-H(21)	126.0
C(11)-C(12)-H(12)	127.4	C(20)-C(21)-H(21)	126.0
N(1)-C(13)-C(14)	126.18(19)	Co(1)-C(21)-H(21)	126.4
N(1)-C(13)-C(17)	124.48(19)	C(18)-C(22)-C(21)	108.4(2)
C(14)-C(13)-C(17)	109.29(19)	C(18)-C(22)-Co(1)	69.81(13)
N(1)-C(13)-Co(1)	129.46(14)	C(21)-C(22)-Co(1)	69.80(13)
C(14)-C(13)-Co(1)	69.36(12)	C(18)-C(22)-H(22)	125.8
C(17)-C(13)-Co(1)	69.35(12)	C(21)-C(22)-H(22)	125.8
C(15)-C(14)-C(13)	107.00(19)	Co(1)-C(22)-H(22)	126.1
C(15)-C(14)-Co(1)	69.14(12)	F(4)-P(1)-F(5)	90.67(9)
C(13)-C(14)-Co(1)	70.20(12)	F(4)-P(1)-F(6)	90.42(9)
C(15)-C(14)-H(14)	126.5	F(5)-P(1)-F(6)	178.79(10)
C(13)-C(14)-H(14)	126.5	F(4)-P(1)-F(2)	91.57(9)
Co(1)-C(14)-H(14)	125.7	F(5)-P(1)-F(2)	90.66(8)
C(14)-C(15)-C(16)	108.52(19)	F(6)-P(1)-F(2)	89.85(8)
C(14)-C(15)-Co(1)	70.22(12)	F(4)-P(1)-F(1)	89.52(9)
C(16)-C(15)-Co(1)	69.54(12)	F(5)-P(1)-F(1)	89.56(8)
C(14)-C(15)-H(15)	125.7	F(6)-P(1)-F(1)	89.91(8)
C(16)-C(15)-H(15)	125.7	F(2)-P(1)-F(1)	178.89(9)
Co(1)-C(15)-H(15)	126.1	F(4)-P(1)-F(3)	179.24(9)

F(5)-P(1)-F(3)	89.28(9)	F(2)-P(1)-F(3)	89.19(9)
F(6)-P(1)-F(3)	89.63(9)	F(1)-P(1)-F(3)	89.73(9)

Table S10. Anisotropic displacement parameters ($Å^2$x 10^3) for **6**. The anisotropic displacement factor exponent takes the form: $-2p^2 [h^2 a^{*2}U^{11} + ... + 2 h k a^* b^* U^{12}]$.

	U^{11}	U^{22}	U^{33}	U^{23}	U^{13}	U^{12}
Co(1)	19(1)	17(1)	15(1)	-1(1)	2(1)	1(1)
Fe(1)	30(1)	16(1)	21(1)	0(1)	6(1)	2(1)
N(1)	21(1)	19(1)	22(1)	2(1)	3(1)	0(1)
N(2)	21(1)	26(1)	31(1)	5(1)	1(1)	-3(1)
N(3)	27(1)	25(1)	30(1)	5(1)	2(1)	-3(1)
C(1)	30(1)	75(2)	70(2)	-48(2)	10(2)	6(2)
C(2)	93(3)	32(2)	35(2)	-2(1)	36(2)	-1(2)
C(3)	57(2)	53(2)	38(2)	-21(1)	-8(1)	17(2)
C(4)	60(2)	29(1)	66(2)	-25(1)	31(2)	-12(1)
C(5)	109(3)	30(2)	29(2)	-3(1)	8(2)	38(2)
C(6)	36(1)	19(1)	28(1)	-3(1)	3(1)	-3(1)
C(7)	53(2)	16(1)	30(1)	1(1)	10(1)	2(1)
C(8)	42(1)	26(1)	27(1)	0(1)	4(1)	14(1)
C(9)	31(1)	24(1)	26(1)	0(1)	7(1)	7(1)
C(10)	31(1)	17(1)	19(1)	-2(1)	4(1)	3(1)
C(11)	27(1)	17(1)	21(1)	-4(1)	2(1)	1(1)
C(12)	22(1)	21(1)	24(1)	2(1)	0(1)	5(1)
C(13)	23(1)	17(1)	20(1)	-2(1)	5(1)	3(1)
C(14)	24(1)	20(1)	19(1)	-6(1)	-2(1)	1(1)
C(15)	33(1)	25(1)	14(1)	-3(1)	4(1)	5(1)
C(16)	27(1)	26(1)	23(1)	-1(1)	11(1)	1(1)
C(17)	21(1)	23(1)	23(1)	0(1)	3(1)	4(1)
C(18)	38(1)	32(1)	18(1)	-7(1)	6(1)	3(1)
C(19)	24(1)	33(1)	42(2)	-15(1)	14(1)	-2(1)
C(20)	37(1)	28(1)	39(2)	-11(1)	-6(1)	15(1)
C(21)	53(2)	16(1)	38(2)	-2(1)	11(1)	-1(1)
C(22)	27(1)	27(1)	33(1)	-14(1)	3(1)	-4(1)
P(1)	20(1)	31(1)	21(1)	6(1)	4(1)	1(1)
F(1)	44(1)	43(1)	36(1)	19(1)	7(1)	3(1)
F(2)	44(1)	42(1)	43(1)	21(1)	10(1)	6(1)
F(3)	63(1)	51(1)	33(1)	-9(1)	-4(1)	-3(1)
F(4)	66(1)	50(1)	42(1)	-13(1)	-5(1)	-4(1)
F(5)	24(1)	72(1)	48(1)	20(1)	12(1)	10(1)
F(6)	24(1)	80(1)	41(1)	19(1)	12(1)	10(1)

Table S11. Hydrogen coordinates (x 10^4) and isotropic displacement parameters ($Å^2$x 10^3) for **6**.

	x	y	z	U(eq)
H(1)	1569	3171	9111	70
H(2)	4231	2869	10980	62
H(3)	8084	3110	10869	60
H(4)	7909	3560	9001	60
H(5)	3916	3605	7872	67
H(6)	2596	2630	6528	33
H(7)	5016	2280	8383	39
H(8)	9033	2501	8501	38
H(9)	9156	2988	6710	32
H(12)	7946	3374	4486	27
H(14)	2421	3877	1138	25
H(15)	4725	4271	-312	29
H(16)	8700	4308	932	30
H(17)	8896	3934	3167	27
H(18)	5336	4407	5457	35
H(19)	1495	4399	4012	39
H(20)	1725	4772	1821	42
H(21)	5706	5014	1927	42
H(22)	7924	4785	4160	35

Table 12. Torsion angles [°] for **6**.

C(12)-N(1)-N(2)-N(3)	0.4(2)	N(2)-N(1)-C(12)-C(11)	-0.3(2)
C(13)-N(1)-N(2)-N(3)	176.75(18)	C(13)-N(1)-C(12)-C(11)	-176.32(19)
N(1)-N(2)-N(3)-C(11)	-0.3(2)	N(3)-C(11)-C(12)-N(1)	0.1(2)
C(5)-C(1)-C(2)-C(3)	-0.2(3)	C(10)-C(11)-C(12)-N(1)	-179.9(2)
Fe(1)-C(1)-C(2)-C(3)	59.63(19)	C(12)-N(1)-C(13)-C(14)	157.9(2)
C(5)-C(1)-C(2)-Fe(1)	-59.85(18)	N(2)-N(1)-C(13)-C(14)	-17.8(3)
C(1)-C(2)-C(3)-C(4)	0.4(3)	C(12)-N(1)-C(13)-C(17)	-19.4(3)
Fe(1)-C(2)-C(3)-C(4)	59.62(19)	N(2)-N(1)-C(13)-C(17)	164.95(19)
C(1)-C(2)-C(3)-Fe(1)	-59.23(19)	C(12)-N(1)-C(13)-Co(1)	-110.0(2)
C(2)-C(3)-C(4)-C(5)	-0.4(3)	N(2)-N(1)-C(13)-Co(1)	74.3(2)
Fe(1)-C(3)-C(4)-C(5)	59.57(19)	N(1)-C(13)-C(14)-C(15)	-175.91(19)
C(2)-C(3)-C(4)-Fe(1)	-59.98(19)	C(17)-C(13)-C(14)-C(15)	1.7(2)
C(3)-C(4)-C(5)-C(1)	0.3(3)	Co(1)-C(13)-C(14)-C(15)	59.62(14)
Fe(1)-C(4)-C(5)-C(1)	60.35(18)	N(1)-C(13)-C(14)-Co(1)	124.5(2)
C(3)-C(4)-C(5)-Fe(1)	-60.08(19)	C(17)-C(13)-C(14)-Co(1)	-57.92(15)
C(2)-C(1)-C(5)-C(4)	0.0(3)	C(13)-C(14)-C(15)-C(16)	-1.1(2)
Fe(1)-C(1)-C(5)-C(4)	-60.43(18)	Co(1)-C(14)-C(15)-C(16)	59.18(15)
C(2)-C(1)-C(5)-Fe(1)	60.40(18)	C(13)-C(14)-C(15)-Co(1)	-60.30(14)
C(10)-C(6)-C(7)-C(8)	0.1(3)	C(14)-C(15)-C(16)-C(17)	0.1(2)
Fe(1)-C(6)-C(7)-C(8)	-58.91(16)	Co(1)-C(15)-C(16)-C(17)	59.73(15)
C(10)-C(6)-C(7)-Fe(1)	59.00(15)	C(14)-C(15)-C(16)-Co(1)	-59.61(14)
C(6)-C(7)-C(8)-C(9)	-0.2(3)	N(1)-C(13)-C(17)-C(16)	176.04(19)
Fe(1)-C(7)-C(8)-C(9)	-58.89(16)	C(14)-C(13)-C(17)-C(16)	-1.6(2)
C(6)-C(7)-C(8)-Fe(1)	58.72(16)	Co(1)-C(13)-C(17)-C(16)	-59.55(14)
C(7)-C(8)-C(9)-C(10)	0.2(3)	N(1)-C(13)-C(17)-Co(1)	-124.4(2)
Fe(1)-C(8)-C(9)-C(10)	-59.01(15)	C(14)-C(13)-C(17)-Co(1)	57.93(15)
C(7)-C(8)-C(9)-Fe(1)	59.19(16)	C(15)-C(16)-C(17)-C(13)	0.9(2)
C(7)-C(6)-C(10)-C(9)	0.0(2)	Co(1)-C(16)-C(17)-C(13)	60.29(14)
Fe(1)-C(6)-C(10)-C(9)	59.50(15)	C(15)-C(16)-C(17)-Co(1)	-59.38(15)
C(7)-C(6)-C(10)-C(11)	-178.0(2)	C(22)-C(18)-C(19)-C(20)	0.1(3)
Fe(1)-C(6)-C(10)-C(11)	-118.5(2)	Co(1)-C(18)-C(19)-C(20)	-59.37(16)
C(7)-C(6)-C(10)-Fe(1)	-59.48(15)	C(22)-C(18)-C(19)-Co(1)	59.43(16)
C(8)-C(9)-C(10)-C(6)	-0.1(2)	C(18)-C(19)-C(20)-C(21)	-0.3(3)
Fe(1)-C(9)-C(10)-C(6)	-59.57(15)	Co(1)-C(19)-C(20)-C(21)	-59.77(16)
C(8)-C(9)-C(10)-C(11)	177.9(2)	C(18)-C(19)-C(20)-Co(1)	59.51(16)
Fe(1)-C(9)-C(10)-C(11)	118.5(2)	C(19)-C(20)-C(21)-C(22)	0.4(3)
C(8)-C(9)-C(10)-Fe(1)	59.45(15)	Co(1)-C(20)-C(21)-C(22)	-59.27(16)
N(2)-N(3)-C(11)-C(12)	0.2(2)	C(19)-C(20)-C(21)-Co(1)	59.63(16)
N(2)-N(3)-C(11)-C(10)	-179.86(19)	C(19)-C(18)-C(22)-C(21)	0.2(3)
C(6)-C(10)-C(11)-C(12)	-169.4(2)	Co(1)-C(18)-C(22)-C(21)	59.36(16)
C(9)-C(10)-C(11)-C(12)	12.9(4)	C(19)-C(18)-C(22)-Co(1)	-59.19(16)
Fe(1)-C(10)-C(11)-C(12)	100.7(3)	C(20)-C(21)-C(22)-C(18)	-0.3(3)
C(6)-C(10)-C(11)-N(3)	10.6(3)	Co(1)-C(21)-C(22)-C(18)	-59.37(16)
C(9)-C(10)-C(11)-N(3)	-167.0(2)	C(20)-C(21)-C(22)-Co(1)	59.04(16)
Fe(1)-C(10)-C(11)-N(3)	-79.3(3)		

Triazole (7) – "1,4-dicobaltoceniumyltriazole bis(hexafluoridophosphate)"

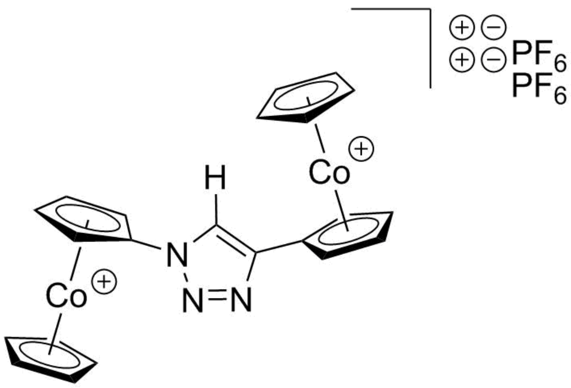

[733.20]

$C_{22}H_{19}N_3Co_2(PF_6)_2$

Analytical data:

IR (ATR [cm^{-1}]): 3121 ($\nu_{C–H}$), 1603, 1540, 1459, 1418 ($\nu_{C=C}$), 1208, 1048, 1024, 816 ($\nu_{P–F}$), 555 ($\nu_{P–F}$), 494, 479, 443.

^{1}H-NMR (300 MHz, C_3D_6O, [ppm]): δ 5.84 (s, 5H, 4-Cc-Cp), 6.01 (s, 5H, 1-Cc-Cp), 6.12 (pseudo-t, 2H, *J* = 2.1 Hz, C3/C4 of substituted 4-Cc-Cp), 6.15 (pseudo-t, 2H, *J* = 2.3 Hz, C3/C4 of substituted 1-Cc-Cp), 6.48 (pseudo-t, 2H, *J* = 2.1 Hz, C2/C5 of substituted 4-Cc-Cp), 6.70 (pseudo-t, 2H, J = 2.3 Hz, C2/C5 of substituted 1-Cc-Cp), 9.15 (s, 1H, CH of triazole).

^{13}C-NMR (75 MHz, C_3D_6O, [ppm]): δ 77.0 (C3/C4 of substituted 4-Cc-Cp), 82.3 (C2/C5 of substituted 4-Cc-Cp), 83.8 (C3/C4 of substituted 1-Cc-Cp), 85.9 (C2/C5 of substituted 1-Cc-Cp), 87.1 (4-Cc-Cp), 87.7 (1-Cc-Cp), 90.0 (quart. carbon of substituted 4-Cc-Cp), 95.0 (quart. carbon of substituted 1-Cc-Cp), 125.4 (CH of triazole), 140.8 (quart. carbon of triazole).

MS (MALDI pos, [m/z]): 588.01 (M^+ - $PF_6^–$).

Melting point [°C]: 231.

Spectra

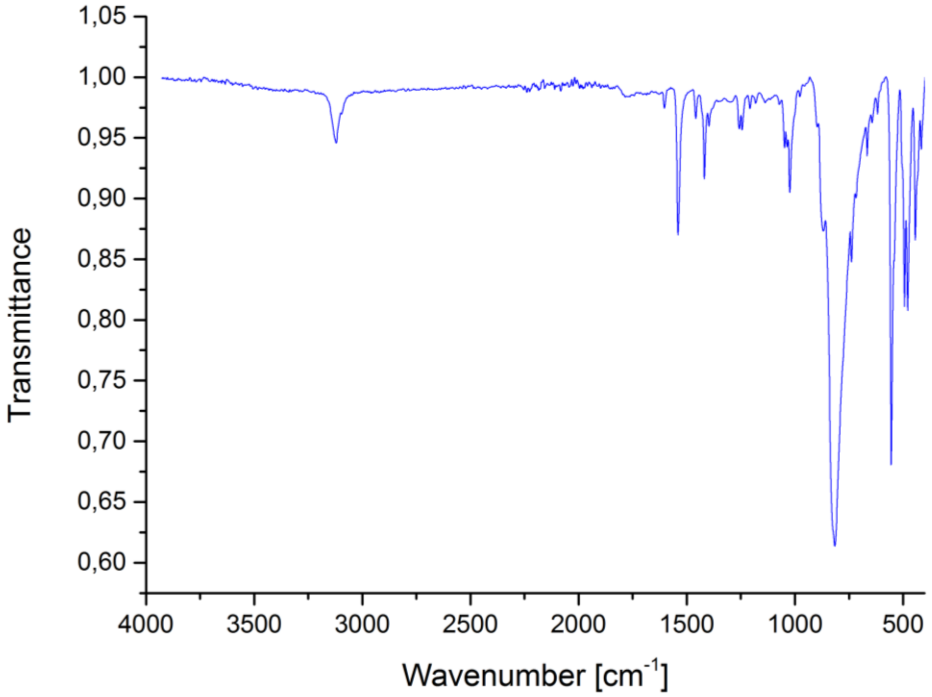

Figure S11. IR-spectrum (ATR, [cm^{-1}]) of "1,4-dicobaltoceniumyltriazole bis(hexafluoridophosphate)".

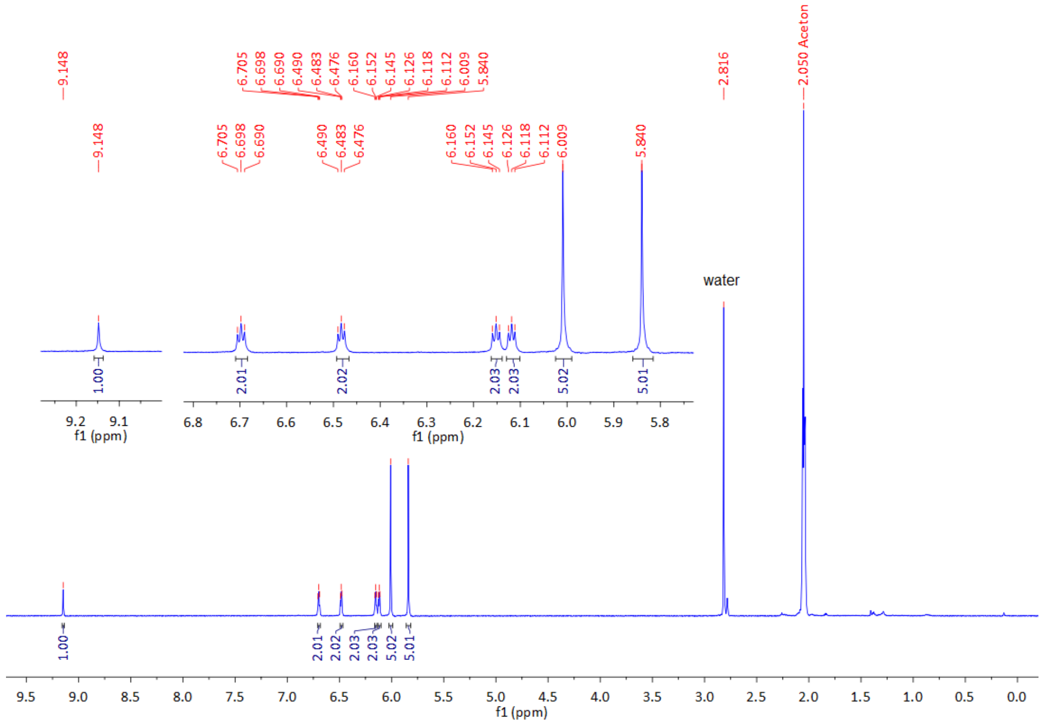

Figure S12. ^{1}H-NMR (300 MHz, C_3D_6O, [ppm]) of “1,4-dicobaltoceniumyltriazole bis(hexafluoridophosphate)“.

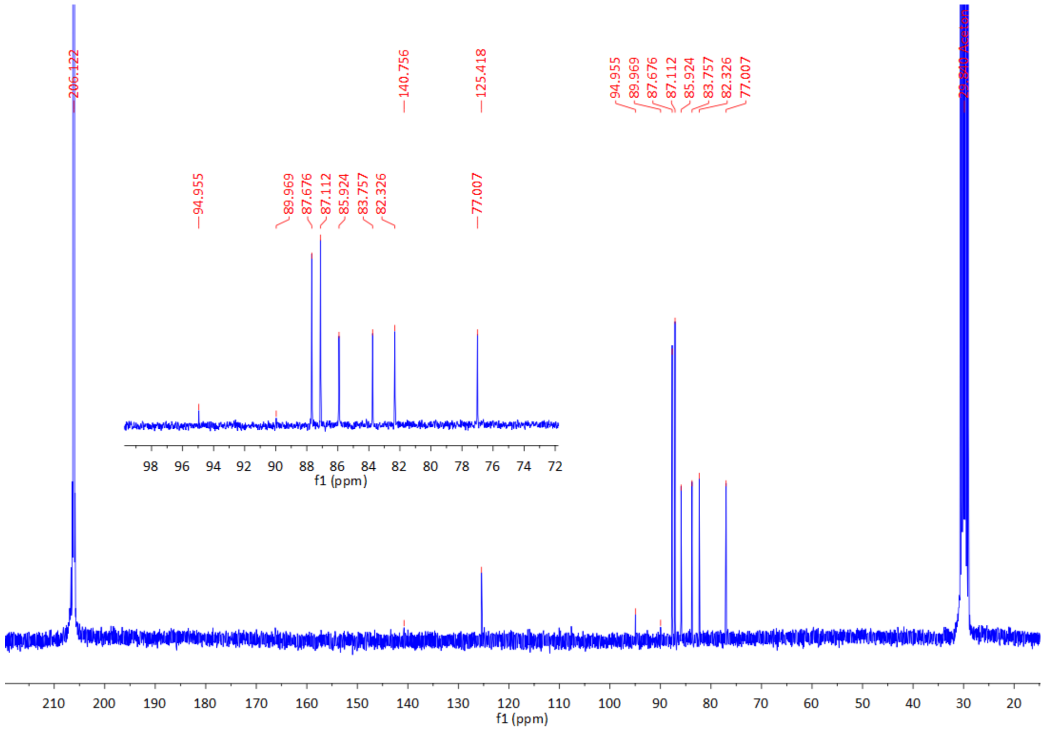

Figure S13. ^{13}C-NMR (75 MHz, C_3D_6O, [ppm]) of “1,4-dicobaltoceniumyltriazole bis(hexafluoridophosphate)“.

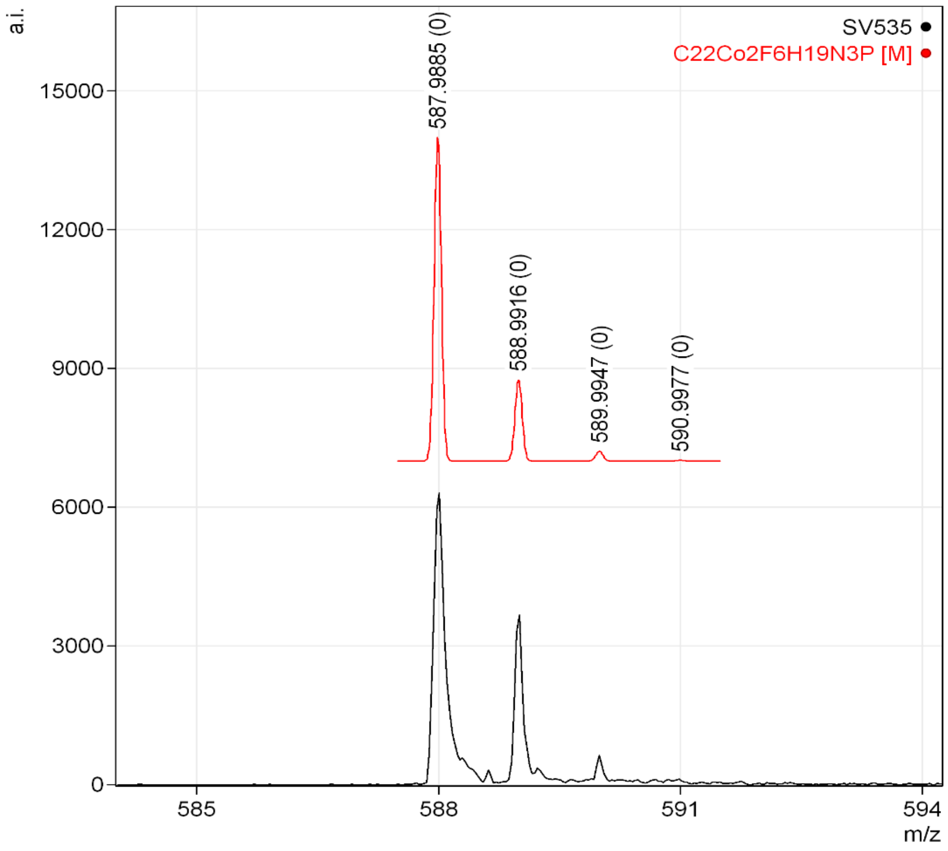

Figure S14. MS (MALDI pos, [m/z]; *top*: simulated, *bottom*: experimental) of “1,4-dicobaltoceniumyltriazole bis(hexafluoridophosphate)“.

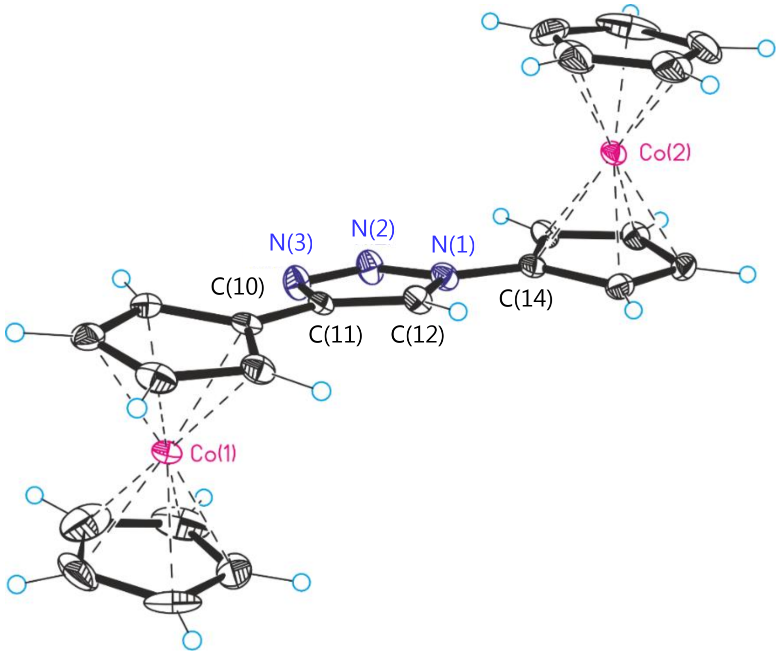

Figure S15. X-ray single crystal structure analysis of “1,4-dicobaltoceniumyltriazole bis(hexafluoridophosphate)“ (counterions omitted for clarity).

X-Ray single crystal structure analysis data

Table S13. Crystal data and structure refinement for **7**.

Empirical formula	C22 H19 Co2 F12 N3 P2	
Formula weight	733.20	
Temperature	193(2) K	
Wavelength	0.71073 Å	
Crystal system	Monoclinic	
Space group	$P2_1/c$ (no. 14)	
Unit cell dimensions	a = 10.6551(16) Å	α = 90°.
	b = 17.215(3) Å	ß = 102.934(5)°.
	c = 14.297(2) Å	γ = 90°.
Volume	2556.0(7) $Å^3$	
Z	4	
Density (calculated)	1.905 Mg/m^3	
Absorption coefficient	1.531 mm^{-1}	
F(000)	1456	
Crystal size	0.180 x 0.110 x 0.080 mm^3	
Theta range for data collection	2.290 to 25.482°.	
Index ranges	-12<h<12, -20<k<20, -17<l<17	
Reflections collected	39515	
Independent reflections	4739 [R(int) = 0.0369]	
Completeness to theta = 25.242°	99.9 %	
Absorption correction	Semi-empirical from equivalents	
Max. and min. transmission	0.862 and 0.787	
Refinement method	Full-matrix least-squares on F^2	
Data / restraints / parameters	4739 / 0 / 460	
Goodness-of-fit on F^2	1.047	
Final R indices [I>2sigma(I)]	R1 = 0.0350, wR2 = 0.0862	
R indices (all data)	R1 = 0.0413, wR2 = 0.0896	
Extinction coefficient	n/a	
Largest diff. peak and hole	1.269 and -0.488 e.$Å^{-3}$	

Table S14. Atomic coordinates (x 10^4) and equivalent isotropic displacement parameters ($Å^2$x 10^3) for **7**. U(eq) is defined as one third of the trace of the orthogonalized U^{ij} tensor.

	x	y	z	U(eq)
Co(1)	10445(1)	2767(1)	4930(1)	25(1)
Co(2)	3971(1)	5007(1)	2238(1)	27(1)
N(1)	6304(2)	4278(1)	3686(2)	33(1)
N(2)	7021(2)	4730(2)	4394(2)	38(1)
N(3)	8204(2)	4494(2)	4540(2)	38(1)
C(1)	11163(6)	2781(3)	6339(3)	92(2)
C(2)	11499(4)	2100(4)	5949(3)	80(2)
C(3)	10399(5)	1709(2)	5530(3)	61(1)
C(4)	9370(4)	2137(3)	5667(3)	58(1)
C(5)	9819(6)	2796(3)	6157(3)	73(2)
C(6)	10686(3)	3836(2)	4384(2)	34(1)
C(7)	11644(3)	3315(2)	4225(2)	38(1)
C(8)	11026(3)	2682(2)	3677(2)	39(1)
C(9)	9676(3)	2812(2)	3492(2)	33(1)
C(10)	9476(3)	3531(2)	3929(2)	29(1)
C(11)	8264(2)	3892(1)	3928(2)	23(1)
C(12)	7072(3)	3747(2)	3386(2)	31(1)
C(14)	4945(3)	4398(2)	3402(2)	28(1)
C(15)	4038(3)	3913(2)	2792(2)	33(1)
C(16)	2805(3)	4252(2)	2729(2)	35(1)
C(17)	2960(3)	4939(2)	3287(2)	36(1)
C(18)	4292(3)	5037(2)	3701(2)	33(1)
C(19)	3920(4)	4941(2)	815(2)	56(1)
C(20)	2992(4)	5451(3)	974(3)	67(1)
C(21)	3627(7)	6035(3)	1582(3)	92(2)
C(22)	4933(6)	5874(3)	1776(3)	88(2)
C(23)	5096(4)	5206(3)	1308(3)	73(1)
P(1)	9100(1)	5265(1)	1796(1)	33(1)
P(2)	5448(1)	2463(1)	649(1)	38(1)
F(1)	10104(2)	5961(1)	2049(2)	65(1)
F(2)	8097(2)	4581(1)	1509(2)	77(1)
F(3)	9972(4)	4665(3)	2456(4)	78(2)
F(4)	8163(6)	5833(3)	1099(5)	68(2)
F(5)	8357(6)	5449(3)	2600(4)	76(2)
F(6)	9764(6)	5056(3)	955(3)	67(2)
F(7)	6085(6)	3231(3)	1122(4)	105(2)
F(8)	4809(4)	1682(2)	203(3)	69(1)
F(9)	4711(5)	2369(3)	1488(3)	86(1)
F(10)	6184(4)	2551(3)	-177(3)	72(1)
F(11)	6603(4)	1983(3)	1276(3)	79(2)
F(12)	4251(5)	2925(3)	47(4)	72(1)
F(3A)	10124(16)	4801(10)	1440(20)	193(13)
F(4A)	8234(11)	5855(8)	2220(15)	116(6)
F(5A)	9816(19)	5109(11)	2884(10)	150(8)
F(6A)	8440(20)	5600(13)	834(12)	142(10)
F(7A)	6306(14)	3086(13)	379(19)	149(10)

F(8A)	4461(11)	1814(9)	851(17)	113(6)
F(9A)	5960(40)	2545(11)	1695(12)	249(17)
F(10A)	4940(30)	2362(11)	-444(11)	181(10)
F(11A)	6460(20)	1819(10)	676(17)	131(8)
F(12A)	4460(20)	3093(12)	611(18)	137(9)

Table S15. Bond lengths [Å] and angles [°] for **7**.

Co(1)-C(1)	1.989(4)	C(7)-H(7)	0.9500
Co(1)-C(2)	1.993(4)	C(8)-C(9)	1.421(4)
Co(1)-C(5)	2.013(3)	C(8)-H(8)	0.9500
Co(1)-C(3)	2.020(3)	C(9)-C(10)	1.424(4)
Co(1)-C(8)	2.026(3)	C(9)-H(9)	0.9500
Co(1)-C(7)	2.027(3)	C(10)-C(11)	1.433(4)
Co(1)-C(4)	2.035(3)	C(11)-C(12)	1.354(4)
Co(1)-C(6)	2.037(3)	C(12)-H(12)	0.9500
Co(1)-C(9)	2.037(3)	C(14)-C(18)	1.418(4)
Co(1)-C(10)	2.044(3)	C(14)-C(15)	1.419(4)
Co(2)-C(21)	1.999(4)	C(15)-C(16)	1.421(4)
Co(2)-C(22)	2.006(4)	C(15)-H(15)	0.9500
Co(2)-C(23)	2.010(4)	C(16)-C(17)	1.416(4)
Co(2)-C(20)	2.024(3)	C(16)-H(16)	0.9500
Co(2)-C(19)	2.026(3)	C(17)-C(18)	1.420(4)
Co(2)-C(16)	2.028(3)	C(17)-H(17)	0.9500
Co(2)-C(17)	2.037(3)	C(18)-H(18)	0.9500
Co(2)-C(15)	2.037(3)	C(19)-C(23)	1.372(6)
Co(2)-C(14)	2.043(3)	C(19)-C(20)	1.379(6)
Co(2)-C(18)	2.043(3)	C(19)-H(19)	0.9500
N(1)-C(12)	1.359(4)	C(20)-C(21)	1.400(7)
N(1)-N(2)	1.366(3)	C(20)-H(20)	0.9500
N(1)-C(14)	1.429(4)	C(21)-C(22)	1.385(8)
N(2)-N(3)	1.296(3)	C(21)-H(21)	0.9500
N(3)-C(11)	1.367(3)	C(22)-C(23)	1.360(7)
C(1)-C(2)	1.380(8)	C(22)-H(22)	0.9500
C(1)-C(5)	1.396(7)	C(23)-H(23)	0.9500
C(1)-H(1)	0.9500	P(1)-F(6A)	1.512(15)
C(2)-C(3)	1.368(7)	P(1)-F(3A)	1.530(13)
C(2)-H(2)	0.9500	P(1)-F(3)	1.558(4)
C(3)-C(4)	1.371(6)	P(1)-F(6)	1.566(4)
C(3)-H(3)	0.9500	P(1)-F(5)	1.567(4)
C(4)-C(5)	1.362(6)	P(1)-F(2)	1.581(2)
C(4)-H(4)	0.9500	P(1)-F(4A)	1.581(11)
C(5)-H(5)	0.9500	P(1)-F(4)	1.582(5)
C(6)-C(10)	1.409(4)	P(1)-F(1)	1.594(2)
C(6)-C(7)	1.414(4)	P(1)-F(5A)	1.596(12)
C(6)-H(6)	0.9500	P(2)-F(9A)	1.480(14)
C(7)-C(8)	1.416(5)	P(2)-F(12A)	1.504(19)

P(2)-F(7A)	1.515(11)	P(2)-F(8)	1.576(3)
P(2)-F(11A)	1.545(14)	P(2)-F(9)	1.582(3)
P(2)-F(10A)	1.545(15)	P(2)-F(12)	1.583(5)
P(2)-F(10)	1.565(3)	P(2)-F(11)	1.584(4)
P(2)-F(7)	1.569(3)	P(2)-F(8A)	1.603(11)

C(1)-Co(1)-C(2)	40.5(2)	C(21)-Co(2)-C(22)	40.5(2)
C(1)-Co(1)-C(5)	40.8(2)	C(21)-Co(2)-C(23)	67.2(2)
C(2)-Co(1)-C(5)	67.59(18)	C(22)-Co(2)-C(23)	39.6(2)
C(1)-Co(1)-C(3)	67.69(19)	C(21)-Co(2)-C(20)	40.7(2)
C(2)-Co(1)-C(3)	39.85(19)	C(22)-Co(2)-C(20)	67.86(18)
C(5)-Co(1)-C(3)	66.87(16)	C(23)-Co(2)-C(20)	66.97(16)
C(1)-Co(1)-C(8)	140.6(2)	C(21)-Co(2)-C(19)	67.51(18)
C(2)-Co(1)-C(8)	111.80(17)	C(22)-Co(2)-C(19)	67.14(18)
C(5)-Co(1)-C(8)	176.88(18)	C(23)-Co(2)-C(19)	39.75(18)
C(3)-Co(1)-C(8)	110.60(13)	C(20)-Co(2)-C(19)	39.80(17)
C(1)-Co(1)-C(7)	110.85(18)	C(21)-Co(2)-C(16)	131.9(2)
C(2)-Co(1)-C(7)	108.49(15)	C(22)-Co(2)-C(16)	171.5(2)
C(5)-Co(1)-C(7)	142.21(19)	C(23)-Co(2)-C(16)	147.2(2)
C(3)-Co(1)-C(7)	135.14(15)	C(20)-Co(2)-C(16)	108.56(15)
C(8)-Co(1)-C(7)	40.87(13)	C(19)-Co(2)-C(16)	115.50(14)
C(1)-Co(1)-C(4)	67.33(18)	C(21)-Co(2)-C(17)	108.94(17)
C(2)-Co(1)-C(4)	66.67(16)	C(22)-Co(2)-C(17)	132.8(2)
C(5)-Co(1)-C(4)	39.33(18)	C(23)-Co(2)-C(17)	171.70(19)
C(3)-Co(1)-C(4)	39.53(17)	C(20)-Co(2)-C(17)	115.33(15)
C(8)-Co(1)-C(4)	137.55(16)	C(19)-Co(2)-C(17)	146.79(15)
C(7)-Co(1)-C(4)	174.58(15)	C(16)-Co(2)-C(17)	40.79(12)
C(1)-Co(1)-C(6)	108.96(17)	C(21)-Co(2)-C(15)	171.0(2)
C(2)-Co(1)-C(6)	134.61(19)	C(22)-Co(2)-C(15)	147.2(2)
C(5)-Co(1)-C(6)	114.01(15)	C(23)-Co(2)-C(15)	116.17(18)
C(3)-Co(1)-C(6)	174.22(17)	C(20)-Co(2)-C(15)	131.52(17)
C(8)-Co(1)-C(6)	68.71(12)	C(19)-Co(2)-C(15)	109.23(14)
C(7)-Co(1)-C(6)	40.73(12)	C(16)-Co(2)-C(15)	40.93(12)
C(4)-Co(1)-C(6)	144.47(16)	C(17)-Co(2)-C(15)	68.85(12)
C(1)-Co(1)-C(9)	177.0(2)	C(21)-Co(2)-C(14)	147.5(2)
C(2)-Co(1)-C(9)	142.5(2)	C(22)-Co(2)-C(14)	116.53(18)
C(5)-Co(1)-C(9)	137.88(19)	C(23)-Co(2)-C(14)	110.93(14)
C(3)-Co(1)-C(9)	114.85(15)	C(20)-Co(2)-C(14)	171.08(17)
C(8)-Co(1)-C(9)	40.94(12)	C(19)-Co(2)-C(14)	133.15(14)
C(7)-Co(1)-C(9)	68.75(13)	C(16)-Co(2)-C(14)	68.18(11)
C(4)-Co(1)-C(9)	113.36(14)	C(17)-Co(2)-C(14)	68.08(11)
C(6)-Co(1)-C(9)	68.66(12)	C(15)-Co(2)-C(14)	40.70(11)
C(1)-Co(1)-C(10)	136.1(2)	C(21)-Co(2)-C(18)	115.41(17)
C(2)-Co(1)-C(10)	174.9(2)	C(22)-Co(2)-C(18)	109.74(16)
C(5)-Co(1)-C(10)	112.34(15)	C(23)-Co(2)-C(18)	133.23(16)
C(3)-Co(1)-C(10)	145.18(17)	C(20)-Co(2)-C(18)	147.09(17)
C(8)-Co(1)-C(10)	68.56(11)	C(19)-Co(2)-C(18)	171.92(15)
C(7)-Co(1)-C(10)	68.24(12)	C(16)-Co(2)-C(18)	68.74(12)
C(4)-Co(1)-C(10)	116.76(14)	C(17)-Co(2)-C(18)	40.74(12)
C(6)-Co(1)-C(10)	40.40(11)	C(15)-Co(2)-C(18)	69.03(11)
C(9)-Co(1)-C(10)	40.84(11)	C(14)-Co(2)-C(18)	40.60(11)

C(12)-N(1)-N(2)	109.9(2)	C(8)-C(9)-C(10)	107.4(3)
C(12)-N(1)-C(14)	130.6(2)	C(8)-C(9)-Co(1)	69.13(16)
N(2)-N(1)-C(14)	119.5(2)	C(10)-C(9)-Co(1)	69.84(15)
N(3)-N(2)-N(1)	107.6(2)	C(8)-C(9)-H(9)	126.3
N(2)-N(3)-C(11)	108.5(2)	C(10)-C(9)-H(9)	126.3
C(2)-C(1)-C(5)	106.8(4)	Co(1)-C(9)-H(9)	126.3
C(2)-C(1)-Co(1)	69.9(2)	C(6)-C(10)-C(9)	108.4(3)
C(5)-C(1)-Co(1)	70.5(2)	C(6)-C(10)-C(11)	124.8(3)
C(2)-C(1)-H(1)	126.6	C(9)-C(10)-C(11)	126.8(3)
C(5)-C(1)-H(1)	126.6	C(6)-C(10)-Co(1)	69.53(15)
Co(1)-C(1)-H(1)	124.6	C(9)-C(10)-Co(1)	69.31(15)
C(3)-C(2)-C(1)	108.7(4)	C(11)-C(10)-Co(1)	126.45(18)
C(3)-C(2)-Co(1)	71.1(2)	C(12)-C(11)-N(3)	109.5(2)
C(1)-C(2)-Co(1)	69.6(2)	C(12)-C(11)-C(10)	130.6(2)
C(3)-C(2)-H(2)	125.6	N(3)-C(11)-C(10)	119.9(2)
C(1)-C(2)-H(2)	125.6	C(11)-C(12)-N(1)	104.4(2)
Co(1)-C(2)-H(2)	125.2	C(11)-C(12)-H(12)	127.8
C(2)-C(3)-C(4)	107.8(4)	N(1)-C(12)-H(12)	127.8
C(2)-C(3)-Co(1)	69.0(2)	C(18)-C(14)-C(15)	109.2(3)
C(4)-C(3)-Co(1)	70.8(2)	C(18)-C(14)-N(1)	124.1(2)
C(2)-C(3)-H(3)	126.1	C(15)-C(14)-N(1)	126.7(2)
C(4)-C(3)-H(3)	126.1	C(18)-C(14)-Co(2)	69.72(15)
Co(1)-C(3)-H(3)	125.7	C(15)-C(14)-Co(2)	69.42(15)
C(5)-C(4)-C(3)	108.8(4)	N(1)-C(14)-Co(2)	127.12(18)
C(5)-C(4)-Co(1)	69.5(2)	C(14)-C(15)-C(16)	106.9(3)
C(3)-C(4)-Co(1)	69.66(19)	C(14)-C(15)-Co(2)	69.88(15)
C(5)-C(4)-H(4)	125.6	C(16)-C(15)-Co(2)	69.20(16)
C(3)-C(4)-H(4)	125.6	C(14)-C(15)-H(15)	126.5
Co(1)-C(4)-H(4)	126.8	C(16)-C(15)-H(15)	126.5
C(4)-C(5)-C(1)	107.9(4)	Co(2)-C(15)-H(15)	126.0
C(4)-C(5)-Co(1)	71.2(2)	C(17)-C(16)-C(15)	108.5(3)
C(1)-C(5)-Co(1)	68.7(2)	C(17)-C(16)-Co(2)	69.95(16)
C(4)-C(5)-H(5)	126.0	C(15)-C(16)-Co(2)	69.88(16)
C(1)-C(5)-H(5)	126.0	C(17)-C(16)-H(16)	125.7
Co(1)-C(5)-H(5)	125.7	C(15)-C(16)-H(16)	125.7
C(10)-C(6)-C(7)	108.0(3)	Co(2)-C(16)-H(16)	126.0
C(10)-C(6)-Co(1)	70.08(15)	C(16)-C(17)-C(18)	108.2(3)
C(7)-C(6)-Co(1)	69.29(16)	C(16)-C(17)-Co(2)	69.27(16)
C(10)-C(6)-H(6)	126.0	C(18)-C(17)-Co(2)	69.88(16)
C(7)-C(6)-H(6)	126.0	C(16)-C(17)-H(17)	125.9
Co(1)-C(6)-H(6)	126.2	C(18)-C(17)-H(17)	125.9
C(6)-C(7)-C(8)	108.2(3)	Co(2)-C(17)-H(17)	126.5
C(6)-C(7)-Co(1)	69.99(16)	C(14)-C(18)-C(17)	107.1(3)
C(8)-C(7)-Co(1)	69.52(16)	C(14)-C(18)-Co(2)	69.67(15)
C(6)-C(7)-H(7)	125.9	C(17)-C(18)-Co(2)	69.37(16)
C(8)-C(7)-H(7)	125.9	C(14)-C(18)-H(18)	126.4
Co(1)-C(7)-H(7)	126.2	C(17)-C(18)-H(18)	126.4
C(7)-C(8)-C(9)	108.0(3)	Co(2)-C(18)-H(18)	126.1
C(7)-C(8)-Co(1)	69.61(16)	C(23)-C(19)-C(20)	108.0(4)
C(9)-C(8)-Co(1)	69.93(16)	C(23)-C(19)-Co(2)	69.5(2)
C(7)-C(8)-H(8)	126.0	C(20)-C(19)-Co(2)	70.0(2)
C(9)-C(8)-H(8)	126.0	C(23)-C(19)-H(19)	126.0
Co(1)-C(8)-H(8)	126.0	C(20)-C(19)-H(19)	126.0

Co(2)-C(19)-H(19)	126.1	F(3A)-P(1)-F(1)	88.6(6)
C(19)-C(20)-C(21)	107.2(4)	F(3)-P(1)-F(1)	94.7(2)
C(19)-C(20)-Co(2)	70.2(2)	F(6)-P(1)-F(1)	87.2(2)
C(21)-C(20)-Co(2)	68.7(2)	F(5)-P(1)-F(1)	96.1(2)
C(19)-C(20)-H(20)	126.4	F(2)-P(1)-F(1)	178.11(15)
C(21)-C(20)-H(20)	126.4	F(4A)-P(1)-F(1)	81.4(5)
Co(2)-C(20)-H(20)	126.3	F(4)-P(1)-F(1)	88.6(3)
C(22)-C(21)-C(20)	107.7(4)	F(6A)-P(1)-F(5A)	167.2(11)
C(22)-C(21)-Co(2)	70.0(2)	F(3A)-P(1)-F(5A)	91.0(13)
C(20)-C(21)-Co(2)	70.6(2)	F(2)-P(1)-F(5A)	106.0(5)
C(22)-C(21)-H(21)	126.1	F(4A)-P(1)-F(5A)	85.8(11)
C(20)-C(21)-H(21)	126.1	F(1)-P(1)-F(5A)	75.8(5)
Co(2)-C(21)-H(21)	124.8	F(9A)-P(2)-F(12A)	93.7(17)
C(23)-C(22)-C(21)	107.7(4)	F(9A)-P(2)-F(7A)	94.8(16)
C(23)-C(22)-Co(2)	70.4(2)	F(12A)-P(2)-F(7A)	86.4(13)
C(21)-C(22)-Co(2)	69.5(3)	F(9A)-P(2)-F(11A)	86.9(14)
C(23)-C(22)-H(22)	126.1	F(12A)-P(2)-F(11A)	179.3(12)
C(21)-C(22)-H(22)	126.1	F(7A)-P(2)-F(11A)	93.1(12)
Co(2)-C(22)-H(22)	125.6	F(9A)-P(2)-F(10A)	178.4(19)
C(22)-C(23)-C(19)	109.4(4)	F(12A)-P(2)-F(10A)	87.9(12)
C(22)-C(23)-Co(2)	70.0(2)	F(7A)-P(2)-F(10A)	85.1(12)
C(19)-C(23)-Co(2)	70.8(2)	F(11A)-P(2)-F(10A)	91.6(14)
C(22)-C(23)-H(23)	125.3	F(10)-P(2)-F(7)	90.1(3)
C(19)-C(23)-H(23)	125.3	F(10)-P(2)-F(8)	91.2(3)
Co(2)-C(23)-H(23)	125.4	F(7)-P(2)-F(8)	178.4(3)
F(6A)-P(1)-F(3A)	96.5(14)	F(10)-P(2)-F(9)	179.5(2)
F(3)-P(1)-F(6)	90.1(3)	F(7)-P(2)-F(9)	90.0(3)
F(3)-P(1)-F(5)	91.2(3)	F(8)-P(2)-F(9)	88.7(2)
F(6)-P(1)-F(5)	176.4(3)	F(10)-P(2)-F(12)	91.1(2)
F(6A)-P(1)-F(2)	84.2(8)	F(7)-P(2)-F(12)	91.7(3)
F(3A)-P(1)-F(2)	90.9(6)	F(8)-P(2)-F(12)	89.2(2)
F(3)-P(1)-F(2)	86.6(2)	F(9)-P(2)-F(12)	89.3(3)
F(6)-P(1)-F(2)	91.5(3)	F(10)-P(2)-F(11)	91.1(2)
F(5)-P(1)-F(2)	85.3(2)	F(7)-P(2)-F(11)	89.4(3)
F(6A)-P(1)-F(4A)	85.0(11)	F(8)-P(2)-F(11)	89.6(3)
F(3A)-P(1)-F(4A)	169.9(7)	F(9)-P(2)-F(11)	88.4(2)
F(2)-P(1)-F(4A)	99.2(5)	F(12)-P(2)-F(11)	177.5(3)
F(3)-P(1)-F(4)	176.7(3)	F(9A)-P(2)-F(8A)	89.6(15)
F(6)-P(1)-F(4)	89.3(3)	F(12A)-P(2)-F(8A)	91.6(11)
F(5)-P(1)-F(4)	89.2(3)	F(7A)-P(2)-F(8A)	175.3(10)
F(2)-P(1)-F(4)	90.1(3)	F(11A)-P(2)-F(8A)	88.8(9)
F(6A)-P(1)-F(1)	94.0(8)	F(10A)-P(2)-F(8A)	90.6(13)

Table S16. Anisotropic displacement parameters (Å^{2}x 10^3) for **7**. The anisotropic displacement factor exponent takes the form: -2p^2 [h^2 a*^{2}U^{11} + ... + 2 h k a* b* U^{12}].

	U^{11}	U^{22}	U^{33}	U^{23}	U^{13}	U^{12}
Co(1)	34(1)	22(1)	20(1)	3(1)	7(1)	3(1)
Co(2)	30(1)	31(1)	19(1)	2(1)	5(1)	0(1)
N(1)	38(1)	34(1)	26(1)	-1(1)	4(1)	2(1)
N(2)	38(1)	41(1)	32(1)	-13(1)	1(1)	3(1)
N(3)	35(1)	38(1)	36(1)	-11(1)	1(1)	4(1)
C(1)	137(5)	104(4)	22(2)	15(2)	-11(2)	-68(4)
C(2)	45(2)	126(4)	71(3)	72(3)	20(2)	27(3)
C(3)	115(4)	28(2)	53(2)	17(2)	44(2)	9(2)
C(4)	44(2)	87(3)	46(2)	27(2)	16(2)	-10(2)
C(5)	131(4)	61(3)	42(2)	16(2)	52(3)	35(3)
C(6)	38(2)	26(1)	38(2)	8(1)	8(1)	0(1)
C(7)	36(2)	42(2)	40(2)	15(1)	16(1)	2(1)
C(8)	53(2)	41(2)	28(1)	7(1)	20(1)	14(1)
C(9)	44(2)	34(2)	21(1)	1(1)	5(1)	4(1)
C(10)	39(2)	26(1)	23(1)	7(1)	7(1)	4(1)
C(11)	29(1)	20(1)	18(1)	1(1)	2(1)	3(1)
C(12)	38(2)	26(1)	26(1)	-3(1)	3(1)	4(1)
C(14)	34(2)	31(1)	20(1)	4(1)	6(1)	1(1)
C(15)	40(2)	29(1)	29(1)	0(1)	8(1)	-4(1)
C(16)	30(2)	42(2)	34(2)	3(1)	8(1)	-8(1)
C(17)	35(2)	46(2)	28(1)	2(1)	12(1)	3(1)
C(18)	40(2)	39(2)	20(1)	-3(1)	6(1)	4(1)
C(19)	82(3)	66(2)	23(2)	3(2)	18(2)	-8(2)
C(20)	40(2)	110(4)	46(2)	40(2)	2(2)	7(2)
C(21)	178(6)	47(2)	62(3)	31(2)	54(4)	44(3)
C(22)	119(4)	89(4)	46(2)	23(2)	-5(3)	-68(3)
C(23)	45(2)	127(4)	52(2)	46(3)	23(2)	8(2)
P(1)	32(1)	33(1)	36(1)	6(1)	9(1)	1(1)
P(2)	37(1)	31(1)	47(1)	-4(1)	13(1)	-1(1)
F(1)	53(1)	58(1)	79(2)	-14(1)	2(1)	-20(1)
F(2)	56(1)	45(1)	129(2)	8(1)	16(1)	-17(1)
F(3)	38(2)	89(3)	98(4)	62(3)	0(2)	13(2)
F(4)	49(2)	39(2)	102(6)	28(2)	-9(3)	5(2)
F(5)	87(3)	91(4)	69(3)	-20(2)	54(2)	-11(3)
F(6)	102(5)	52(3)	64(2)	-9(2)	56(3)	-5(2)
F(7)	155(5)	62(2)	88(3)	-28(2)	8(3)	-54(3)
F(8)	71(2)	34(2)	95(3)	-10(2)	4(2)	-9(2)
F(9)	105(3)	90(3)	85(3)	8(2)	67(3)	12(3)
F(10)	70(2)	91(3)	64(2)	13(2)	35(2)	3(2)
F(11)	37(2)	107(4)	86(3)	41(3)	3(2)	9(2)
F(12)	48(2)	55(3)	107(4)	27(3)	6(2)	12(2)
F(3A)	69(9)	95(12)	450(40)	-150(18)	131(18)	-31(9)
F(4A)	42(5)	98(10)	220(20)	-71(10)	51(9)	3(6)
F(5A)	165(16)	178(16)	77(9)	96(10)	-40(9)	-87(13)
F(6A)	147(19)	210(20)	39(6)	46(10)	-54(9)	-99(16)
F(7A)	71(9)	150(17)	200(20)	121(16)	-30(12)	-66(11)

F(8A)	50(7)	81(10)	210(20)	61(12)	27(9)	-10(6)
F(9A)	500(50)	96(13)	70(10)	-29(9)	-99(18)	-10(20)
F(10A)	310(30)	127(15)	70(9)	-17(9)	-24(14)	-13(18)
F(11A)	149(16)	63(8)	210(20)	34(13)	109(17)	59(9)
F(12A)	131(18)	67(10)	220(30)	7(15)	55(18)	52(11)

Table S17. Hydrogen coordinates (x 10^4) and isotropic displacement parameters ($Å^2$x 10^3) for **7**.

	x	y	z	U(eq)
H(1)	11734	3165	6668	110
H(2)	12353	1930	5968	95
H(3)	10356	1226	5203	73
H(4)	8490	1998	5454	70
H(5)	9309	3195	6342	88
H(6)	10833	4308	4735	41
H(7)	12547	3379	4448	46
H(8)	11441	2248	3470	47
H(9)	9026	2480	3141	40
H(12)	6828	3361	2904	37
H(15)	4220	3451	2484	39
H(16)	2011	4051	2371	42
H(17)	2288	5276	3371	43
H(18)	4674	5452	4103	39
H(19)	3770	4485	432	68
H(20)	2091	5414	718	80
H(21)	3232	6465	1819	110
H(22)	5596	6176	2165	106
H(23)	5900	4962	1321	87

Table S18. Torsion angles [°] for **7**.

C(12)-N(1)-N(2)-N(3)	-0.1(3)	N(3)-C(11)-C(12)-N(1)	0.2(3)
C(14)-N(1)-N(2)-N(3)	178.0(2)	C(10)-C(11)-C(12)-N(1)	-178.9(3)
N(1)-N(2)-N(3)-C(11)	0.2(3)	N(2)-N(1)-C(12)-C(11)	0.0(3)
C(5)-C(1)-C(2)-C(3)	0.6(4)	C(14)-N(1)-C(12)-C(11)	-177.9(3)
Co(1)-C(1)-C(2)-C(3)	-60.6(3)	C(12)-N(1)-C(14)-C(18)	-172.3(3)
C(5)-C(1)-C(2)-Co(1)	61.2(3)	N(2)-N(1)-C(14)-C(18)	10.0(4)
C(1)-C(2)-C(3)-C(4)	-0.8(4)	C(12)-N(1)-C(14)-C(15)	7.8(5)
Co(1)-C(2)-C(3)-C(4)	-60.4(2)	N(2)-N(1)-C(14)-C(15)	-169.9(3)
C(1)-C(2)-C(3)-Co(1)	59.6(3)	C(12)-N(1)-C(14)-Co(2)	-83.1(4)
C(2)-C(3)-C(4)-C(5)	0.7(4)	N(2)-N(1)-C(14)-Co(2)	99.3(3)
Co(1)-C(3)-C(4)-C(5)	-58.6(3)	C(18)-C(14)-C(15)-C(16)	-1.1(3)
C(2)-C(3)-C(4)-Co(1)	59.3(2)	N(1)-C(14)-C(15)-C(16)	178.8(2)
C(3)-C(4)-C(5)-C(1)	-0.3(4)	Co(2)-C(14)-C(15)-C(16)	-59.55(19)
Co(1)-C(4)-C(5)-C(1)	-59.1(3)	C(18)-C(14)-C(15)-Co(2)	58.49(19)
C(3)-C(4)-C(5)-Co(1)	58.7(2)	N(1)-C(14)-C(15)-Co(2)	-121.6(3)
C(2)-C(1)-C(5)-C(4)	-0.1(4)	C(14)-C(15)-C(16)-C(17)	0.5(3)
Co(1)-C(1)-C(5)-C(4)	60.7(3)	Co(2)-C(15)-C(16)-C(17)	-59.5(2)
C(2)-C(1)-C(5)-Co(1)	-60.8(3)	C(14)-C(15)-C(16)-Co(2)	59.99(18)
C(10)-C(6)-C(7)-C(8)	-0.4(3)	C(15)-C(16)-C(17)-C(18)	0.2(3)
Co(1)-C(6)-C(7)-C(8)	59.19(19)	Co(2)-C(16)-C(17)-C(18)	-59.2(2)
C(10)-C(6)-C(7)-Co(1)	-59.61(19)	C(15)-C(16)-C(17)-Co(2)	59.44(19)
C(6)-C(7)-C(8)-C(9)	0.1(3)	C(15)-C(14)-C(18)-C(17)	1.2(3)
Co(1)-C(7)-C(8)-C(9)	59.62(19)	N(1)-C(14)-C(18)-C(17)	-178.7(2)
C(6)-C(7)-C(8)-Co(1)	-59.48(19)	Co(2)-C(14)-C(18)-C(17)	59.51(19)
C(7)-C(8)-C(9)-C(10)	0.2(3)	C(15)-C(14)-C(18)-Co(2)	-58.31(19)
Co(1)-C(8)-C(9)-C(10)	59.61(18)	N(1)-C(14)-C(18)-Co(2)	121.8(3)
C(7)-C(8)-C(9)-Co(1)	-59.42(19)	C(16)-C(17)-C(18)-C(14)	-0.9(3)
C(7)-C(6)-C(10)-C(9)	0.5(3)	Co(2)-C(17)-C(18)-C(14)	-59.70(18)
Co(1)-C(6)-C(10)-C(9)	-58.57(18)	C(16)-C(17)-C(18)-Co(2)	58.8(2)
C(7)-C(6)-C(10)-C(11)	180.0(2)	C(23)-C(19)-C(20)-C(21)	-0.4(4)
Co(1)-C(6)-C(10)-C(11)	120.9(3)	Co(2)-C(19)-C(20)-C(21)	58.9(3)
C(7)-C(6)-C(10)-Co(1)	59.12(19)	C(23)-C(19)-C(20)-Co(2)	-59.3(2)
C(8)-C(9)-C(10)-C(6)	-0.5(3)	C(19)-C(20)-C(21)-C(22)	0.6(4)
Co(1)-C(9)-C(10)-C(6)	58.70(18)	Co(2)-C(20)-C(21)-C(22)	60.5(3)
C(8)-C(9)-C(10)-C(11)	-179.9(2)	C(19)-C(20)-C(21)-Co(2)	-59.9(3)
Co(1)-C(9)-C(10)-C(11)	-120.7(3)	C(20)-C(21)-C(22)-C(23)	-0.6(5)
C(8)-C(9)-C(10)-Co(1)	-59.16(19)	Co(2)-C(21)-C(22)-C(23)	60.3(3)
N(2)-N(3)-C(11)-C(12)	-0.3(3)	C(20)-C(21)-C(22)-Co(2)	-60.8(3)
N(2)-N(3)-C(11)-C(10)	178.9(2)	C(21)-C(22)-C(23)-C(19)	0.4(5)
C(6)-C(10)-C(11)-C(12)	166.0(3)	Co(2)-C(22)-C(23)-C(19)	60.1(3)
C(9)-C(10)-C(11)-C(12)	-14.7(5)	C(21)-C(22)-C(23)-Co(2)	-59.7(3)
Co(1)-C(10)-C(11)-C(12)	-105.0(3)	C(20)-C(19)-C(23)-C(22)	0.0(4)
C(6)-C(10)-C(11)-N(3)	-13.0(4)	Co(2)-C(19)-C(23)-C(22)	-59.6(3)
C(9)-C(10)-C(11)-N(3)	166.3(3)	C(20)-C(19)-C(23)-Co(2)	59.6(3)
Co(1)-C(10)-C(11)-N(3)	76.1(3)		

Triazolium salt (8a) – "4-cobaltoceniumyl-1-ferrocenyl-3-methyltriazolium hexafluoridophosphate triflate"

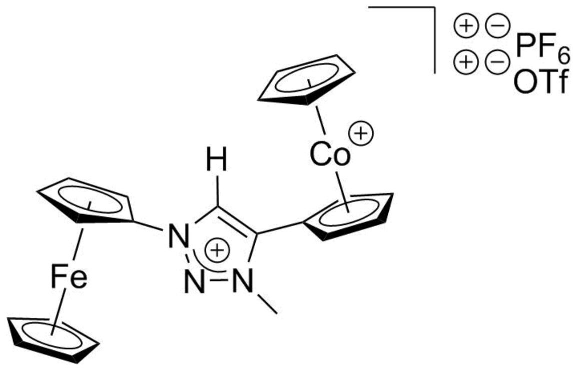

[749.25]

$C_{23}H_{22}N_3CoFe(PF_6)(CF_3SO_3)$

Analytical data:

IR (ATR [cm^{-1}]): 3116 (ν_{C-H}), 1604, 1440, 1419 ($\nu_{C=C}$), 1255 (ν_{SO3}), 1226 (ν_{CF3}), 1154 (ν_{CF3}), 1028 (ν_{SO3}), 835 (ν_{P-F}), 636, 558 (ν_{P-F}), 508, 446.

^{1}H-NMR (300 MHz, C_3D_6O, [ppm]): δ 4.43 (s, 5H, Fc-Cp), 4.61 (m, 5H, CH_3 of triazolium and C3/C4 of substituted Fc-Cp), 5.33 (pseudo-t, 2H, *J* = 2.0 Hz, C2/C5 of substituted Fc-Cp), 6.15 (s, 5H, Cc-Cp), 6.31 (pseudo-t, 2H, *J* = 2.1 Hz, C3/C4 of substituted Cc-Cp), 6.77 (pseudo-t, 2H, J = 2.1 Hz, C2/C5 of substituted Cc-Cp), 9.90 (s, 1H, CH of triazolium).

^{13}C-NMR (75 MHz, C_3D_6O, [ppm]): δ 40.8 (CH_3 of triazolium), 64.0 (C3/C4 of substituted Fc-Cp), 69.7 (C2/C5 of substituted Fc-Cp), 72.2 (Fc-Cp), 85.9 (C3/C4 of substituted Cc-Cp), 86.6 (quart. carbon of substituted Fc-Cp), 87.3 (C2/C5 of substituted Cc-Cp), 88.3 (Cc-Cp), 92.7 (quart. carbon of substituted Cc-Cp), 130.5 (CH of triazolium), 137.6 (quart. carbon of triazolium).

MS (ESI pos, [m/z]): 604.01 ($[M(CF_3SO_3)]^+$).

Melting point [°C]: 232.

Spectra

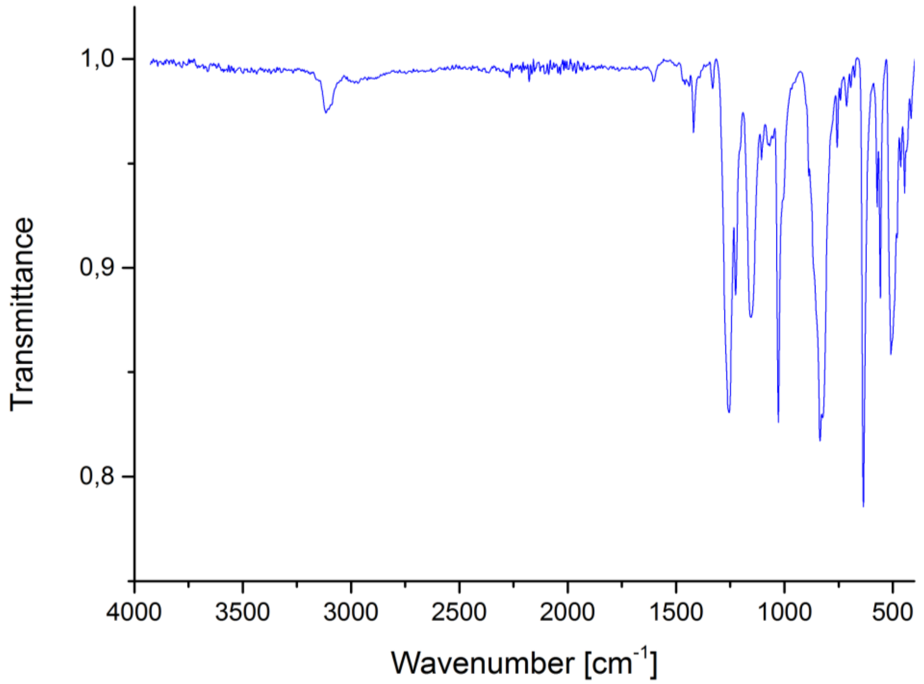

Figure S16. IR-spectrum (ATR, [cm^{-1}]) of "4-cobaltoceniumyl-1-ferrocenyl-3-methyltriazolium hexafluoridophosphate triflate".

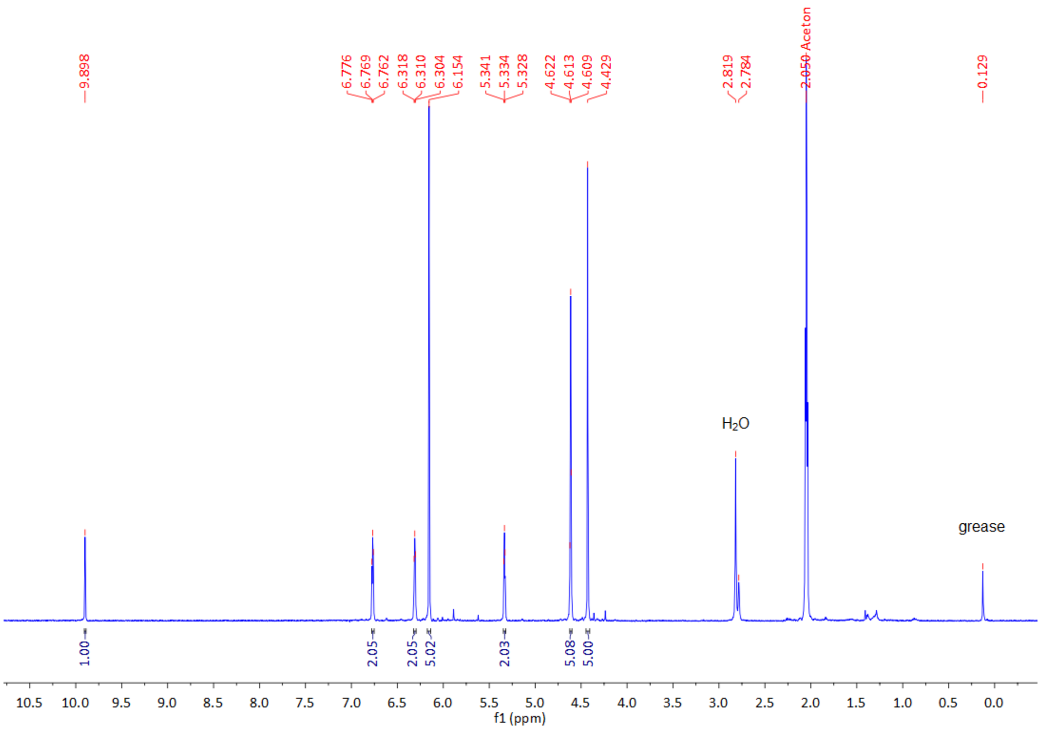

Figure S17. ^{1}H-NMR (300 MHz, C_3D_6O, [ppm]) of “4-cobaltoceniumyl-1-ferrocenyl-3-methyltriazolium hexafluoridophosphate triflate“.

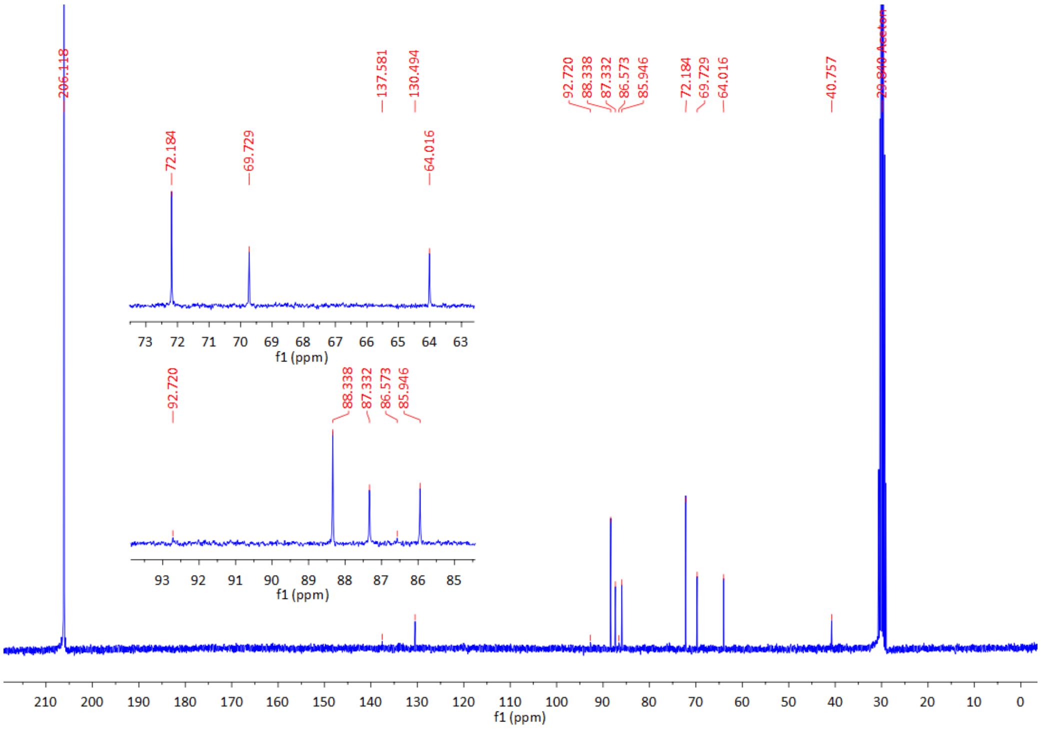

Figure S18. ^{13}C-NMR (75 MHz, C_3D_6O, [ppm]) of “4-cobaltoceniumyl-1-ferrocenyl-3-methyltriazolium hexafluoridophosphate triflate“.

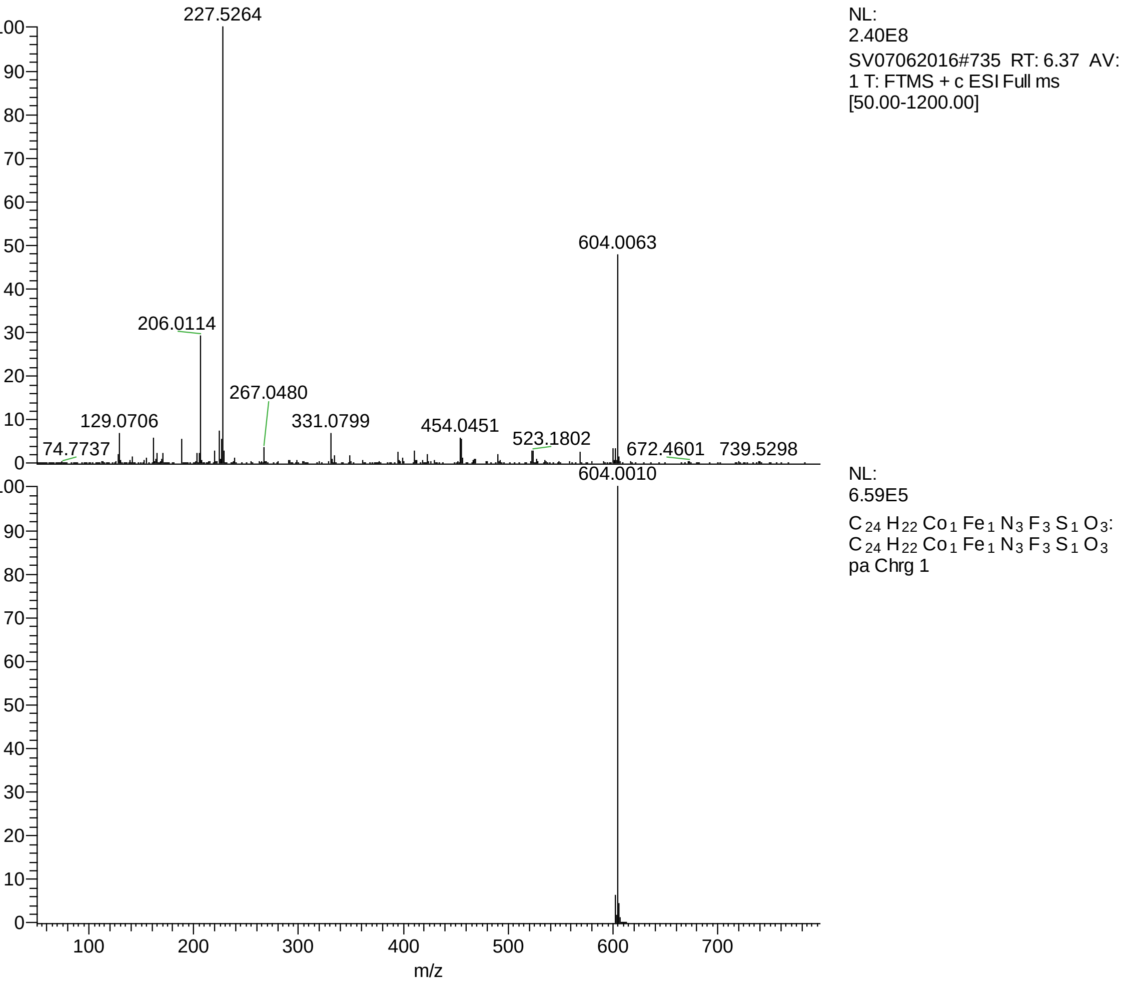

Figure S19. MS (ESI pos, [m/z]; *top*: experimental, *bottom*: simulated) of "4-cobaltoceniumyl-1-ferrocenyl-3-methyltriazolium hexafluoridophosphate triflate".

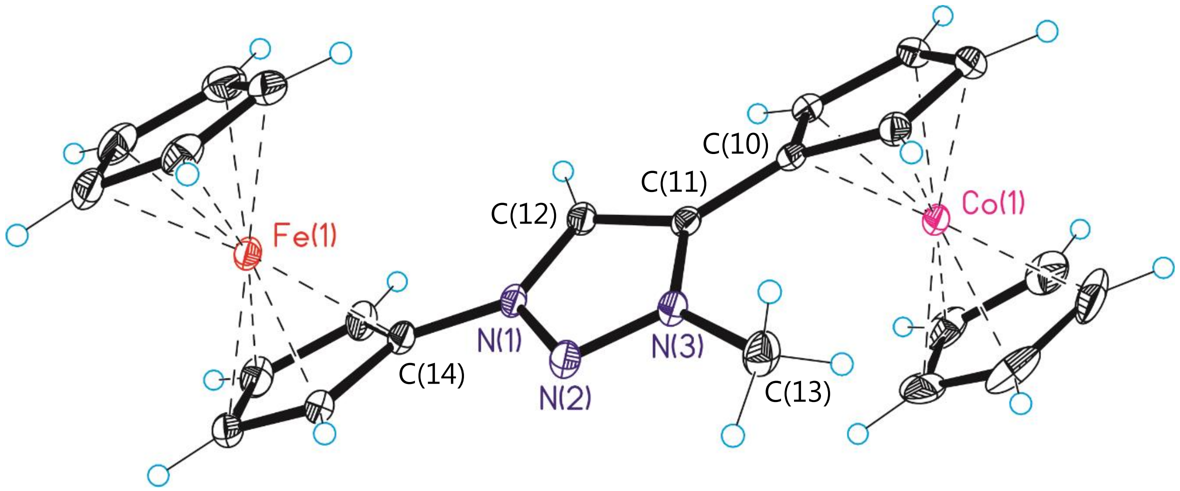

Figure S20. X-ray single crystal structure analysis of "4-cobaltoceniumyl-1-ferrocenyl-3-methyltriazolium hexafluoridophosphate triflate" (counterions omitted for clarity).

X-Ray single crystal structure analysis data

Table S19. Crystal data and structure refinement for **8a**.

Empirical formula	C25 H22 Co F6 Fe N3 O6 S2	
Formula weight	753.35	
Temperature	173(2) K	
Wavelength	0.71073 Å	
Crystal system	Triclinic	
Space group	P-1 (no. 2)	
Unit cell dimensions	a = 9.5145(4) Å	α = 103.310(1)°.
	b = 10.5985(5) Å	ß = 108.231(1)°.
	c = 14.8777(7) Å	γ = 98.602(1)°.
Volume	1346.23(11) $Å^3$	
Z	2	
Density (calculated)	1.858 Mg/m^3	
Absorption coefficient	1.401 mm^{-1}	
F(000)	760	
Crystal size	0.160 x 0.110 x 0.070 mm^3	
Theta range for data collection	2.145 to 25.999°.	
Index ranges	-11<h<11, -13<k<13, -18<l<18	
Reflections collected	54524	
Independent reflections	5287 [R(int) = 0.0303]	
Completeness to theta = 25.242°	100.0 %	
Absorption correction	Semi-empirical from equivalents	
Max. and min. transmission	0.928 and 0.881	
Refinement method	Full-matrix least-squares on F^2	
Data / restraints / parameters	5287 / 0 / 398	
Goodness-of-fit on F^2	1.049	
Final R indices [I>2sigma(I)]	R1 = 0.0273, wR2 = 0.0657	
R indices (all data)	R1 = 0.0328, wR2 = 0.0683	
Extinction coefficient	n/a	
Largest diff. peak and hole	0.630 and -0.508 e.$Å^{-3}$	

Table S20. Atomic coordinates (x 10^4) and equivalent isotropic displacement parameters ($Å^2$x 10^3) for **8a**. U(eq) is defined as one third of the trace of the orthogonalized U^{ij} tensor.

	x	y	z	U(eq)
Co(1)	-729(1)	5878(1)	7379(1)	17(1)
Fe(1)	4378(1)	10238(1)	12987(1)	17(1)
N(1)	2769(2)	7936(2)	11007(1)	17(1)
N(2)	3287(2)	6878(2)	10722(1)	20(1)
N(3)	2789(2)	6585(2)	9748(1)	18(1)
C(1)	-2637(3)	5003(2)	7571(2)	35(1)
C(2)	-2878(3)	4746(2)	6557(2)	36(1)
C(3)	-1772(3)	4075(2)	6367(2)	41(1)
C(4)	-868(3)	3919(2)	7260(2)	40(1)
C(5)	-1391(3)	4491(2)	8001(2)	39(1)
C(6)	-129(2)	7890(2)	8059(1)	19(1)
C(7)	-605(2)	7618(2)	7015(2)	23(1)
C(8)	355(2)	6878(2)	6677(2)	23(1)
C(9)	1437(2)	6684(2)	7510(1)	20(1)
C(10)	1145(2)	7318(2)	8370(1)	17(1)
C(11)	1938(2)	7422(2)	9404(1)	16(1)
C(12)	1947(2)	8314(2)	10239(1)	18(1)
C(13)	3204(2)	5454(2)	9197(2)	25(1)
C(14)	3054(2)	8468(2)	12036(1)	19(1)
C(15)	4221(2)	8234(2)	12805(1)	21(1)
C(16)	4032(2)	8843(2)	13701(2)	24(1)
C(17)	2769(2)	9437(2)	13478(2)	27(1)
C(18)	2145(2)	9210(2)	12435(2)	23(1)
C(19)	5313(3)	11305(2)	12245(2)	29(1)
C(20)	6471(2)	11026(2)	12995(2)	27(1)
C(21)	6349(2)	11601(2)	13915(2)	27(1)
C(22)	5119(3)	12237(2)	13743(2)	30(1)
C(23)	4481(3)	12053(2)	12710(2)	31(1)
S(1)	2014(1)	12312(1)	9889(1)	25(1)
S(2)	-2494(1)	6386(1)	4042(1)	27(1)
C(24)	2663(3)	11537(2)	8920(2)	33(1)
C(25)	-1443(3)	8103(2)	4293(2)	31(1)
O(1)	908(2)	12960(2)	9411(1)	35(1)
O(2)	3386(2)	13191(2)	10624(1)	40(1)
O(3)	1388(2)	11185(2)	10147(1)	40(1)
O(4)	-1285(2)	5714(2)	4301(1)	37(1)
O(5)	-3326(2)	6541(2)	4700(1)	44(1)
O(6)	-3395(2)	5997(2)	3005(1)	46(1)
F(1)	3749(2)	10921(2)	9270(1)	63(1)
F(2)	1529(2)	10637(2)	8168(1)	57(1)
F(3)	3252(2)	12419(2)	8556(1)	45(1)
F(4)	-2361(2)	8925(2)	4169(1)	55(1)
F(5)	-659(2)	8198(2)	3710(2)	67(1)
F(6)	-465(2)	8594(2)	5221(1)	49(1)

Table S21. Bond lengths [Å] and angles [°] for **8a**.

Co(1)-C(3)	2.019(2)	C(8)-H(8)	0.9500
Co(1)-C(4)	2.023(2)	C(9)-C(10)	1.432(3)
Co(1)-C(5)	2.031(2)	C(9)-H(9)	0.9500
Co(1)-C(8)	2.033(2)	C(10)-C(11)	1.455(3)
Co(1)-C(2)	2.035(2)	C(11)-C(12)	1.374(3)
Co(1)-C(7)	2.035(2)	C(12)-H(12)	0.9500
Co(1)-C(9)	2.040(2)	C(13)-H(13A)	0.9800
Co(1)-C(6)	2.0438(19)	C(13)-H(13B)	0.9800
Co(1)-C(1)	2.045(2)	C(13)-H(13C)	0.9800
Co(1)-C(10)	2.0462(19)	C(14)-C(15)	1.422(3)
Fe(1)-C(14)	2.0159(19)	C(14)-C(18)	1.424(3)
Fe(1)-C(20)	2.037(2)	C(15)-C(16)	1.419(3)
Fe(1)-C(21)	2.040(2)	C(15)-H(15)	0.9500
Fe(1)-C(18)	2.044(2)	C(16)-C(17)	1.422(3)
Fe(1)-C(19)	2.045(2)	C(16)-H(16)	0.9500
Fe(1)-C(17)	2.048(2)	C(17)-C(18)	1.424(3)
Fe(1)-C(22)	2.052(2)	C(17)-H(17)	0.9500
Fe(1)-C(23)	2.054(2)	C(18)-H(18)	0.9500
Fe(1)-C(16)	2.054(2)	C(19)-C(23)	1.419(3)
Fe(1)-C(15)	2.054(2)	C(19)-C(20)	1.424(3)
N(1)-N(2)	1.326(2)	C(19)-H(19)	0.9500
N(1)-C(12)	1.350(2)	C(20)-C(21)	1.413(3)
N(1)-C(14)	1.425(2)	C(20)-H(20)	0.9500
N(2)-N(3)	1.320(2)	C(21)-C(22)	1.421(3)
N(3)-C(11)	1.367(2)	C(21)-H(21)	0.9500
N(3)-C(13)	1.469(2)	C(22)-C(23)	1.418(3)
C(1)-C(2)	1.407(4)	C(22)-H(22)	0.9500
C(1)-C(5)	1.409(4)	C(23)-H(23)	0.9500
C(1)-H(1)	0.9500	S(1)-O(2)	1.4294(18)
C(2)-C(3)	1.418(4)	S(1)-O(1)	1.4346(17)
C(2)-H(2)	0.9500	S(1)-O(3)	1.4382(17)
C(3)-C(4)	1.400(4)	S(1)-C(24)	1.815(2)
C(3)-H(3)	0.9500	S(2)-O(6)	1.4348(17)
C(4)-C(5)	1.399(4)	S(2)-O(5)	1.4357(18)
C(4)-H(4)	0.9500	S(2)-O(4)	1.4399(17)
C(5)-H(5)	0.9500	S(2)-C(25)	1.833(3)
C(6)-C(7)	1.419(3)	C(24)-F(3)	1.322(3)
C(6)-C(10)	1.431(3)	C(24)-F(2)	1.331(3)
C(6)-H(6)	0.9500	C(24)-F(1)	1.337(3)
C(7)-C(8)	1.422(3)	C(25)-F(5)	1.320(3)
C(7)-H(7)	0.9500	C(25)-F(4)	1.325(3)
C(8)-C(9)	1.421(3)	C(25)-F(6)	1.327(3)

C(3)-Co(1)-C(4)	40.53(12)	C(5)-Co(1)-C(8)	162.10(10)
C(3)-Co(1)-C(5)	68.09(11)	C(3)-Co(1)-C(2)	40.94(11)
C(4)-Co(1)-C(5)	40.38(11)	C(4)-Co(1)-C(2)	68.32(10)
C(3)-Co(1)-C(8)	103.46(10)	C(5)-Co(1)-C(2)	67.99(10)
C(4)-Co(1)-C(8)	123.19(10)	C(8)-Co(1)-C(2)	116.68(10)

C(3)-Co(1)-C(7)	122.87(10)
C(4)-Co(1)-C(7)	160.21(11)
C(5)-Co(1)-C(7)	156.89(10)
C(8)-Co(1)-C(7)	40.91(9)
C(2)-Co(1)-C(7)	105.94(9)
C(3)-Co(1)-C(9)	116.58(9)
C(4)-Co(1)-C(9)	106.52(9)
C(5)-Co(1)-C(9)	127.36(10)
C(8)-Co(1)-C(9)	40.83(8)
C(2)-Co(1)-C(9)	151.22(10)
C(7)-Co(1)-C(9)	68.87(8)
C(3)-Co(1)-C(6)	161.68(10)
C(4)-Co(1)-C(6)	157.43(10)
C(5)-Co(1)-C(6)	123.79(10)
C(8)-Co(1)-C(6)	68.84(8)
C(2)-Co(1)-C(6)	126.30(9)
C(7)-Co(1)-C(6)	40.72(8)
C(9)-Co(1)-C(6)	69.14(8)
C(3)-Co(1)-C(1)	68.33(10)
C(4)-Co(1)-C(1)	68.13(10)
C(5)-Co(1)-C(1)	40.46(11)
C(8)-Co(1)-C(1)	152.91(10)
C(2)-Co(1)-C(1)	40.35(10)
C(7)-Co(1)-C(1)	120.57(10)
C(9)-Co(1)-C(1)	166.11(10)
C(6)-Co(1)-C(1)	110.55(9)
C(3)-Co(1)-C(10)	153.42(10)
C(4)-Co(1)-C(10)	121.37(9)
C(5)-Co(1)-C(10)	111.42(9)
C(8)-Co(1)-C(10)	68.67(8)
C(2)-Co(1)-C(10)	165.44(9)
C(7)-Co(1)-C(10)	68.59(8)
C(9)-Co(1)-C(10)	41.01(8)
C(6)-Co(1)-C(10)	40.96(8)
C(1)-Co(1)-C(10)	129.76(9)
C(14)-Fe(1)-C(20)	122.61(8)
C(14)-Fe(1)-C(21)	157.06(9)
C(20)-Fe(1)-C(21)	40.57(9)
C(14)-Fe(1)-C(18)	41.06(8)
C(20)-Fe(1)-C(18)	158.74(9)
C(21)-Fe(1)-C(18)	159.59(9)
C(14)-Fe(1)-C(19)	109.38(8)
C(20)-Fe(1)-C(19)	40.83(9)
C(21)-Fe(1)-C(19)	68.34(9)
C(18)-Fe(1)-C(19)	123.04(9)
C(14)-Fe(1)-C(17)	67.98(8)
C(20)-Fe(1)-C(17)	158.97(9)
C(21)-Fe(1)-C(17)	122.75(9)
C(18)-Fe(1)-C(17)	40.74(8)
C(19)-Fe(1)-C(17)	158.50(10)
C(14)-Fe(1)-C(22)	161.62(9)
C(20)-Fe(1)-C(22)	68.34(9)
C(21)-Fe(1)-C(22)	40.62(9)

C(18)-Fe(1)-C(22)	123.73(9)
C(19)-Fe(1)-C(22)	68.20(9)
C(17)-Fe(1)-C(22)	107.19(9)
C(14)-Fe(1)-C(23)	125.95(9)
C(20)-Fe(1)-C(23)	68.34(9)
C(21)-Fe(1)-C(23)	68.14(9)
C(18)-Fe(1)-C(23)	108.42(9)
C(19)-Fe(1)-C(23)	40.50(9)
C(17)-Fe(1)-C(23)	122.47(10)
C(22)-Fe(1)-C(23)	40.41(10)
C(14)-Fe(1)-C(16)	67.79(8)
C(20)-Fe(1)-C(16)	122.67(9)
C(21)-Fe(1)-C(16)	106.15(9)
C(18)-Fe(1)-C(16)	68.75(9)
C(19)-Fe(1)-C(16)	160.07(9)
C(17)-Fe(1)-C(16)	40.57(9)
C(22)-Fe(1)-C(16)	120.91(9)
C(23)-Fe(1)-C(16)	157.09(9)
C(14)-Fe(1)-C(15)	40.89(8)
C(20)-Fe(1)-C(15)	106.60(9)
C(21)-Fe(1)-C(15)	120.15(9)
C(18)-Fe(1)-C(15)	69.59(8)
C(19)-Fe(1)-C(15)	124.42(9)
C(17)-Fe(1)-C(15)	68.56(9)
C(22)-Fe(1)-C(15)	155.76(9)
C(23)-Fe(1)-C(15)	161.82(9)
C(16)-Fe(1)-C(15)	40.41(8)
N(2)-N(1)-C(12)	112.81(16)
N(2)-N(1)-C(14)	118.21(16)
C(12)-N(1)-C(14)	128.86(16)
N(3)-N(2)-N(1)	104.16(15)
N(2)-N(3)-C(11)	112.58(15)
N(2)-N(3)-C(13)	117.81(16)
C(11)-N(3)-C(13)	129.61(17)
C(2)-C(1)-C(5)	107.7(2)
C(2)-C(1)-Co(1)	69.46(13)
C(5)-C(1)-Co(1)	69.26(13)
C(2)-C(1)-H(1)	126.2
C(5)-C(1)-H(1)	126.2
Co(1)-C(1)-H(1)	126.7
C(1)-C(2)-C(3)	107.8(2)
C(1)-C(2)-Co(1)	70.19(13)
C(3)-C(2)-Co(1)	68.92(13)
C(1)-C(2)-H(2)	126.1
C(3)-C(2)-H(2)	126.1
Co(1)-C(2)-H(2)	126.3
C(4)-C(3)-C(2)	107.9(2)
C(4)-C(3)-Co(1)	69.89(13)
C(2)-C(3)-Co(1)	70.13(13)
C(4)-C(3)-H(3)	126.0
C(2)-C(3)-H(3)	126.0
Co(1)-C(3)-H(3)	125.5
C(5)-C(4)-C(3)	108.2(2)

C(5)-C(4)-Co(1)	70.13(13)	C(15)-C(14)-C(18)	110.49(18)
C(3)-C(4)-Co(1)	69.58(14)	C(15)-C(14)-N(1)	124.20(18)
C(5)-C(4)-H(4)	125.9	C(18)-C(14)-N(1)	125.14(18)
C(3)-C(4)-H(4)	125.9	C(15)-C(14)-Fe(1)	71.00(11)
Co(1)-C(4)-H(4)	126.0	C(18)-C(14)-Fe(1)	70.52(11)
C(4)-C(5)-C(1)	108.4(2)	N(1)-C(14)-Fe(1)	129.14(14)
C(4)-C(5)-Co(1)	69.50(14)	C(16)-C(15)-C(14)	106.07(18)
C(1)-C(5)-Co(1)	70.27(13)	C(16)-C(15)-Fe(1)	69.79(12)
C(4)-C(5)-H(5)	125.8	C(14)-C(15)-Fe(1)	68.11(11)
C(1)-C(5)-H(5)	125.8	C(16)-C(15)-H(15)	127.0
Co(1)-C(5)-H(5)	126.0	C(14)-C(15)-H(15)	127.0
C(7)-C(6)-C(10)	107.58(18)	Fe(1)-C(15)-H(15)	126.7
C(7)-C(6)-Co(1)	69.31(11)	C(15)-C(16)-C(17)	108.85(18)
C(10)-C(6)-Co(1)	69.61(11)	C(15)-C(16)-Fe(1)	69.81(11)
C(7)-C(6)-H(6)	126.2	C(17)-C(16)-Fe(1)	69.49(12)
C(10)-C(6)-H(6)	126.2	C(15)-C(16)-H(16)	125.6
Co(1)-C(6)-H(6)	126.4	C(17)-C(16)-H(16)	125.6
C(6)-C(7)-C(8)	108.43(18)	Fe(1)-C(16)-H(16)	126.7
C(6)-C(7)-Co(1)	69.97(11)	C(16)-C(17)-C(18)	108.76(18)
C(8)-C(7)-Co(1)	69.47(11)	C(16)-C(17)-Fe(1)	69.95(12)
C(6)-C(7)-H(7)	125.8	C(18)-C(17)-Fe(1)	69.48(12)
C(8)-C(7)-H(7)	125.8	C(16)-C(17)-H(17)	125.6
Co(1)-C(7)-H(7)	126.4	C(18)-C(17)-H(17)	125.6
C(9)-C(8)-C(7)	108.33(18)	Fe(1)-C(17)-H(17)	126.5
C(9)-C(8)-Co(1)	69.85(11)	C(14)-C(18)-C(17)	105.82(18)
C(7)-C(8)-Co(1)	69.62(11)	C(14)-C(18)-Fe(1)	68.42(11)
C(9)-C(8)-H(8)	125.8	C(17)-C(18)-Fe(1)	69.78(12)
C(7)-C(8)-H(8)	125.8	C(14)-C(18)-H(18)	127.1
Co(1)-C(8)-H(8)	126.3	C(17)-C(18)-H(18)	127.1
C(8)-C(9)-C(10)	107.55(18)	Fe(1)-C(18)-H(18)	126.3
C(8)-C(9)-Co(1)	69.32(11)	C(23)-C(19)-C(20)	107.8(2)
C(10)-C(9)-Co(1)	69.72(11)	C(23)-C(19)-Fe(1)	70.07(13)
C(8)-C(9)-H(9)	126.2	C(20)-C(19)-Fe(1)	69.27(12)
C(10)-C(9)-H(9)	126.2	C(23)-C(19)-H(19)	126.1
Co(1)-C(9)-H(9)	126.3	C(20)-C(19)-H(19)	126.1
C(6)-C(10)-C(9)	108.11(17)	Fe(1)-C(19)-H(19)	126.1
C(6)-C(10)-C(11)	122.46(17)	C(21)-C(20)-C(19)	107.9(2)
C(9)-C(10)-C(11)	129.43(18)	C(21)-C(20)-Fe(1)	69.83(12)
C(6)-C(10)-Co(1)	69.43(11)	C(19)-C(20)-Fe(1)	69.89(12)
C(9)-C(10)-Co(1)	69.26(11)	C(21)-C(20)-H(20)	126.0
C(11)-C(10)-Co(1)	127.10(13)	C(19)-C(20)-H(20)	126.0
N(3)-C(11)-C(12)	104.84(16)	Fe(1)-C(20)-H(20)	125.8
N(3)-C(11)-C(10)	126.29(17)	C(20)-C(21)-C(22)	108.3(2)
C(12)-C(11)-C(10)	128.79(18)	C(20)-C(21)-Fe(1)	69.60(12)
N(1)-C(12)-C(11)	105.59(17)	C(22)-C(21)-Fe(1)	70.16(12)
N(1)-C(12)-H(12)	127.2	C(20)-C(21)-H(21)	125.9
C(11)-C(12)-H(12)	127.2	C(22)-C(21)-H(21)	125.9
N(3)-C(13)-H(13A)	109.5	Fe(1)-C(21)-H(21)	126.0
N(3)-C(13)-H(13B)	109.5	C(23)-C(22)-C(21)	107.8(2)
H(13A)-C(13)-H(13B)	109.5	C(23)-C(22)-Fe(1)	69.84(12)
N(3)-C(13)-H(13C)	109.5	C(21)-C(22)-Fe(1)	69.22(12)
H(13A)-C(13)-H(13C)	109.5	C(23)-C(22)-H(22)	126.1
H(13B)-C(13)-H(13C)	109.5	C(21)-C(22)-H(22)	126.1

Fe(1)-C(22)-H(22)	126.4	O(6)-S(2)-C(25)	103.76(11)
C(22)-C(23)-C(19)	108.2(2)	O(5)-S(2)-C(25)	102.60(11)
C(22)-C(23)-Fe(1)	69.75(13)	O(4)-S(2)-C(25)	102.34(11)
C(19)-C(23)-Fe(1)	69.43(12)	F(3)-C(24)-F(2)	107.3(2)
C(22)-C(23)-H(23)	125.9	F(3)-C(24)-F(1)	106.84(19)
C(19)-C(23)-H(23)	125.9	F(2)-C(24)-F(1)	108.0(2)
Fe(1)-C(23)-H(23)	126.5	F(3)-C(24)-S(1)	112.25(16)
O(2)-S(1)-O(1)	115.08(11)	F(2)-C(24)-S(1)	111.55(16)
O(2)-S(1)-O(3)	115.61(12)	F(1)-C(24)-S(1)	110.70(17)
O(1)-S(1)-O(3)	114.54(11)	F(5)-C(25)-F(4)	106.7(2)
O(2)-S(1)-C(24)	102.63(11)	F(5)-C(25)-F(6)	107.3(2)
O(1)-S(1)-C(24)	103.85(11)	F(4)-C(25)-F(6)	106.26(19)
O(3)-S(1)-C(24)	102.60(11)	F(5)-C(25)-S(2)	112.46(17)
O(6)-S(2)-O(5)	115.78(12)	F(4)-C(25)-S(2)	112.04(17)
O(6)-S(2)-O(4)	114.75(11)	F(6)-C(25)-S(2)	111.65(16)
O(5)-S(2)-O(4)	114.96(11)		

Table S22. Anisotropic displacement parameters (Å^{2}x 10^3) for **8a**. The anisotropic displacement factor exponent takes the form: -2p^2 [h^2 a*^{2}U^{11} + ... + 2 h k a* b* U^{12}].

	U^{11}	U^{22}	U^{33}	U^{23}	U^{13}	U^{12}
Co(1)	18(1)	15(1)	15(1)	2(1)	5(1)	0(1)
Fe(1)	18(1)	15(1)	15(1)	1(1)	5(1)	1(1)
N(1)	16(1)	14(1)	15(1)	1(1)	2(1)	1(1)
N(2)	20(1)	18(1)	17(1)	1(1)	4(1)	4(1)
N(3)	18(1)	16(1)	16(1)	1(1)	4(1)	3(1)
C(1)	32(1)	21(1)	50(2)	0(1)	26(1)	-5(1)
C(2)	21(1)	30(1)	40(1)	6(1)	-3(1)	-7(1)
C(3)	40(1)	27(1)	39(1)	-15(1)	18(1)	-14(1)
C(4)	28(1)	15(1)	70(2)	5(1)	14(1)	0(1)
C(5)	40(1)	27(1)	39(1)	16(1)	6(1)	-12(1)
C(6)	23(1)	14(1)	20(1)	4(1)	6(1)	2(1)
C(7)	28(1)	20(1)	19(1)	9(1)	6(1)	4(1)
C(8)	29(1)	22(1)	17(1)	6(1)	8(1)	-1(1)
C(9)	20(1)	19(1)	19(1)	3(1)	9(1)	-1(1)
C(10)	17(1)	13(1)	18(1)	3(1)	6(1)	-2(1)
C(11)	15(1)	13(1)	18(1)	4(1)	4(1)	1(1)
C(12)	18(1)	16(1)	17(1)	3(1)	4(1)	3(1)
C(13)	27(1)	22(1)	24(1)	1(1)	8(1)	11(1)
C(14)	19(1)	16(1)	16(1)	2(1)	4(1)	-1(1)
C(15)	22(1)	16(1)	17(1)	3(1)	2(1)	0(1)
C(16)	28(1)	22(1)	17(1)	4(1)	6(1)	-2(1)
C(17)	26(1)	27(1)	22(1)	0(1)	13(1)	-3(1)
C(18)	18(1)	24(1)	22(1)	1(1)	7(1)	1(1)
C(19)	34(1)	24(1)	26(1)	8(1)	12(1)	-3(1)

C(20)	23(1)	22(1)	34(1)	4(1)	14(1)	-2(1)
C(21)	24(1)	22(1)	24(1)	1(1)	4(1)	-5(1)
C(22)	35(1)	17(1)	34(1)	-2(1)	15(1)	1(1)
C(23)	33(1)	21(1)	39(1)	12(1)	11(1)	5(1)
S(1)	28(1)	25(1)	28(1)	8(1)	15(1)	7(1)
S(2)	28(1)	32(1)	18(1)	3(1)	7(1)	12(1)
C(24)	33(1)	29(1)	37(1)	4(1)	20(1)	5(1)
C(25)	33(1)	40(1)	24(1)	12(1)	11(1)	16(1)
O(1)	32(1)	36(1)	46(1)	18(1)	18(1)	15(1)
O(2)	33(1)	51(1)	28(1)	1(1)	9(1)	2(1)
O(3)	52(1)	34(1)	58(1)	26(1)	39(1)	18(1)
O(4)	42(1)	36(1)	34(1)	8(1)	9(1)	21(1)
O(5)	39(1)	52(1)	40(1)	2(1)	24(1)	7(1)
O(6)	53(1)	49(1)	20(1)	1(1)	-2(1)	16(1)
F(1)	70(1)	71(1)	78(1)	32(1)	46(1)	51(1)
F(2)	51(1)	47(1)	56(1)	-18(1)	29(1)	-9(1)
F(3)	53(1)	44(1)	46(1)	11(1)	34(1)	3(1)
F(4)	48(1)	38(1)	68(1)	13(1)	1(1)	23(1)
F(5)	98(2)	58(1)	72(1)	23(1)	64(1)	16(1)
F(6)	47(1)	42(1)	38(1)	15(1)	-6(1)	-2(1)

Table S23. Hydrogen coordinates (x 10^4) and isotropic displacement parameters ($Å^2$x 10^3) for **8a**.

	x	y	z	U(eq)
H(1)	-3211	5442	7906	42
H(2)	-3645	4980	6084	44
H(3)	-1664	3783	5744	49
H(4)	-41	3498	7347	49
H(5)	-976	4528	8679	46
H(6)	-575	8365	8476	23
H(7)	-1426	7885	6611	28
H(8)	285	6567	6009	28
H(9)	2213	6219	7498	24
H(12)	1474	9043	10270	22
H(13A)	3999	5789	8963	38
H(13B)	2304	4901	8629	38
H(13C)	3586	4917	9633	38
H(15)	4975	7766	12734	25
H(16)	4647	8852	14344	29
H(17)	2403	9907	13948	32
H(18)	1298	9496	12079	28
H(19)	5130	11038	11555	35
H(20)	7198	10539	12894	33
H(21)	6981	11568	14542	32
H(22)	4783	12703	14232	36
H(23)	3643	12375	12386	37

Table S24. Torsion angles [°] for **8a**.

C(12)-N(1)-N(2)-N(3)	0.5(2)	Co(1)-C(1)-C(5)-C(4)	-59.22(16)
C(14)-N(1)-N(2)-N(3)	176.91(16)	C(2)-C(1)-C(5)-Co(1)	59.05(16)
N(1)-N(2)-N(3)-C(11)	-1.1(2)	C(10)-C(6)-C(7)-C(8)	0.3(2)
N(1)-N(2)-N(3)-C(13)	178.75(16)	Co(1)-C(6)-C(7)-C(8)	-59.01(14)
C(5)-C(1)-C(2)-C(3)	-0.1(3)	C(10)-C(6)-C(7)-Co(1)	59.31(13)
Co(1)-C(1)-C(2)-C(3)	58.88(16)	C(6)-C(7)-C(8)-C(9)	0.0(2)
C(5)-C(1)-C(2)-Co(1)	-58.93(15)	Co(1)-C(7)-C(8)-C(9)	-59.32(14)
C(1)-C(2)-C(3)-C(4)	0.3(3)	C(6)-C(7)-C(8)-Co(1)	59.32(14)
Co(1)-C(2)-C(3)-C(4)	59.92(16)	C(7)-C(8)-C(9)-C(10)	-0.3(2)
C(1)-C(2)-C(3)-Co(1)	-59.67(16)	Co(1)-C(8)-C(9)-C(10)	-59.48(13)
C(2)-C(3)-C(4)-C(5)	-0.4(3)	C(7)-C(8)-C(9)-Co(1)	59.18(14)
Co(1)-C(3)-C(4)-C(5)	59.72(16)	C(7)-C(6)-C(10)-C(9)	-0.5(2)
C(2)-C(3)-C(4)-Co(1)	-60.08(16)	Co(1)-C(6)-C(10)-C(9)	58.63(13)
C(3)-C(4)-C(5)-C(1)	0.3(3)	C(7)-C(6)-C(10)-C(11)	179.21(17)
Co(1)-C(4)-C(5)-C(1)	59.70(15)	Co(1)-C(6)-C(10)-C(11)	-121.66(17)
C(3)-C(4)-C(5)-Co(1)	-59.38(16)	C(7)-C(6)-C(10)-Co(1)	-59.12(13)
C(2)-C(1)-C(5)-C(4)	-0.2(3)	C(8)-C(9)-C(10)-C(6)	0.5(2)

Co(1)-C(9)-C(10)-C(6)	-58.73(13)	C(15)-C(14)-C(18)-Fe(1)	59.94(14)
C(8)-C(9)-C(10)-C(11)	-179.19(18)	N(1)-C(14)-C(18)-Fe(1)	-124.73(19)
Co(1)-C(9)-C(10)-C(11)	121.6(2)	C(16)-C(17)-C(18)-C(14)	0.1(2)
C(8)-C(9)-C(10)-Co(1)	59.23(13)	Fe(1)-C(17)-C(18)-C(14)	59.22(13)
N(2)-N(3)-C(11)-C(12)	1.3(2)	C(16)-C(17)-C(18)-Fe(1)	-59.08(14)
C(13)-N(3)-C(11)-C(12)	-178.54(19)	C(23)-C(19)-C(20)-C(21)	0.0(2)
N(2)-N(3)-C(11)-C(10)	-175.79(17)	Fe(1)-C(19)-C(20)-C(21)	-59.72(14)
C(13)-N(3)-C(11)-C(10)	4.3(3)	C(23)-C(19)-C(20)-Fe(1)	59.69(15)
C(6)-C(10)-C(11)-N(3)	156.80(19)	C(19)-C(20)-C(21)-C(22)	0.0(2)
C(9)-C(10)-C(11)-N(3)	-23.6(3)	Fe(1)-C(20)-C(21)-C(22)	-59.71(15)
Co(1)-C(10)-C(11)-N(3)	69.1(2)	C(19)-C(20)-C(21)-Fe(1)	59.76(14)
C(6)-C(10)-C(11)-C(12)	-19.6(3)	C(20)-C(21)-C(22)-C(23)	0.0(2)
C(9)-C(10)-C(11)-C(12)	160.0(2)	Fe(1)-C(21)-C(22)-C(23)	-59.41(15)
Co(1)-C(10)-C(11)-C(12)	-107.3(2)	C(20)-C(21)-C(22)-Fe(1)	59.36(14)
N(2)-N(1)-C(12)-C(11)	0.3(2)	C(21)-C(22)-C(23)-C(19)	0.0(2)
C(14)-N(1)-C(12)-C(11)	-175.63(18)	Fe(1)-C(22)-C(23)-C(19)	-58.99(15)
N(3)-C(11)-C(12)-N(1)	-0.9(2)	C(21)-C(22)-C(23)-Fe(1)	59.02(15)
C(10)-C(11)-C(12)-N(1)	176.09(18)	C(20)-C(19)-C(23)-C(22)	0.0(2)
N(2)-N(1)-C(14)-C(15)	20.4(3)	Fe(1)-C(19)-C(23)-C(22)	59.19(15)
C(12)-N(1)-C(14)-C(15)	-163.82(19)	C(20)-C(19)-C(23)-Fe(1)	-59.19(15)
N(2)-N(1)-C(14)-C(18)	-154.27(19)	O(2)-S(1)-C(24)-F(3)	-61.4(2)
C(12)-N(1)-C(14)-C(18)	21.5(3)	O(1)-S(1)-C(24)-F(3)	58.73(19)
N(2)-N(1)-C(14)-Fe(1)	113.04(18)	O(3)-S(1)-C(24)-F(3)	178.29(17)
C(12)-N(1)-C(14)-Fe(1)	-71.2(3)	O(2)-S(1)-C(24)-F(2)	178.10(18)
C(18)-C(14)-C(15)-C(16)	0.1(2)	O(1)-S(1)-C(24)-F(2)	-61.7(2)
N(1)-C(14)-C(15)-C(16)	-175.25(18)	O(3)-S(1)-C(24)-F(2)	57.8(2)
Fe(1)-C(14)-C(15)-C(16)	59.78(13)	O(2)-S(1)-C(24)-F(1)	57.85(19)
C(18)-C(14)-C(15)-Fe(1)	-59.65(14)	O(1)-S(1)-C(24)-F(1)	178.02(16)
N(1)-C(14)-C(15)-Fe(1)	124.96(19)	O(3)-S(1)-C(24)-F(1)	-62.41(19)
C(14)-C(15)-C(16)-C(17)	0.0(2)	O(6)-S(2)-C(25)-F(5)	-55.2(2)
Fe(1)-C(15)-C(16)-C(17)	58.66(14)	O(5)-S(2)-C(25)-F(5)	-176.08(18)
C(14)-C(15)-C(16)-Fe(1)	-58.70(13)	O(4)-S(2)-C(25)-F(5)	64.47(19)
C(15)-C(16)-C(17)-C(18)	-0.1(2)	O(6)-S(2)-C(25)-F(4)	65.04(19)
Fe(1)-C(16)-C(17)-C(18)	58.79(15)	O(5)-S(2)-C(25)-F(4)	-55.85(19)
C(15)-C(16)-C(17)-Fe(1)	-58.85(14)	O(4)-S(2)-C(25)-F(4)	-175.30(16)
C(15)-C(14)-C(18)-C(17)	-0.2(2)	O(6)-S(2)-C(25)-F(6)	-175.89(16)
N(1)-C(14)-C(18)-C(17)	175.16(18)	O(5)-S(2)-C(25)-F(6)	63.21(19)
Fe(1)-C(14)-C(18)-C(17)	-60.11(14)	O(4)-S(2)-C(25)-F(6)	-56.24(18)

Triazolium salt (8b) – "4-cobaltoceniumyl-1-ferrocenyl-3-methyltriazolium bis(hexafluoridophosphate)"

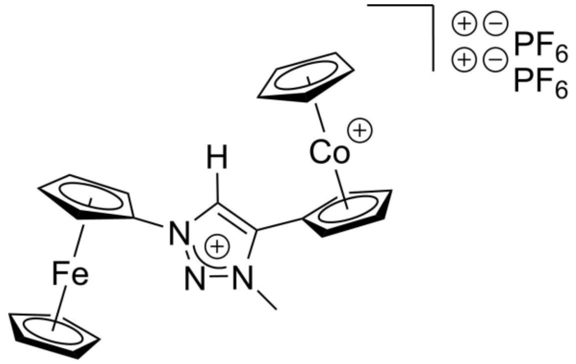

[745.15]

$C_{23}H_{22}N_3CoFe(PF_6)_2$

Analytical data:

IR (ATR [cm^{-1}]): 3160 (ν_{C-H}), 3127 (ν_{C-H}), 1600, 1441, 1418 ($\nu_{C=C}$), 812 (ν_{P-F}), 555 (ν_{P-F}), 500, 444, 427.

^{1}H-NMR (300 MHz, C_3D_6O, [ppm]): δ 4.47 (s, 5H, Fc-Cp), 4.59 (s, 3H, CH_3 of triazolium), 4.63 (s, broad, 2H, C3/C4 of substituted Fc-Cp), 5.35 (s, broad, 2H, J = 2.0 Hz, C2/C5 of substituted Fc-Cp), 6.14 (s, 5H, Cc-Cp), 6.29 (pseudo-t, 2H, J = 1.7 Hz, C3/C4 of substituted Cc-Cp), 6.73 (pseudo-t, 2H, J = 1.7 Hz, C2/C5 of substituted Cc-Cp), 9.83 (s, 1H, CH of triazolium).

^{13}C-NMR (75 MHz, C_3D_6O, [ppm]): δ 40.7 (CH_3 of triazolium), 64.0 (C3/C4 of substituted Fc-Cp), 69.7 (C2/C5 of substituted Fc-Cp), 72.2 (Fc-Cp), 85.9 (C3/C4 of substituted Cc-Cp), 86.6 (quart. carbon of substituted Fc-Cp), 87.3 (C2/C5 of substituted Cc-Cp), 88.3 (Cc-Cp), 92.7 (quart. carbon of substituted Cc-Cp), 130.4 (CH of triazolium), 137.6 (quart. carbon of triazolium).

MS (ESI pos, [m/z]): 600.01 ($[M(PF_6)]^+$).

Melting point [°C]: 275 (dec.).

Spectra

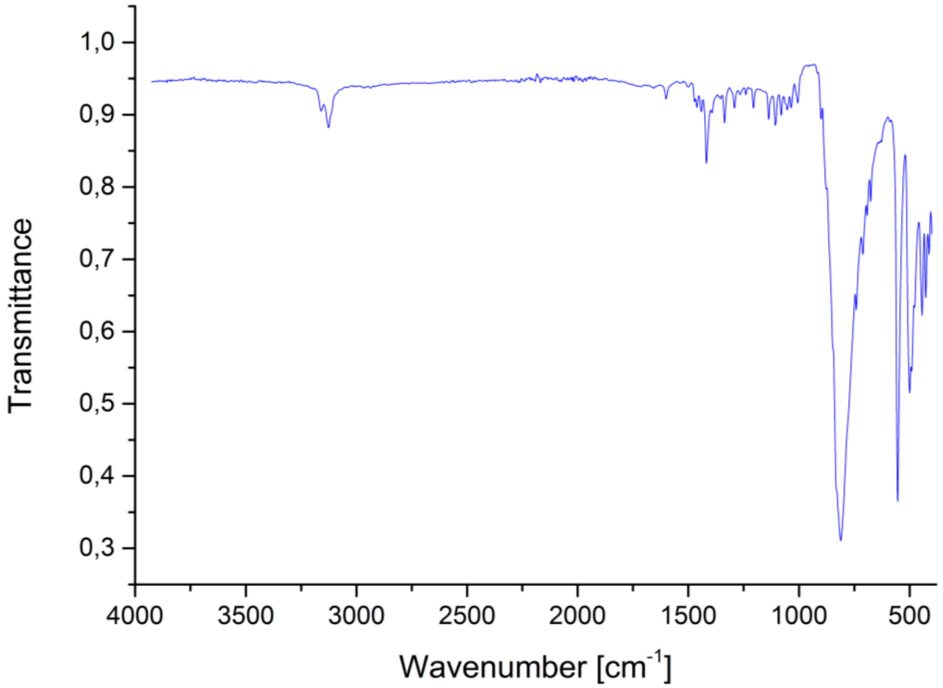

Figure S21. IR-spectrum (ATR, [cm^{-1}]) of “4-cobaltoceniumyl-1-ferrocenyl-3-methyltriazolium bis(hexafluoridophosphate)“.

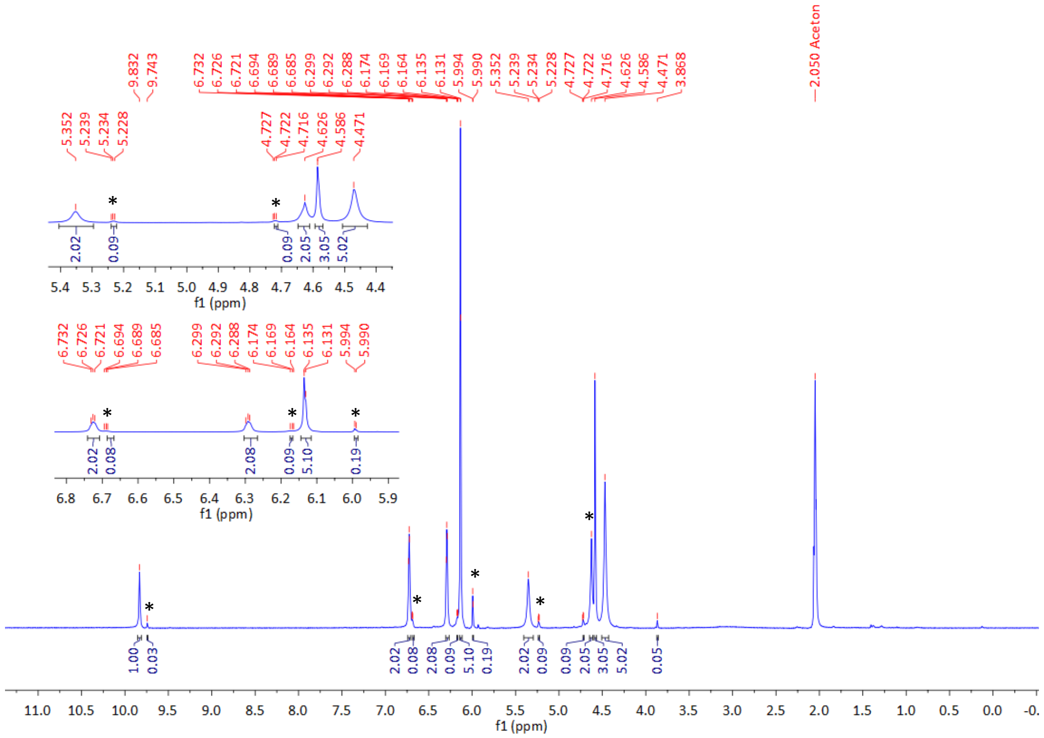

Figure S22. ^{1}H-NMR (300 MHz, C_3D_6O, [ppm]) of "4-cobaltoceniumyl-1-ferrocenyl-3-methyltriazolium bis(hexafluoridophosphate)" (impurity marked, < 2%).

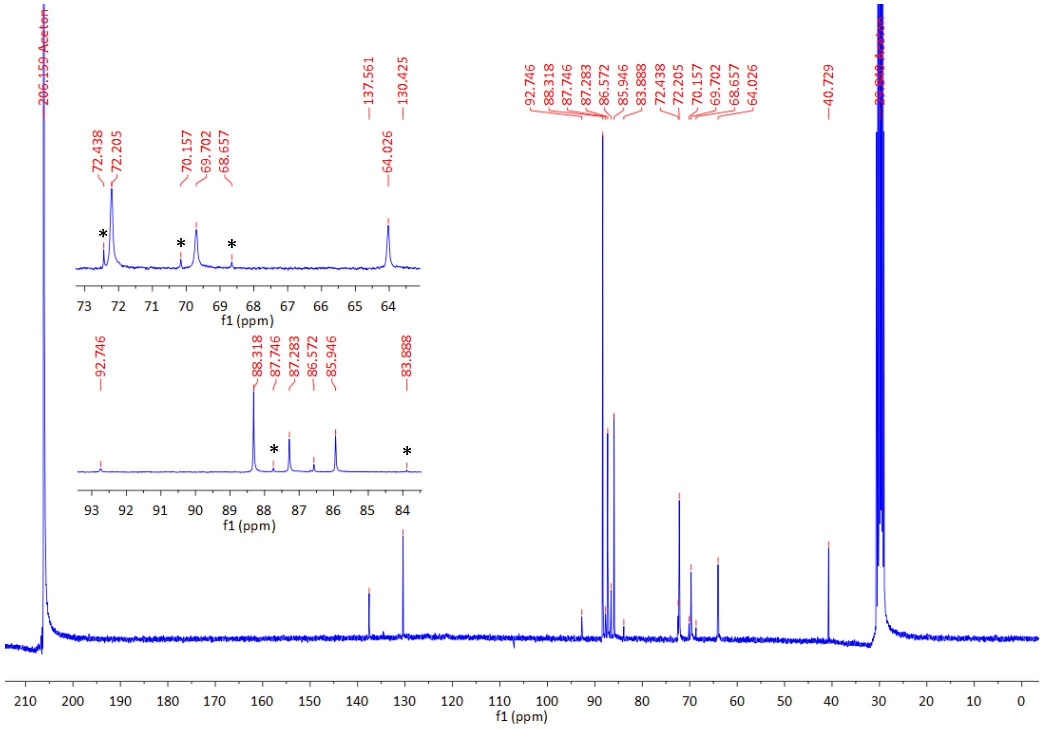

Figure S23. ^{13}C-NMR (75 MHz, C_3D_6O, [ppm]) of "4-cobaltoceniumyl-1-ferrocenyl-3-methyltriazolium bis(hexafluoridophosphate)" (impurity marked, < 2%).

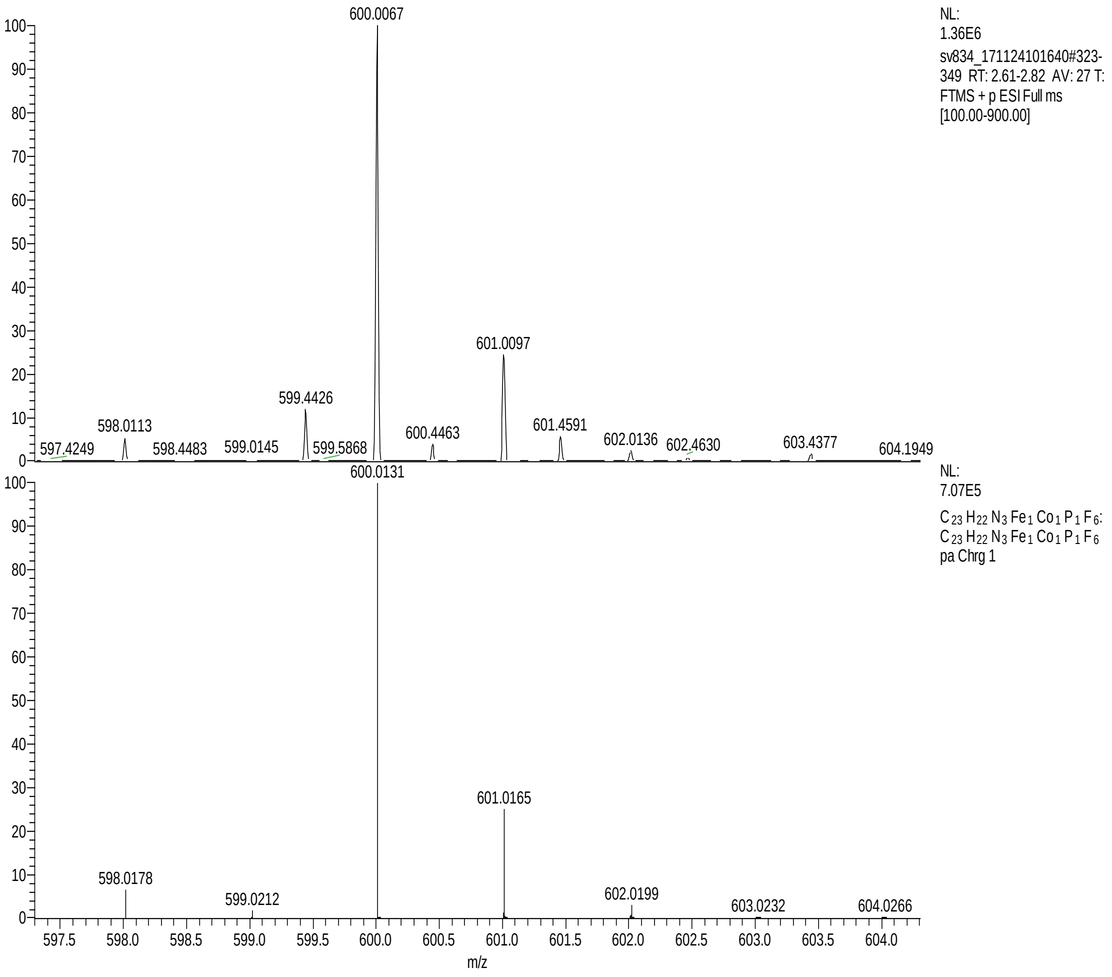

Figure S24. MS (ESI pos, [m/z]; *top*: experimental, *bottom*: simulated) of "4-cobaltoceniumyl-1-ferrocenyl-3-methyltriazolium bis(hexafluoridophosphate)".

Triazolium salt (9a) – "1-cobaltoceniumyl-4-ferrocenyl-3-methyltriazolium hexafluoridophosphate triflate"

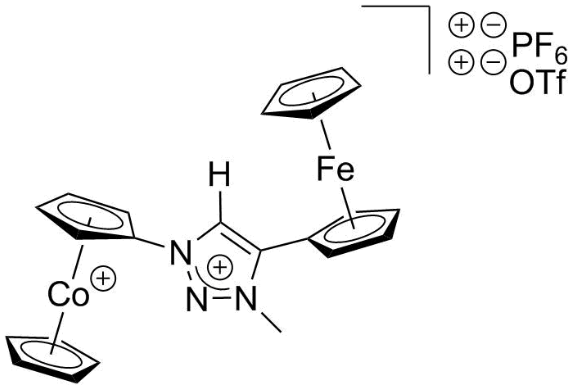

[749.25]

$C_{23}H_{22}N_3CoFe(PF_6)(CF_3SO_3)$

Analytical data:

IR (ATR [cm^{-1}]): 3127 (ν_{C-H}), 1601, 1521, 1420 ($\nu_{C=C}$), 1257 (ν_{SO3}), 1224 (ν_{CF3}), 1152 (ν_{CF3}), 1031 (ν_{SO3}), 827 (ν_{P-F}), 636, 556 (ν_{P-F}), 510, 443.

^{1}H-NMR (300 MHz, C_3D_6O, [ppm]): δ 4.39 (s, 5H, Fc-Cp), 4.60 (s, 3H, CH_3 of triazolium), 4.70 (pseudo-t, 2H, J = 1.7 Hz, C3/C4 of substituted Fc-Cp), 5.10 (pseudo-t, 2H, J = 1.8 Hz, C2/C5 of substituted Fc-Cp), 6.18 (s, 5H, Cc-Cp), 6.24 (pseudo-t, 2H, J = 2.3 Hz, C3/C4 of substituted Cc-Cp), 6.94 (pseudo-t, 2H, J = 2.3 Hz, C2/C5 of substituted Cc-Cp), 9.67 (s, 1H, CH of triazole).

^{13}C-NMR (75 MHz, C_3D_6O, [ppm]): δ 40.8 (CH_3 of triazole), 66.1 (quart. carbon of substituted Fc-Cp), 70.3 (C3/C4 of substituted Fc-Cp), 71.4 (Fc-Cp), 72.5 (C2/C5 of substituted Fc-Cp), 79.6 (C3/C4 of substituted Cc-Cp), 84.9 (C2/C5 of substituted Cc-Cp), 88.7 (Cc-Cp), 105.3 (quart. carbon of substituted Cc-Cp), 128.8 (CH of triazolium), 147.0 (quart. carbon of triazolium).

MS (MALDI pos, [m/z]): 604.03 (M^+ - PF_6^-).

Melting point [°C]: 236 (dec.).

Spectra

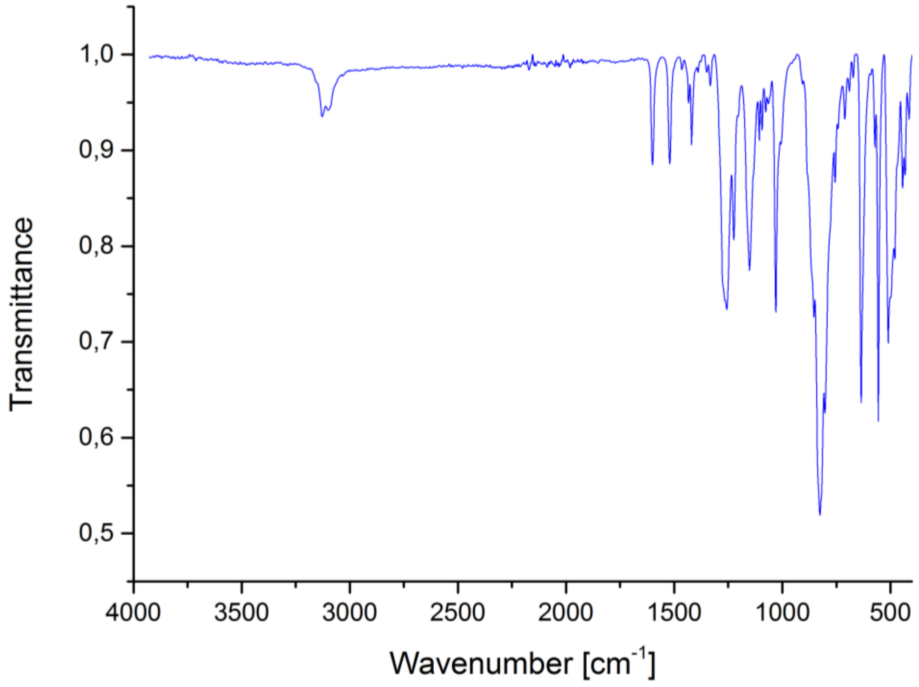

Figure S25. IR-spectrum (ATR, [cm^{-1}]) of "1-cobaltoceniumyl-4-ferrocenyl-3-methyltriazolium hexafluoridophosphate triflate".

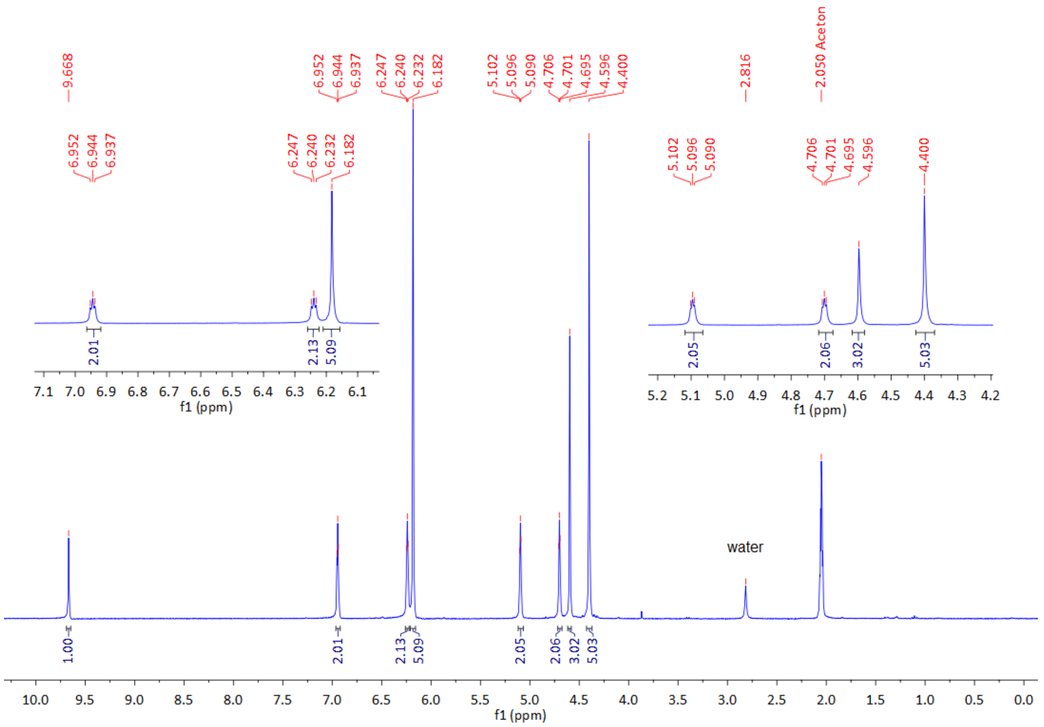

Figure S26. ^{1}H-NMR (300 MHz, C_3D_6O, [ppm]) of "1-cobaltoceniumyl-4-ferrocenyl-3-methyltriazolium hexafluoridophosphate triflate".

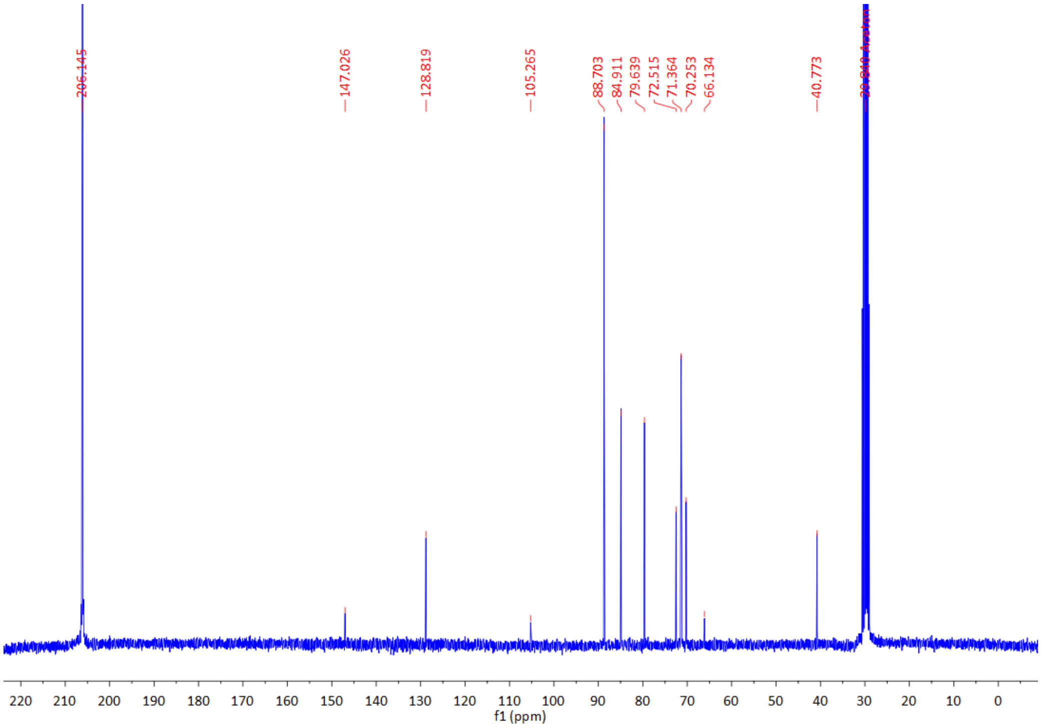

Figure S27. ^{13}C-NMR (75 MHz, C_3D_6O, [ppm]) of "1-cobaltoceniumyl-4-ferrocenyl-3-methyltriazolium hexafluoridophosphate triflate".

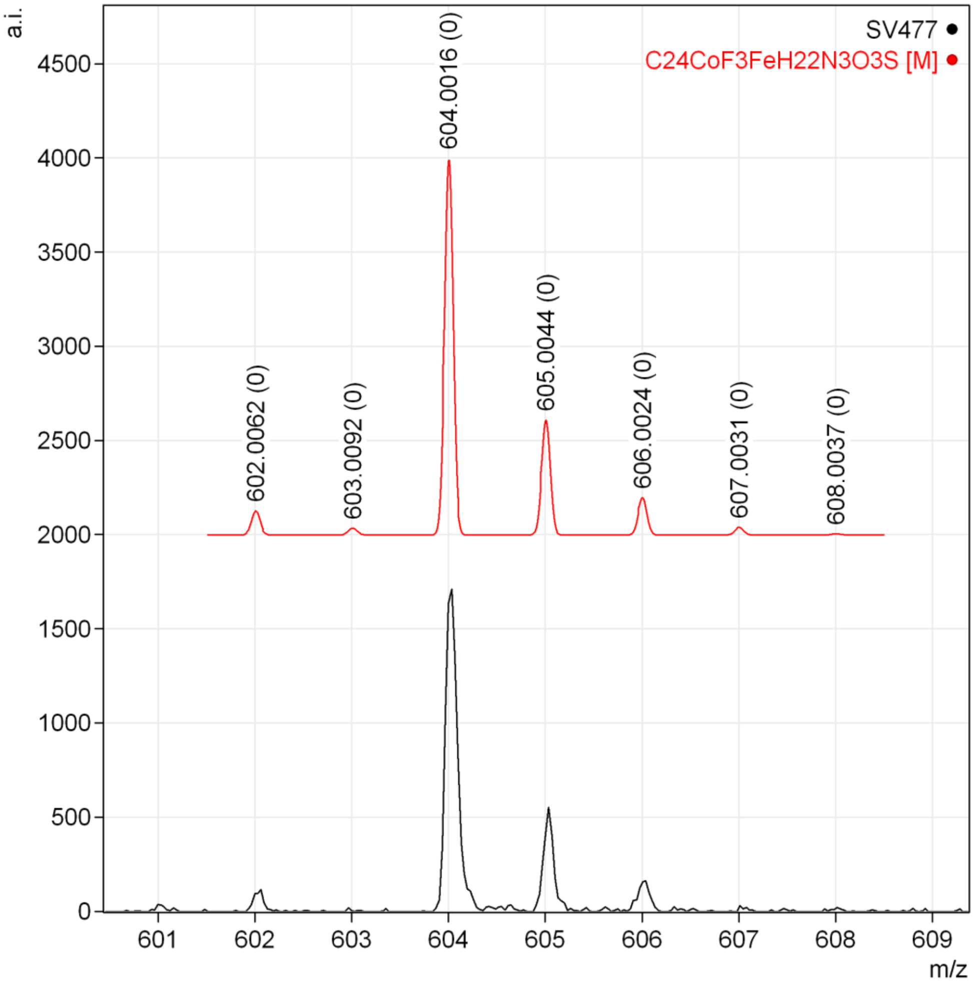

Figure S28. MS (MALDI pos, [m/z]; *top*: simulated, *bottom*: experimental) of "1-cobaltoceniumyl-4-ferrocenyl-3-methyltriazolium hexafluoridophosphate triflate".

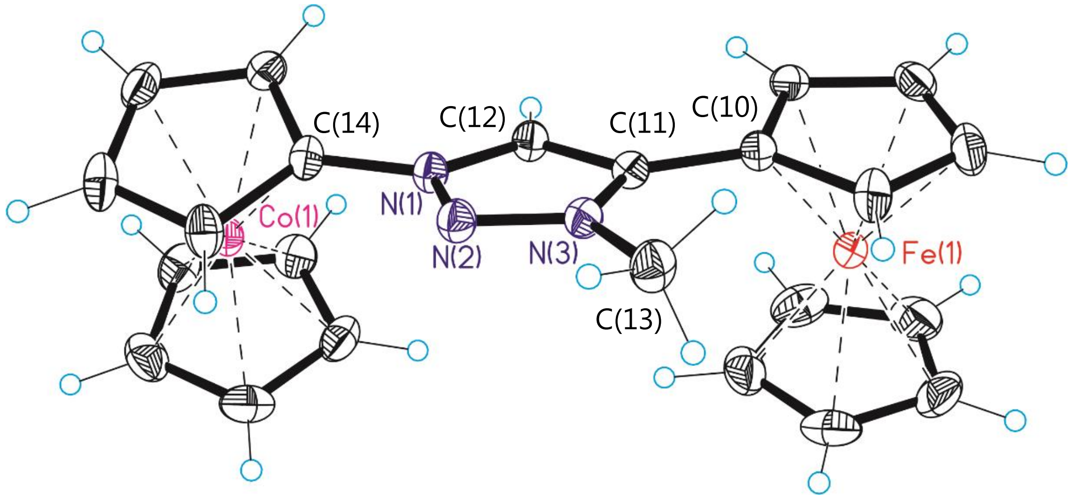

Figure S29. X-ray single crystal structure analysis of "1-cobaltoceniumyl-4-ferrocenyl-3-methyltriazolium hexafluoridophosphate triflate" (counterions omitted for clarity).

X-Ray single crystal structure analysis data

Table S25. Crystal data and structure refinement for **9a**.

Empirical formula	C23 H22 Co Fe N3 1.9 (F3C-SO3) 0.1 (PF6)	
Formula weight	752.94	
Temperature	193(2) K	
Wavelength	0.71073 Å	
Crystal system	Monoclinic	
Space group	$P2_1/c$ (no. 14)	
Unit cell dimensions	a = 13.9769(6) Å	α = 90°.
	b = 10.3569(4) Å	ß = 107.7460(10)°.
	c = 20.4038(9) Å	γ = 90°.
Volume	2813.1(2) $Å^3$	
Z	4	
Density (calculated)	1.778 Mg/m^3	
Absorption coefficient	1.340 mm^{-1}	
F(000)	1518	
Crystal size	0.180 x 0.160 x 0.120 mm^3	
Theta range for data collection	2.186 to 25.500°.	
Index ranges	-16<h<15, -12<k<12, -24<l<24	
Reflections collected	71057	
Independent reflections	5234 [R(int) = 0.0291]	
Completeness to theta = 25.242°	99.9 %	
Absorption correction	Semi-empirical from equivalents	
Max. and min. transmission	0.862 and 0.756	
Refinement method	Full-matrix least-squares on F^2	
Data / restraints / parameters	5234 / 0 / 454	
Goodness-of-fit on F^2	1.014	
Final R indices [I>2sigma(I)]	R1 = 0.0373, wR2 = 0.0937	
R indices (all data)	R1 = 0.0422, wR2 = 0.0969	
Extinction coefficient	n/a	
Largest diff. peak and hole	1.161 and -1.019 e.$Å^{-3}$	

Table S26. Atomic coordinates (x 10^4) and equivalent isotropic displacement parameters ($Å^2$x 10^3) for **9a**. U(eq) is defined as one third of the trace of the orthogonalized U^{ij} tensor.

	x	y	z	U(eq)
Co(1)	6179(1)	8212(1)	3521(1)	30(1)
Fe(1)	9083(1)	4936(1)	6548(1)	33(1)
N(1)	8368(2)	7473(2)	4341(1)	30(1)
N(2)	9124(2)	8248(2)	4650(1)	33(1)
N(3)	9643(2)	7578(2)	5193(1)	31(1)
C(1)	8603(3)	6355(4)	7063(2)	63(1)
C(2)	8908(3)	5273(4)	7489(2)	66(1)
C(3)	8319(3)	4224(4)	7173(2)	63(1)
C(4)	7640(3)	4654(4)	6554(2)	60(1)
C(5)	7817(3)	5973(4)	6484(2)	60(1)
C(6)	10404(2)	5496(3)	6406(2)	44(1)
C(7)	10510(2)	4263(3)	6715(2)	47(1)
C(8)	9810(2)	3413(3)	6277(2)	44(1)
C(9)	9249(2)	4117(3)	5687(1)	37(1)
C(10)	9625(2)	5413(3)	5759(1)	33(1)
C(11)	9229(2)	6401(2)	5240(1)	30(1)
C(12)	8396(2)	6336(3)	4673(1)	32(1)
C(13)	10567(2)	8167(3)	5638(2)	42(1)
C(14)	7669(2)	7881(3)	3714(1)	33(1)
C(15)	7112(2)	7048(3)	3184(1)	39(1)
C(16)	6538(2)	7859(3)	2646(1)	46(1)
C(17)	6737(2)	9157(3)	2849(2)	47(1)
C(18)	7440(2)	9191(3)	3520(2)	41(1)
C(19)	5755(2)	7877(3)	4373(2)	47(1)
C(20)	5178(2)	7117(3)	3819(2)	45(1)
C(21)	4677(2)	7952(3)	3277(2)	48(1)
C(22)	4945(2)	9221(3)	3491(2)	54(1)
C(23)	5609(3)	9184(3)	4170(2)	54(1)
S(1)	3660(1)	6642(1)	6305(1)	39(1)
C(24)	4196(3)	6732(4)	5599(2)	70(1)
O(1)	3755(2)	7925(2)	6573(1)	71(1)
O(2)	4293(2)	5709(2)	6754(1)	57(1)
O(3)	2662(2)	6211(3)	5981(2)	74(1)
F(1)	4113(3)	5607(3)	5278(1)	113(1)
F(2)	3750(3)	7579(3)	5138(1)	114(1)
F(3)	5154(3)	7060(5)	5829(2)	164(2)
S(2)	1696(2)	10349(2)	4088(1)	41(1)
C(25)	2347(7)	9568(9)	3568(5)	61(2)
O(4)	1365(6)	9241(6)	4370(4)	91(2)
O(5)	985(4)	11058(6)	3595(3)	74(1)
O(6)	2409(7)	11140(9)	4551(5)	92(3)
F(4)	2815(12)	10522(17)	3311(7)	91(4)
F(5)	3112(8)	8899(11)	3959(5)	167(5)
F(6)	1767(7)	8905(5)	3091(3)	129(2)
S(2A)	1802(2)	10203(3)	4248(2)	41(1)
C(25A)	2731(6)	9804(7)	3795(4)	33(2)

O(4A)	2013(8)	9394(8)	4801(5)	82(3)
O(5A)	884(5)	10150(12)	3715(5)	96(3)
O(6A)	2138(10)	11524(11)	4453(8)	56(3)
F(4A)	2615(18)	10640(30)	3275(10)	91(6)
F(5A)	3622(5)	9896(8)	4255(4)	80(2)
F(6A)	2604(9)	8568(8)	3624(7)	104(5)
P(1)	2209(6)	10162(8)	3923(4)	40(2)
F(1B)	1365(6)	9241(6)	4370(4)	91(2)
F(2B)	985(4)	11058(6)	3595(3)	74(1)
F(3B)	2409(7)	11140(9)	4551(5)	92(3)
F(4B)	2815(12)	10522(17)	3311(7)	91(4)
F(5B)	3112(8)	8899(11)	3959(5)	167(5)
F(6B)	1767(7)	8905(5)	3091(3)	129(2)

Table S27. Bond lengths [Å] and angles [°] for **9a**.

Co(1)-C(22)	2.002(3)	C(3)-C(4)	1.401(5)
Co(1)-C(23)	2.011(3)	C(3)-H(3)	0.9500
Co(1)-C(21)	2.023(3)	C(4)-C(5)	1.403(6)
Co(1)-C(17)	2.024(3)	C(4)-H(4)	0.9500
Co(1)-C(14)	2.028(3)	C(5)-H(5)	0.9500
Co(1)-C(16)	2.029(3)	C(6)-C(7)	1.412(4)
Co(1)-C(19)	2.030(3)	C(6)-C(10)	1.435(4)
Co(1)-C(20)	2.033(3)	C(6)-H(6)	0.9500
Co(1)-C(18)	2.034(3)	C(7)-C(8)	1.414(5)
Co(1)-C(15)	2.042(3)	C(7)-H(7)	0.9500
Fe(1)-C(9)	2.027(3)	C(8)-C(9)	1.420(4)
Fe(1)-C(3)	2.035(3)	C(8)-H(8)	0.9500
Fe(1)-C(1)	2.035(3)	C(9)-C(10)	1.433(4)
Fe(1)-C(2)	2.037(3)	C(9)-H(9)	0.9500
Fe(1)-C(10)	2.038(3)	C(10)-C(11)	1.456(4)
Fe(1)-C(5)	2.039(3)	C(11)-C(12)	1.369(4)
Fe(1)-C(6)	2.040(3)	C(12)-H(12)	0.9500
Fe(1)-C(7)	2.041(3)	C(13)-H(13A)	0.9800
Fe(1)-C(4)	2.041(3)	C(13)-H(13B)	0.9800
Fe(1)-C(8)	2.042(3)	C(13)-H(13C)	0.9800
N(1)-N(2)	1.323(3)	C(14)-C(15)	1.417(4)
N(1)-C(12)	1.353(3)	C(14)-C(18)	1.422(4)
N(1)-C(14)	1.417(3)	C(15)-C(16)	1.421(4)
N(2)-N(3)	1.322(3)	C(15)-H(15)	0.9500
N(3)-C(11)	1.366(3)	C(16)-C(17)	1.409(5)
N(3)-C(13)	1.465(3)	C(16)-H(16)	0.9500
C(1)-C(5)	1.403(5)	C(17)-C(18)	1.422(4)
C(1)-C(2)	1.403(5)	C(17)-H(17)	0.9500
C(1)-H(1)	0.9500	C(18)-H(18)	0.9500
C(2)-C(3)	1.397(6)	C(19)-C(20)	1.411(4)
C(2)-H(2)	0.9500	C(19)-C(23)	1.412(5)

C(19)-H(19)	0.9500	F(5)-F(6A)	0.891(14)
C(20)-C(21)	1.411(4)	F(5)-C(25A)	1.079(9)
C(20)-H(20)	0.9500	F(5)-F(5A)	1.295(14)
C(21)-C(22)	1.400(5)	F(6)-F(6A)	1.376(13)
C(21)-H(21)	0.9500	F(6)-C(25A)	1.888(11)
C(22)-C(23)	1.414(5)	S(2A)-P(1)	0.998(9)
C(22)-H(22)	0.9500	S(2A)-F(1B)	1.234(6)
C(23)-H(23)	0.9500	S(2A)-F(3B)	1.312(10)
S(1)-O(3)	1.422(3)	S(2A)-O(4A)	1.363(9)
S(1)-O(1)	1.427(2)	S(2A)-O(5A)	1.408(8)
S(1)-O(2)	1.435(2)	S(2A)-O(6A)	1.465(13)
S(1)-C(24)	1.818(4)	S(2A)-F(2B)	1.714(6)
C(24)-F(2)	1.298(4)	S(2A)-C(25A)	1.856(8)
C(24)-F(3)	1.321(5)	C(25A)-P(1)	0.924(10)
C(24)-F(1)	1.324(5)	C(25A)-F(5B)	1.079(9)
S(2)-O(5A)	1.176(7)	C(25A)-F(4B)	1.269(14)
S(2)-O(5)	1.389(6)	C(25A)-F(5A)	1.315(11)
S(2)-O(6)	1.408(10)	C(25A)-F(6A)	1.325(10)
S(2)-O(4)	1.424(6)	C(25A)-F(4A)	1.34(2)
S(2)-O(6A)	1.461(14)	C(25A)-F(6B)	1.888(11)
S(2)-O(4A)	1.702(9)	O(4A)-F(1B)	1.065(10)
S(2)-C(25)	1.788(9)	O(4A)-P(1)	2.053(11)
S(2)-C(25A)	1.816(8)	O(5A)-F(2B)	0.993(11)
C(25)-C(25A)	0.641(9)	O(5A)-F(1B)	1.606(14)
C(25)-F(6A)	1.090(12)	O(5A)-P(1)	1.770(11)
C(25)-F(6)	1.262(12)	O(6A)-F(3B)	0.543(15)
C(25)-F(5)	1.319(12)	O(6A)-P(1)	1.798(15)
C(25)-F(4A)	1.37(2)	F(4A)-P(1)	1.66(2)
C(25)-F(4)	1.374(17)	F(5A)-F(5B)	1.295(14)
C(25)-S(2A)	1.893(9)	F(5A)-P(1)	1.902(11)
O(4)-O(4A)	1.065(10)	F(6A)-F(5B)	0.891(14)
O(4)-S(2A)	1.234(6)	F(6A)-F(6B)	1.376(13)
O(4)-O(5A)	1.606(14)	F(6A)-P(1)	1.899(11)
O(5)-O(5A)	0.993(11)	P(1)-F(3B)	1.591(12)
O(5)-S(2A)	1.714(6)	P(1)-F(4B)	1.749(14)
O(6)-O(6A)	0.543(15)	P(1)-F(5B)	1.802(11)
O(6)-S(2A)	1.312(10)	P(1)-F(2B)	1.881(10)
F(4)-C(25A)	1.269(14)	P(1)-F(1B)	1.947(10)

C(22)-Co(1)-C(23)	41.25(14)	C(23)-Co(1)-C(16)	157.39(14)
C(22)-Co(1)-C(21)	40.71(14)	C(21)-Co(1)-C(16)	106.02(13)
C(23)-Co(1)-C(21)	68.81(14)	C(17)-Co(1)-C(16)	40.69(14)
C(22)-Co(1)-C(17)	103.49(14)	C(14)-Co(1)-C(16)	68.05(11)
C(23)-Co(1)-C(17)	121.05(15)	C(22)-Co(1)-C(19)	69.01(14)
C(21)-Co(1)-C(17)	118.92(12)	C(23)-Co(1)-C(19)	40.89(14)
C(22)-Co(1)-C(14)	156.74(13)	C(21)-Co(1)-C(19)	68.64(12)
C(23)-Co(1)-C(14)	123.11(12)	C(17)-Co(1)-C(19)	159.35(14)
C(21)-Co(1)-C(14)	162.38(13)	C(14)-Co(1)-C(19)	110.80(11)
C(17)-Co(1)-C(14)	68.18(11)	C(16)-Co(1)-C(19)	159.63(15)
C(22)-Co(1)-C(16)	120.58(14)	C(22)-Co(1)-C(20)	68.62(14)

C(23)-Co(1)-C(20)	68.61(14)	C(1)-Fe(1)-C(4)	67.59(16)
C(21)-Co(1)-C(20)	40.70(13)	C(2)-Fe(1)-C(4)	67.48(16)
C(17)-Co(1)-C(20)	156.20(13)	C(10)-Fe(1)-C(4)	130.17(13)
C(14)-Co(1)-C(20)	127.39(12)	C(5)-Fe(1)-C(4)	40.21(16)
C(16)-Co(1)-C(20)	122.68(13)	C(6)-Fe(1)-C(4)	168.73(15)
C(19)-Co(1)-C(20)	40.65(13)	C(7)-Fe(1)-C(4)	150.12(16)
C(22)-Co(1)-C(18)	118.60(14)	C(9)-Fe(1)-C(8)	40.84(11)
C(23)-Co(1)-C(18)	105.58(14)	C(3)-Fe(1)-C(8)	106.83(14)
C(21)-Co(1)-C(18)	154.31(13)	C(1)-Fe(1)-C(8)	165.28(15)
C(17)-Co(1)-C(18)	41.01(12)	C(2)-Fe(1)-C(8)	126.88(14)
C(14)-Co(1)-C(18)	40.99(11)	C(10)-Fe(1)-C(8)	68.72(11)
C(16)-Co(1)-C(18)	69.06(13)	C(5)-Fe(1)-C(8)	152.01(16)
C(19)-Co(1)-C(18)	124.40(13)	C(6)-Fe(1)-C(8)	68.51(13)
C(20)-Co(1)-C(18)	162.53(12)	C(7)-Fe(1)-C(8)	40.51(13)
C(22)-Co(1)-C(15)	158.50(13)	C(4)-Fe(1)-C(8)	117.73(15)
C(23)-Co(1)-C(15)	159.80(13)	N(2)-N(1)-C(12)	113.1(2)
C(21)-Co(1)-C(15)	124.04(13)	N(2)-N(1)-C(14)	118.6(2)
C(17)-Co(1)-C(15)	68.93(13)	C(12)-N(1)-C(14)	128.2(2)
C(14)-Co(1)-C(15)	40.75(11)	N(3)-N(2)-N(1)	103.6(2)
C(16)-Co(1)-C(15)	40.86(12)	N(2)-N(3)-C(11)	113.1(2)
C(19)-Co(1)-C(15)	124.74(13)	N(2)-N(3)-C(13)	117.1(2)
C(20)-Co(1)-C(15)	109.82(13)	C(11)-N(3)-C(13)	129.8(2)
C(18)-Co(1)-C(15)	69.62(13)	C(5)-C(1)-C(2)	107.9(4)
C(9)-Fe(1)-C(3)	127.73(15)	C(5)-C(1)-Fe(1)	70.01(19)
C(9)-Fe(1)-C(1)	153.18(14)	C(2)-C(1)-Fe(1)	69.92(19)
C(3)-Fe(1)-C(1)	67.67(16)	C(5)-C(1)-H(1)	126.0
C(9)-Fe(1)-C(2)	165.10(15)	C(2)-C(1)-H(1)	126.0
C(3)-Fe(1)-C(2)	40.12(17)	Fe(1)-C(1)-H(1)	125.6
C(1)-Fe(1)-C(2)	40.30(16)	C(3)-C(2)-C(1)	108.1(3)
C(9)-Fe(1)-C(10)	41.27(11)	C(3)-C(2)-Fe(1)	69.83(19)
C(3)-Fe(1)-C(10)	167.24(15)	C(1)-C(2)-Fe(1)	69.78(19)
C(1)-Fe(1)-C(10)	119.55(14)	C(3)-C(2)-H(2)	126.0
C(2)-Fe(1)-C(10)	152.00(16)	C(1)-C(2)-H(2)	126.0
C(9)-Fe(1)-C(5)	119.44(13)	Fe(1)-C(2)-H(2)	126.0
C(3)-Fe(1)-C(5)	67.68(15)	C(2)-C(3)-C(4)	108.1(3)
C(1)-Fe(1)-C(5)	40.27(15)	C(2)-C(3)-Fe(1)	70.0(2)
C(2)-Fe(1)-C(5)	67.63(15)	C(4)-C(3)-Fe(1)	70.13(19)
C(10)-Fe(1)-C(5)	110.26(12)	C(2)-C(3)-H(3)	126.0
C(9)-Fe(1)-C(6)	69.35(12)	C(4)-C(3)-H(3)	126.0
C(3)-Fe(1)-C(6)	149.70(15)	Fe(1)-C(3)-H(3)	125.5
C(1)-Fe(1)-C(6)	108.88(15)	C(3)-C(4)-C(5)	108.0(3)
C(2)-Fe(1)-C(6)	117.34(15)	C(3)-C(4)-Fe(1)	69.6(2)
C(10)-Fe(1)-C(6)	41.21(11)	C(5)-C(4)-Fe(1)	69.8(2)
C(5)-Fe(1)-C(6)	130.32(15)	C(3)-C(4)-H(4)	126.0
C(9)-Fe(1)-C(7)	68.72(12)	C(5)-C(4)-H(4)	126.0
C(3)-Fe(1)-C(7)	116.38(14)	Fe(1)-C(4)-H(4)	126.1
C(1)-Fe(1)-C(7)	128.28(16)	C(1)-C(5)-C(4)	107.9(3)
C(2)-Fe(1)-C(7)	107.02(14)	C(1)-C(5)-Fe(1)	69.72(19)
C(10)-Fe(1)-C(7)	68.58(11)	C(4)-C(5)-Fe(1)	70.0(2)
C(5)-Fe(1)-C(7)	167.17(16)	C(1)-C(5)-H(5)	126.1
C(6)-Fe(1)-C(7)	40.49(12)	C(4)-C(5)-H(5)	126.1
C(9)-Fe(1)-C(4)	108.64(13)	Fe(1)-C(5)-H(5)	125.8
C(3)-Fe(1)-C(4)	40.22(16)	C(7)-C(6)-C(10)	107.7(3)

C(7)-C(6)-Fe(1)	69.81(18)
C(10)-C(6)-Fe(1)	69.36(16)
C(7)-C(6)-H(6)	126.2
C(10)-C(6)-H(6)	126.2
Fe(1)-C(6)-H(6)	126.2
C(6)-C(7)-C(8)	108.8(3)
C(6)-C(7)-Fe(1)	69.71(17)
C(8)-C(7)-Fe(1)	69.81(17)
C(6)-C(7)-H(7)	125.6
C(8)-C(7)-H(7)	125.6
Fe(1)-C(7)-H(7)	126.5
C(7)-C(8)-C(9)	108.2(3)
C(7)-C(8)-Fe(1)	69.68(17)
C(9)-C(8)-Fe(1)	68.99(15)
C(7)-C(8)-H(8)	125.9
C(9)-C(8)-H(8)	125.9
Fe(1)-C(8)-H(8)	127.0
C(8)-C(9)-C(10)	107.7(3)
C(8)-C(9)-Fe(1)	70.16(16)
C(10)-C(9)-Fe(1)	69.80(15)
C(8)-C(9)-H(9)	126.2
C(10)-C(9)-H(9)	126.2
Fe(1)-C(9)-H(9)	125.5
C(9)-C(10)-C(6)	107.6(2)
C(9)-C(10)-C(11)	122.6(2)
C(6)-C(10)-C(11)	129.9(3)
C(9)-C(10)-Fe(1)	68.94(15)
C(6)-C(10)-Fe(1)	69.44(16)
C(11)-C(10)-Fe(1)	126.47(19)
N(3)-C(11)-C(12)	104.7(2)
N(3)-C(11)-C(10)	127.1(2)
C(12)-C(11)-C(10)	128.1(2)
N(1)-C(12)-C(11)	105.5(2)
N(1)-C(12)-H(12)	127.2
C(11)-C(12)-H(12)	127.2
N(3)-C(13)-H(13A)	109.5
N(3)-C(13)-H(13B)	109.5
H(13A)-C(13)-H(13B)	109.5
N(3)-C(13)-H(13C)	109.5
H(13A)-C(13)-H(13C)	109.5
H(13B)-C(13)-H(13C)	109.5
C(15)-C(14)-N(1)	125.1(2)
C(15)-C(14)-C(18)	110.1(2)
N(1)-C(14)-C(18)	124.8(2)
C(15)-C(14)-Co(1)	70.18(16)
N(1)-C(14)-Co(1)	127.63(18)
C(18)-C(14)-Co(1)	69.75(16)
C(14)-C(15)-C(16)	106.2(3)
C(14)-C(15)-Co(1)	69.07(16)
C(16)-C(15)-Co(1)	69.07(17)
C(14)-C(15)-H(15)	126.9
C(16)-C(15)-H(15)	126.9
Co(1)-C(15)-H(15)	126.5
C(17)-C(16)-C(15)	108.8(3)
C(17)-C(16)-Co(1)	69.45(17)
C(15)-C(16)-Co(1)	70.07(16)
C(17)-C(16)-H(16)	125.6
C(15)-C(16)-H(16)	125.6
Co(1)-C(16)-H(16)	126.5
C(16)-C(17)-C(18)	108.9(3)
C(16)-C(17)-Co(1)	69.86(17)
C(18)-C(17)-Co(1)	69.88(16)
C(16)-C(17)-H(17)	125.5
C(18)-C(17)-H(17)	125.5
Co(1)-C(17)-H(17)	126.3
C(17)-C(18)-C(14)	106.0(3)
C(17)-C(18)-Co(1)	69.11(17)
C(14)-C(18)-Co(1)	69.26(15)
C(17)-C(18)-H(18)	127.0
C(14)-C(18)-H(18)	127.0
Co(1)-C(18)-H(18)	126.2
C(20)-C(19)-C(23)	107.7(3)
C(20)-C(19)-Co(1)	69.81(16)
C(23)-C(19)-Co(1)	68.86(18)
C(20)-C(19)-H(19)	126.1
C(23)-C(19)-H(19)	126.1
Co(1)-C(19)-H(19)	126.8
C(21)-C(20)-C(19)	108.1(3)
C(21)-C(20)-Co(1)	69.24(17)
C(19)-C(20)-Co(1)	69.54(17)
C(21)-C(20)-H(20)	125.9
C(19)-C(20)-H(20)	125.9
Co(1)-C(20)-H(20)	126.8
C(22)-C(21)-C(20)	108.1(3)
C(22)-C(21)-Co(1)	68.84(18)
C(20)-C(21)-Co(1)	70.05(17)
C(22)-C(21)-H(21)	126.0
C(20)-C(21)-H(21)	126.0
Co(1)-C(21)-H(21)	126.7
C(21)-C(22)-C(23)	108.2(3)
C(21)-C(22)-Co(1)	70.45(18)
C(23)-C(22)-Co(1)	69.74(18)
C(21)-C(22)-H(22)	125.9
C(23)-C(22)-H(22)	125.9
Co(1)-C(22)-H(22)	125.5
C(19)-C(23)-C(22)	107.9(3)
C(19)-C(23)-Co(1)	70.25(17)
C(22)-C(23)-Co(1)	69.01(19)
C(19)-C(23)-H(23)	126.1
C(22)-C(23)-H(23)	126.1
Co(1)-C(23)-H(23)	126.2
O(3)-S(1)-O(1)	115.73(18)
O(3)-S(1)-O(2)	114.31(17)
O(1)-S(1)-O(2)	114.52(14)
O(3)-S(1)-C(24)	103.34(19)
O(1)-S(1)-C(24)	104.1(2)

O(2)-S(1)-C(24)	102.56(17)	F(5)-C(25)-S(2)	110.1(7)
F(2)-C(24)-F(3)	107.2(4)	F(4A)-C(25)-S(2)	98.6(12)
F(2)-C(24)-F(1)	106.7(3)	F(4)-C(25)-S(2)	106.8(10)
F(3)-C(24)-F(1)	109.2(5)	C(25A)-C(25)-S(2A)	76.9(12)
F(2)-C(24)-S(1)	112.7(3)	F(6A)-C(25)-S(2A)	116.6(9)
F(3)-C(24)-S(1)	110.5(3)	F(6)-C(25)-S(2A)	116.5(7)
F(1)-C(24)-S(1)	110.5(3)	F(5)-C(25)-S(2A)	99.9(7)
O(5A)-S(2)-O(5)	44.6(6)	F(4A)-C(25)-S(2A)	105.3(11)
O(5A)-S(2)-O(6)	151.9(6)	F(4)-C(25)-S(2A)	112.0(10)
O(5)-S(2)-O(6)	112.5(5)	S(2)-C(25)-S(2A)	10.39(12)
O(5A)-S(2)-O(4)	75.6(7)	O(4A)-O(4)-S(2A)	72.3(6)
O(5)-S(2)-O(4)	118.1(4)	O(4A)-O(4)-S(2)	85.0(6)
O(6)-S(2)-O(4)	116.6(6)	S(2A)-O(4)-S(2)	12.71(18)
O(5A)-S(2)-O(6A)	130.6(7)	O(4A)-O(4)-O(5A)	129.4(8)
O(5)-S(2)-O(6A)	91.5(6)	S(2A)-O(4)-O(5A)	57.7(4)
O(6)-S(2)-O(6A)	21.7(6)	S(2)-O(4)-O(5A)	45.2(4)
O(4)-S(2)-O(6A)	127.2(6)	O(5A)-O(5)-S(2)	56.3(5)
O(5A)-S(2)-O(4A)	113.6(8)	O(5A)-O(5)-S(2A)	55.2(5)
O(5)-S(2)-O(4A)	150.3(4)	S(2)-O(5)-S(2A)	4.79(16)
O(6)-S(2)-O(4A)	79.5(6)	O(6A)-O(6)-S(2A)	95(3)
O(4)-S(2)-O(4A)	38.5(4)	O(6A)-O(6)-S(2)	85(2)
O(6A)-S(2)-O(4A)	95.1(7)	S(2A)-O(6)-S(2)	14.21(18)
O(5A)-S(2)-C(25)	95.8(6)	C(25A)-F(4)-C(25)	27.7(5)
O(5)-S(2)-C(25)	100.9(4)	F(6A)-F(5)-C(25A)	83.9(9)
O(6)-S(2)-C(25)	106.1(5)	F(6A)-F(5)-F(5A)	149.5(12)
O(4)-S(2)-C(25)	99.3(4)	C(25A)-F(5)-F(5A)	66.5(9)
O(6A)-S(2)-C(25)	117.8(6)	F(6A)-F(5)-C(25)	55.1(8)
O(4A)-S(2)-C(25)	101.4(4)	C(25A)-F(5)-C(25)	28.9(5)
O(5A)-S(2)-C(25A)	116.1(5)	F(5A)-F(5)-C(25)	95.3(9)
O(5)-S(2)-C(25A)	113.0(4)	C(25)-F(6)-F(6A)	48.6(6)
O(6)-S(2)-C(25A)	86.0(5)	C(25)-F(6)-C(25A)	5.2(5)
O(4)-S(2)-C(25A)	105.8(4)	F(6A)-F(6)-C(25A)	44.5(4)
O(6A)-S(2)-C(25A)	99.8(5)	P(1)-S(2A)-F(1B)	121.1(6)
O(4A)-S(2)-C(25A)	94.3(4)	P(1)-S(2A)-F(3B)	85.9(7)
C(25)-S(2)-C(25A)	20.5(3)	F(1B)-S(2A)-F(3B)	142.3(7)
C(25A)-C(25)-F(6A)	96.4(15)	O(4)-S(2A)-O(6)	142.3(7)
C(25A)-C(25)-F(6)	164.5(16)	P(1)-S(2A)-O(4A)	120.0(6)
F(6A)-C(25)-F(6)	71.2(9)	F(1B)-S(2A)-O(4A)	48.1(4)
C(25A)-C(25)-F(5)	54.4(12)	O(4)-S(2A)-O(4A)	48.1(4)
F(6A)-C(25)-F(5)	42.1(7)	F(3B)-S(2A)-O(4A)	96.8(7)
F(6)-C(25)-F(5)	113.1(10)	O(6)-S(2A)-O(4A)	96.8(7)
C(25A)-C(25)-F(4A)	74.0(15)	P(1)-S(2A)-O(5A)	93.1(7)
F(6A)-C(25)-F(4A)	133.7(15)	F(1B)-S(2A)-O(5A)	74.5(6)
F(6)-C(25)-F(4A)	107.5(11)	O(4)-S(2A)-O(5A)	74.5(6)
F(5)-C(25)-F(4A)	114.3(14)	F(3B)-S(2A)-O(5A)	134.3(7)
C(25A)-C(25)-F(4)	67.0(13)	O(6)-S(2A)-O(5A)	134.3(7)
F(6A)-C(25)-F(4)	122.9(12)	O(4A)-S(2A)-O(5A)	122.1(8)
F(6)-C(25)-F(4)	111.4(10)	P(1)-S(2A)-O(6A)	91.8(7)
F(5)-C(25)-F(4)	102.5(11)	F(1B)-S(2A)-O(6A)	146.4(6)
F(4A)-C(25)-F(4)	12.3(18)	O(4)-S(2A)-O(6A)	146.4(6)
C(25A)-C(25)-S(2)	82.2(12)	F(3B)-S(2A)-O(6A)	21.7(7)
F(6A)-C(25)-S(2)	125.4(9)	O(6)-S(2A)-O(6A)	21.7(7)
F(6)-C(25)-S(2)	112.3(7)	O(4A)-S(2A)-O(6A)	111.7(8)

O(5A)-S(2A)-O(6A)	113.2(7)	F(4B)-C(25A)-F(4A)	12.5(18)
P(1)-S(2A)-F(2B)	83.3(5)	F(5A)-C(25A)-F(4A)	112.3(12)
F(1B)-S(2A)-F(2B)	108.7(5)	F(6A)-C(25A)-F(4A)	116.1(14)
F(3B)-S(2A)-F(2B)	99.6(5)	C(25)-C(25A)-S(2)	77.3(12)
O(4A)-S(2A)-F(2B)	152.4(5)	F(5)-C(25A)-S(2)	122.3(7)
O(5A)-S(2A)-F(2B)	35.4(5)	F(4)-C(25A)-S(2)	110.2(9)
O(6A)-S(2A)-F(2B)	79.6(6)	F(5A)-C(25A)-S(2)	114.7(6)
O(4)-S(2A)-O(5)	108.7(5)	F(6A)-C(25A)-S(2)	109.3(7)
O(6)-S(2A)-O(5)	99.6(5)	F(4A)-C(25A)-S(2)	98.3(12)
O(4A)-S(2A)-O(5)	152.4(5)	C(25)-C(25A)-S(2A)	83.4(12)
O(5A)-S(2A)-O(5)	35.4(5)	P(1)-C(25A)-S(2A)	15.7(5)
O(6A)-S(2A)-O(5)	79.6(6)	F(5B)-C(25A)-S(2A)	113.0(6)
P(1)-S(2A)-C(25A)	14.5(5)	F(5)-C(25A)-S(2A)	113.0(6)
F(1B)-S(2A)-C(25A)	112.7(4)	F(4)-C(25A)-S(2A)	120.1(9)
O(4)-S(2A)-C(25A)	112.7(4)	F(4B)-C(25A)-S(2A)	120.1(9)
F(3B)-S(2A)-C(25A)	87.1(5)	F(5A)-C(25A)-S(2A)	106.3(6)
O(6)-S(2A)-C(25A)	87.1(5)	F(6A)-C(25A)-S(2A)	106.8(7)
O(4A)-S(2A)-C(25A)	105.5(4)	F(4A)-C(25A)-S(2A)	108.5(12)
O(5A)-S(2A)-C(25A)	102.5(5)	S(2)-C(25A)-S(2A)	10.85(12)
O(6A)-S(2A)-C(25A)	97.9(5)	P(1)-C(25A)-F(6B)	88.3(8)
F(2B)-S(2A)-C(25A)	97.4(3)	F(5B)-C(25A)-F(6B)	88.8(9)
O(5)-S(2A)-C(25A)	97.4(3)	F(4B)-C(25A)-F(6B)	84.9(9)
O(4)-S(2A)-C(25)	101.6(4)	F(5A)-C(25A)-F(6B)	151.7(7)
O(6)-S(2A)-C(25)	104.8(6)	F(6A)-C(25A)-F(6B)	46.8(6)
O(4A)-S(2A)-C(25)	111.0(5)	F(4A)-C(25A)-F(6B)	80.2(11)
O(5A)-S(2A)-C(25)	84.0(5)	S(2A)-C(25A)-F(6B)	92.5(4)
O(6A)-S(2A)-C(25)	111.5(6)	F(1B)-O(4A)-S(2A)	59.6(6)
O(5)-S(2A)-C(25)	86.0(4)	O(4)-O(4A)-S(2A)	59.6(6)
C(25A)-S(2A)-C(25)	19.7(3)	O(4)-O(4A)-S(2)	56.4(5)
P(1)-C(25A)-F(5B)	128.1(10)	S(2A)-O(4A)-S(2)	3.2(2)
C(25)-C(25A)-F(5)	96.8(15)	F(1B)-O(4A)-P(1)	69.1(6)
C(25)-C(25A)-F(4)	85.3(14)	S(2A)-O(4A)-P(1)	24.9(3)
F(5)-C(25A)-F(4)	126.7(11)	O(5)-O(5A)-S(2)	79.2(7)
P(1)-C(25A)-F(4B)	104.7(11)	F(2B)-O(5A)-S(2A)	89.4(7)
F(5B)-C(25A)-F(4B)	126.7(11)	O(5)-O(5A)-S(2A)	89.4(7)
C(25)-C(25A)-F(5A)	161.1(15)	S(2)-O(5A)-S(2A)	11.7(2)
P(1)-C(25A)-F(5A)	115.2(9)	O(5)-O(5A)-O(4)	134.9(8)
F(5B)-C(25A)-F(5A)	64.6(9)	S(2)-O(5A)-O(4)	59.2(5)
F(5)-C(25A)-F(5A)	64.6(9)	S(2A)-O(5A)-O(4)	47.8(4)
F(4)-C(25A)-F(5A)	102.8(10)	F(2B)-O(5A)-F(1B)	134.9(8)
F(4B)-C(25A)-F(5A)	102.8(10)	S(2A)-O(5A)-F(1B)	47.8(4)
C(25)-C(25A)-F(6A)	54.9(12)	F(2B)-O(5A)-P(1)	80.5(7)
P(1)-C(25A)-F(6A)	114.1(10)	S(2A)-O(5A)-P(1)	34.3(3)
F(5B)-C(25A)-F(6A)	42.0(8)	F(1B)-O(5A)-P(1)	70.3(5)
F(5)-C(25A)-F(6A)	42.0(8)	O(6)-O(6A)-S(2)	74(2)
F(4)-C(25A)-F(6A)	113.5(12)	F(3B)-O(6A)-S(2A)	63(2)
F(4B)-C(25A)-F(6A)	113.5(12)	O(6)-O(6A)-S(2A)	63(2)
F(5A)-C(25A)-F(6A)	106.3(9)	S(2)-O(6A)-S(2A)	13.71(18)
C(25)-C(25A)-F(4A)	78.6(15)	F(3B)-O(6A)-P(1)	59(2)
P(1)-C(25A)-F(4A)	92.9(13)	S(2A)-O(6A)-P(1)	33.7(4)
F(5B)-C(25A)-F(4A)	137.4(13)	C(25A)-F(4A)-C(25)	27.4(6)
F(5)-C(25A)-F(4A)	137.4(13)	C(25A)-F(4A)-P(1)	33.7(6)
F(4)-C(25A)-F(4A)	12.5(18)	F(5B)-F(5A)-C(25A)	48.9(5)

F(5)-F(5A)-C(25A)	48.9(5)
F(5B)-F(5A)-P(1)	65.5(5)
C(25A)-F(5A)-P(1)	26.1(4)
F(5)-F(6A)-C(25)	82.8(9)
F(5B)-F(6A)-C(25A)	54.1(7)
F(5)-F(6A)-C(25A)	54.1(7)
C(25)-F(6A)-C(25A)	28.7(5)
F(5B)-F(6A)-F(6B)	142.6(12)
C(25A)-F(6A)-F(6B)	88.7(8)
F(5)-F(6A)-F(6)	142.6(12)
C(25)-F(6A)-F(6)	60.2(9)
C(25A)-F(6A)-F(6)	88.7(8)
F(5B)-F(6A)-P(1)	70.1(8)
C(25A)-F(6A)-P(1)	26.4(4)
F(6B)-F(6A)-P(1)	76.9(5)
C(25A)-P(1)-S(2A)	149.8(10)
C(25A)-P(1)-F(3B)	121.6(9)
S(2A)-P(1)-F(3B)	55.4(6)
C(25A)-P(1)-F(4A)	53.5(11)
S(2A)-P(1)-F(4A)	156.4(12)
F(3B)-P(1)-F(4A)	115.6(11)
C(25A)-P(1)-F(4B)	44.6(8)
S(2A)-P(1)-F(4B)	164.5(10)
F(3B)-P(1)-F(4B)	115.8(9)
F(4A)-P(1)-F(4B)	9.4(14)
C(25A)-P(1)-O(5A)	142.0(9)
S(2A)-P(1)-O(5A)	52.6(6)
F(3B)-P(1)-O(5A)	96.4(6)
F(4A)-P(1)-O(5A)	113.4(10)
F(4B)-P(1)-O(5A)	122.0(8)
C(25A)-P(1)-O(6A)	132.6(10)
S(2A)-P(1)-O(6A)	54.5(6)
F(3B)-P(1)-O(6A)	17.1(6)
F(4A)-P(1)-O(6A)	109.8(11)
F(4B)-P(1)-O(6A)	112.9(8)
O(5A)-P(1)-O(6A)	84.5(6)
C(25A)-P(1)-F(5B)	28.1(6)
S(2A)-P(1)-F(5B)	122.5(7)
F(3B)-P(1)-F(5B)	118.7(7)
F(4A)-P(1)-F(5B)	81.0(11)
F(4B)-P(1)-F(5B)	72.5(7)
O(5A)-P(1)-F(5B)	132.3(8)
O(6A)-P(1)-F(5B)	135.3(8)
C(25A)-P(1)-F(2B)	143.9(9)
S(2A)-P(1)-F(2B)	64.8(5)
F(3B)-P(1)-F(2B)	83.7(5)
F(4A)-P(1)-F(2B)	93.7(10)
F(4B)-P(1)-F(2B)	103.1(7)
O(5A)-P(1)-F(2B)	31.4(4)
O(6A)-P(1)-F(2B)	67.4(6)
F(5B)-P(1)-F(2B)	157.2(7)
C(25A)-P(1)-F(6A)	39.6(7)
S(2A)-P(1)-F(6A)	122.1(7)
F(3B)-P(1)-F(6A)	144.7(7)
F(4A)-P(1)-F(6A)	78.4(11)
F(4B)-P(1)-F(6A)	72.8(8)
O(5A)-P(1)-F(6A)	107.6(6)
O(6A)-P(1)-F(6A)	161.8(8)
F(5B)-P(1)-F(6A)	27.7(5)
F(2B)-P(1)-F(6A)	129.5(6)
C(25A)-P(1)-F(5A)	38.7(7)
S(2A)-P(1)-F(5A)	120.6(7)
F(3B)-P(1)-F(5A)	84.1(5)
F(4A)-P(1)-F(5A)	76.0(9)
F(4B)-P(1)-F(5A)	67.0(7)
O(5A)-P(1)-F(5A)	168.9(7)
O(6A)-P(1)-F(5A)	98.1(6)
F(5B)-P(1)-F(5A)	40.8(5)
F(2B)-P(1)-F(5A)	158.6(6)
F(6A)-P(1)-F(5A)	67.5(5)
C(25A)-P(1)-F(1B)	125.4(8)
S(2A)-P(1)-F(1B)	32.8(4)
F(3B)-P(1)-F(1B)	85.2(6)
F(4A)-P(1)-F(1B)	156.8(10)
F(4B)-P(1)-F(1B)	159.0(8)
O(5A)-P(1)-F(1B)	50.9(5)
O(6A)-P(1)-F(1B)	87.2(6)
F(5B)-P(1)-F(1B)	98.2(5)
F(2B)-P(1)-F(1B)	78.1(4)
F(6A)-P(1)-F(1B)	90.1(5)
F(5A)-P(1)-F(1B)	118.3(5)
O(4A)-F(1B)-S(2A)	72.3(6)
O(4A)-F(1B)-O(5A)	129.4(8)
S(2A)-F(1B)-O(5A)	57.7(4)
O(4A)-F(1B)-P(1)	80.2(6)
S(2A)-F(1B)-P(1)	26.0(3)
O(5A)-F(1B)-P(1)	58.8(4)
O(5A)-F(2B)-S(2A)	55.2(5)
O(5A)-F(2B)-P(1)	68.1(6)
S(2A)-F(2B)-P(1)	31.8(3)
O(6A)-F(3B)-S(2A)	95(3)
O(6A)-F(3B)-P(1)	104(3)
S(2A)-F(3B)-P(1)	38.7(4)
C(25A)-F(4B)-P(1)	30.7(5)
F(6A)-F(5B)-C(25A)	83.9(9)
F(6A)-F(5B)-F(5A)	149.5(12)
C(25A)-F(5B)-F(5A)	66.5(9)
F(6A)-F(5B)-P(1)	82.2(8)
C(25A)-F(5B)-P(1)	23.8(5)
F(5A)-F(5B)-P(1)	73.7(6)
F(6A)-F(6B)-C(25A)	44.5(4)
F(6A)-F(6B)-P(1)	62.9(4)
C(25A)-F(6B)-P(1)	26.4(3)

Table S28. Anisotropic displacement parameters ($Å^2 x 10^3$) for **9a**. The anisotropic displacement factor exponent takes the form: $-2p^2 [h^2 a^{*2}U^{11} + ... + 2 h k a^* b^* U^{12}]$.

	U^{11}	U^{22}	U^{33}	U^{23}	U^{13}	U^{12}
Co(1)	32(1)	28(1)	26(1)	3(1)	4(1)	0(1)
Fe(1)	39(1)	34(1)	25(1)	1(1)	8(1)	7(1)
N(1)	30(1)	31(1)	27(1)	2(1)	5(1)	0(1)
N(2)	32(1)	33(1)	31(1)	2(1)	6(1)	-1(1)
N(3)	31(1)	31(1)	28(1)	-1(1)	6(1)	0(1)
C(1)	79(3)	50(2)	71(2)	-20(2)	39(2)	6(2)
C(2)	69(2)	99(3)	31(2)	-6(2)	17(2)	22(2)
C(3)	89(3)	58(2)	61(2)	13(2)	50(2)	17(2)
C(4)	52(2)	82(3)	57(2)	-18(2)	32(2)	-8(2)
C(5)	58(2)	78(3)	49(2)	16(2)	26(2)	32(2)
C(6)	35(2)	52(2)	38(2)	10(1)	3(1)	1(1)
C(7)	40(2)	58(2)	42(2)	18(1)	10(1)	16(1)
C(8)	57(2)	37(2)	46(2)	11(1)	26(2)	18(1)
C(9)	47(2)	32(1)	33(1)	1(1)	16(1)	6(1)
C(10)	34(1)	35(1)	30(1)	3(1)	9(1)	5(1)
C(11)	31(1)	31(1)	28(1)	-1(1)	9(1)	2(1)
C(12)	33(1)	30(1)	30(1)	2(1)	7(1)	-2(1)
C(13)	37(2)	41(2)	40(2)	-2(1)	-1(1)	-6(1)
C(14)	31(1)	40(1)	28(1)	7(1)	7(1)	1(1)
C(15)	40(2)	44(2)	30(1)	-4(1)	8(1)	5(1)
C(16)	44(2)	67(2)	25(1)	5(1)	7(1)	6(2)
C(17)	43(2)	54(2)	41(2)	22(1)	9(1)	0(1)
C(18)	39(2)	38(2)	43(2)	13(1)	8(1)	-6(1)
C(19)	41(2)	71(2)	30(1)	4(1)	13(1)	4(2)
C(20)	46(2)	44(2)	50(2)	4(1)	22(1)	-6(1)
C(21)	33(2)	68(2)	40(2)	3(2)	6(1)	-4(1)
C(22)	45(2)	52(2)	65(2)	10(2)	16(2)	17(2)
C(23)	51(2)	54(2)	59(2)	-21(2)	19(2)	3(2)
S(1)	45(1)	28(1)	36(1)	-4(1)	2(1)	-3(1)
C(24)	82(3)	80(3)	44(2)	15(2)	11(2)	5(2)
O(1)	115(2)	32(1)	53(1)	-8(1)	5(1)	0(1)
O(2)	79(2)	43(1)	39(1)	7(1)	3(1)	11(1)
O(3)	47(1)	77(2)	89(2)	-17(2)	5(1)	-12(1)
F(1)	192(3)	99(2)	58(2)	2(1)	51(2)	52(2)
F(2)	196(3)	85(2)	58(1)	34(1)	32(2)	19(2)
F(3)	89(2)	302(6)	108(2)	30(3)	43(2)	-47(3)
O(4)	117(5)	76(3)	107(5)	2(4)	74(4)	-15(4)
O(5)	63(3)	83(3)	68(3)	-5(3)	8(2)	20(3)
O(6)	75(5)	113(8)	73(4)	-47(5)	3(4)	-25(5)
F(4)	87(6)	107(5)	106(7)	59(4)	71(6)	40(4)
F(5)	164(10)	216(11)	170(9)	145(8)	125(8)	149(8)
F(6)	210(7)	83(4)	105(4)	-19(3)	65(5)	28(4)
O(4A)	104(7)	69(5)	100(6)	26(5)	71(5)	29(5)
O(5A)	29(3)	132(8)	106(7)	-60(7)	-11(4)	0(5)
O(6A)	66(7)	38(4)	78(7)	-15(4)	43(6)	-1(4)
F(4A)	97(12)	120(11)	54(6)	24(6)	19(6)	28(8)

F(5A)	57(4)	114(6)	68(4)	-10(4)	20(3)	8(4)
F(6A)	130(10)	40(4)	168(12)	-50(6)	85(8)	0(5)
F(1B)	117(5)	76(3)	107(5)	2(4)	74(4)	-15(4)
F(2B)	63(3)	83(3)	68(3)	-5(3)	8(2)	20(3)
F(3B)	75(5)	113(8)	73(4)	-47(5)	3(4)	-25(5)
F(4B)	87(6)	107(5)	106(7)	59(4)	71(6)	40(4)
F(5B)	164(10)	216(11)	170(9)	145(8)	125(8)	149(8)
F(6B)	210(7)	83(4)	105(4)	-19(3)	65(5)	28(4)

Table S29. Hydrogen coordinates (x 10^4) and isotropic displacement parameters (Å^{2}x 10^3) for **9a**.

	x	y	z	U(eq)
H(1)	8880	7199	7151	76
H(2)	9425	5256	7917	79
H(3)	8369	3368	7347	76
H(4)	7147	4143	6237	73
H(5)	7467	6511	6111	71
H(6)	10782	6245	6592	52
H(7)	10974	4041	7147	57
H(8)	9729	2524	6361	53
H(9)	8720	3787	5313	44
H(12)	7935	5640	4541	38
H(13A)	10747	8909	5402	63
H(13B)	11111	7530	5740	63
H(13C)	10459	8453	6067	63
H(15)	7120	6131	3188	46
H(16)	6092	7574	2219	56
H(17)	6449	9890	2581	56
H(18)	7703	9937	3785	49
H(19)	6168	7566	4804	56
H(20)	5135	6201	3813	54
H(21)	4235	7697	2843	57
H(22)	4719	9978	3225	65
H(23)	5904	9909	4441	65

Table S30. Torsion angles [°] for **9a**.

C(12)-N(1)-N(2)-N(3)	-0.1(3)	N(3)-C(11)-C(12)-N(1)	-0.8(3)
C(14)-N(1)-N(2)-N(3)	-177.8(2)	C(10)-C(11)-C(12)-N(1)	-177.7(2)
N(1)-N(2)-N(3)-C(11)	-0.5(3)	N(2)-N(1)-C(14)-C(15)	151.4(3)
N(1)-N(2)-N(3)-C(13)	177.9(2)	C(12)-N(1)-C(14)-C(15)	-26.0(4)
C(5)-C(1)-C(2)-C(3)	0.4(4)	N(2)-N(1)-C(14)-C(18)	-27.3(4)
Fe(1)-C(1)-C(2)-C(3)	-59.5(2)	C(12)-N(1)-C(14)-C(18)	155.3(3)
C(5)-C(1)-C(2)-Fe(1)	59.9(2)	N(2)-N(1)-C(14)-Co(1)	-117.5(2)
C(1)-C(2)-C(3)-C(4)	-0.6(4)	C(12)-N(1)-C(14)-Co(1)	65.1(4)
Fe(1)-C(2)-C(3)-C(4)	-60.1(2)	N(1)-C(14)-C(15)-C(16)	-177.9(2)
C(1)-C(2)-C(3)-Fe(1)	59.5(2)	C(18)-C(14)-C(15)-C(16)	1.0(3)
C(2)-C(3)-C(4)-C(5)	0.5(4)	Co(1)-C(14)-C(15)-C(16)	59.37(19)
Fe(1)-C(3)-C(4)-C(5)	-59.5(2)	N(1)-C(14)-C(15)-Co(1)	122.7(3)
C(2)-C(3)-C(4)-Fe(1)	60.0(2)	C(18)-C(14)-C(15)-Co(1)	-58.4(2)
C(2)-C(1)-C(5)-C(4)	-0.1(4)	C(14)-C(15)-C(16)-C(17)	-0.5(3)
Fe(1)-C(1)-C(5)-C(4)	59.8(2)	Co(1)-C(15)-C(16)-C(17)	58.8(2)
C(2)-C(1)-C(5)-Fe(1)	-59.9(2)	C(14)-C(15)-C(16)-Co(1)	-59.37(19)
C(3)-C(4)-C(5)-C(1)	-0.3(4)	C(15)-C(16)-C(17)-C(18)	-0.1(4)
Fe(1)-C(4)-C(5)-C(1)	-59.7(2)	Co(1)-C(16)-C(17)-C(18)	59.1(2)
C(3)-C(4)-C(5)-Fe(1)	59.4(2)	C(15)-C(16)-C(17)-Co(1)	-59.2(2)
C(10)-C(6)-C(7)-C(8)	-0.2(3)	C(16)-C(17)-C(18)-C(14)	0.6(3)
Fe(1)-C(6)-C(7)-C(8)	59.0(2)	Co(1)-C(17)-C(18)-C(14)	59.77(19)
C(10)-C(6)-C(7)-Fe(1)	-59.2(2)	C(16)-C(17)-C(18)-Co(1)	-59.1(2)
C(6)-C(7)-C(8)-C(9)	-0.6(3)	C(15)-C(14)-C(18)-C(17)	-1.0(3)
Fe(1)-C(7)-C(8)-C(9)	58.4(2)	N(1)-C(14)-C(18)-C(17)	177.9(2)
C(6)-C(7)-C(8)-Fe(1)	-59.0(2)	Co(1)-C(14)-C(18)-C(17)	-59.7(2)
C(7)-C(8)-C(9)-C(10)	1.2(3)	C(15)-C(14)-C(18)-Co(1)	58.7(2)
Fe(1)-C(8)-C(9)-C(10)	59.97(19)	N(1)-C(14)-C(18)-Co(1)	-122.4(3)
C(7)-C(8)-C(9)-Fe(1)	-58.8(2)	C(23)-C(19)-C(20)-C(21)	-0.1(3)
C(8)-C(9)-C(10)-C(6)	-1.3(3)	Co(1)-C(19)-C(20)-C(21)	-58.7(2)
Fe(1)-C(9)-C(10)-C(6)	58.9(2)	C(23)-C(19)-C(20)-Co(1)	58.6(2)
C(8)-C(9)-C(10)-C(11)	179.1(2)	C(19)-C(20)-C(21)-C(22)	0.3(4)
Fe(1)-C(9)-C(10)-C(11)	-120.7(3)	Co(1)-C(20)-C(21)-C(22)	-58.5(2)
C(8)-C(9)-C(10)-Fe(1)	-60.20(19)	C(19)-C(20)-C(21)-Co(1)	58.8(2)
C(7)-C(6)-C(10)-C(9)	1.0(3)	C(20)-C(21)-C(22)-C(23)	-0.5(4)
Fe(1)-C(6)-C(10)-C(9)	-58.58(19)	Co(1)-C(21)-C(22)-C(23)	-59.7(2)
C(7)-C(6)-C(10)-C(11)	-179.5(3)	C(20)-C(21)-C(22)-Co(1)	59.3(2)
Fe(1)-C(6)-C(10)-C(11)	120.9(3)	C(20)-C(19)-C(23)-C(22)	-0.2(4)
C(7)-C(6)-C(10)-Fe(1)	59.5(2)	Co(1)-C(19)-C(23)-C(22)	59.0(2)
N(2)-N(3)-C(11)-C(12)	0.9(3)	C(20)-C(19)-C(23)-Co(1)	-59.2(2)
C(13)-N(3)-C(11)-C(12)	-177.3(3)	C(21)-C(22)-C(23)-C(19)	0.4(4)
N(2)-N(3)-C(11)-C(10)	177.8(2)	Co(1)-C(22)-C(23)-C(19)	-59.7(2)
C(13)-N(3)-C(11)-C(10)	-0.3(4)	C(21)-C(22)-C(23)-Co(1)	60.2(2)
C(9)-C(10)-C(11)-N(3)	-167.0(3)	O(3)-S(1)-C(24)-F(2)	63.5(4)
C(6)-C(10)-C(11)-N(3)	13.5(5)	O(1)-S(1)-C(24)-F(2)	-57.7(4)
Fe(1)-C(10)-C(11)-N(3)	106.3(3)	O(2)-S(1)-C(24)-F(2)	-177.4(3)
C(9)-C(10)-C(11)-C(12)	9.2(4)	O(3)-S(1)-C(24)-F(3)	-176.7(4)
C(6)-C(10)-C(11)-C(12)	-170.3(3)	O(1)-S(1)-C(24)-F(3)	62.1(4)
Fe(1)-C(10)-C(11)-C(12)	-77.5(3)	O(2)-S(1)-C(24)-F(3)	-57.6(4)
N(2)-N(1)-C(12)-C(11)	0.6(3)	O(3)-S(1)-C(24)-F(1)	-55.7(3)
C(14)-N(1)-C(12)-C(11)	178.1(2)	O(1)-S(1)-C(24)-F(1)	-177.0(3)

O(2)-S(1)-C(24)-F(1)	63.4(3)	C(25)-S(2)-O(4)-O(4A)	96.6(7)
O(5A)-S(2)-C(25)-C(25A)	173.2(13)	C(25A)-S(2)-O(4)-O(4A)	76.7(7)
O(5)-S(2)-C(25)-C(25A)	128.4(12)	O(5A)-S(2)-O(4)-S(2A)	-168.1(11)
O(6)-S(2)-C(25)-C(25A)	10.9(13)	O(5)-S(2)-O(4)-S(2A)	-154.0(10)
O(4)-S(2)-C(25)-C(25A)	-110.5(12)	O(6)-S(2)-O(4)-S(2A)	-15.2(9)
O(6A)-S(2)-C(25)-C(25A)	30.7(14)	O(6A)-S(2)-O(4)-S(2A)	-37.7(10)
O(4A)-S(2)-C(25)-C(25A)	-71.3(12)	O(4A)-S(2)-O(4)-S(2A)	1.6(10)
O(5A)-S(2)-C(25)-F(6A)	-94.6(15)	C(25)-S(2)-O(4)-S(2A)	98.2(10)
O(5)-S(2)-C(25)-F(6A)	-139.4(13)	C(25A)-S(2)-O(4)-S(2A)	78.3(9)
O(6)-S(2)-C(25)-F(6A)	103.2(13)	O(5)-S(2)-O(4)-O(5A)	14.1(6)
O(4)-S(2)-C(25)-F(6A)	-18.2(14)	O(6)-S(2)-O(4)-O(5A)	152.9(6)
O(6A)-S(2)-C(25)-F(6A)	123.0(13)	O(6A)-S(2)-O(4)-O(5A)	130.4(8)
O(4A)-S(2)-C(25)-F(6A)	20.9(14)	O(4A)-S(2)-O(4)-O(5A)	169.7(8)
C(25A)-S(2)-C(25)-F(6A)	92.3(17)	C(25)-S(2)-O(4)-O(5A)	-93.7(6)
O(5A)-S(2)-C(25)-F(6)	-12.3(10)	C(25A)-S(2)-O(4)-O(5A)	-113.6(6)
O(5)-S(2)-C(25)-F(6)	-57.1(7)	O(6)-S(2)-O(5)-O(5A)	-160.1(9)
O(6)-S(2)-C(25)-F(6)	-174.6(7)	O(4)-S(2)-O(5)-O(5A)	-19.7(9)
O(4)-S(2)-C(25)-F(6)	64.0(8)	O(6A)-S(2)-O(5)-O(5A)	-154.1(9)
O(6A)-S(2)-C(25)-F(6)	-154.8(8)	O(4A)-S(2)-O(5)-O(5A)	-50.9(13)
O(4A)-S(2)-C(25)-F(6)	103.2(8)	C(25)-S(2)-O(5)-O(5A)	87.2(9)
C(25A)-S(2)-C(25)-F(6)	174.5(17)	C(25A)-S(2)-O(5)-O(5A)	104.5(8)
O(5A)-S(2)-C(25)-F(5)	-139.4(11)	O(5A)-S(2)-O(5)-S(2A)	76(2)
O(5)-S(2)-C(25)-F(5)	175.8(9)	O(6)-S(2)-O(5)-S(2A)	-84(2)
O(6)-S(2)-C(25)-F(5)	58.3(10)	O(4)-S(2)-O(5)-S(2A)	56(2)
O(4)-S(2)-C(25)-F(5)	-63.0(10)	O(6A)-S(2)-O(5)-S(2A)	-78(2)
O(6A)-S(2)-C(25)-F(5)	78.2(11)	O(4A)-S(2)-O(5)-S(2A)	25.1(19)
O(4A)-S(2)-C(25)-F(5)	-23.9(10)	C(25)-S(2)-O(5)-S(2A)	163(2)
C(25A)-S(2)-C(25)-F(5)	47.5(12)	C(25A)-S(2)-O(5)-S(2A)	-179(100)
O(5A)-S(2)-C(25)-F(4A)	100.7(13)	O(5A)-S(2)-O(6)-O(6A)	-14(4)
O(5)-S(2)-C(25)-F(4A)	55.9(11)	O(5)-S(2)-O(6)-O(6A)	16(3)
O(6)-S(2)-C(25)-F(4A)	-61.6(12)	O(4)-S(2)-O(6)-O(6A)	-125(2)
O(4)-S(2)-C(25)-F(4A)	177.1(11)	O(4A)-S(2)-O(6)-O(6A)	-135(3)
O(6A)-S(2)-C(25)-F(4A)	-41.7(13)	C(25)-S(2)-O(6)-O(6A)	126(2)
O(4A)-S(2)-C(25)-F(4A)	-143.8(11)	C(25A)-S(2)-O(6)-O(6A)	130(2)
C(25A)-S(2)-C(25)-F(4A)	-72.5(15)	O(5A)-S(2)-O(6)-S(2A)	123.3(19)
O(5A)-S(2)-C(25)-F(4)	110.1(11)	O(5)-S(2)-O(6)-S(2A)	153.8(9)
O(5)-S(2)-C(25)-F(4)	65.3(10)	O(4)-S(2)-O(6)-S(2A)	12.8(8)
O(6)-S(2)-C(25)-F(4)	-52.2(10)	O(6A)-S(2)-O(6)-S(2A)	138(3)
O(4)-S(2)-C(25)-F(4)	-173.5(9)	O(4A)-S(2)-O(6)-S(2A)	2.2(7)
O(6A)-S(2)-C(25)-F(4)	-32.3(12)	C(25)-S(2)-O(6)-S(2A)	-96.7(8)
O(4A)-S(2)-C(25)-F(4)	-134.4(10)	C(25A)-S(2)-O(6)-S(2A)	-92.9(8)
C(25A)-S(2)-C(25)-F(4)	-63.1(13)	F(6A)-C(25)-F(4)-C(25A)	-82.5(17)
O(5A)-S(2)-C(25)-S(2A)	-128.2(11)	F(6)-C(25)-F(4)-C(25A)	-163.3(17)
O(5)-S(2)-C(25)-S(2A)	-173.0(9)	F(5)-C(25)-F(4)-C(25A)	-42.1(12)
O(6)-S(2)-C(25)-S(2A)	69.5(8)	F(4A)-C(25)-F(4)-C(25A)	123(7)
O(4)-S(2)-C(25)-S(2A)	-51.9(8)	S(2)-C(25)-F(4)-C(25A)	73.6(13)
O(6A)-S(2)-C(25)-S(2A)	89.3(10)	S(2A)-C(25)-F(4)-C(25A)	64.1(12)
O(4A)-S(2)-C(25)-S(2A)	-12.7(8)	C(25A)-C(25)-F(5)-F(6A)	-175.1(19)
C(25A)-S(2)-C(25)-S(2A)	58.6(14)	F(6)-C(25)-F(5)-F(6A)	-5.6(13)
O(5A)-S(2)-O(4)-O(4A)	-169.7(8)	F(4A)-C(25)-F(5)-F(6A)	-129.1(18)
O(5)-S(2)-O(4)-O(4A)	-155.6(6)	F(4)-C(25)-F(5)-F(6A)	-125.7(15)
O(6)-S(2)-O(4)-O(4A)	-16.8(8)	S(2)-C(25)-F(5)-F(6A)	121.0(13)
O(6A)-S(2)-O(4)-O(4A)	-39.3(9)	S(2A)-C(25)-F(5)-F(6A)	119.0(13)

F(6A)-C(25)-F(5)-C(25A)	175.1(19)
F(6)-C(25)-F(5)-C(25A)	169.5(17)
F(4A)-C(25)-F(5)-C(25A)	46.0(15)
F(4)-C(25)-F(5)-C(25A)	49.4(13)
S(2)-C(25)-F(5)-C(25A)	-63.9(12)
S(2A)-C(25)-F(5)-C(25A)	-66.0(12)
C(25A)-C(25)-F(5)-F(5A)	-3.3(12)
F(6A)-C(25)-F(5)-F(5A)	171.8(14)
F(6)-C(25)-F(5)-F(5A)	166.2(8)
F(4A)-C(25)-F(5)-F(5A)	42.7(14)
F(4)-C(25)-F(5)-F(5A)	46.1(11)
S(2)-C(25)-F(5)-F(5A)	-67.2(7)
S(2A)-C(25)-F(5)-F(5A)	-69.2(6)
C(25A)-C(25)-F(6)-F(6A)	38(6)
F(5)-C(25)-F(6)-F(6A)	4.0(10)
F(4A)-C(25)-F(6)-F(6A)	131.1(15)
F(4)-C(25)-F(6)-F(6A)	118.8(13)
S(2)-C(25)-F(6)-F(6A)	-121.4(10)
S(2A)-C(25)-F(6)-F(6A)	-111.0(10)
F(6A)-C(25)-F(6)-C(25A)	-38(6)
F(5)-C(25)-F(6)-C(25A)	-34(5)
F(4A)-C(25)-F(6)-C(25A)	93(6)
F(4)-C(25)-F(6)-C(25A)	81(6)
S(2)-C(25)-F(6)-C(25A)	-159(6)
S(2A)-C(25)-F(6)-C(25A)	-149(6)
O(4A)-O(4)-S(2A)-O(6)	-25.9(11)
S(2)-O(4)-S(2A)-O(6)	155.8(14)
O(5A)-O(4)-S(2A)-O(6)	145.8(9)
S(2)-O(4)-S(2A)-O(4A)	-178.3(10)
O(5A)-O(4)-S(2A)-O(4A)	171.7(7)
O(4A)-O(4)-S(2A)-O(5A)	-171.7(7)
S(2)-O(4)-S(2A)-O(5A)	10.0(9)
O(4A)-O(4)-S(2A)-O(6A)	-62.9(14)
S(2)-O(4)-S(2A)-O(6A)	118.8(16)
O(5A)-O(4)-S(2A)-O(6A)	108.8(12)
O(4A)-O(4)-S(2A)-O(5)	-162.4(6)
S(2)-O(4)-S(2A)-O(5)	19.3(8)
O(5A)-O(4)-S(2A)-O(5)	9.4(5)
O(4A)-O(4)-S(2A)-C(25A)	90.9(7)
S(2)-O(4)-S(2A)-C(25A)	-87.4(9)
O(5A)-O(4)-S(2A)-C(25A)	-97.4(5)
O(4A)-O(4)-S(2A)-C(25)	107.9(6)
S(2)-O(4)-S(2A)-C(25)	-70.4(9)
O(5A)-O(4)-S(2A)-C(25)	-80.3(5)
O(6A)-O(6)-S(2A)-O(4)	-116(2)
S(2)-O(6)-S(2A)-O(4)	-158.1(13)
O(6A)-O(6)-S(2A)-O(4A)	-135(3)
S(2)-O(6)-S(2A)-O(4A)	-177.3(9)
O(6A)-O(6)-S(2A)-O(5A)	15(3)
S(2)-O(6)-S(2A)-O(5A)	-27.4(9)
S(2)-O(6)-S(2A)-O(6A)	-42(3)
O(6A)-O(6)-S(2A)-O(5)	23(3)
S(2)-O(6)-S(2A)-O(5)	-19.6(7)
O(6A)-O(6)-S(2A)-C(25A)	120(3)
S(2)-O(6)-S(2A)-C(25A)	77.4(8)
O(6A)-O(6)-S(2A)-C(25)	111(3)
S(2)-O(6)-S(2A)-C(25)	68.8(8)
O(5A)-O(5)-S(2A)-O(4)	-15.7(9)
S(2)-O(5)-S(2A)-O(4)	-117(2)
O(5A)-O(5)-S(2A)-O(6)	-170.4(9)
S(2)-O(5)-S(2A)-O(6)	89(2)
O(5A)-O(5)-S(2A)-O(4A)	-44.8(14)
S(2)-O(5)-S(2A)-O(4A)	-146(2)
S(2)-O(5)-S(2A)-O(5A)	-101(2)
O(5A)-O(5)-S(2A)-O(6A)	-162.0(10)
S(2)-O(5)-S(2A)-O(6A)	97(2)
O(5A)-O(5)-S(2A)-C(25A)	101.3(9)
S(2)-O(5)-S(2A)-C(25A)	0(2)
O(5A)-O(5)-S(2A)-C(25)	85.2(9)
S(2)-O(5)-S(2A)-C(25)	-16(2)
C(25A)-C(25)-S(2A)-O(4)	-126.4(12)
F(6A)-C(25)-S(2A)-O(4)	-35.8(12)
F(6)-C(25)-S(2A)-O(4)	45.4(9)
F(5)-C(25)-S(2A)-O(4)	-76.8(10)
F(4A)-C(25)-S(2A)-O(4)	164.5(12)
F(4)-C(25)-S(2A)-O(4)	175.4(10)
S(2)-C(25)-S(2A)-O(4)	113.8(9)
C(25A)-C(25)-S(2A)-O(6)	26.4(13)
F(6A)-C(25)-S(2A)-O(6)	117.1(12)
F(6)-C(25)-S(2A)-O(6)	-161.7(8)
F(5)-C(25)-S(2A)-O(6)	76.1(10)
F(4A)-C(25)-S(2A)-O(6)	-42.7(13)
F(4)-C(25)-S(2A)-O(6)	-31.8(11)
S(2)-C(25)-S(2A)-O(6)	-93.3(9)
C(25A)-C(25)-S(2A)-O(4A)	-77.1(13)
F(6A)-C(25)-S(2A)-O(4A)	13.6(13)
F(6)-C(25)-S(2A)-O(4A)	94.7(9)
F(5)-C(25)-S(2A)-O(4A)	-27.4(11)
F(4A)-C(25)-S(2A)-O(4A)	-146.2(12)
F(4)-C(25)-S(2A)-O(4A)	-135.3(10)
S(2)-C(25)-S(2A)-O(4A)	163.2(10)
C(25A)-C(25)-S(2A)-O(5A)	160.8(13)
F(6A)-C(25)-S(2A)-O(5A)	-108.6(12)
F(6)-C(25)-S(2A)-O(5A)	-27.4(9)
F(5)-C(25)-S(2A)-O(5A)	-149.6(10)
F(4A)-C(25)-S(2A)-O(5A)	91.7(13)
F(4)-C(25)-S(2A)-O(5A)	102.5(11)
S(2)-C(25)-S(2A)-O(5A)	41.0(9)
C(25A)-C(25)-S(2A)-O(6A)	48.2(14)
F(6A)-C(25)-S(2A)-O(6A)	138.8(12)
F(6)-C(25)-S(2A)-O(6A)	-140.0(9)
F(5)-C(25)-S(2A)-O(6A)	97.8(10)
F(4A)-C(25)-S(2A)-O(6A)	-20.9(13)
F(4)-C(25)-S(2A)-O(6A)	-10.1(12)
S(2)-C(25)-S(2A)-O(6A)	-71.6(9)
C(25A)-C(25)-S(2A)-O(5)	125.3(12)

F(6A)-C(25)-S(2A)-O(5)	-144.1(11)	F(4)-C(25)-C(25A)-S(2A)	121.1(9)
F(6)-C(25)-S(2A)-O(5)	-62.9(7)	S(2)-C(25)-C(25A)-S(2A)	9.10(17)
F(5)-C(25)-S(2A)-O(5)	174.9(9)	F(6A)-F(5)-C(25A)-C(25)	4.1(16)
F(4A)-C(25)-S(2A)-O(5)	56.2(12)	F(5A)-F(5)-C(25A)-C(25)	176.4(13)
F(4)-C(25)-S(2A)-O(5)	67.1(10)	F(6A)-F(5)-C(25A)-F(4)	-85.1(18)
S(2)-C(25)-S(2A)-O(5)	5.5(7)	F(5A)-F(5)-C(25A)-F(4)	87.3(15)
F(6A)-C(25)-S(2A)-C(25A)	90.6(16)	C(25)-F(5)-C(25A)-F(4)	-89.2(18)
F(6)-C(25)-S(2A)-C(25A)	171.8(17)	F(6A)-F(5)-C(25A)-F(5A)	-172.4(12)
F(5)-C(25)-S(2A)-C(25A)	49.6(12)	C(25)-F(5)-C(25A)-F(5A)	-176.4(13)
F(4A)-C(25)-S(2A)-C(25A)	-69.1(16)	F(5A)-F(5)-C(25A)-F(6A)	172.4(12)
F(4)-C(25)-S(2A)-C(25A)	-58.2(13)	C(25)-F(5)-C(25A)-F(6A)	-4.1(16)
S(2)-C(25)-S(2A)-C(25A)	-119.8(14)	F(6A)-F(5)-C(25A)-F(4A)	-76(2)
F(6A)-C(25)-C(25A)-F(5)	-3.3(13)	F(5A)-F(5)-C(25A)-F(4A)	96(2)
F(6)-C(25)-C(25A)-F(5)	-39(6)	C(25)-F(5)-C(25A)-F(4A)	-80(2)
F(4A)-C(25)-C(25A)-F(5)	-137.1(13)	F(6A)-F(5)-C(25A)-S(2)	83.3(13)
F(4)-C(25)-C(25A)-F(5)	-126.4(11)	F(5A)-F(5)-C(25A)-S(2)	-104.3(9)
S(2)-C(25)-C(25A)-F(5)	121.6(6)	C(25)-F(5)-C(25A)-S(2)	79.3(14)
S(2A)-C(25)-C(25A)-F(5)	112.5(7)	F(6A)-F(5)-C(25A)-S(2A)	89.8(12)
F(6A)-C(25)-C(25A)-F(4)	123.1(12)	F(5A)-F(5)-C(25A)-S(2A)	-97.8(7)
F(6)-C(25)-C(25A)-F(4)	87(6)	C(25)-F(5)-C(25A)-S(2A)	85.7(13)
F(5)-C(25)-C(25A)-F(4)	126.4(11)	C(25)-F(4)-C(25A)-F(5)	95.0(19)
F(4A)-C(25)-C(25A)-F(4)	-10.6(19)	C(25)-F(4)-C(25A)-F(5A)	162.7(15)
S(2)-C(25)-C(25A)-F(4)	-112.0(9)	C(25)-F(4)-C(25A)-F(6A)	48.4(12)
S(2A)-C(25)-C(25A)-F(4)	-121.1(9)	C(25)-F(4)-C(25A)-F(4A)	-57(8)
F(6A)-C(25)-C(25A)-F(5A)	7(5)	C(25)-F(4)-C(25A)-S(2)	-74.6(12)
F(6)-C(25)-C(25A)-F(5A)	-29(9)	C(25)-F(4)-C(25A)-S(2A)	-79.6(13)
F(5)-C(25)-C(25A)-F(5A)	10(4)	O(5A)-S(2)-C(25A)-C(25)	-7.6(15)
F(4A)-C(25)-C(25A)-F(5A)	-127(4)	O(5)-S(2)-C(25A)-C(25)	-56.8(12)
F(4)-C(25)-C(25A)-F(5A)	-116(4)	O(6)-S(2)-C(25A)-C(25)	-169.5(13)
S(2)-C(25)-C(25A)-F(5A)	132(4)	O(4)-S(2)-C(25A)-C(25)	73.9(12)
S(2A)-C(25)-C(25A)-F(5A)	122(4)	O(6A)-S(2)-C(25A)-C(25)	-152.7(13)
F(6)-C(25)-C(25A)-F(6A)	-36(5)	O(4A)-S(2)-C(25A)-C(25)	111.3(12)
F(5)-C(25)-C(25A)-F(6A)	3.3(13)	O(5A)-S(2)-C(25A)-F(5)	-97.6(15)
F(4A)-C(25)-C(25A)-F(6A)	-133.7(15)	O(5)-S(2)-C(25A)-F(5)	-146.8(12)
F(4)-C(25)-C(25A)-F(6A)	-123.1(12)	O(6)-S(2)-C(25A)-F(5)	100.4(13)
S(2)-C(25)-C(25A)-F(6A)	124.9(9)	O(4)-S(2)-C(25A)-F(5)	-16.2(14)
S(2A)-C(25)-C(25A)-F(6A)	115.8(9)	O(6A)-S(2)-C(25A)-F(5)	117.3(13)
F(6A)-C(25)-C(25A)-F(4A)	133.7(15)	O(4A)-S(2)-C(25A)-F(5)	21.3(13)
F(6)-C(25)-C(25A)-F(4A)	98(6)	C(25)-S(2)-C(25A)-F(5)	-90.1(17)
F(5)-C(25)-C(25A)-F(4A)	137.1(13)	O(5A)-S(2)-C(25A)-F(4)	72.5(13)
F(4)-C(25)-C(25A)-F(4A)	10.6(19)	O(5)-S(2)-C(25A)-F(4)	23.3(11)
S(2)-C(25)-C(25A)-F(4A)	-101.3(11)	O(6)-S(2)-C(25A)-F(4)	-89.4(11)
S(2A)-C(25)-C(25A)-F(4A)	-110.4(11)	O(4)-S(2)-C(25A)-F(4)	154.0(10)
F(6A)-C(25)-C(25A)-S(2)	-124.9(9)	O(6A)-S(2)-C(25A)-F(4)	-72.6(11)
F(6)-C(25)-C(25A)-S(2)	-161(6)	O(4A)-S(2)-C(25A)-F(4)	-168.6(10)
F(5)-C(25)-C(25A)-S(2)	-121.6(6)	C(25)-S(2)-C(25A)-F(4)	80.1(15)
F(4A)-C(25)-C(25A)-S(2)	101.3(11)	O(5A)-S(2)-C(25A)-F(5A)	-172.1(9)
F(4)-C(25)-C(25A)-S(2)	112.0(9)	O(5)-S(2)-C(25A)-F(5A)	138.7(7)
S(2A)-C(25)-C(25A)-S(2)	-9.10(17)	O(6)-S(2)-C(25A)-F(5A)	26.0(8)
F(6A)-C(25)-C(25A)-S(2A)	-115.8(9)	O(4)-S(2)-C(25A)-F(5A)	-90.6(7)
F(6)-C(25)-C(25A)-S(2A)	-151(6)	O(6A)-S(2)-C(25A)-F(5A)	42.8(9)
F(5)-C(25)-C(25A)-S(2A)	-112.5(7)	O(4A)-S(2)-C(25A)-F(5A)	-53.2(8)
F(4A)-C(25)-C(25A)-S(2A)	110.4(11)	C(25)-S(2)-C(25A)-F(5A)	-164.5(16)

O(5A)-S(2)-C(25A)-F(6A)	-52.9(12)	C(25)-S(2A)-C(25A)-F(4)	80.7(16)
O(5)-S(2)-C(25A)-F(6A)	-102.1(9)	P(1)-S(2A)-C(25A)-F(4B)	11(2)
O(6)-S(2)-C(25A)-F(6A)	145.2(10)	F(1B)-S(2A)-C(25A)-F(4B)	139.4(11)
O(4)-S(2)-C(25A)-F(6A)	28.6(10)	F(3B)-S(2A)-C(25A)-F(4B)	-73.8(12)
O(6A)-S(2)-C(25A)-F(6A)	162.0(10)	O(4A)-S(2A)-C(25A)-F(4B)	-170.1(12)
O(4A)-S(2)-C(25A)-F(6A)	66.0(9)	O(5A)-S(2A)-C(25A)-F(4B)	61.1(12)
C(25)-S(2)-C(25A)-F(6A)	-45.3(12)	O(6A)-S(2A)-C(25A)-F(4B)	-54.9(12)
O(5A)-S(2)-C(25A)-F(4A)	68.6(14)	F(2B)-S(2A)-C(25A)-F(4B)	25.5(11)
O(5)-S(2)-C(25A)-F(4A)	19.5(12)	P(1)-S(2A)-C(25A)-F(5A)	127(2)
O(6)-S(2)-C(25A)-F(4A)	-93.3(12)	F(1B)-S(2A)-C(25A)-F(5A)	-104.7(7)
O(4)-S(2)-C(25A)-F(4A)	150.1(11)	O(4)-S(2A)-C(25A)-F(5A)	-104.7(7)
O(6A)-S(2)-C(25A)-F(4A)	-76.5(13)	F(3B)-S(2A)-C(25A)-F(5A)	42.1(7)
O(4A)-S(2)-C(25A)-F(4A)	-172.4(12)	O(6)-S(2A)-C(25A)-F(5A)	42.1(7)
C(25)-S(2)-C(25A)-F(4A)	76.2(15)	O(4A)-S(2A)-C(25A)-F(5A)	-54.2(8)
O(5A)-S(2)-C(25A)-S(2A)	-131.0(11)	O(5A)-S(2A)-C(25A)-F(5A)	177.0(8)
O(5)-S(2)-C(25A)-S(2A)	179.8(9)	O(6A)-S(2A)-C(25A)-F(5A)	61.0(8)
O(6)-S(2)-C(25A)-S(2A)	67.0(8)	F(2B)-S(2A)-C(25A)-F(5A)	141.4(6)
O(4)-S(2)-C(25A)-S(2A)	-49.6(7)	O(5)-S(2A)-C(25A)-F(5A)	141.4(6)
O(6A)-S(2)-C(25A)-S(2A)	83.9(9)	C(25)-S(2A)-C(25A)-F(5A)	-163.4(16)
O(4A)-S(2)-C(25A)-S(2A)	-12.1(8)	P(1)-S(2A)-C(25A)-F(6A)	-120(2)
C(25)-S(2)-C(25A)-S(2A)	-123.5(14)	F(1B)-S(2A)-C(25A)-F(6A)	8.4(10)
O(4)-S(2A)-C(25A)-C(25)	58.7(13)	O(4)-S(2A)-C(25A)-F(6A)	8.4(10)
O(6)-S(2A)-C(25A)-C(25)	-154.5(13)	F(3B)-S(2A)-C(25A)-F(6A)	155.3(10)
O(4A)-S(2A)-C(25A)-C(25)	109.2(13)	O(6)-S(2A)-C(25A)-F(6A)	155.3(10)
O(5A)-S(2A)-C(25A)-C(25)	-19.6(13)	O(4A)-S(2A)-C(25A)-F(6A)	59.0(10)
O(6A)-S(2A)-C(25A)-C(25)	-135.6(13)	O(5A)-S(2A)-C(25A)-F(6A)	-69.9(10)
O(5)-S(2A)-C(25A)-C(25)	-55.2(12)	O(6A)-S(2A)-C(25A)-F(6A)	174.1(10)
F(1B)-S(2A)-C(25A)-P(1)	129(2)	F(2B)-S(2A)-C(25A)-F(6A)	-105.4(9)
F(3B)-S(2A)-C(25A)-P(1)	-85(2)	O(5)-S(2A)-C(25A)-F(6A)	-105.4(9)
O(4A)-S(2A)-C(25A)-P(1)	179(2)	C(25)-S(2A)-C(25A)-F(6A)	-50.3(12)
O(5A)-S(2A)-C(25A)-P(1)	50(2)	P(1)-S(2A)-C(25A)-F(4A)	6(2)
O(6A)-S(2A)-C(25A)-P(1)	-66(2)	F(1B)-S(2A)-C(25A)-F(4A)	134.3(12)
F(2B)-S(2A)-C(25A)-P(1)	15(2)	O(4)-S(2A)-C(25A)-F(4A)	134.3(12)
P(1)-S(2A)-C(25A)-F(5B)	-164(3)	F(3B)-S(2A)-C(25A)-F(4A)	-78.9(13)
F(1B)-S(2A)-C(25A)-F(5B)	-35.9(13)	O(6)-S(2A)-C(25A)-F(4A)	-78.9(13)
F(3B)-S(2A)-C(25A)-F(5B)	110.9(12)	O(4A)-S(2A)-C(25A)-F(4A)	-175.2(13)
O(4A)-S(2A)-C(25A)-F(5B)	14.6(13)	O(5A)-S(2A)-C(25A)-F(4A)	56.0(13)
O(5A)-S(2A)-C(25A)-F(5B)	-114.2(13)	O(6A)-S(2A)-C(25A)-F(4A)	-60.0(13)
O(6A)-S(2A)-C(25A)-F(5B)	129.8(12)	F(2B)-S(2A)-C(25A)-F(4A)	20.4(12)
F(2B)-S(2A)-C(25A)-F(5B)	-149.8(11)	O(5)-S(2A)-C(25A)-F(4A)	20.4(12)
O(4)-S(2A)-C(25A)-F(5)	-35.9(13)	C(25)-S(2A)-C(25A)-F(4A)	75.6(16)
O(6)-S(2A)-C(25A)-F(5)	110.9(12)	O(4)-S(2A)-C(25A)-S(2)	113.7(9)
O(4A)-S(2A)-C(25A)-F(5)	14.6(13)	O(6)-S(2A)-C(25A)-S(2)	-99.4(8)
O(5A)-S(2A)-C(25A)-F(5)	-114.2(13)	O(4A)-S(2A)-C(25A)-S(2)	164.3(10)
O(6A)-S(2A)-C(25A)-F(5)	129.8(12)	O(5A)-S(2A)-C(25A)-S(2)	35.4(8)
O(5)-S(2A)-C(25A)-F(5)	-149.8(11)	O(6A)-S(2A)-C(25A)-S(2)	-80.6(9)
C(25)-S(2A)-C(25A)-F(5)	-94.6(16)	O(5)-S(2A)-C(25A)-S(2)	-0.2(7)
O(4)-S(2A)-C(25A)-F(4)	139.4(11)	C(25)-S(2A)-C(25A)-S(2)	55.0(14)
O(6)-S(2A)-C(25A)-F(4)	-73.8(12)	P(1)-S(2A)-C(25A)-F(6B)	-75(2)
O(4A)-S(2A)-C(25A)-F(4)	-170.1(12)	F(1B)-S(2A)-C(25A)-F(6B)	53.8(6)
O(5A)-S(2A)-C(25A)-F(4)	61.1(12)	F(3B)-S(2A)-C(25A)-F(6B)	-159.4(6)
O(6A)-S(2A)-C(25A)-F(4)	-54.9(12)	O(4A)-S(2A)-C(25A)-F(6B)	104.3(6)
O(5)-S(2A)-C(25A)-F(4)	25.5(11)	O(5A)-S(2A)-C(25A)-F(6B)	-24.5(7)

O(6A)-S(2A)-C(25A)-F(6B)	-140.5(6)	C(25A)-S(2)-O(5A)-O(5)	-97.0(7)
F(2B)-S(2A)-C(25A)-F(6B)	-60.1(4)	O(5)-S(2)-O(5A)-S(2A)	-150.8(11)
S(2)-O(4)-O(4A)-S(2A)	0.4(2)	O(6)-S(2)-O(5A)-S(2A)	-109(2)
O(5A)-O(4)-O(4A)-S(2A)	-9.0(8)	O(4)-S(2)-O(5A)-S(2A)	11.4(10)
S(2A)-O(4)-O(4A)-S(2)	-0.4(2)	O(6A)-S(2)-O(5A)-S(2A)	-115.7(14)
O(5A)-O(4)-O(4A)-S(2)	-9.4(7)	O(4A)-S(2)-O(5A)-S(2A)	4.4(9)
P(1)-S(2A)-O(4A)-F(1B)	-106.5(8)	C(25)-S(2)-O(5A)-S(2A)	109.5(11)
F(3B)-S(2A)-O(4A)-F(1B)	164.4(7)	C(25A)-S(2)-O(5A)-S(2A)	112.2(11)
O(5A)-S(2A)-O(4A)-F(1B)	9.4(8)	O(5)-S(2)-O(5A)-O(4)	-162.2(8)
O(6A)-S(2A)-O(4A)-F(1B)	148.0(7)	O(6)-S(2)-O(5A)-O(4)	-120.2(16)
F(2B)-S(2A)-O(4A)-F(1B)	38.2(13)	O(6A)-S(2)-O(5A)-O(4)	-127.1(9)
C(25A)-S(2A)-O(4A)-F(1B)	-106.7(6)	O(4A)-S(2)-O(5A)-O(4)	-7.0(5)
O(6)-S(2A)-O(4A)-O(4)	164.4(7)	C(25)-S(2)-O(5A)-O(4)	98.2(4)
O(5A)-S(2A)-O(4A)-O(4)	9.4(8)	C(25A)-S(2)-O(5A)-O(4)	100.8(5)
O(6A)-S(2A)-O(4A)-O(4)	148.0(7)	P(1)-S(2A)-O(5A)-F(2B)	-74.0(9)
O(5)-S(2A)-O(4A)-O(4)	38.2(13)	F(1B)-S(2A)-O(5A)-F(2B)	164.6(9)
C(25A)-S(2A)-O(4A)-O(4)	-106.7(6)	F(3B)-S(2A)-O(5A)-F(2B)	13.3(13)
C(25)-S(2A)-O(4A)-O(4)	-86.8(7)	O(4A)-S(2A)-O(5A)-F(2B)	157.3(7)
O(4)-S(2A)-O(4A)-S(2)	6(3)	O(6A)-S(2A)-O(5A)-F(2B)	19.3(11)
O(6)-S(2A)-O(4A)-S(2)	170(3)	C(25A)-S(2A)-O(5A)-F(2B)	-85.1(8)
O(5A)-S(2A)-O(4A)-S(2)	15(3)	O(4)-S(2A)-O(5A)-O(5)	164.6(9)
O(6A)-S(2A)-O(4A)-S(2)	154(3)	O(6)-S(2A)-O(5A)-O(5)	13.3(13)
O(5)-S(2A)-O(4A)-S(2)	44(3)	O(4A)-S(2A)-O(5A)-O(5)	157.3(7)
C(25A)-S(2A)-O(4A)-S(2)	-101(3)	O(6A)-S(2A)-O(5A)-O(5)	19.3(11)
C(25)-S(2A)-O(4A)-S(2)	-81(3)	C(25A)-S(2A)-O(5A)-O(5)	-85.1(8)
F(1B)-S(2A)-O(4A)-P(1)	106.5(8)	C(25)-S(2A)-O(5A)-O(5)	-91.6(8)
F(3B)-S(2A)-O(4A)-P(1)	-89.2(8)	O(4)-S(2A)-O(5A)-S(2)	-166.8(12)
O(5A)-S(2A)-O(4A)-P(1)	115.9(9)	O(6)-S(2A)-O(5A)-S(2)	41.9(12)
O(6A)-S(2A)-O(4A)-P(1)	-105.6(9)	O(4A)-S(2A)-O(5A)-S(2)	-174.1(12)
F(2B)-S(2A)-O(4A)-P(1)	144.7(14)	O(6A)-S(2A)-O(5A)-S(2)	47.9(11)
C(25A)-S(2A)-O(4A)-P(1)	-0.3(6)	O(5)-S(2A)-O(5A)-S(2)	28.6(11)
O(5A)-S(2)-O(4A)-O(4)	10.9(8)	C(25A)-S(2A)-O(5A)-S(2)	-56.4(10)
O(5)-S(2)-O(4A)-O(4)	47.3(12)	C(25)-S(2A)-O(5A)-S(2)	-62.9(10)
O(6)-S(2)-O(4A)-O(4)	164.7(7)	O(6)-S(2A)-O(5A)-O(4)	-151.3(9)
O(6A)-S(2)-O(4A)-O(4)	149.6(7)	O(4A)-S(2A)-O(5A)-O(4)	-7.3(6)
C(25)-S(2)-O(4A)-O(4)	-90.7(7)	O(6A)-S(2A)-O(5A)-O(4)	-145.3(7)
C(25A)-S(2)-O(4A)-O(4)	-110.1(6)	O(5)-S(2A)-O(5A)-O(4)	-164.6(9)
O(5A)-S(2)-O(4A)-S(2A)	-163(3)	C(25A)-S(2A)-O(5A)-O(4)	110.4(4)
O(5)-S(2)-O(4A)-S(2A)	-127(3)	C(25)-S(2A)-O(5A)-O(4)	103.9(4)
O(6)-S(2)-O(4A)-S(2A)	-9(3)	P(1)-S(2A)-O(5A)-F(1B)	121.5(6)
O(4)-S(2)-O(4A)-S(2A)	-174(3)	F(3B)-S(2A)-O(5A)-F(1B)	-151.3(9)
O(6A)-S(2)-O(4A)-S(2A)	-25(3)	O(4A)-S(2A)-O(5A)-F(1B)	-7.3(6)
C(25)-S(2)-O(4A)-S(2A)	95(3)	O(6A)-S(2A)-O(5A)-F(1B)	-145.3(7)
C(25A)-S(2)-O(4A)-S(2A)	76(3)	F(2B)-S(2A)-O(5A)-F(1B)	-164.6(9)
S(2A)-O(5)-O(5A)-S(2)	-5.7(2)	C(25A)-S(2A)-O(5A)-F(1B)	110.4(4)
S(2)-O(5)-O(5A)-S(2A)	5.7(2)	F(1B)-S(2A)-O(5A)-P(1)	-121.5(6)
S(2)-O(5)-O(5A)-O(4)	21.8(9)	F(3B)-S(2A)-O(5A)-P(1)	87.3(10)
S(2A)-O(5)-O(5A)-O(4)	16.2(8)	O(4A)-S(2A)-O(5A)-P(1)	-128.7(7)
O(6)-S(2)-O(5A)-O(5)	42(2)	O(6A)-S(2A)-O(5A)-P(1)	93.3(8)
O(4)-S(2)-O(5A)-O(5)	162.2(8)	F(2B)-S(2A)-O(5A)-P(1)	74.0(9)
O(6A)-S(2)-O(5A)-O(5)	35.1(13)	C(25A)-S(2A)-O(5A)-P(1)	-11.1(5)
O(4A)-S(2)-O(5A)-O(5)	155.2(6)	O(4A)-O(4)-O(5A)-O(5)	-11.9(19)
C(25)-S(2)-O(5A)-O(5)	-99.7(7)	S(2A)-O(4)-O(5A)-O(5)	-22.1(12)

S(2)-O(4)-O(5A)-O(5)	-25.2(11)	F(5A)-C(25A)-F(4A)-P(1)	-118.8(12)
O(4A)-O(4)-O(5A)-S(2)	13.3(10)	F(6A)-C(25A)-F(4A)-P(1)	118.6(14)
S(2A)-O(4)-O(5A)-S(2)	3.1(3)	S(2A)-C(25A)-F(4A)-P(1)	-1.6(6)
O(4A)-O(4)-O(5A)-S(2A)	10.2(9)	F(6B)-C(25A)-F(4A)-P(1)	87.8(8)
S(2)-O(4)-O(5A)-S(2A)	-3.1(3)	F(6A)-C(25)-F(4A)-C(25A)	-83(2)
S(2A)-O(6)-O(6A)-S(2)	-9.6(5)	F(6)-C(25)-F(4A)-C(25A)	-163.9(16)
S(2)-O(6)-O(6A)-S(2A)	9.6(5)	F(5)-C(25)-F(4A)-C(25A)	-37.4(13)
O(5A)-S(2)-O(6A)-O(6)	171(2)	F(4)-C(25)-F(4A)-C(25A)	-53(7)
O(5)-S(2)-O(6A)-O(6)	-165(2)	S(2)-C(25)-F(4A)-C(25A)	79.3(12)
O(4)-S(2)-O(6A)-O(6)	67(3)	S(2A)-C(25)-F(4A)-C(25A)	71.2(12)
O(4A)-S(2)-O(6A)-O(6)	44(2)	F(6A)-F(5)-F(5A)-C(25A)	15(2)
C(25)-S(2)-O(6A)-O(6)	-62(3)	C(25)-F(5)-F(5A)-C(25A)	1.7(7)
C(25A)-S(2)-O(6A)-O(6)	-51(3)	P(1)-C(25A)-F(5A)-F(5B)	122.1(11)
O(5A)-S(2)-O(6A)-S(2A)	132.5(13)	F(4B)-C(25A)-F(5A)-F(5B)	-124.7(11)
O(5)-S(2)-O(6A)-S(2A)	156.2(8)	F(6A)-C(25A)-F(5A)-F(5B)	-5.3(8)
O(6)-S(2)-O(6A)-S(2A)	-39(3)	F(4A)-C(25A)-F(5A)-F(5B)	-133.3(14)
O(4)-S(2)-O(6A)-S(2A)	28.5(8)	S(2A)-C(25A)-F(5A)-F(5B)	108.2(7)
O(4A)-S(2)-O(6A)-S(2A)	5.2(7)	F(6B)-C(25A)-F(5A)-F(5B)	-21.5(13)
C(25)-S(2)-O(6A)-S(2A)	-100.5(9)	C(25)-C(25A)-F(5A)-F(5)	-11(4)
C(25A)-S(2)-O(6A)-S(2A)	-90.1(8)	F(4)-C(25A)-F(5A)-F(5)	-124.7(11)
P(1)-S(2A)-O(6A)-F(3B)	-74(3)	F(6A)-C(25A)-F(5A)-F(5)	-5.3(8)
F(1B)-S(2A)-O(6A)-F(3B)	95(3)	F(4A)-C(25A)-F(5A)-F(5)	-133.3(14)
O(4A)-S(2A)-O(6A)-F(3B)	49(3)	S(2)-C(25A)-F(5A)-F(5)	115.6(7)
O(5A)-S(2A)-O(6A)-F(3B)	-168(2)	S(2A)-C(25A)-F(5A)-F(5)	108.2(7)
F(2B)-S(2A)-O(6A)-F(3B)	-157(3)	F(5B)-C(25A)-F(5A)-P(1)	-122.1(11)
C(25A)-S(2A)-O(6A)-F(3B)	-61(3)	F(4B)-C(25A)-F(5A)-P(1)	113.2(14)
O(4)-S(2A)-O(6A)-O(6)	95(3)	F(6A)-C(25A)-F(5A)-P(1)	-127.4(12)
O(4A)-S(2A)-O(6A)-O(6)	49(3)	F(4A)-C(25A)-F(5A)-P(1)	104.6(16)
O(5A)-S(2A)-O(6A)-O(6)	-168(2)	S(2A)-C(25A)-F(5A)-P(1)	-13.9(6)
O(5)-S(2A)-O(6A)-O(6)	-157(3)	F(6B)-C(25A)-F(5A)-P(1)	-143.5(19)
C(25A)-S(2A)-O(6A)-O(6)	-61(3)	C(25A)-F(5)-F(6A)-C(25)	-2.4(9)
C(25)-S(2A)-O(6A)-O(6)	-76(3)	F(5A)-F(5)-F(6A)-C(25)	-16(3)
O(4)-S(2A)-O(6A)-S(2)	-127.6(14)	F(5A)-F(5)-F(6A)-C(25A)	-14(2)
O(6)-S(2A)-O(6A)-S(2)	138(3)	C(25)-F(5)-F(6A)-C(25A)	2.4(9)
O(4A)-S(2A)-O(6A)-S(2)	-173.0(10)	C(25A)-F(5)-F(6A)-F(6)	5(2)
O(5A)-S(2A)-O(6A)-S(2)	-30.6(8)	F(5A)-F(5)-F(6A)-F(6)	-8(4)
O(5)-S(2A)-O(6A)-S(2)	-19.4(7)	C(25)-F(5)-F(6A)-F(6)	7.8(18)
C(25A)-S(2A)-O(6A)-S(2)	76.7(7)	C(25A)-C(25)-F(6A)-F(5)	4.0(16)
C(25)-S(2A)-O(6A)-S(2)	62.0(8)	F(6)-C(25)-F(6A)-F(5)	174.6(13)
F(1B)-S(2A)-O(6A)-P(1)	168.9(13)	F(4A)-C(25)-F(6A)-F(5)	78(2)
F(3B)-S(2A)-O(6A)-P(1)	74(3)	F(4)-C(25)-F(6A)-F(5)	70.7(18)
O(4A)-S(2A)-O(6A)-P(1)	123.4(7)	S(2)-C(25)-F(6A)-F(5)	-80.9(14)
O(5A)-S(2A)-O(6A)-P(1)	-94.1(8)	S(2A)-C(25)-F(6A)-F(5)	-74.5(12)
F(2B)-S(2A)-O(6A)-P(1)	-82.9(6)	F(6)-C(25)-F(6A)-C(25A)	170.5(14)
C(25A)-S(2A)-O(6A)-P(1)	13.2(5)	F(5)-C(25)-F(6A)-C(25A)	-4.0(16)
F(5)-C(25A)-F(4A)-C(25)	88(2)	F(4A)-C(25)-F(6A)-C(25A)	74(2)
F(4)-C(25A)-F(4A)-C(25)	122(8)	F(4)-C(25)-F(6A)-C(25A)	66.7(15)
F(5A)-C(25A)-F(4A)-C(25)	163.8(16)	S(2)-C(25)-F(6A)-C(25A)	-84.9(15)
F(6A)-C(25A)-F(4A)-C(25)	41.2(13)	S(2A)-C(25)-F(6A)-C(25A)	-78.5(13)
S(2)-C(25A)-F(4A)-C(25)	-75.2(12)	C(25A)-C(25)-F(6A)-F(6)	-170.5(14)
S(2A)-C(25A)-F(4A)-C(25)	-79.0(13)	F(5)-C(25)-F(6A)-F(6)	-174.6(13)
F(5B)-C(25A)-F(4A)-P(1)	165(2)	F(4A)-C(25)-F(6A)-F(6)	-96.6(19)
F(4B)-C(25A)-F(4A)-P(1)	-161(8)	F(4)-C(25)-F(6A)-F(6)	-103.9(14)

S(2)-C(25)-F(6A)-F(6)	104.5(11)
S(2A)-C(25)-F(6A)-F(6)	110.9(9)
P(1)-C(25A)-F(6A)-F(5B)	-120.9(13)
F(4B)-C(25A)-F(6A)-F(5B)	119.4(15)
F(5A)-C(25A)-F(6A)-F(5B)	7.2(11)
F(4A)-C(25A)-F(6A)-F(5B)	132.8(17)
S(2A)-C(25A)-F(6A)-F(5B)	-106.0(10)
F(6B)-C(25A)-F(6A)-F(5B)	176.7(15)
C(25)-C(25A)-F(6A)-F(5)	-175.1(19)
F(4)-C(25A)-F(6A)-F(5)	119.4(15)
F(5A)-C(25A)-F(6A)-F(5)	7.2(11)
F(4A)-C(25A)-F(6A)-F(5)	132.8(17)
S(2)-C(25A)-F(6A)-F(5)	-117.1(10)
S(2A)-C(25A)-F(6A)-F(5)	-106.0(10)
F(5)-C(25A)-F(6A)-C(25)	175.1(19)
F(4)-C(25A)-F(6A)-C(25)	-65.5(15)
F(5A)-C(25A)-F(6A)-C(25)	-177.8(16)
F(4A)-C(25A)-F(6A)-C(25)	-52.1(17)
S(2)-C(25A)-F(6A)-C(25)	57.9(13)
S(2A)-C(25A)-F(6A)-C(25)	69.0(13)
P(1)-C(25A)-F(6A)-F(6B)	62.4(12)
F(5B)-C(25A)-F(6A)-F(6B)	-176.7(15)
F(4B)-C(25A)-F(6A)-F(6B)	-57.3(12)
F(5A)-C(25A)-F(6A)-F(6B)	-169.6(7)
F(4A)-C(25A)-F(6A)-F(6B)	-43.9(15)
S(2A)-C(25A)-F(6A)-F(6B)	77.2(8)
C(25)-C(25A)-F(6A)-F(6)	8.2(12)
F(5)-C(25A)-F(6A)-F(6)	-176.7(15)
F(4)-C(25A)-F(6A)-F(6)	-57.3(12)
F(5A)-C(25A)-F(6A)-F(6)	-169.6(7)
F(4A)-C(25A)-F(6A)-F(6)	-43.9(15)
S(2)-C(25A)-F(6A)-F(6)	66.2(8)
S(2A)-C(25A)-F(6A)-F(6)	77.2(8)
F(5B)-C(25A)-F(6A)-P(1)	120.9(13)
F(4B)-C(25A)-F(6A)-P(1)	-119.7(16)
F(5A)-C(25A)-F(6A)-P(1)	128.0(12)
F(4A)-C(25A)-F(6A)-P(1)	-106.3(18)
S(2A)-C(25A)-F(6A)-P(1)	14.8(7)
F(6B)-C(25A)-F(6A)-P(1)	-62.4(12)
C(25)-F(6)-F(6A)-F(5)	-9(2)
C(25A)-F(6)-F(6A)-F(5)	-4.4(19)
C(25A)-F(6)-F(6A)-C(25)	4.5(7)
C(25)-F(6)-F(6A)-C(25A)	-4.5(7)
F(5B)-C(25A)-P(1)-S(2A)	18(3)
F(4B)-C(25A)-P(1)-S(2A)	-170.3(19)
F(5A)-C(25A)-P(1)-S(2A)	-58(2)
F(6A)-C(25A)-P(1)-S(2A)	65(2)
F(4A)-C(25A)-P(1)-S(2A)	-175(2)
F(6B)-C(25A)-P(1)-S(2A)	105(2)
F(5B)-C(25A)-P(1)-F(3B)	92.6(17)
F(4B)-C(25A)-P(1)-F(3B)	-96.0(13)
F(5A)-C(25A)-P(1)-F(3B)	16.1(14)
F(6A)-C(25A)-P(1)-F(3B)	139.4(11)

F(4A)-C(25A)-P(1)-F(3B)	-100.2(14)
S(2A)-C(25A)-P(1)-F(3B)	74(2)
F(6B)-C(25A)-P(1)-F(3B)	179.7(8)
F(5B)-C(25A)-P(1)-F(4A)	-167(2)
F(4B)-C(25A)-P(1)-F(4A)	4.2(18)
F(5A)-C(25A)-P(1)-F(4A)	116.3(14)
F(6A)-C(25A)-P(1)-F(4A)	-120.4(16)
S(2A)-C(25A)-P(1)-F(4A)	175(2)
F(6B)-C(25A)-P(1)-F(4A)	-80.1(11)
F(5B)-C(25A)-P(1)-F(4B)	-171(2)
F(5A)-C(25A)-P(1)-F(4B)	112.1(13)
F(6A)-C(25A)-P(1)-F(4B)	-124.6(16)
F(4A)-C(25A)-P(1)-F(4B)	-4.2(18)
S(2A)-C(25A)-P(1)-F(4B)	170.3(19)
F(6B)-C(25A)-P(1)-F(4B)	-84.3(10)
F(5B)-C(25A)-P(1)-O(5A)	-86(2)
F(4B)-C(25A)-P(1)-O(5A)	85.7(17)
F(5A)-C(25A)-P(1)-O(5A)	-162.2(12)
F(6A)-C(25A)-P(1)-O(5A)	-39(2)
F(4A)-C(25A)-P(1)-O(5A)	81.5(18)
S(2A)-C(25A)-P(1)-O(5A)	-104(3)
F(6B)-C(25A)-P(1)-O(5A)	1.4(15)
F(5B)-C(25A)-P(1)-O(6A)	109.0(17)
F(4B)-C(25A)-P(1)-O(6A)	-79.6(15)
F(5A)-C(25A)-P(1)-O(6A)	32.5(15)
F(6A)-C(25A)-P(1)-O(6A)	155.9(11)
F(4A)-C(25A)-P(1)-O(6A)	-83.8(16)
S(2A)-C(25A)-P(1)-O(6A)	91(2)
F(6B)-C(25A)-P(1)-O(6A)	-163.9(10)
F(4B)-C(25A)-P(1)-F(5B)	171(2)
F(5A)-C(25A)-P(1)-F(5B)	-76.5(15)
F(6A)-C(25A)-P(1)-F(5B)	46.8(12)
F(4A)-C(25A)-P(1)-F(5B)	167(2)
S(2A)-C(25A)-P(1)-F(5B)	-18(3)
F(6B)-C(25A)-P(1)-F(5B)	87.1(16)
F(5B)-C(25A)-P(1)-F(2B)	-138.9(15)
F(4B)-C(25A)-P(1)-F(2B)	32.5(19)
F(5A)-C(25A)-P(1)-F(2B)	144.6(11)
F(6A)-C(25A)-P(1)-F(2B)	-92.1(16)
F(4A)-C(25A)-P(1)-F(2B)	28.3(19)
S(2A)-C(25A)-P(1)-F(2B)	-157(3)
F(6B)-C(25A)-P(1)-F(2B)	-51.8(14)
F(5B)-C(25A)-P(1)-F(6A)	-46.8(12)
F(4B)-C(25A)-P(1)-F(6A)	124.6(16)
F(5A)-C(25A)-P(1)-F(6A)	-123.3(13)
F(4A)-C(25A)-P(1)-F(6A)	120.4(16)
S(2A)-C(25A)-P(1)-F(6A)	-65(2)
F(6B)-C(25A)-P(1)-F(6A)	40.3(9)
F(5B)-C(25A)-P(1)-F(5A)	76.5(15)
F(4B)-C(25A)-P(1)-F(5A)	-112.1(13)
F(6A)-C(25A)-P(1)-F(5A)	123.3(13)
F(4A)-C(25A)-P(1)-F(5A)	-116.3(14)
S(2A)-C(25A)-P(1)-F(5A)	58(2)

F(6B)-C(25A)-P(1)-F(5A)	163.6(9)	F(1B)-S(2A)-P(1)-F(2B)	107.9(6)
F(5B)-C(25A)-P(1)-F(1B)	-16(2)	F(3B)-S(2A)-P(1)-F(2B)	-100.1(5)
F(4B)-C(25A)-P(1)-F(1B)	155.5(10)	O(4A)-S(2A)-P(1)-F(2B)	164.3(7)
F(5A)-C(25A)-P(1)-F(1B)	-92.4(11)	O(5A)-S(2A)-P(1)-F(2B)	34.1(4)
F(6A)-C(25A)-P(1)-F(1B)	31.0(15)	O(6A)-S(2A)-P(1)-F(2B)	-79.3(6)
F(4A)-C(25A)-P(1)-F(1B)	151.3(12)	C(25A)-S(2A)-P(1)-F(2B)	165(2)
S(2A)-C(25A)-P(1)-F(1B)	-34.2(15)	F(1B)-S(2A)-P(1)-F(6A)	-14.5(12)
F(6B)-C(25A)-P(1)-F(1B)	71.2(9)	F(3B)-S(2A)-P(1)-F(6A)	137.5(9)
F(1B)-S(2A)-P(1)-C(25A)	-57(2)	O(4A)-S(2A)-P(1)-F(6A)	42.0(11)
F(3B)-S(2A)-P(1)-C(25A)	94(2)	O(5A)-S(2A)-P(1)-F(6A)	-88.3(9)
O(4A)-S(2A)-P(1)-C(25A)	-1(2)	O(6A)-S(2A)-P(1)-F(6A)	158.4(9)
O(5A)-S(2A)-P(1)-C(25A)	-131(2)	F(2B)-S(2A)-P(1)-F(6A)	-122.4(8)
O(6A)-S(2A)-P(1)-C(25A)	115(2)	C(25A)-S(2A)-P(1)-F(6A)	43.0(19)
F(2B)-S(2A)-P(1)-C(25A)	-165(2)	F(1B)-S(2A)-P(1)-F(5A)	-95.6(8)
F(1B)-S(2A)-P(1)-F(3B)	-152.0(8)	F(3B)-S(2A)-P(1)-F(5A)	56.3(8)
O(4A)-S(2A)-P(1)-F(3B)	-95.5(8)	O(4A)-S(2A)-P(1)-F(5A)	-39.2(10)
O(5A)-S(2A)-P(1)-F(3B)	134.2(7)	O(5A)-S(2A)-P(1)-F(5A)	-169.4(8)
O(6A)-S(2A)-P(1)-F(3B)	20.9(8)	O(6A)-S(2A)-P(1)-F(5A)	77.2(8)
F(2B)-S(2A)-P(1)-F(3B)	100.1(5)	F(2B)-S(2A)-P(1)-F(5A)	156.5(7)
C(25A)-S(2A)-P(1)-F(3B)	-94(2)	C(25A)-S(2A)-P(1)-F(5A)	-38.1(18)
F(1B)-S(2A)-P(1)-F(4A)	134(3)	F(3B)-S(2A)-P(1)-F(1B)	152.0(8)
F(3B)-S(2A)-P(1)-F(4A)	-74(3)	O(4A)-S(2A)-P(1)-F(1B)	56.5(6)
O(4A)-S(2A)-P(1)-F(4A)	-170(3)	O(5A)-S(2A)-P(1)-F(1B)	-73.8(7)
O(5A)-S(2A)-P(1)-F(4A)	60(3)	O(6A)-S(2A)-P(1)-F(1B)	172.9(9)
O(6A)-S(2A)-P(1)-F(4A)	-54(3)	F(2B)-S(2A)-P(1)-F(1B)	-107.9(6)
F(2B)-S(2A)-P(1)-F(4A)	26(3)	C(25A)-S(2A)-P(1)-F(1B)	57(2)
C(25A)-S(2A)-P(1)-F(4A)	-169(4)	C(25A)-F(4A)-P(1)-S(2A)	173(3)
F(1B)-S(2A)-P(1)-F(4B)	149(3)	C(25A)-F(4A)-P(1)-F(3B)	111.5(12)
F(3B)-S(2A)-P(1)-F(4B)	-59(3)	C(25A)-F(4A)-P(1)-F(4B)	18(8)
O(4A)-S(2A)-P(1)-F(4B)	-155(3)	C(25A)-F(4A)-P(1)-O(5A)	-138.5(11)
O(5A)-S(2A)-P(1)-F(4B)	75(3)	C(25A)-F(4A)-P(1)-O(6A)	129.0(12)
O(6A)-S(2A)-P(1)-F(4B)	-38(3)	C(25A)-F(4A)-P(1)-F(5B)	-6.1(10)
F(2B)-S(2A)-P(1)-F(4B)	41(3)	C(25A)-F(4A)-P(1)-F(2B)	-163.7(11)
C(25A)-S(2A)-P(1)-F(4B)	-154(5)	C(25A)-F(4A)-P(1)-F(6A)	-34.1(10)
F(1B)-S(2A)-P(1)-O(5A)	73.8(7)	C(25A)-F(4A)-P(1)-F(5A)	35.3(9)
F(3B)-S(2A)-P(1)-O(5A)	-134.2(7)	C(25A)-F(4A)-P(1)-F(1B)	-96(3)
O(4A)-S(2A)-P(1)-O(5A)	130.3(8)	F(2B)-O(5A)-P(1)-C(25A)	-114.8(15)
O(6A)-S(2A)-P(1)-O(5A)	-113.4(8)	S(2A)-O(5A)-P(1)-C(25A)	142.1(16)
F(2B)-S(2A)-P(1)-O(5A)	-34.1(4)	F(1B)-O(5A)-P(1)-C(25A)	100.0(15)
C(25A)-S(2A)-P(1)-O(5A)	131(2)	F(2B)-O(5A)-P(1)-S(2A)	103.0(8)
F(1B)-S(2A)-P(1)-O(6A)	-172.9(9)	F(1B)-O(5A)-P(1)-S(2A)	-42.1(5)
F(3B)-S(2A)-P(1)-O(6A)	-20.9(8)	F(2B)-O(5A)-P(1)-F(3B)	66.6(9)
O(4A)-S(2A)-P(1)-O(6A)	-116.4(9)	S(2A)-O(5A)-P(1)-F(3B)	-36.4(6)
O(5A)-S(2A)-P(1)-O(6A)	113.4(8)	F(1B)-O(5A)-P(1)-F(3B)	-78.5(6)
F(2B)-S(2A)-P(1)-O(6A)	79.3(6)	F(2B)-O(5A)-P(1)-F(4A)	-54.9(13)
C(25A)-S(2A)-P(1)-O(6A)	-115(2)	S(2A)-O(5A)-P(1)-F(4A)	-157.9(13)
F(1B)-S(2A)-P(1)-F(5B)	-47.4(12)	F(1B)-O(5A)-P(1)-F(4A)	160.0(12)
F(3B)-S(2A)-P(1)-F(5B)	104.6(9)	F(2B)-O(5A)-P(1)-F(4B)	-59.2(11)
O(4A)-S(2A)-P(1)-F(5B)	9.1(12)	S(2A)-O(5A)-P(1)-F(4B)	-162.3(11)
O(5A)-S(2A)-P(1)-F(5B)	-121.2(10)	F(1B)-O(5A)-P(1)-F(4B)	155.6(9)
O(6A)-S(2A)-P(1)-F(5B)	125.5(10)	F(2B)-O(5A)-P(1)-O(6A)	54.3(8)
F(2B)-S(2A)-P(1)-F(5B)	-155.3(9)	S(2A)-O(5A)-P(1)-O(6A)	-48.7(6)
C(25A)-S(2A)-P(1)-F(5B)	10.1(17)	F(1B)-O(5A)-P(1)-O(6A)	-90.8(6)

F(2B)-O(5A)-P(1)-F(5B)	-154.3(9)	C(25A)-F(6A)-P(1)-F(5B)	-47.7(12)
S(2A)-O(5A)-P(1)-F(5B)	102.6(9)	F(6B)-F(6A)-P(1)-F(5B)	-162.2(15)
F(1B)-O(5A)-P(1)-F(5B)	60.5(8)	F(5B)-F(6A)-P(1)-F(2B)	177.9(10)
S(2A)-O(5A)-P(1)-F(2B)	-103.0(8)	C(25A)-F(6A)-P(1)-F(2B)	130.2(13)
F(1B)-O(5A)-P(1)-F(2B)	-145.2(9)	F(6B)-F(6A)-P(1)-F(2B)	15.7(10)
F(2B)-O(5A)-P(1)-F(6A)	-139.6(8)	F(5B)-F(6A)-P(1)-F(5A)	13.2(9)
S(2A)-O(5A)-P(1)-F(6A)	117.4(8)	C(25A)-F(6A)-P(1)-F(5A)	-34.4(10)
F(1B)-O(5A)-P(1)-F(6A)	75.2(7)	F(6B)-F(6A)-P(1)-F(5A)	-149.0(8)
F(2B)-O(5A)-P(1)-F(5A)	158(3)	F(5B)-F(6A)-P(1)-F(1B)	-107.5(11)
S(2A)-O(5A)-P(1)-F(5A)	55(3)	C(25A)-F(6A)-P(1)-F(1B)	-155.2(12)
F(1B)-O(5A)-P(1)-F(5A)	13(3)	F(6B)-F(6A)-P(1)-F(1B)	90.2(6)
F(2B)-O(5A)-P(1)-F(1B)	145.2(9)	P(1)-O(4A)-F(1B)-S(2A)	-25.6(3)
S(2A)-O(5A)-P(1)-F(1B)	42.1(5)	S(2A)-O(4A)-F(1B)-O(5A)	-9.0(8)
F(3B)-O(6A)-P(1)-C(25A)	-55(3)	P(1)-O(4A)-F(1B)-O(5A)	-34.6(8)
S(2A)-O(6A)-P(1)-C(25A)	-141.9(14)	S(2A)-O(4A)-F(1B)-P(1)	25.6(3)
F(3B)-O(6A)-P(1)-S(2A)	87(3)	P(1)-S(2A)-F(1B)-O(4A)	104.1(9)
S(2A)-O(6A)-P(1)-F(3B)	-87(3)	F(3B)-S(2A)-F(1B)-O(4A)	-25.9(11)
F(3B)-O(6A)-P(1)-F(4A)	-113(3)	O(5A)-S(2A)-F(1B)-O(4A)	-171.7(7)
S(2A)-O(6A)-P(1)-F(4A)	160.0(12)	O(6A)-S(2A)-F(1B)-O(4A)	-62.9(14)
F(3B)-O(6A)-P(1)-F(4B)	-104(3)	F(2B)-S(2A)-F(1B)-O(4A)	-162.4(6)
S(2A)-O(6A)-P(1)-F(4B)	169.6(10)	C(25A)-S(2A)-F(1B)-O(4A)	90.9(7)
F(3B)-O(6A)-P(1)-O(5A)	134(3)	P(1)-S(2A)-F(1B)-O(5A)	-84.2(8)
S(2A)-O(6A)-P(1)-O(5A)	47.1(7)	F(3B)-S(2A)-F(1B)-O(5A)	145.8(9)
F(3B)-O(6A)-P(1)-F(5B)	-16(3)	O(4A)-S(2A)-F(1B)-O(5A)	171.7(7)
S(2A)-O(6A)-P(1)-F(5B)	-102.6(10)	O(6A)-S(2A)-F(1B)-O(5A)	108.8(12)
F(3B)-O(6A)-P(1)-F(2B)	161(3)	F(2B)-S(2A)-F(1B)-O(5A)	9.4(5)
S(2A)-O(6A)-P(1)-F(2B)	74.4(6)	C(25A)-S(2A)-F(1B)-O(5A)	-97.4(5)
F(3B)-O(6A)-P(1)-F(6A)	1(4)	F(3B)-S(2A)-F(1B)-P(1)	-130.0(12)
S(2A)-O(6A)-P(1)-F(6A)	-86(2)	O(4A)-S(2A)-F(1B)-P(1)	-104.1(9)
F(3B)-O(6A)-P(1)-F(5A)	-35(3)	O(5A)-S(2A)-F(1B)-P(1)	84.2(8)
S(2A)-O(6A)-P(1)-F(5A)	-122.0(7)	O(6A)-S(2A)-F(1B)-P(1)	-167.0(15)
F(3B)-O(6A)-P(1)-F(1B)	83(3)	F(2B)-S(2A)-F(1B)-P(1)	93.5(7)
S(2A)-O(6A)-P(1)-F(1B)	-3.9(5)	C(25A)-S(2A)-F(1B)-P(1)	-13.2(6)
F(5B)-F(6A)-P(1)-C(25A)	47.7(12)	F(2B)-O(5A)-F(1B)-O(4A)	-11.9(19)
F(6B)-F(6A)-P(1)-C(25A)	-114.5(14)	S(2A)-O(5A)-F(1B)-O(4A)	10.2(9)
F(5B)-F(6A)-P(1)-S(2A)	-99.7(12)	P(1)-O(5A)-F(1B)-O(4A)	40.9(10)
C(25A)-F(6A)-P(1)-S(2A)	-147.4(14)	F(2B)-O(5A)-F(1B)-S(2A)	-22.1(12)
F(6B)-F(6A)-P(1)-S(2A)	98.0(10)	P(1)-O(5A)-F(1B)-S(2A)	30.7(4)
F(5B)-F(6A)-P(1)-F(3B)	-25.7(18)	F(2B)-O(5A)-F(1B)-P(1)	-52.8(13)
C(25A)-F(6A)-P(1)-F(3B)	-73.4(16)	S(2A)-O(5A)-F(1B)-P(1)	-30.7(4)
F(6B)-F(6A)-P(1)-F(3B)	172.1(12)	F(1B)-O(5A)-F(2B)-S(2A)	16.2(8)
F(5B)-F(6A)-P(1)-F(4A)	92.7(14)	P(1)-O(5A)-F(2B)-S(2A)	-33.3(4)
C(25A)-F(6A)-P(1)-F(4A)	45.0(13)	S(2A)-O(5A)-F(2B)-P(1)	33.3(4)
F(6B)-F(6A)-P(1)-F(4A)	-69.5(10)	F(1B)-O(5A)-F(2B)-P(1)	49.4(11)
F(5B)-F(6A)-P(1)-F(4B)	84.9(12)	P(1)-S(2A)-F(2B)-O(5A)	105.0(9)
C(25A)-F(6A)-P(1)-F(4B)	37.2(11)	F(1B)-S(2A)-F(2B)-O(5A)	-15.7(9)
F(6B)-F(6A)-P(1)-F(4B)	-77.3(8)	F(3B)-S(2A)-F(2B)-O(5A)	-170.4(9)
F(5B)-F(6A)-P(1)-O(5A)	-156.2(11)	O(4A)-S(2A)-F(2B)-O(5A)	-44.8(14)
C(25A)-F(6A)-P(1)-O(5A)	156.1(13)	O(6A)-S(2A)-F(2B)-O(5A)	-162.0(10)
F(6B)-F(6A)-P(1)-O(5A)	41.6(8)	C(25A)-S(2A)-F(2B)-O(5A)	101.3(9)
F(5B)-F(6A)-P(1)-O(6A)	-26(3)	F(1B)-S(2A)-F(2B)-P(1)	-120.7(6)
C(25A)-F(6A)-P(1)-O(6A)	-74(3)	F(3B)-S(2A)-F(2B)-P(1)	84.7(7)
F(6B)-F(6A)-P(1)-O(6A)	172(2)	O(4A)-S(2A)-F(2B)-P(1)	-149.7(12)

O(5A)-S(2A)-F(2B)-P(1)	-105.0(9)	F(2B)-P(1)-F(3B)-S(2A)	63.7(5)
O(6A)-S(2A)-F(2B)-P(1)	93.1(7)	F(6A)-P(1)-F(3B)-S(2A)	-98.2(13)
C(25A)-S(2A)-F(2B)-P(1)	-3.7(5)	F(5A)-P(1)-F(3B)-S(2A)	-133.9(7)
C(25A)-P(1)-F(2B)-O(5A)	108.8(16)	F(1B)-P(1)-F(3B)-S(2A)	-14.8(4)
S(2A)-P(1)-F(2B)-O(5A)	-58.8(8)	F(5B)-C(25A)-F(4B)-P(1)	172(2)
F(3B)-P(1)-F(2B)-O(5A)	-113.4(9)	F(5A)-C(25A)-F(4B)-P(1)	-120.7(12)
F(4A)-P(1)-F(2B)-O(5A)	131.2(12)	F(6A)-C(25A)-F(4B)-P(1)	125.0(15)
F(4B)-P(1)-F(2B)-O(5A)	131.5(10)	F(4A)-C(25A)-F(4B)-P(1)	20(8)
O(6A)-P(1)-F(2B)-O(5A)	-118.9(9)	S(2A)-C(25A)-F(4B)-P(1)	-3.0(6)
F(5B)-P(1)-F(2B)-O(5A)	55.7(17)	F(6B)-C(25A)-F(4B)-P(1)	86.9(9)
F(6A)-P(1)-F(2B)-O(5A)	53.2(11)	S(2A)-P(1)-F(4B)-C(25A)	162(4)
F(5A)-P(1)-F(2B)-O(5A)	-168.8(17)	F(3B)-P(1)-F(4B)-C(25A)	109.8(12)
F(1B)-P(1)-F(2B)-O(5A)	-27.0(8)	F(4A)-P(1)-F(4B)-C(25A)	-159(9)
C(25A)-P(1)-F(2B)-S(2A)	167.6(18)	O(5A)-P(1)-F(4B)-C(25A)	-133.7(13)
F(3B)-P(1)-F(2B)-S(2A)	-54.6(6)	O(6A)-P(1)-F(4B)-C(25A)	128.2(13)
F(4A)-P(1)-F(2B)-S(2A)	-170.0(11)	F(5B)-P(1)-F(4B)-C(25A)	-4.2(11)
F(4B)-P(1)-F(2B)-S(2A)	-169.6(9)	F(2B)-P(1)-F(4B)-C(25A)	-161.0(12)
O(5A)-P(1)-F(2B)-S(2A)	58.8(8)	F(6A)-P(1)-F(4B)-C(25A)	-33.3(11)
O(6A)-P(1)-F(2B)-S(2A)	-60.1(6)	F(5A)-P(1)-F(4B)-C(25A)	39.0(10)
F(5B)-P(1)-F(2B)-S(2A)	114.5(16)	F(1B)-P(1)-F(4B)-C(25A)	-70(2)
F(6A)-P(1)-F(2B)-S(2A)	112.0(9)	F(6B)-F(6A)-F(5B)-C(25A)	5(2)
F(5A)-P(1)-F(2B)-S(2A)	-110.0(17)	P(1)-F(6A)-F(5B)-C(25A)	-23.9(5)
F(1B)-P(1)-F(2B)-S(2A)	31.8(4)	C(25A)-F(6A)-F(5B)-F(5A)	-14(2)
P(1)-O(6A)-F(3B)-S(2A)	-38.4(7)	F(6B)-F(6A)-F(5B)-F(5A)	-8(4)
S(2A)-O(6A)-F(3B)-P(1)	38.4(7)	P(1)-F(6A)-F(5B)-F(5A)	-38(2)
P(1)-S(2A)-F(3B)-O(6A)	105(3)	C(25A)-F(6A)-F(5B)-P(1)	23.9(5)
F(1B)-S(2A)-F(3B)-O(6A)	-116(2)	F(6B)-F(6A)-F(5B)-P(1)	29(2)
O(4A)-S(2A)-F(3B)-O(6A)	-135(3)	P(1)-C(25A)-F(5B)-F(6A)	84.5(17)
O(5A)-S(2A)-F(3B)-O(6A)	15(3)	F(4B)-C(25A)-F(5B)-F(6A)	-85.1(18)
F(2B)-S(2A)-F(3B)-O(6A)	23(3)	F(5A)-C(25A)-F(5B)-F(6A)	-172.4(12)
C(25A)-S(2A)-F(3B)-O(6A)	120(3)	F(4A)-C(25A)-F(5B)-F(6A)	-76(2)
F(1B)-S(2A)-F(3B)-P(1)	138.9(10)	S(2A)-C(25A)-F(5B)-F(6A)	89.8(12)
O(4A)-S(2A)-F(3B)-P(1)	119.8(6)	F(6B)-C(25A)-F(5B)-F(6A)	-2.4(11)
O(5A)-S(2A)-F(3B)-P(1)	-90.3(9)	P(1)-C(25A)-F(5B)-F(5A)	-103.1(15)
O(6A)-S(2A)-F(3B)-P(1)	-105(3)	F(4B)-C(25A)-F(5B)-F(5A)	87.3(15)
F(2B)-S(2A)-F(3B)-P(1)	-82.5(6)	F(6A)-C(25A)-F(5B)-F(5A)	172.4(12)
C(25A)-S(2A)-F(3B)-P(1)	14.5(5)	F(4A)-C(25A)-F(5B)-F(5A)	96(2)
C(25A)-P(1)-F(3B)-O(6A)	135(3)	S(2A)-C(25A)-F(5B)-F(5A)	-97.8(7)
S(2A)-P(1)-F(3B)-O(6A)	-81(3)	F(6B)-C(25A)-F(5B)-F(5A)	170.0(6)
F(4A)-P(1)-F(3B)-O(6A)	74(3)	F(4B)-C(25A)-F(5B)-P(1)	-170(3)
F(4B)-P(1)-F(3B)-O(6A)	84(3)	F(5A)-C(25A)-F(5B)-P(1)	103.1(15)
O(5A)-P(1)-F(3B)-O(6A)	-46(3)	F(6A)-C(25A)-F(5B)-P(1)	-84.5(17)
F(5B)-P(1)-F(3B)-O(6A)	167(2)	F(4A)-C(25A)-F(5B)-P(1)	-161(3)
F(2B)-P(1)-F(3B)-O(6A)	-17(3)	S(2A)-C(25A)-F(5B)-P(1)	5.3(9)
F(6A)-P(1)-F(3B)-O(6A)	-179(2)	F(6B)-C(25A)-F(5B)-P(1)	-86.9(15)
F(5A)-P(1)-F(3B)-O(6A)	145(3)	C(25A)-F(5A)-F(5B)-F(6A)	15(2)
F(1B)-P(1)-F(3B)-O(6A)	-96(3)	P(1)-F(5A)-F(5B)-F(6A)	39(2)
C(25A)-P(1)-F(3B)-S(2A)	-144.0(12)	P(1)-F(5A)-F(5B)-C(25A)	24.2(5)
F(4A)-P(1)-F(3B)-S(2A)	154.7(13)	C(25A)-F(5A)-F(5B)-P(1)	-24.2(5)
F(4B)-P(1)-F(3B)-S(2A)	165.2(10)	C(25A)-P(1)-F(5B)-F(6A)	-92.6(18)
O(5A)-P(1)-F(3B)-S(2A)	35.0(6)	S(2A)-P(1)-F(5B)-F(6A)	98.2(13)
O(6A)-P(1)-F(3B)-S(2A)	81(3)	F(3B)-P(1)-F(5B)-F(6A)	163.4(12)
F(5B)-P(1)-F(3B)-S(2A)	-111.5(8)	F(4A)-P(1)-F(5B)-F(6A)	-82.2(14)

F(4B)-P(1)-F(5B)-F(6A)	-86.3(13)	F(5B)-F(6A)-F(6B)-C(25A)	-4.4(19)
O(5A)-P(1)-F(5B)-F(6A)	31.4(14)	P(1)-F(6A)-F(6B)-C(25A)	23.9(5)
O(6A)-P(1)-F(5B)-F(6A)	168.7(13)	F(5B)-F(6A)-F(6B)-P(1)	-28(2)
F(2B)-P(1)-F(5B)-F(6A)	-4(2)	C(25A)-F(6A)-F(6B)-P(1)	-23.9(5)
F(5A)-P(1)-F(5B)-F(6A)	-161.1(13)	P(1)-C(25A)-F(6B)-F(6A)	-125.9(11)
F(1B)-P(1)-F(5B)-F(6A)	74.4(12)	F(5B)-C(25A)-F(6B)-F(6A)	2.2(10)
S(2A)-P(1)-F(5B)-C(25A)	-169.2(19)	F(4B)-C(25A)-F(6B)-F(6A)	129.2(12)
F(3B)-P(1)-F(5B)-C(25A)	-104.0(18)	F(5A)-C(25A)-F(6B)-F(6A)	21.5(15)
F(4A)-P(1)-F(5B)-C(25A)	10.4(17)	F(4A)-C(25A)-F(6B)-F(6A)	140.8(14)
F(4B)-P(1)-F(5B)-C(25A)	6.3(16)	S(2A)-C(25A)-F(6B)-F(6A)	-110.8(8)
O(5A)-P(1)-F(5B)-C(25A)	123.9(18)	F(5B)-C(25A)-F(6B)-P(1)	128.1(9)
O(6A)-P(1)-F(5B)-C(25A)	-98.8(18)	F(4B)-C(25A)-F(6B)-P(1)	-104.9(11)
F(2B)-P(1)-F(5B)-C(25A)	88(2)	F(5A)-C(25A)-F(6B)-P(1)	147.4(17)
F(6A)-P(1)-F(5B)-C(25A)	92.6(18)	F(6A)-C(25A)-F(6B)-P(1)	125.9(11)
F(5A)-P(1)-F(5B)-C(25A)	-68.5(16)	F(4A)-C(25A)-F(6B)-P(1)	-93.2(13)
F(1B)-P(1)-F(5B)-C(25A)	167.0(17)	S(2A)-C(25A)-F(6B)-P(1)	15.1(5)
C(25A)-P(1)-F(5B)-F(5A)	68.5(16)	F(5A)-C(25A)-F(6B)-F(6A)	21.5(15)
S(2A)-P(1)-F(5B)-F(5A)	-100.7(9)	F(4A)-C(25A)-F(6B)-F(6A)	140.8(14)
F(3B)-P(1)-F(5B)-F(5A)	-35.5(8)	S(2A)-C(25A)-F(6B)-F(6A)	-110.8(8)
F(4A)-P(1)-F(5B)-F(5A)	78.9(10)	F(5B)-C(25A)-F(6B)-P(1)	128.1(9)
F(4B)-P(1)-F(5B)-F(5A)	74.9(8)	F(4B)-C(25A)-F(6B)-P(1)	-104.9(11)
O(5A)-P(1)-F(5B)-F(5A)	-167.5(9)	F(5A)-C(25A)-F(6B)-P(1)	147.4(17)
O(6A)-P(1)-F(5B)-F(5A)	-30.2(10)	F(6A)-C(25A)-F(6B)-P(1)	125.9(11)
F(2B)-P(1)-F(5B)-F(5A)	156.9(16)	F(4A)-C(25A)-F(6B)-P(1)	-93.2(13)
F(6A)-P(1)-F(5B)-F(5A)	161.1(13)	S(2A)-C(25A)-F(6B)-P(1)	15.1(5)
F(1B)-P(1)-F(5B)-F(5A)	-124.5(6)		

Triazolium salt (9b) – "1-cobaltoceniumyl-4-ferrocenyl-3-methyltriazolium bis(hexafluoridophosphate)"

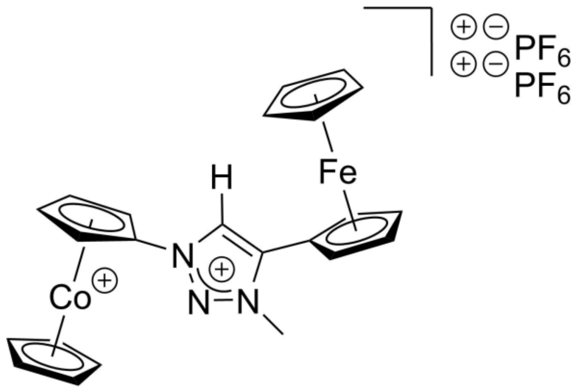

[745.15]

$C_{23}H_{22}N_3CoFe(PF_6)_2$

Analytical data:

IR (ATR [cm^{-1}]): 3156 (ν_{C-H}), 3130 (ν_{C-H}), 1600, 1519, 1434, 1419 ($\nu_{C=C}$), 824 (ν_{P-F}), 555 (ν_{P-F}), 510, 442.

^{1}H-NMR (300 MHz, C_3D_6O, [ppm]): δ 4.38 (s, 5H, Fc-Cp), 4.62 (s, 3H, CH_3 of triazolium), 4.74 (pseudo-t, 2H, J = 1.8 Hz, C3/C4 of substituted Fc-Cp), 5.05 (pseudo-t, 2H, J = 1.8 Hz, C2/C5 of substituted Fc-Cp), 6.15 (s, 5H, Cc-Cp), 6.25 (pseudo-t, 2H, J = 2.3 Hz, C3/C4 of substituted Cc-Cp), 6.87 (pseudo-t, 2H, J = 2.3 Hz, C2/C5 of substituted Cc-Cp), 9.49 (s, 1H, CH of triazole).

^{13}C-NMR (75 MHz, C_3D_6O, [ppm]): δ 40.8 (CH_3 of triazole), 66.0 (quart. carbon of substituted Fc-Cp), 70.2 (C3/C4 of substituted Fc-Cp), 71.3 (Fc-Cp), 72.7 (C2/C5 of substituted Fc-Cp), 79.6 (C3/C4 of substituted Cc-Cp), 85.0 (C2/C5 of substituted Cc-Cp), 88.8 (Cc-Cp), 105.2 (quart. carbon of substituted Cc-Cp), 128.7 (CH of triazolium), 147.0 (quart. carbon of triazolium).

MS (ESI pos, [m/z]): 600.01 ($[M(PF_6)]^+$).

Melting point [°C]: 247 (dec.).

Spectra

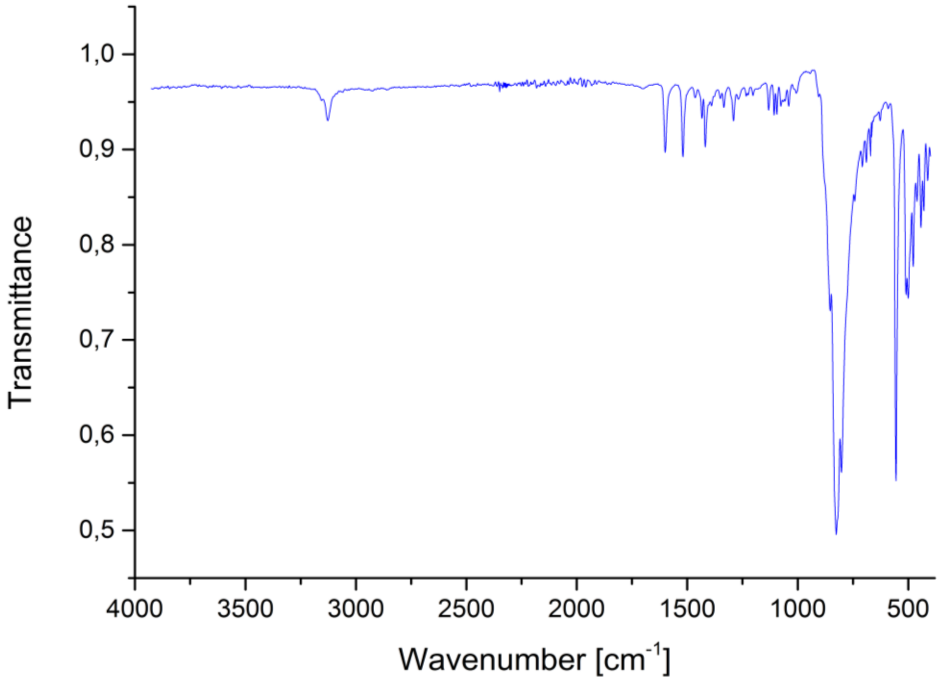

Figure S30. IR-spectrum (ATR, [cm^{-1}]) of "1-cobaltoceniumyl-4-ferrocenyl-3-methyltriazolium bis(hexafluoridophosphate)".

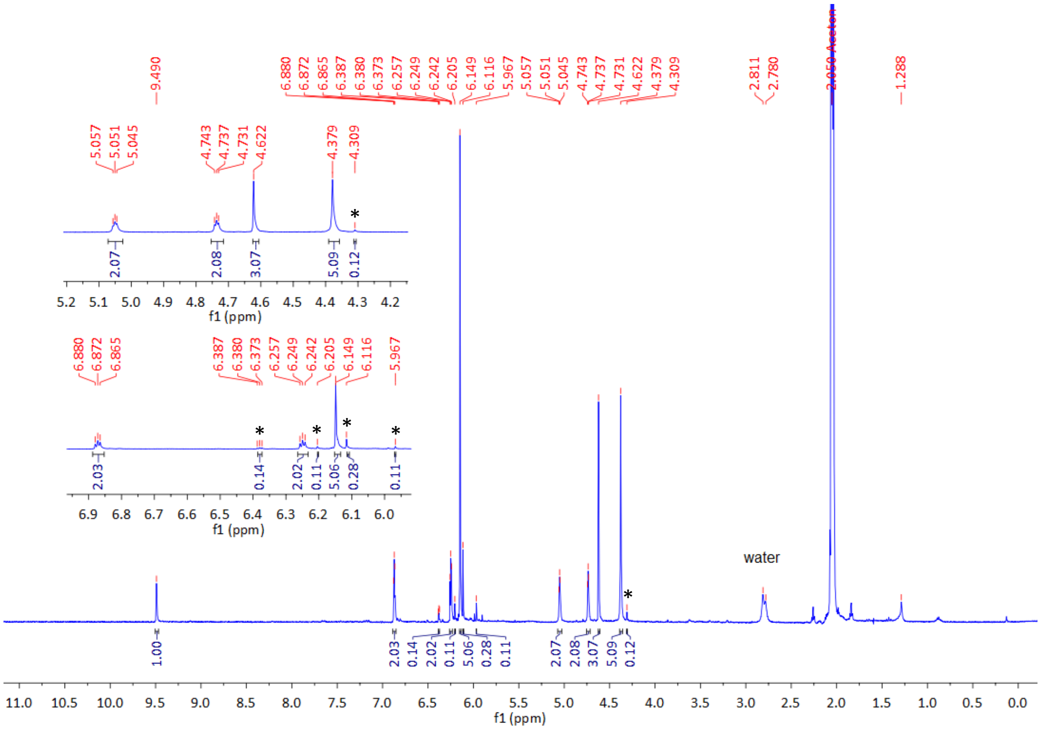

Figure S31. ^{1}H-NMR (300 MHz, C_3D_6O, [ppm]) of "1-cobaltoceniumyl-4-ferrocenyl-3-methyltriazolium bis(hexafluoridophosphate)" (impurity marked, < 2%).

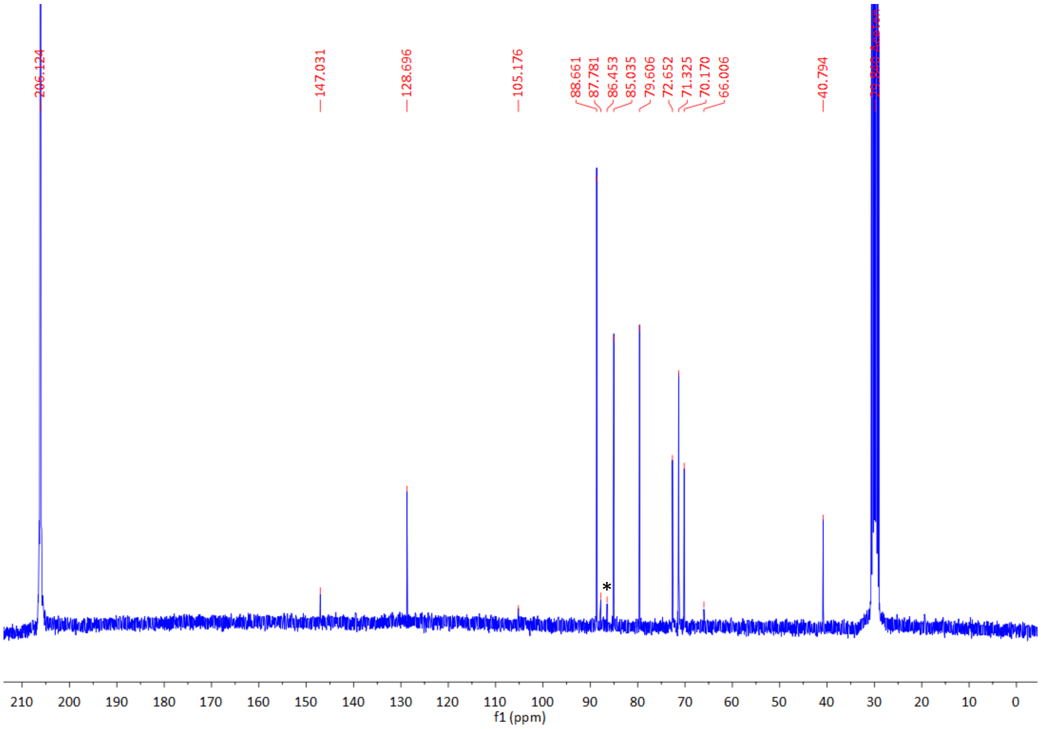

Figure S32. ^{13}C-NMR (75 MHz, C_3D_6O, [ppm]) of "1-cobaltoceniumyl-4-ferrocenyl-3-methyltriazolium bis(hexafluoridophosphate)" (impurity marked, < 2%).

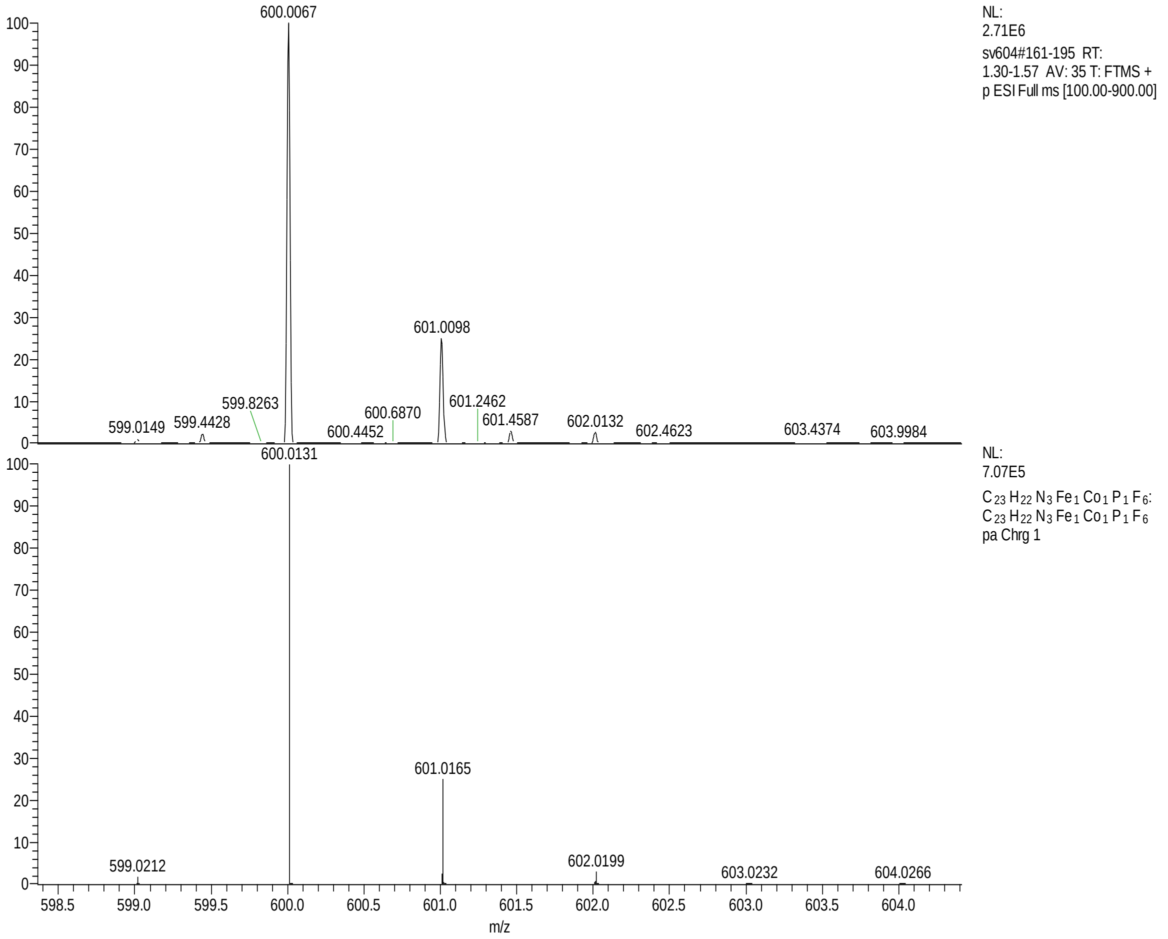

Figure S33. MS (MALDI pos, [m/z]; *top*: experimental, *bottom*: simulated) of "1-cobaltoceniumyl-4-ferrocenyl-3-methyltriazolium bis(hexafluoridophosphate)".

Triazolium salt (10a) – "1,4-dicobaltoceniumyl-3-methyltriazolium bis(hexafluorido-phosphate) triflate"

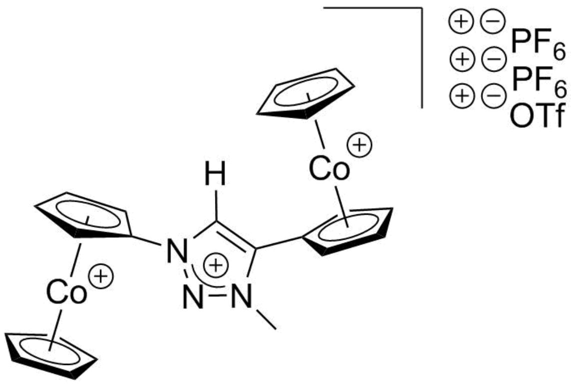

[897.30]

$C_{23}H_{22}N_3Co_2(PF_6)_2(CF_3SO_3)$

Analytical data:

IR (ATR [cm^{-1}]): 3115 (ν_{C-H}), 1525, 1466, 1417 ($\nu_{C=C}$), 1254 (ν_{SO3}), 1224 (ν_{CF3}), 1151 (ν_{CF3}), 1028 (ν_{SO3}), 835 (ν_{P-F}), 634, 572 (ν_{P-F}), 516, 500, 473, 442.

^{1}H-NMR (300 MHz, C_3D_6O, [ppm]): δ 4.68 (s, 3H, CH_3 of triazolium), 6.18 (s, 5H, 4-Cc-Cp), 6.23 (s, 5H, 1-Cc-Cp), 6.28 (pseudo-t, 2H, *J* = 2.3 Hz, C3/C4 of substituted 1-Cc-Cp), 6.34 (pseudo-t, 2H, *J* = 2.1 Hz, C3/C4 of substituted 4-Cc-Cp), 6.70 (pseudo-t, 2H, *J* = 2.1 Hz, C2/C5 of substituted 4-Cc-Cp), 6.93 (pseudo-t, 2H, J = 2.3 Hz, C2/C5 of substituted 1-Cc-Cp), 10.14 (s, 1H, CH of triazolium).

^{13}C-NMR (75 MHz, C_3D_6O, [ppm]): δ 41.5 (CH_3 of triazolium), 79.9 (C3/C4 of substituted 4-Cc-Cp), 85.0 (C3/C4 of substituted 1-Cc-Cp), 85.9 (quart. carbon of substituted 4-Cc-Cp), 86.5 (C2/C5 of substituted 4-Cc-Cp), 87.4 (C2/C5 of substituted 1-Cc-Cp), 88.5 (4-Cc-Cp), 89.0 (1-Cc-Cp), 105.0 (quart. carbon of substituted 1-Cc-Cp), 119.9 (CF_3 of triflate), 124.1 (CF_3 of triflate), 132.6 (CH of triazolium), 138.8 (quart. carbon of triazolium).

MS (MALDI pos, [m/z]): 755.96 ($[M(CF_3SO_3)_2]^+$).

Melting point [°C]: 240.

Spectra

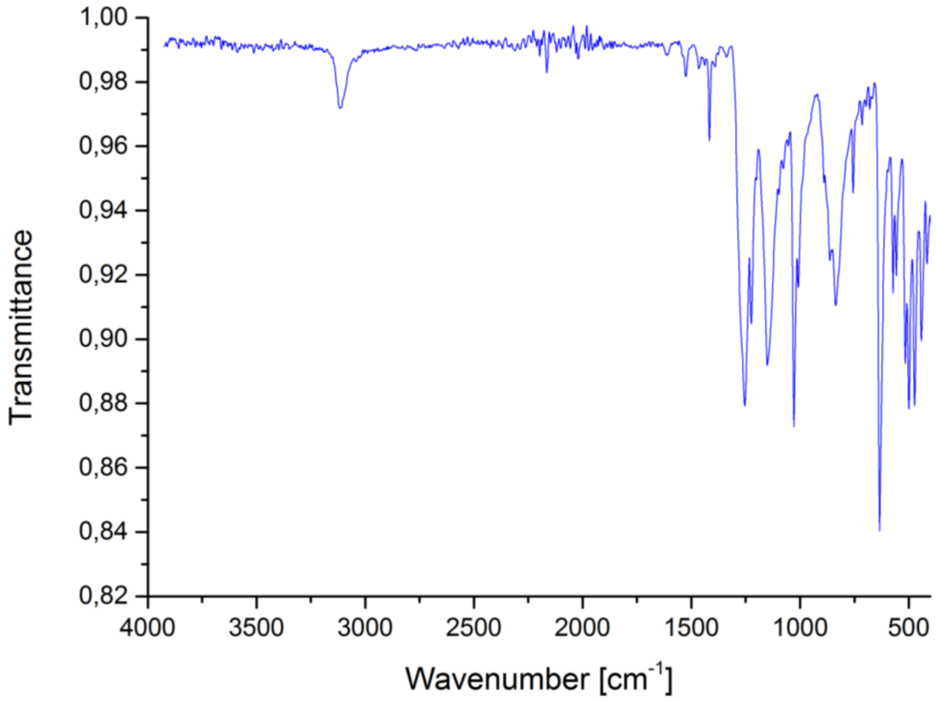

Figure S34. IR-spectrum (ATR, [cm^{-1}]) of “1,4-dicobaltoceniumyl-3-methyltriazolium bis(hexafluoridophosphate) triflate“.

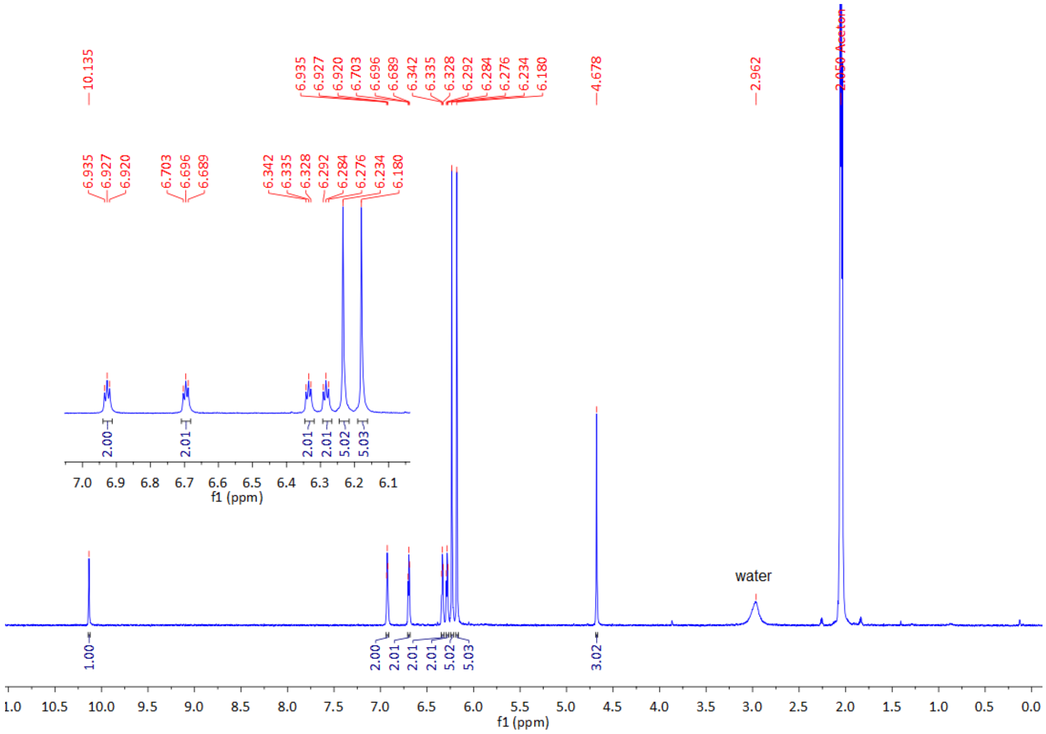

Figure S35. ^{1}H-NMR (300 MHz, C_3D_6O, [ppm]) of "1,4-dicobaltoceniumyl-3-methyltriazolium bis(hexafluoridophosphate) triflate".

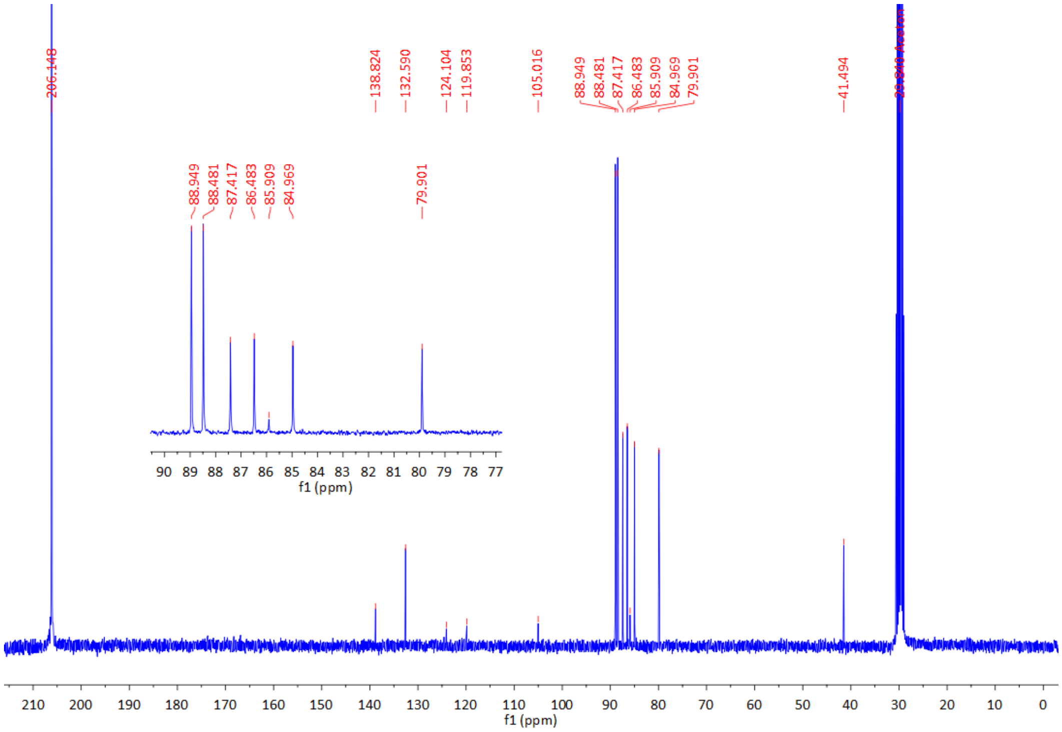

Figure S36. ^{13}C-NMR (75 MHz, C_3D_6O, [ppm]) of "1,4-dicobaltoceniumyl-3-methyltriazolium bis(hexafluoridophosphate) triflate".

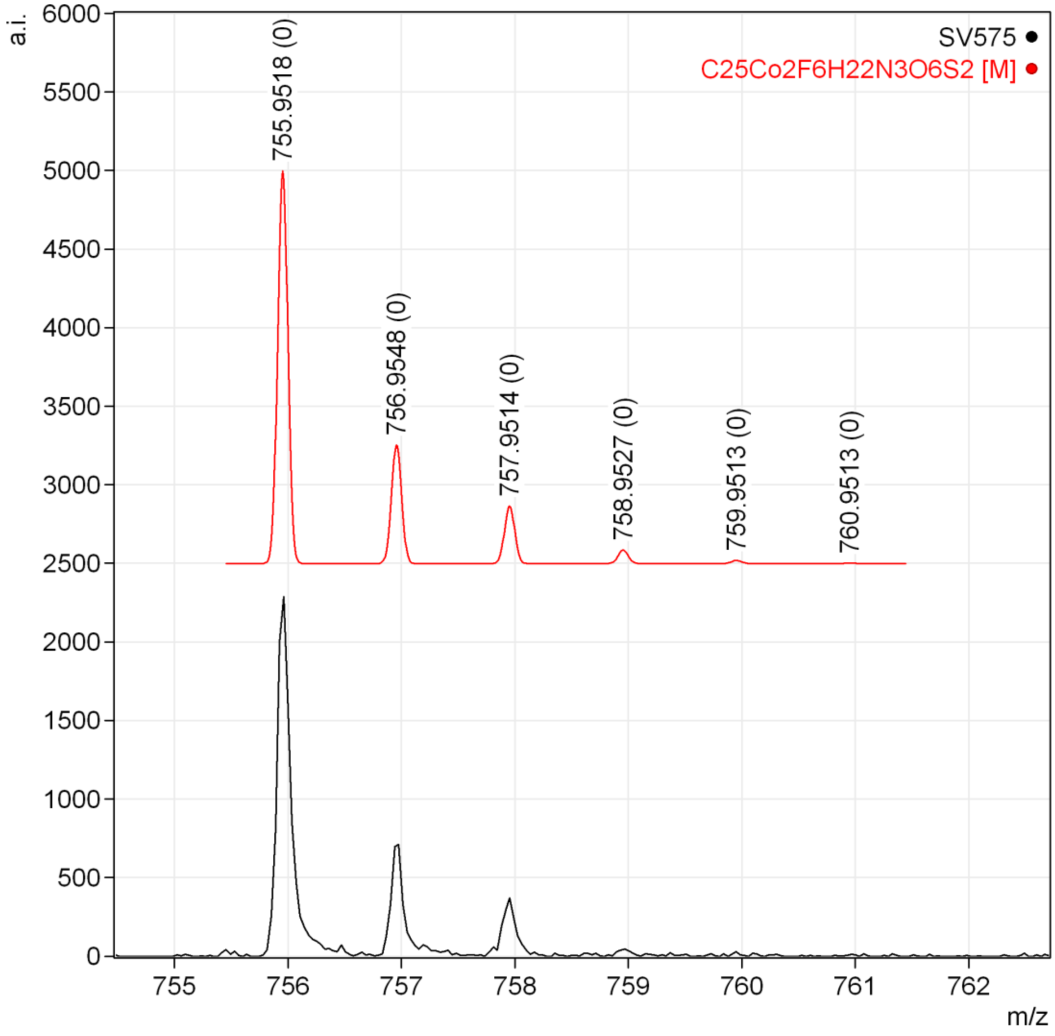

Figure S37. MS (MALDI pos, [m/z]; *top*: simulated, *bottom*: experimental) of "1,4-dicobaltoceniumyl-3-methyltriazolium bis(hexafluoridophosphate) triflate".

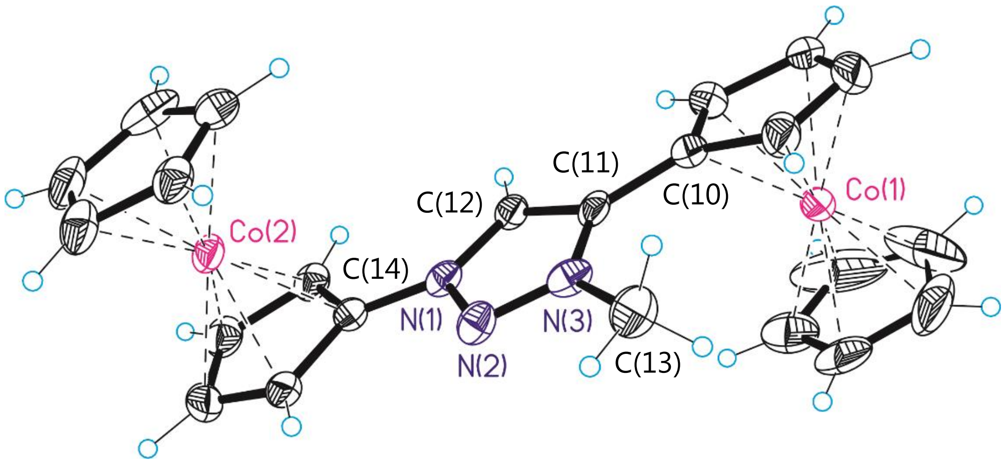

Figure S38. X-ray single crystal structure analysis of "1,4-dicobaltoceniumyl-3-methyltriazolium bis(hexafluoridophosphate) triflate" (counterions omitted for clarity).

X-Ray single crystal structure analysis data

Table S31. Crystal data and structure refinement for **10a**.

Empirical formula	C26 H22 Co2 F9 N3 O9 S3 X C3H6O	
Formula weight	963.58	
Temperature	203(2) K	
Wavelength	0.71073 Å	
Crystal system	Triclinic	
Space group	P-1 (no. 2)	
Unit cell dimensions	a = 12.8390(10) Å	α = 91.539(2)°.
	b = 16.453(2) Å	ß = 108.422(2)°.
	c = 18.336(2) Å	γ = 95.085(2)°.
Volume	3654.2(7) $Å^3$	
Z	4	
Density (calculated)	1.751 Mg/m^3	
Absorption coefficient	1.184 mm^{-1}	
F(000)	1944	
Crystal size	0.180 x 0.120 x 0.100 mm^3	
Theta range for data collection	2.179 to 25.000°.	
Index ranges	-15<h<15, -19<k<17, -21<l<21	
Reflections collected	46648	
Independent reflections	12852 [R(int) = 0.0421]	
Completeness to theta = 25.000°	99.7 %	
Absorption correction	Semi-empirical from equivalents	
Max. and min. transmission	0.887 and 0.795	
Refinement method	Full-matrix least-squares on F^2	
Data / restraints / parameters	12852 / 0 / 1025	
Goodness-of-fit on F^2	1.025	
Final R indices [I>2sigma(I)]	R1 = 0.0716, wR2 = 0.1631	
R indices (all data)	R1 = 0.1026, wR2 = 0.1787	
Extinction coefficient	n/a	
Largest diff. peak and hole	0.818 and -0.641 e.$Å^{-3}$	

Table S32. Atomic coordinates (x 10^4) and equivalent isotropic displacement parameters (Å^{2}x 10^3) for **10a**. U(eq) is defined as one third of the trace of the orthogonalized U^{ij} tensor.

	x	y	z	U(eq)
Co(1)	7179(1)	7624(1)	2670(1)	50(1)
Co(2)	2580(1)	7818(1)	4761(1)	41(1)
Co(3)	7331(1)	7740(1)	7679(1)	46(1)
Co(4)	2525(1)	6794(1)	9604(1)	38(1)
N(1)	4525(3)	8121(3)	4125(3)	38(1)
N(2)	4717(4)	8885(3)	3957(3)	44(1)
N(3)	5081(4)	8810(3)	3378(3)	43(1)
N(5)	4711(5)	6068(3)	8645(5)	85(2)
N(6)	5164(5)	6258(4)	8130(5)	85(2)
C(1)	8439(10)	7122(8)	3386(10)	164(8)
C(2)	8700(11)	7443(17)	2796(10)	231(16)
C(3)	8623(9)	8284(13)	2838(6)	174(8)
C(4)	8285(6)	8435(6)	3440(5)	79(2)
C(5)	8157(7)	7735(6)	3774(5)	82(2)
C(6)	5766(5)	6896(4)	2483(4)	59(2)
C(7)	6161(6)	6819(6)	1849(4)	74(2)
C(8)	6224(6)	7588(6)	1561(4)	74(2)
C(9)	5875(5)	8141(5)	1994(4)	58(2)
C(10)	5571(4)	7722(4)	2576(3)	45(1)
C(11)	5159(4)	8014(3)	3173(3)	36(1)
C(12)	4789(4)	7567(3)	3671(3)	36(1)
C(13)	5364(6)	9575(4)	3054(4)	60(2)
C(14)	4161(4)	7995(3)	4770(3)	38(1)
C(15)	3948(4)	8627(4)	5222(3)	48(2)
C(16)	3629(5)	8249(5)	5810(4)	60(2)
C(17)	3636(5)	7397(5)	5710(3)	54(2)
C(18)	3963(4)	7230(4)	5051(3)	42(1)
C(19)	1473(5)	8378(4)	3927(4)	52(2)
C(20)	1149(5)	8323(5)	4594(4)	60(2)
C(21)	1016(5)	7503(5)	4745(5)	68(2)
C(22)	1269(5)	7034(5)	4177(5)	72(2)
C(23)	1560(5)	7574(4)	3669(4)	57(2)
C(31)	8670(9)	8345(6)	8424(9)	155(7)
C(32)	8910(8)	8008(15)	7823(9)	195(12)
C(33)	8711(8)	7181(11)	7818(5)	143(6)
C(34)	8307(5)	7014(4)	8412(4)	62(2)
C(35)	8291(6)	7732(5)	8776(4)	71(2)
C(36)	5976(5)	8332(4)	7569(4)	51(2)
C(37)	6391(5)	8504(5)	6951(4)	56(2)
C(38)	6388(5)	7755(5)	6560(4)	56(2)
C(39)	5976(4)	7107(4)	6926(3)	49(2)
C(40)	5704(4)	7479(4)	7543(3)	43(1)
C(42)	4831(4)	7396(3)	8599(3)	40(1)

C(44)	4108(4)	6761(3)	9596(3)	41(1)
C(45)	3782(5)	6073(4)	9942(4)	51(2)
C(46)	3502(5)	6365(4)	10576(4)	55(2)
C(47)	3631(5)	7228(4)	10614(3)	54(2)
C(48)	4002(4)	7477(4)	10002(3)	44(1)
C(49)	1232(5)	6190(5)	8770(4)	62(2)
C(50)	959(5)	6322(5)	9451(4)	65(2)
C(51)	1066(5)	7167(5)	9620(4)	63(2)
C(52)	1412(5)	7551(4)	9050(4)	57(2)
C(53)	1522(5)	6949(5)	8529(4)	58(2)
C(24)	-1341(7)	6310(6)	10691(4)	75(2)
C(25)	904(11)	5559(7)	6364(6)	115(4)
C(26)	1766(6)	8949(5)	7277(4)	68(2)
C(27)	8314(7)	4362(5)	7801(5)	77(2)
C(28)	8817(5)	9194(4)	5733(4)	53(2)
C(29)	905(9)	8980(8)	1603(7)	116(4)
C(54)	6609(7)	5890(6)	4938(6)	87(3)
C(55)	6226(10)	5022(6)	4713(7)	124(4)
C(56)	7770(9)	6098(8)	5375(6)	131(4)
C(57)	6590(7)	9470(6)	9817(6)	98(3)
C(58)	6146(8)	10257(6)	9647(5)	90(3)
C(59)	7754(8)	9477(8)	10316(6)	125(4)
O(20)	6060(7)	8863(5)	9599(8)	245(7)
O(19)	6020(7)	6402(5)	4817(6)	172(4)
S(1)	-2739(1)	6240(1)	10122(1)	59(1)
S(2)	2291(2)	5460(2)	6930(1)	86(1)
S(3)	3136(1)	9171(1)	7968(1)	43(1)
S(4)	7006(2)	4489(1)	7087(1)	77(1)
S(5)	7384(1)	9270(1)	5178(1)	44(1)
S(6)	2348(2)	9036(1)	1881(1)	68(1)
O(1)	-2942(6)	7043(4)	10031(6)	153(4)
O(2)	-2716(6)	5781(6)	9467(4)	150(4)
O(3)	-3344(5)	5826(3)	10553(3)	79(2)
O(4)	2112(9)	5251(8)	7626(4)	213(6)
O(5)	2847(8)	6186(5)	6973(7)	211(6)
O(6)	2603(7)	4761(5)	6647(5)	149(3)
O(7)	3200(4)	10028(3)	8137(3)	67(1)
O(8)	3841(4)	8932(3)	7550(3)	60(1)
O(9)	3108(3)	8668(3)	8590(2)	55(1)
O(10)	6222(5)	4195(4)	7461(4)	123(3)
O(11)	7077(4)	5349(3)	6972(3)	74(1)
O(12)	7016(6)	3972(3)	6439(3)	104(2)
O(13)	7461(4)	9752(3)	4561(2)	65(1)
O(14)	6964(4)	9676(3)	5709(3)	74(1)
O(15)	6964(4)	8441(3)	4977(3)	66(1)
O(16)	2610(6)	8707(6)	2614(4)	149(3)
O(17)	2547(10)	8522(4)	1346(6)	201(5)
O(18)	2682(5)	9870(3)	1827(4)	101(2)
F(1)	-985(7)	5586(6)	10746(7)	218(5)
F(2)	-1175(5)	6579(8)	11360(3)	224(5)

F(3)	-692(4)	6729(4)	10389(3)	101(2)
F(4)	927(9)	5736(6)	5645(4)	221(5)
F(5)	264(6)	4892(5)	6208(5)	176(3)
F(6)	458(7)	6116(6)	6607(6)	207(5)
F(7)	1545(5)	8180(3)	7058(3)	121(2)
F(8)	1019(4)	9161(4)	7589(3)	116(2)
F(9)	1661(3)	9366(3)	6656(2)	94(2)
F(10)	9136(4)	4580(5)	7568(3)	128(2)
F(11)	8379(6)	3607(4)	8000(3)	131(2)
F(12)	8444(5)	4810(3)	8443(3)	111(2)
F(13)	8888(4)	8754(4)	6318(3)	128(2)
F(14)	9338(3)	8852(4)	5327(3)	105(2)
F(15)	9371(4)	9912(3)	5999(3)	96(2)
F(16)	525(7)	9279(9)	939(6)	291(8)
F(17)	609(8)	9452(6)	2088(8)	229(6)
F(18)	447(6)	8261(5)	1621(5)	192(4)
N(4)	4513(4)	6761(3)	8960(3)	40(1)
C(41)	5244(4)	7075(3)	8065(3)	42(1)
C(43)	5460(10)	5509(7)	7891(7)	51(3)
N(4A)	5244(4)	7075(3)	8065(3)	42(1)
C(41A)	4513(4)	6761(3)	8960(3)	40(1)
C(43A)	4576(13)	5241(7)	8577(8)	50(4)

Table S33. Bond lengths [Å] and angles [°] for **10a**.

Co(1)-C(2)	1.943(11)	Co(3)-C(32)	1.967(10)
Co(1)-C(3)	1.994(10)	Co(3)-C(31)	1.989(9)
Co(1)-C(1)	1.994(10)	Co(3)-C(35)	2.002(7)
Co(1)-C(5)	2.011(8)	Co(3)-C(33)	2.016(8)
Co(1)-C(8)	2.011(7)	Co(3)-C(34)	2.023(6)
Co(1)-C(6)	2.012(6)	Co(3)-C(39)	2.024(6)
Co(1)-C(9)	2.014(7)	Co(3)-C(36)	2.027(6)
Co(1)-C(7)	2.017(6)	Co(3)-C(38)	2.028(6)
Co(1)-C(4)	2.021(7)	Co(3)-C(40)	2.028(5)
Co(1)-C(10)	2.039(6)	Co(3)-C(37)	2.039(6)
Co(2)-C(22)	2.012(7)	Co(4)-C(47)	2.012(6)
Co(2)-C(21)	2.019(6)	Co(4)-C(46)	2.016(6)
Co(2)-C(14)	2.020(5)	Co(4)-C(50)	2.020(6)
Co(2)-C(17)	2.022(6)	Co(4)-C(52)	2.022(6)
Co(2)-C(20)	2.022(6)	Co(4)-C(53)	2.022(6)
Co(2)-C(23)	2.022(6)	Co(4)-C(48)	2.023(6)
Co(2)-C(18)	2.027(5)	Co(4)-C(49)	2.025(6)
Co(2)-C(19)	2.034(6)	Co(4)-C(51)	2.031(6)
Co(2)-C(16)	2.034(6)	Co(4)-C(45)	2.035(6)
Co(2)-C(15)	2.040(6)	Co(4)-C(44)	2.043(5)

N(1)-N(2)	1.323(6)	C(20)-C(21)	1.392(10)
N(1)-C(12)	1.353(7)	C(20)-H(20)	0.9500
N(1)-C(14)	1.415(7)	C(21)-C(22)	1.413(11)
N(2)-N(3)	1.294(7)	C(21)-H(21)	0.9500
N(3)-C(11)	1.374(7)	C(22)-C(23)	1.415(10)
N(3)-C(13)	1.472(7)	C(22)-H(22)	0.9500
N(5)-N(6)	1.288(10)	C(23)-H(23)	0.9500
N(5)-C(41A)	1.344(7)	C(31)-C(35)	1.349(14)
N(5)-N(4)	1.344(7)	C(31)-C(32)	1.35(2)
N(5)-C(43A)	1.354(13)	C(31)-H(31)	0.9500
N(6)-N(4A)	1.350(8)	C(32)-C(33)	1.36(2)
N(6)-C(41)	1.350(8)	C(32)-H(32)	0.9500
N(6)-C(43)	1.419(11)	C(33)-C(34)	1.371(14)
C(1)-C(2)	1.34(3)	C(33)-H(33)	0.9500
C(1)-C(5)	1.357(15)	C(34)-C(35)	1.346(10)
C(1)-H(1)	0.9500	C(34)-H(34)	0.9500
C(2)-C(3)	1.40(3)	C(35)-H(35)	0.9500
C(2)-H(2)	0.9500	C(36)-C(40)	1.412(9)
C(3)-C(4)	1.333(14)	C(36)-C(37)	1.421(9)
C(3)-H(3)	0.9500	C(36)-H(36)	0.9500
C(4)-C(5)	1.341(11)	C(37)-C(38)	1.408(10)
C(4)-H(4)	0.9500	C(37)-H(37)	0.9500
C(5)-H(5)	0.9500	C(38)-C(39)	1.425(9)
C(6)-C(7)	1.415(9)	C(38)-H(38)	0.9500
C(6)-C(10)	1.418(9)	C(39)-C(40)	1.424(8)
C(6)-H(6)	0.9500	C(39)-H(39)	0.9500
C(7)-C(8)	1.390(11)	C(40)-N(4A)	1.425(8)
C(7)-H(7)	0.9500	C(40)-C(41)	1.425(8)
C(8)-C(9)	1.384(10)	C(42)-C(41A)	1.352(7)
C(8)-H(8)	0.9500	C(42)-N(4)	1.352(7)
C(9)-C(10)	1.423(8)	C(42)-C(41)	1.367(7)
C(9)-H(9)	0.9500	C(42)-N(4A)	1.367(7)
C(10)-C(11)	1.445(8)	C(42)-H(42)	0.9500
C(11)-C(12)	1.360(7)	C(44)-C(45)	1.407(8)
C(12)-H(12)	0.9500	C(44)-C(48)	1.416(8)
C(13)-H(13A)	0.9800	C(44)-C(41A)	1.420(8)
C(13)-H(13B)	0.9800	C(44)-N(4)	1.420(8)
C(13)-H(13C)	0.9800	C(45)-C(46)	1.405(9)
C(14)-C(18)	1.407(8)	C(45)-H(45)	0.9500
C(14)-C(15)	1.412(8)	C(46)-C(47)	1.412(9)
C(15)-C(16)	1.408(9)	C(46)-H(46)	0.9500
C(15)-H(15)	0.9500	C(47)-C(48)	1.409(8)
C(16)-C(17)	1.411(10)	C(47)-H(47)	0.9500
C(16)-H(16)	0.9500	C(48)-H(48)	0.9500
C(17)-C(18)	1.425(8)	C(49)-C(53)	1.394(9)
C(17)-H(17)	0.9500	C(49)-C(50)	1.416(9)
C(18)-H(18)	0.9500	C(49)-H(49)	0.9500
C(19)-C(20)	1.414(9)	C(50)-C(51)	1.402(10)
C(19)-C(23)	1.419(9)	C(50)-H(50)	0.9500
C(19)-H(19)	0.9500	C(51)-C(52)	1.403(9)

C(51)-H(51)	0.9500
C(52)-C(53)	1.405(9)
C(52)-H(52)	0.9500
C(53)-H(53)	0.9500
C(24)-F(2)	1.238(9)
C(24)-F(3)	1.299(8)
C(24)-F(1)	1.309(10)
C(24)-S(1)	1.759(8)
C(25)-F(6)	1.265(13)
C(25)-F(5)	1.279(12)
C(25)-F(4)	1.365(13)
C(25)-S(2)	1.780(13)
C(26)-F(7)	1.297(9)
C(26)-F(9)	1.321(7)
C(26)-F(8)	1.328(9)
C(26)-S(3)	1.812(7)
C(27)-F(10)	1.286(10)
C(27)-F(11)	1.306(9)
C(27)-F(12)	1.329(9)
C(27)-S(4)	1.807(8)
C(28)-F(13)	1.294(7)
C(28)-F(14)	1.296(7)
C(28)-F(15)	1.319(8)
C(28)-S(5)	1.817(6)
C(29)-F(18)	1.280(12)
C(29)-F(16)	1.289(12)
C(29)-F(17)	1.327(15)
C(29)-S(6)	1.753(12)
C(54)-O(19)	1.161(9)
C(54)-C(56)	1.456(13)
C(54)-C(55)	1.473(13)
C(55)-H(55A)	0.9800
C(55)-H(55B)	0.9800
C(55)-H(55C)	0.9800
C(56)-H(56A)	0.9800
C(56)-H(56B)	0.9800
C(56)-H(56C)	0.9800
C(57)-O(20)	1.143(10)
C(57)-C(58)	1.462(12)
C(57)-C(59)	1.484(12)
C(58)-H(58A)	0.9800
C(58)-H(58B)	0.9800
C(58)-H(58C)	0.9800
C(59)-H(59A)	0.9800
C(59)-H(59B)	0.9800
C(59)-H(59C)	0.9800
S(1)-O(1)	1.374(6)
S(1)-O(2)	1.412(6)
S(1)-O(3)	1.419(5)
S(2)-O(5)	1.324(7)
S(2)-O(6)	1.391(7)
S(2)-O(4)	1.411(8)
S(3)-O(7)	1.426(5)
S(3)-O(8)	1.431(4)
S(3)-O(9)	1.435(4)
S(4)-O(11)	1.435(5)
S(4)-O(10)	1.444(7)
S(4)-O(12)	1.448(6)
S(5)-O(15)	1.415(5)
S(5)-O(13)	1.423(4)
S(5)-O(14)	1.431(5)
S(6)-O(17)	1.376(7)
S(6)-O(16)	1.415(6)
S(6)-O(18)	1.416(6)
C(43)-H(43A)	0.9800
C(43)-H(43B)	0.9800
C(43)-H(43C)	0.9800
C(43A)-H(43D)	0.9800
C(43A)-H(43E)	0.9800
C(43A)-H(43F)	0.9800

C(2)-Co(1)-C(3)	41.6(8)
C(2)-Co(1)-C(1)	39.7(8)
C(3)-Co(1)-C(1)	67.4(7)
C(2)-Co(1)-C(5)	66.7(5)
C(3)-Co(1)-C(5)	66.0(4)
C(1)-Co(1)-C(5)	39.6(5)
C(2)-Co(1)-C(8)	113.2(5)
C(3)-Co(1)-C(8)	111.5(4)
C(1)-Co(1)-C(8)	142.5(6)
C(5)-Co(1)-C(8)	176.5(4)
C(2)-Co(1)-C(6)	134.9(8)
C(3)-Co(1)-C(6)	176.3(7)
C(1)-Co(1)-C(6)	110.3(5)
C(5)-Co(1)-C(6)	114.3(3)
C(8)-Co(1)-C(6)	68.4(3)
C(2)-Co(1)-C(9)	143.1(9)
C(3)-Co(1)-C(9)	113.8(6)
C(1)-Co(1)-C(9)	176.9(6)
C(5)-Co(1)-C(9)	137.8(3)
C(8)-Co(1)-C(9)	40.2(3)
C(6)-Co(1)-C(9)	68.6(3)
C(2)-Co(1)-C(7)	109.2(5)

C(3)-Co(1)-C(7)	136.5(5)	C(18)-Co(2)-C(19)	146.9(2)
C(1)-Co(1)-C(7)	113.1(4)	C(22)-Co(2)-C(16)	146.6(3)
C(5)-Co(1)-C(7)	143.2(4)	C(21)-Co(2)-C(16)	114.2(3)
C(8)-Co(1)-C(7)	40.4(3)	C(14)-Co(2)-C(16)	67.8(2)
C(6)-Co(1)-C(7)	41.1(3)	C(17)-Co(2)-C(16)	40.7(3)
C(9)-Co(1)-C(7)	68.2(3)	C(20)-Co(2)-C(16)	107.7(3)
C(2)-Co(1)-C(4)	67.0(5)	C(23)-Co(2)-C(16)	170.9(3)
C(3)-Co(1)-C(4)	38.8(4)	C(18)-Co(2)-C(16)	69.1(3)
C(1)-Co(1)-C(4)	66.1(4)	C(19)-Co(2)-C(16)	131.2(3)
C(5)-Co(1)-C(4)	38.9(3)	C(22)-Co(2)-C(15)	172.7(3)
C(8)-Co(1)-C(4)	137.7(4)	C(21)-Co(2)-C(15)	144.7(3)
C(6)-Co(1)-C(4)	143.6(3)	C(14)-Co(2)-C(15)	40.7(2)
C(9)-Co(1)-C(4)	112.9(3)	C(17)-Co(2)-C(15)	68.6(3)
C(7)-Co(1)-C(4)	175.3(3)	C(20)-Co(2)-C(15)	113.4(3)
C(2)-Co(1)-C(10)	175.1(10)	C(23)-Co(2)-C(15)	132.2(3)
C(3)-Co(1)-C(10)	142.6(7)	C(18)-Co(2)-C(15)	69.4(2)
C(1)-Co(1)-C(10)	136.2(6)	C(19)-Co(2)-C(15)	108.0(3)
C(5)-Co(1)-C(10)	111.7(3)	C(16)-Co(2)-C(15)	40.5(3)
C(8)-Co(1)-C(10)	68.7(3)	C(32)-Co(3)-C(31)	39.9(7)
C(6)-Co(1)-C(10)	41.0(3)	C(32)-Co(3)-C(35)	66.6(5)
C(9)-Co(1)-C(10)	41.1(2)	C(31)-Co(3)-C(35)	39.5(4)
C(7)-Co(1)-C(10)	69.1(3)	C(32)-Co(3)-C(33)	39.9(6)
C(4)-Co(1)-C(10)	114.9(3)	C(31)-Co(3)-C(33)	66.7(6)
C(22)-Co(2)-C(21)	41.1(3)	C(35)-Co(3)-C(33)	66.1(4)
C(22)-Co(2)-C(14)	134.2(3)	C(32)-Co(3)-C(34)	66.9(5)
C(21)-Co(2)-C(14)	173.4(3)	C(31)-Co(3)-C(34)	66.4(3)
C(22)-Co(2)-C(17)	115.8(3)	C(35)-Co(3)-C(34)	39.1(3)
C(21)-Co(2)-C(17)	109.1(3)	C(33)-Co(3)-C(34)	39.7(4)
C(14)-Co(2)-C(17)	68.0(2)	C(32)-Co(3)-C(39)	139.8(7)
C(22)-Co(2)-C(20)	68.5(3)	C(31)-Co(3)-C(39)	179.1(4)
C(21)-Co(2)-C(20)	40.3(3)	C(35)-Co(3)-C(39)	139.8(3)
C(14)-Co(2)-C(20)	146.0(3)	C(33)-Co(3)-C(39)	112.6(5)
C(17)-Co(2)-C(20)	131.6(3)	C(34)-Co(3)-C(39)	112.7(3)
C(22)-Co(2)-C(23)	41.1(3)	C(32)-Co(3)-C(36)	138.5(8)
C(21)-Co(2)-C(23)	69.1(3)	C(31)-Co(3)-C(36)	111.2(5)
C(14)-Co(2)-C(23)	110.0(2)	C(35)-Co(3)-C(36)	112.3(3)
C(17)-Co(2)-C(23)	147.6(3)	C(33)-Co(3)-C(36)	177.9(4)
C(20)-Co(2)-C(23)	69.0(3)	C(34)-Co(3)-C(36)	139.7(3)
C(22)-Co(2)-C(18)	109.7(3)	C(39)-Co(3)-C(36)	69.5(3)
C(21)-Co(2)-C(18)	133.3(3)	C(32)-Co(3)-C(38)	112.2(4)
C(14)-Co(2)-C(18)	40.7(2)	C(31)-Co(3)-C(38)	139.5(5)
C(17)-Co(2)-C(18)	41.2(2)	C(35)-Co(3)-C(38)	178.7(3)
C(20)-Co(2)-C(18)	171.8(3)	C(33)-Co(3)-C(38)	113.0(3)
C(23)-Co(2)-C(18)	115.3(3)	C(34)-Co(3)-C(38)	140.8(3)
C(22)-Co(2)-C(19)	68.6(3)	C(39)-Co(3)-C(38)	41.2(3)
C(21)-Co(2)-C(19)	68.4(3)	C(36)-Co(3)-C(38)	68.6(3)
C(14)-Co(2)-C(19)	115.5(2)	C(32)-Co(3)-C(40)	179.0(8)
C(17)-Co(2)-C(19)	170.7(3)	C(31)-Co(3)-C(40)	139.1(6)
C(20)-Co(2)-C(19)	40.8(3)	C(35)-Co(3)-C(40)	112.8(3)
C(23)-Co(2)-C(19)	40.9(3)	C(33)-Co(3)-C(40)	140.8(6)

C(34)-Co(3)-C(40)	113.2(3)	C(46)-Co(4)-C(44)	67.7(2)
C(39)-Co(3)-C(40)	41.1(2)	C(50)-Co(4)-C(44)	155.0(3)
C(36)-Co(3)-C(40)	40.8(2)	C(52)-Co(4)-C(44)	128.1(2)
C(38)-Co(3)-C(40)	68.4(2)	C(53)-Co(4)-C(44)	109.8(2)
C(32)-Co(3)-C(37)	111.4(5)	C(48)-Co(4)-C(44)	40.8(2)
C(31)-Co(3)-C(37)	111.6(4)	C(49)-Co(4)-C(44)	121.4(3)
C(35)-Co(3)-C(37)	139.6(3)	C(51)-Co(4)-C(44)	164.0(3)
C(33)-Co(3)-C(37)	139.5(5)	C(45)-Co(4)-C(44)	40.4(2)
C(34)-Co(3)-C(37)	178.0(3)	N(2)-N(1)-C(12)	113.2(5)
C(39)-Co(3)-C(37)	69.3(3)	N(2)-N(1)-C(14)	117.4(5)
C(36)-Co(3)-C(37)	40.9(2)	C(12)-N(1)-C(14)	129.3(5)
C(38)-Co(3)-C(37)	40.5(3)	N(3)-N(2)-N(1)	103.5(4)
C(40)-Co(3)-C(37)	68.5(2)	N(2)-N(3)-C(11)	113.7(4)
C(47)-Co(4)-C(46)	41.0(3)	N(2)-N(3)-C(13)	116.2(5)
C(47)-Co(4)-C(50)	125.5(3)	C(11)-N(3)-C(13)	130.1(5)
C(46)-Co(4)-C(50)	107.7(3)	N(6)-N(5)-C(41A)	108.4(6)
C(47)-Co(4)-C(52)	116.9(3)	N(6)-N(5)-N(4)	108.4(6)
C(46)-Co(4)-C(52)	151.5(3)	N(6)-N(5)-C(43A)	103.1(8)
C(50)-Co(4)-C(52)	68.0(3)	C(41A)-N(5)-C(43A)	148.2(9)
C(47)-Co(4)-C(53)	151.9(3)	N(5)-N(6)-N(4A)	110.4(6)
C(46)-Co(4)-C(53)	166.4(3)	N(5)-N(6)-C(41)	110.4(6)
C(50)-Co(4)-C(53)	68.5(3)	N(5)-N(6)-C(43)	104.9(8)
C(52)-Co(4)-C(53)	40.7(3)	C(41)-N(6)-C(43)	144.5(9)
C(47)-Co(4)-C(48)	40.9(2)	C(2)-C(1)-C(5)	107.7(14)
C(46)-Co(4)-C(48)	68.7(3)	C(2)-C(1)-Co(1)	68.1(8)
C(50)-Co(4)-C(48)	162.8(3)	C(5)-C(1)-Co(1)	70.9(5)
C(52)-Co(4)-C(48)	106.7(3)	C(2)-C(1)-H(1)	126.2
C(53)-Co(4)-C(48)	118.9(2)	C(5)-C(1)-H(1)	126.2
C(47)-Co(4)-C(49)	164.8(3)	Co(1)-C(1)-H(1)	126.4
C(46)-Co(4)-C(49)	128.5(3)	C(1)-C(2)-C(3)	107.9(10)
C(50)-Co(4)-C(49)	41.0(3)	C(1)-C(2)-Co(1)	72.2(8)
C(52)-Co(4)-C(49)	67.8(3)	C(3)-C(2)-Co(1)	71.1(7)
C(53)-Co(4)-C(49)	40.3(3)	C(1)-C(2)-H(2)	126.0
C(48)-Co(4)-C(49)	154.0(3)	C(3)-C(2)-H(2)	126.0
C(47)-Co(4)-C(51)	105.2(3)	Co(1)-C(2)-H(2)	122.3
C(46)-Co(4)-C(51)	117.7(3)	C(4)-C(3)-C(2)	106.5(14)
C(50)-Co(4)-C(51)	40.5(3)	C(4)-C(3)-Co(1)	71.7(5)
C(52)-Co(4)-C(51)	40.5(3)	C(2)-C(3)-Co(1)	67.3(7)
C(53)-Co(4)-C(51)	68.6(3)	C(4)-C(3)-H(3)	126.8
C(48)-Co(4)-C(51)	125.0(3)	C(2)-C(3)-H(3)	126.8
C(49)-Co(4)-C(51)	68.4(3)	Co(1)-C(3)-H(3)	125.8
C(47)-Co(4)-C(45)	69.0(3)	C(3)-C(4)-C(5)	109.4(12)
C(46)-Co(4)-C(45)	40.6(3)	C(3)-C(4)-Co(1)	69.5(6)
C(50)-Co(4)-C(45)	120.0(3)	C(5)-C(4)-Co(1)	70.2(5)
C(52)-Co(4)-C(45)	166.2(3)	C(3)-C(4)-H(4)	125.3
C(53)-Co(4)-C(45)	128.9(3)	C(5)-C(4)-H(4)	125.3
C(48)-Co(4)-C(45)	68.9(2)	Co(1)-C(4)-H(4)	126.6
C(49)-Co(4)-C(45)	110.0(3)	C(4)-C(5)-C(1)	108.5(12)
C(51)-Co(4)-C(45)	152.7(3)	C(4)-C(5)-Co(1)	71.0(5)
C(47)-Co(4)-C(44)	68.3(3)	C(1)-C(5)-Co(1)	69.5(6)

C(4)-C(5)-H(5)	125.8	N(1)-C(14)-Co(2)	126.6(4)
C(1)-C(5)-H(5)	125.8	C(16)-C(15)-C(14)	106.5(6)
Co(1)-C(5)-H(5)	125.3	C(16)-C(15)-Co(2)	69.5(4)
C(7)-C(6)-C(10)	108.5(7)	C(14)-C(15)-Co(2)	68.9(3)
C(7)-C(6)-Co(1)	69.7(4)	C(16)-C(15)-H(15)	126.7
C(10)-C(6)-Co(1)	70.5(3)	C(14)-C(15)-H(15)	126.7
C(7)-C(6)-H(6)	125.7	Co(2)-C(15)-H(15)	126.4
C(10)-C(6)-H(6)	125.7	C(15)-C(16)-C(17)	108.6(6)
Co(1)-C(6)-H(6)	125.7	C(15)-C(16)-Co(2)	70.0(3)
C(8)-C(7)-C(6)	107.5(7)	C(17)-C(16)-Co(2)	69.2(4)
C(8)-C(7)-Co(1)	69.6(4)	C(15)-C(16)-H(16)	125.7
C(6)-C(7)-Co(1)	69.2(4)	C(17)-C(16)-H(16)	125.7
C(8)-C(7)-H(7)	126.2	Co(2)-C(16)-H(16)	126.7
C(6)-C(7)-H(7)	126.2	C(16)-C(17)-C(18)	108.6(6)
Co(1)-C(7)-H(7)	126.5	C(16)-C(17)-Co(2)	70.1(4)
C(9)-C(8)-C(7)	109.1(7)	C(18)-C(17)-Co(2)	69.6(3)
C(9)-C(8)-Co(1)	70.0(4)	C(16)-C(17)-H(17)	125.7
C(7)-C(8)-Co(1)	70.1(4)	C(18)-C(17)-H(17)	125.7
C(9)-C(8)-H(8)	125.5	Co(2)-C(17)-H(17)	126.2
C(7)-C(8)-H(8)	125.5	C(14)-C(18)-C(17)	105.8(6)
Co(1)-C(8)-H(8)	126.1	C(14)-C(18)-Co(2)	69.4(3)
C(8)-C(9)-C(10)	108.9(7)	C(17)-C(18)-Co(2)	69.2(3)
C(8)-C(9)-Co(1)	69.8(4)	C(14)-C(18)-H(18)	127.1
C(10)-C(9)-Co(1)	70.4(4)	C(17)-C(18)-H(18)	127.1
C(8)-C(9)-H(9)	125.6	Co(2)-C(18)-H(18)	125.9
C(10)-C(9)-H(9)	125.6	C(20)-C(19)-C(23)	108.0(6)
Co(1)-C(9)-H(9)	125.9	C(20)-C(19)-Co(2)	69.2(3)
C(6)-C(10)-C(9)	106.0(6)	C(23)-C(19)-Co(2)	69.1(3)
C(6)-C(10)-C(11)	123.1(5)	C(20)-C(19)-H(19)	126.0
C(9)-C(10)-C(11)	130.9(6)	C(23)-C(19)-H(19)	126.0
C(6)-C(10)-Co(1)	68.5(3)	Co(2)-C(19)-H(19)	127.3
C(9)-C(10)-Co(1)	68.5(3)	C(21)-C(20)-C(19)	108.5(7)
C(11)-C(10)-Co(1)	126.7(4)	C(21)-C(20)-Co(2)	69.7(4)
C(12)-C(11)-N(3)	104.3(5)	C(19)-C(20)-Co(2)	70.0(3)
C(12)-C(11)-C(10)	127.9(5)	C(21)-C(20)-H(20)	125.8
N(3)-C(11)-C(10)	127.8(5)	C(19)-C(20)-H(20)	125.8
N(1)-C(12)-C(11)	105.3(5)	Co(2)-C(20)-H(20)	126.1
N(1)-C(12)-H(12)	127.4	C(20)-C(21)-C(22)	108.1(7)
C(11)-C(12)-H(12)	127.4	C(20)-C(21)-Co(2)	70.0(4)
N(3)-C(13)-H(13A)	109.5	C(22)-C(21)-Co(2)	69.2(4)
N(3)-C(13)-H(13B)	109.5	C(20)-C(21)-H(21)	126.0
H(13A)-C(13)-H(13B)	109.5	C(22)-C(21)-H(21)	126.0
N(3)-C(13)-H(13C)	109.5	Co(2)-C(21)-H(21)	126.5
H(13A)-C(13)-H(13C)	109.5	C(21)-C(22)-C(23)	108.4(7)
H(13B)-C(13)-H(13C)	109.5	C(21)-C(22)-Co(2)	69.8(4)
C(18)-C(14)-C(15)	110.4(5)	C(23)-C(22)-Co(2)	69.9(4)
C(18)-C(14)-N(1)	125.2(5)	C(21)-C(22)-H(22)	125.8
C(15)-C(14)-N(1)	124.4(5)	C(23)-C(22)-H(22)	125.8
C(18)-C(14)-Co(2)	69.9(3)	Co(2)-C(22)-H(22)	126.1
C(15)-C(14)-Co(2)	70.4(3)	C(22)-C(23)-C(19)	107.1(7)

C(22)-C(23)-Co(2)	69.1(4)	C(37)-C(38)-H(38)	125.4
C(19)-C(23)-Co(2)	70.0(4)	C(39)-C(38)-H(38)	125.4
C(22)-C(23)-H(23)	126.5	Co(3)-C(38)-H(38)	126.7
C(19)-C(23)-H(23)	126.5	C(40)-C(39)-C(38)	106.4(6)
Co(2)-C(23)-H(23)	126.1	C(40)-C(39)-Co(3)	69.6(3)
C(35)-C(31)-C(32)	107.5(12)	C(38)-C(39)-Co(3)	69.6(4)
C(35)-C(31)-Co(3)	70.7(5)	C(40)-C(39)-H(39)	126.8
C(32)-C(31)-Co(3)	69.2(7)	C(38)-C(39)-H(39)	126.8
C(35)-C(31)-H(31)	126.2	Co(3)-C(39)-H(39)	125.6
C(32)-C(31)-H(31)	126.2	C(36)-C(40)-C(39)	109.0(6)
Co(3)-C(31)-H(31)	125.4	C(36)-C(40)-N(4A)	124.2(5)
C(31)-C(32)-C(33)	108.5(9)	C(39)-C(40)-N(4A)	126.8(6)
C(31)-C(32)-Co(3)	70.9(6)	C(36)-C(40)-C(41)	124.2(5)
C(33)-C(32)-Co(3)	72.0(6)	C(39)-C(40)-C(41)	126.8(6)
C(31)-C(32)-H(32)	125.7	C(36)-C(40)-Co(3)	69.6(3)
C(33)-C(32)-H(32)	125.7	C(39)-C(40)-Co(3)	69.3(3)
Co(3)-C(32)-H(32)	123.0	N(4A)-C(40)-Co(3)	126.7(4)
C(32)-C(33)-C(34)	107.2(11)	C(41)-C(40)-Co(3)	126.7(4)
C(32)-C(33)-Co(3)	68.1(7)	N(4)-C(42)-C(41)	106.9(5)
C(34)-C(33)-Co(3)	70.4(4)	C(41A)-C(42)-N(4A)	106.9(5)
C(32)-C(33)-H(33)	126.4	N(4)-C(42)-H(42)	126.6
C(34)-C(33)-H(33)	126.4	C(41)-C(42)-H(42)	126.6
Co(3)-C(33)-H(33)	126.6	C(45)-C(44)-C(48)	108.9(5)
C(35)-C(34)-C(33)	107.5(9)	C(45)-C(44)-C(41A)	127.0(5)
C(35)-C(34)-Co(3)	69.6(4)	C(48)-C(44)-C(41A)	124.1(5)
C(33)-C(34)-Co(3)	69.9(5)	C(45)-C(44)-N(4)	127.0(5)
C(35)-C(34)-H(34)	126.3	C(48)-C(44)-N(4)	124.1(5)
C(33)-C(34)-H(34)	126.3	C(45)-C(44)-Co(4)	69.5(3)
Co(3)-C(34)-H(34)	125.8	C(48)-C(44)-Co(4)	68.9(3)
C(34)-C(35)-C(31)	109.2(10)	C(41A)-C(44)-Co(4)	129.0(4)
C(34)-C(35)-Co(3)	71.3(4)	N(4)-C(44)-Co(4)	129.0(4)
C(31)-C(35)-Co(3)	69.7(5)	C(46)-C(45)-C(44)	107.1(6)
C(34)-C(35)-H(35)	125.4	C(46)-C(45)-Co(4)	69.0(3)
C(31)-C(35)-H(35)	125.4	C(44)-C(45)-Co(4)	70.1(3)
Co(3)-C(35)-H(35)	125.1	C(46)-C(45)-H(45)	126.5
C(40)-C(36)-C(37)	107.8(6)	C(44)-C(45)-H(45)	126.5
C(40)-C(36)-Co(3)	69.7(3)	Co(4)-C(45)-H(45)	126.0
C(37)-C(36)-Co(3)	70.0(3)	C(45)-C(46)-C(47)	108.9(6)
C(40)-C(36)-H(36)	126.1	C(45)-C(46)-Co(4)	70.4(4)
C(37)-C(36)-H(36)	126.1	C(47)-C(46)-Co(4)	69.3(3)
Co(3)-C(36)-H(36)	125.8	C(45)-C(46)-H(46)	125.5
C(38)-C(37)-C(36)	107.7(6)	C(47)-C(46)-H(46)	125.5
C(38)-C(37)-Co(3)	69.3(4)	Co(4)-C(46)-H(46)	126.3
C(36)-C(37)-Co(3)	69.1(3)	C(48)-C(47)-C(46)	107.7(6)
C(38)-C(37)-H(37)	126.1	C(48)-C(47)-Co(4)	70.0(3)
C(36)-C(37)-H(37)	126.1	C(46)-C(47)-Co(4)	69.6(4)
Co(3)-C(37)-H(37)	127.0	C(48)-C(47)-H(47)	126.1
C(37)-C(38)-C(39)	109.1(6)	C(46)-C(47)-H(47)	126.1
C(37)-C(38)-Co(3)	70.2(4)	Co(4)-C(47)-H(47)	125.8
C(39)-C(38)-Co(3)	69.3(3)	C(47)-C(48)-C(44)	107.4(5)

C(47)-C(48)-Co(4)	69.1(3)	F(7)-C(26)-S(3)	112.0(5)
C(44)-C(48)-Co(4)	70.4(3)	F(9)-C(26)-S(3)	111.0(5)
C(47)-C(48)-H(48)	126.3	F(8)-C(26)-S(3)	109.6(6)
C(44)-C(48)-H(48)	126.3	F(10)-C(27)-F(11)	108.3(8)
Co(4)-C(48)-H(48)	125.7	F(10)-C(27)-F(12)	107.3(7)
C(53)-C(49)-C(50)	108.2(7)	F(11)-C(27)-F(12)	106.0(7)
C(53)-C(49)-Co(4)	69.7(4)	F(10)-C(27)-S(4)	112.3(6)
C(50)-C(49)-Co(4)	69.3(4)	F(11)-C(27)-S(4)	111.9(6)
C(53)-C(49)-H(49)	125.9	F(12)-C(27)-S(4)	110.7(6)
C(50)-C(49)-H(49)	125.9	F(13)-C(28)-F(14)	106.9(6)
Co(4)-C(49)-H(49)	126.6	F(13)-C(28)-F(15)	107.4(6)
C(51)-C(50)-C(49)	108.0(7)	F(14)-C(28)-F(15)	106.7(6)
C(51)-C(50)-Co(4)	70.2(4)	F(13)-C(28)-S(5)	110.9(5)
C(49)-C(50)-Co(4)	69.7(4)	F(14)-C(28)-S(5)	111.8(4)
C(51)-C(50)-H(50)	126.0	F(15)-C(28)-S(5)	112.8(5)
C(49)-C(50)-H(50)	126.0	F(18)-C(29)-F(16)	112.1(11)
Co(4)-C(50)-H(50)	125.7	F(18)-C(29)-F(17)	106.0(11)
C(50)-C(51)-C(52)	107.4(6)	F(16)-C(29)-F(17)	105.7(14)
C(50)-C(51)-Co(4)	69.3(4)	F(18)-C(29)-S(6)	113.4(10)
C(52)-C(51)-Co(4)	69.4(3)	F(16)-C(29)-S(6)	109.8(9)
C(50)-C(51)-H(51)	126.3	F(17)-C(29)-S(6)	109.5(9)
C(52)-C(51)-H(51)	126.3	O(19)-C(54)-C(56)	119.3(11)
Co(4)-C(51)-H(51)	126.5	O(19)-C(54)-C(55)	123.0(10)
C(51)-C(52)-C(53)	108.9(6)	C(56)-C(54)-C(55)	117.7(9)
C(51)-C(52)-Co(4)	70.1(4)	C(54)-C(55)-H(55A)	109.5
C(53)-C(52)-Co(4)	69.7(4)	C(54)-C(55)-H(55B)	109.5
C(51)-C(52)-H(52)	125.6	H(55A)-C(55)-H(55B)	109.5
C(53)-C(52)-H(52)	125.6	C(54)-C(55)-H(55C)	109.5
Co(4)-C(52)-H(52)	126.2	H(55A)-C(55)-H(55C)	109.5
C(49)-C(53)-C(52)	107.6(6)	H(55B)-C(55)-H(55C)	109.5
C(49)-C(53)-Co(4)	70.0(4)	C(54)-C(56)-H(56A)	109.5
C(52)-C(53)-Co(4)	69.6(4)	C(54)-C(56)-H(56B)	109.5
C(49)-C(53)-H(53)	126.2	H(56A)-C(56)-H(56B)	109.5
C(52)-C(53)-H(53)	126.2	C(54)-C(56)-H(56C)	109.5
Co(4)-C(53)-H(53)	125.7	H(56A)-C(56)-H(56C)	109.5
F(2)-C(24)-F(3)	109.8(9)	H(56B)-C(56)-H(56C)	109.5
F(2)-C(24)-F(1)	106.1(9)	O(20)-C(57)-C(58)	122.0(9)
F(3)-C(24)-F(1)	103.7(7)	O(20)-C(57)-C(59)	120.2(11)
F(2)-C(24)-S(1)	113.2(7)	C(58)-C(57)-C(59)	117.8(9)
F(3)-C(24)-S(1)	113.2(5)	C(57)-C(58)-H(58A)	109.5
F(1)-C(24)-S(1)	110.3(8)	C(57)-C(58)-H(58B)	109.5
F(6)-C(25)-F(5)	110.5(13)	H(58A)-C(58)-H(58B)	109.5
F(6)-C(25)-F(4)	108.2(11)	C(57)-C(58)-H(58C)	109.5
F(5)-C(25)-F(4)	100.5(9)	H(58A)-C(58)-H(58C)	109.5
F(6)-C(25)-S(2)	114.8(8)	H(58B)-C(58)-H(58C)	109.5
F(5)-C(25)-S(2)	114.9(9)	C(57)-C(59)-H(59A)	109.5
F(4)-C(25)-S(2)	106.5(10)	C(57)-C(59)-H(59B)	109.5
F(7)-C(26)-F(9)	107.7(7)	H(59A)-C(59)-H(59B)	109.5
F(7)-C(26)-F(8)	108.4(7)	C(57)-C(59)-H(59C)	109.5
F(9)-C(26)-F(8)	108.0(6)	H(59A)-C(59)-H(59C)	109.5

H(59B)-C(59)-H(59C)	109.5	O(17)-S(6)-O(16)	111.8(6)
O(1)-S(1)-O(2)	117.1(6)	O(17)-S(6)-O(18)	113.5(4)
O(1)-S(1)-O(3)	113.0(4)	O(16)-S(6)-O(18)	119.1(5)
O(2)-S(1)-O(3)	114.0(4)	O(17)-S(6)-C(29)	104.0(7)
O(1)-S(1)-C(24)	103.3(5)	O(16)-S(6)-C(29)	101.8(5)
O(2)-S(1)-C(24)	101.0(4)	O(18)-S(6)-C(29)	104.3(5)
O(3)-S(1)-C(24)	106.5(4)	N(5)-N(4)-C(42)	108.1(6)
O(5)-S(2)-O(6)	121.9(7)	N(5)-N(4)-C(44)	121.9(6)
O(5)-S(2)-O(4)	113.7(8)	C(42)-N(4)-C(44)	129.8(5)
O(6)-S(2)-O(4)	105.8(7)	N(6)-C(41)-C(42)	106.1(6)
O(5)-S(2)-C(25)	106.5(6)	N(6)-C(41)-C(40)	124.2(6)
O(6)-S(2)-C(25)	107.4(6)	C(42)-C(41)-C(40)	129.6(5)
O(4)-S(2)-C(25)	99.1(6)	N(6)-C(43)-H(43A)	109.5
O(7)-S(3)-O(8)	115.1(3)	N(6)-C(43)-H(43B)	109.5
O(7)-S(3)-O(9)	115.1(3)	H(43A)-C(43)-H(43B)	109.5
O(8)-S(3)-O(9)	114.6(3)	N(6)-C(43)-H(43C)	109.5
O(7)-S(3)-C(26)	102.8(4)	H(43A)-C(43)-H(43C)	109.5
O(8)-S(3)-C(26)	103.3(3)	H(43B)-C(43)-H(43C)	109.5
O(9)-S(3)-C(26)	103.5(3)	N(6)-N(4A)-C(42)	106.1(6)
O(11)-S(4)-O(10)	116.0(4)	N(6)-N(4A)-C(40)	124.2(6)
O(11)-S(4)-O(12)	114.7(4)	C(42)-N(4A)-C(40)	129.6(5)
O(10)-S(4)-O(12)	114.7(4)	N(5)-C(41A)-C(42)	108.1(6)
O(11)-S(4)-C(27)	103.5(4)	N(5)-C(41A)-C(44)	121.9(6)
O(10)-S(4)-C(27)	102.5(4)	C(42)-C(41A)-C(44)	129.8(5)
O(12)-S(4)-C(27)	102.8(4)	N(5)-C(43A)-H(43D)	109.5
O(15)-S(5)-O(13)	116.0(3)	N(5)-C(43A)-H(43E)	109.5
O(15)-S(5)-O(14)	115.4(3)	H(43D)-C(43A)-H(43E)	109.5
O(13)-S(5)-O(14)	113.7(3)	N(5)-C(43A)-H(43F)	109.5
O(15)-S(5)-C(28)	102.5(3)	H(43D)-C(43A)-H(43F)	109.5
O(13)-S(5)-C(28)	103.3(3)	H(43E)-C(43A)-H(43F)	109.5
O(14)-S(5)-C(28)	103.5(3)		

Table S34. Anisotropic displacement parameters ($Å^2$x 10^3) for **10a**. The anisotropic displacement factor exponent takes the form: $-2p^2 [h^2 a^{*2}U^{11} + ... + 2 h k a^* b^* U^{12}]$.

	U^{11}	U^{22}	U^{33}	U^{23}	U^{13}	U^{12}
Co(1)	32(1)	77(1)	40(1)	-15(1)	12(1)	-2(1)
Co(2)	26(1)	57(1)	41(1)	5(1)	10(1)	6(1)
Co(3)	26(1)	74(1)	40(1)	16(1)	10(1)	6(1)
Co(4)	28(1)	43(1)	43(1)	4(1)	13(1)	1(1)
N(1)	27(2)	36(3)	48(3)	1(2)	8(2)	2(2)
N(2)	33(2)	41(3)	56(3)	5(2)	14(2)	6(2)
N(3)	35(3)	34(3)	53(3)	7(2)	3(2)	2(2)

N(5)	27(3)	33(3)	177(8)	-26(4)	9(4)	2(2)
N(6)	24(3)	51(4)	160(7)	-43(4)	3(4)	6(3)
C(1)	89(9)	102(8)	214(16)	-55(11)	-79(10)	47(7)
C(2)	43(6)	450(40)	163(15)	-210(20)	-6(8)	60(12)
C(3)	64(7)	370(20)	63(7)	-8(11)	18(5)	-101(11)
C(4)	41(4)	88(6)	82(6)	-15(5)	-12(4)	-12(4)
C(5)	64(5)	95(7)	65(5)	-2(5)	-10(4)	5(5)
C(6)	54(4)	65(4)	59(4)	-21(3)	27(3)	-11(3)
C(7)	52(4)	103(6)	63(5)	-44(5)	28(4)	-31(4)
C(8)	48(4)	129(8)	41(4)	-11(4)	16(3)	-8(4)
C(9)	41(3)	88(5)	43(4)	13(3)	12(3)	1(3)
C(10)	32(3)	60(4)	38(3)	-3(3)	8(2)	-6(3)
C(11)	20(2)	49(3)	38(3)	6(2)	6(2)	0(2)
C(12)	28(3)	34(3)	45(3)	0(2)	12(2)	1(2)
C(13)	60(4)	51(4)	68(4)	15(3)	20(4)	3(3)
C(14)	26(3)	50(3)	36(3)	-3(3)	6(2)	5(2)
C(15)	33(3)	55(4)	54(4)	-11(3)	13(3)	2(3)
C(16)	41(4)	96(6)	42(4)	-13(4)	12(3)	5(3)
C(17)	36(3)	83(5)	40(3)	10(3)	7(3)	5(3)
C(18)	29(3)	57(4)	39(3)	9(3)	6(2)	8(3)
C(19)	32(3)	72(5)	50(4)	15(3)	9(3)	10(3)
C(20)	39(3)	81(5)	67(4)	18(4)	23(3)	19(3)
C(21)	34(3)	98(6)	73(5)	17(4)	21(3)	3(4)
C(22)	38(4)	57(4)	96(6)	14(4)	-9(4)	-10(3)
C(23)	35(3)	75(5)	50(4)	1(3)	-1(3)	10(3)
C(31)	81(8)	76(6)	205(14)	56(9)	-90(9)	-41(6)
C(32)	23(4)	400(30)	146(13)	196(18)	5(6)	-12(9)
C(33)	61(6)	321(19)	49(6)	-17(8)	0(5)	110(10)
C(34)	32(3)	60(4)	77(5)	5(4)	-7(3)	10(3)
C(35)	66(5)	88(6)	47(4)	4(4)	-2(4)	16(4)
C(36)	36(3)	65(4)	54(4)	9(3)	14(3)	13(3)
C(37)	37(3)	80(5)	56(4)	26(4)	14(3)	20(3)
C(38)	37(3)	93(5)	42(4)	20(4)	12(3)	18(3)
C(39)	29(3)	73(4)	40(3)	0(3)	3(3)	9(3)
C(40)	26(3)	62(4)	40(3)	3(3)	7(2)	10(3)
C(42)	31(3)	38(3)	50(3)	4(3)	11(3)	9(2)
C(44)	27(3)	42(3)	52(4)	11(3)	9(3)	3(2)
C(45)	49(4)	48(4)	65(4)	21(3)	25(3)	16(3)
C(46)	53(4)	62(4)	53(4)	24(3)	16(3)	11(3)
C(47)	37(3)	77(5)	40(3)	0(3)	3(3)	4(3)
C(48)	31(3)	47(3)	49(4)	8(3)	5(3)	0(2)
C(49)	38(3)	80(5)	60(4)	-13(4)	15(3)	-15(3)
C(50)	43(4)	91(6)	63(4)	1(4)	26(3)	-17(4)
C(51)	37(3)	87(6)	71(5)	-1(4)	24(3)	6(3)
C(52)	23(3)	69(4)	75(5)	10(4)	7(3)	14(3)
C(53)	29(3)	86(5)	53(4)	16(4)	8(3)	-3(3)
C(24)	75(5)	104(6)	57(5)	26(4)	32(4)	21(5)
C(25)	154(11)	96(8)	77(7)	1(6)	11(7)	11(8)
C(26)	42(4)	92(6)	63(5)	33(4)	7(3)	-1(4)
C(27)	80(6)	73(6)	63(5)	-5(4)	7(4)	-5(4)

C(28)	50(4)	63(4)	43(4)	5(3)	8(3)	13(3)
C(29)	90(7)	130(10)	104(8)	37(7)	6(6)	-22(7)
C(54)	72(5)	85(6)	114(7)	50(5)	34(5)	36(5)
C(55)	172(12)	94(8)	138(10)	9(7)	93(9)	15(7)
C(56)	91(8)	217(14)	94(8)	33(8)	36(6)	21(8)
C(57)	64(5)	92(7)	118(8)	-60(6)	7(5)	-8(5)
C(58)	97(7)	106(7)	74(6)	1(5)	42(5)	-2(6)
C(59)	72(6)	193(12)	97(7)	-44(7)	8(5)	27(7)
O(20)	120(7)	95(6)	394(17)	-117(8)	-85(8)	12(5)
O(19)	104(6)	103(6)	274(12)	62(6)	-3(6)	41(5)
S(1)	49(1)	83(1)	48(1)	10(1)	24(1)	-10(1)
S(2)	88(2)	97(2)	80(2)	-8(1)	39(1)	6(1)
S(3)	36(1)	51(1)	44(1)	6(1)	12(1)	7(1)
S(4)	63(1)	66(1)	81(1)	13(1)	1(1)	-19(1)
S(5)	40(1)	56(1)	39(1)	10(1)	12(1)	12(1)
S(6)	58(1)	78(1)	73(1)	7(1)	29(1)	3(1)
O(1)	99(5)	120(6)	288(11)	134(7)	106(6)	60(4)
O(2)	123(6)	253(9)	70(4)	-72(5)	65(4)	-92(6)
O(3)	96(4)	75(3)	84(4)	-21(3)	67(3)	-35(3)
O(4)	197(10)	413(17)	80(5)	86(8)	78(6)	153(11)
O(5)	146(8)	100(6)	279(12)	60(7)	-72(8)	-56(5)
O(6)	160(8)	132(6)	168(8)	-34(6)	67(6)	39(6)
O(7)	78(3)	53(3)	79(3)	2(2)	41(3)	5(2)
O(8)	52(3)	78(3)	60(3)	6(2)	28(2)	17(2)
O(9)	55(3)	64(3)	42(2)	19(2)	11(2)	7(2)
O(10)	74(4)	128(6)	154(6)	37(5)	28(4)	-40(4)
O(11)	78(3)	56(3)	86(4)	12(3)	24(3)	-2(3)
O(12)	131(5)	71(4)	69(4)	-11(3)	-21(3)	-11(3)
O(13)	66(3)	86(3)	44(3)	27(2)	13(2)	12(3)
O(14)	76(3)	89(4)	70(3)	6(3)	40(3)	25(3)
O(15)	52(3)	62(3)	77(3)	2(2)	10(2)	3(2)
O(16)	94(5)	241(10)	97(5)	84(6)	7(4)	0(5)
O(17)	340(14)	94(5)	297(12)	-5(6)	280(12)	27(7)
O(18)	95(4)	67(4)	137(5)	-7(3)	44(4)	-28(3)
F(1)	185(8)	190(8)	322(13)	152(9)	104(8)	119(7)
F(2)	98(5)	499(17)	53(4)	-60(6)	23(3)	-62(7)
F(3)	52(3)	163(5)	89(3)	-1(3)	33(2)	-20(3)
F(4)	276(11)	239(10)	86(5)	57(6)	-18(6)	-36(8)
F(5)	124(6)	133(6)	225(9)	34(6)	7(5)	-45(5)
F(6)	133(6)	200(8)	221(9)	-75(7)	-47(6)	83(6)
F(7)	124(5)	101(4)	85(4)	14(3)	-28(3)	-48(3)
F(8)	43(2)	202(6)	112(4)	58(4)	31(3)	20(3)
F(9)	60(3)	145(4)	67(3)	55(3)	3(2)	9(3)
F(10)	66(3)	203(7)	108(4)	11(4)	20(3)	8(4)
F(11)	151(6)	94(4)	113(4)	23(3)	-9(4)	22(4)
F(12)	122(4)	120(4)	68(3)	-16(3)	3(3)	4(3)
F(13)	84(4)	194(6)	89(4)	87(4)	-2(3)	18(4)
F(14)	45(2)	161(5)	101(4)	-47(3)	10(2)	31(3)
F(15)	60(3)	78(3)	122(4)	-17(3)	-9(3)	4(2)
F(16)	108(6)	449(18)	233(10)	202(12)	-57(7)	-55(8)

F(17)	192(9)	189(9)	407(17)	76(9)	213(11)	88(7)
F(18)	129(6)	168(7)	254(10)	11(6)	51(6)	-89(5)
N(4)	25(2)	30(3)	63(3)	7(2)	11(2)	5(2)
C(41)	24(2)	40(3)	57(3)	-10(2)	9(2)	4(2)
C(43)	56(7)	45(6)	60(7)	-9(5)	27(6)	5(5)
N(4A)	24(2)	40(3)	57(3)	-10(2)	9(2)	4(2)
C(41A)	25(2)	30(3)	63(3)	7(2)	11(2)	5(2)
C(43A)	76(10)	30(7)	65(9)	3(6)	53(8)	5(6)

Table S35. Hydrogen coordinates (x 10^4) and isotropic displacement parameters (Å^{2}x 10^3) for **10a**.

	x	y	z	U(eq)
H(1)	8449	6566	3511	196
H(2)	8902	7151	2416	277
H(3)	8781	8672	2503	209
H(4)	8155	8957	3606	95
H(5)	7911	7677	4209	98
H(6)	5650	6467	2795	71
H(7)	6349	6331	1656	88
H(8)	6467	7714	1135	89
H(9)	5845	8708	1916	70
H(12)	4728	6990	3694	43
H(13A)	5214	10041	3337	90
H(13B)	4918	9571	2510	90
H(13C)	6148	9624	3098	90
H(15)	4009	9196	5146	57
H(16)	3441	8522	6206	73
H(17)	3454	7000	6028	65
H(18)	4032	6709	4845	51
H(19)	1609	8867	3693	62
H(20)	1039	8771	4890	72
H(21)	795	7294	5157	81
H(22)	1247	6455	4142	86
H(23)	1774	7426	3236	68
H(31)	8754	8912	8571	185
H(32)	9173	8299	7465	234
H(33)	8829	6792	7468	171
H(34)	8080	6488	8543	74
H(35)	8051	7797	9212	86
H(36)	5895	8722	7934	62
H(37)	6629	9029	6825	68
H(38)	6623	7692	6121	68
H(39)	5899	6539	6786	59

H(42)	4779	7958	8698	48
H(45)	3755	5517	9778	61
H(46)	3266	6035	10923	66
H(47)	3493	7577	10987	64
H(48)	4152	8024	9882	53
H(49)	1220	5674	8520	74
H(50)	741	5910	9741	78
H(51)	930	7431	10043	76
H(52)	1550	8124	9022	68
H(53)	1752	7042	8091	69
H(55A)	6117	4745	5154	186
H(55B)	6780	4765	4547	186
H(55C)	5527	4977	4289	186
H(56A)	7912	6689	5497	197
H(56B)	8226	5934	5068	197
H(56C)	7953	5811	5853	197
H(58A)	5410	10170	9262	135
H(58B)	6636	10609	9446	135
H(58C)	6095	10519	10119	135
H(59A)	7977	8922	10326	188
H(59B)	7823	9676	10840	188
H(59C)	8230	9839	10112	188
H(43A)	5431	5099	8264	77
H(43B)	6210	5591	7861	77
H(43C)	4944	5319	7384	77
H(43D)	3790	5051	8444	75
H(43E)	4983	5024	9066	75
H(43F)	4857	5051	8171	75

Table S36. Torsion angles [°] for **10a**.

C(12)-N(1)-N(2)-N(3)	1.5(6)	C(1)-C(2)-C(3)-Co(1)	-63.3(10)
C(14)-N(1)-N(2)-N(3)	177.0(4)	C(2)-C(3)-C(4)-C(5)	0.4(12)
N(1)-N(2)-N(3)-C(11)	-1.5(6)	Co(1)-C(3)-C(4)-C(5)	59.0(7)
N(1)-N(2)-N(3)-C(13)	179.6(5)	C(2)-C(3)-C(4)-Co(1)	-58.5(8)
C(41A)-N(5)-N(6)-N(4A)	-2.2(8)	C(3)-C(4)-C(5)-C(1)	1.1(10)
C(43A)-N(5)-N(6)-N(4A)	173.2(8)	Co(1)-C(4)-C(5)-C(1)	59.7(6)
N(4)-N(5)-N(6)-C(41)	-2.2(8)	C(3)-C(4)-C(5)-Co(1)	-58.6(6)
N(4)-N(5)-N(6)-C(43)	173.5(6)	C(2)-C(1)-C(5)-C(4)	-2.3(13)
C(5)-C(1)-C(2)-C(3)	2.5(16)	Co(1)-C(1)-C(5)-C(4)	-60.6(6)
Co(1)-C(1)-C(2)-C(3)	62.6(10)	C(2)-C(1)-C(5)-Co(1)	58.3(9)
C(5)-C(1)-C(2)-Co(1)	-60.1(8)	C(10)-C(6)-C(7)-C(8)	0.8(8)
C(1)-C(2)-C(3)-C(4)	-1.8(16)	Co(1)-C(6)-C(7)-C(8)	-59.3(5)
Co(1)-C(2)-C(3)-C(4)	61.4(8)	C(10)-C(6)-C(7)-Co(1)	60.1(4)

C(6)-C(7)-C(8)-C(9)	-0.2(8)	C(15)-C(14)-C(18)-Co(2)	58.8(4)
Co(1)-C(7)-C(8)-C(9)	-59.3(5)	N(1)-C(14)-C(18)-Co(2)	-121.3(5)
C(6)-C(7)-C(8)-Co(1)	59.0(5)	C(16)-C(17)-C(18)-C(14)	0.7(6)
C(7)-C(8)-C(9)-C(10)	-0.4(8)	Co(2)-C(17)-C(18)-C(14)	60.1(4)
Co(1)-C(8)-C(9)-C(10)	-59.7(4)	C(16)-C(17)-C(18)-Co(2)	-59.4(4)
C(7)-C(8)-C(9)-Co(1)	59.3(5)	C(23)-C(19)-C(20)-C(21)	-1.0(7)
C(7)-C(6)-C(10)-C(9)	-1.0(7)	Co(2)-C(19)-C(20)-C(21)	-59.3(4)
Co(1)-C(6)-C(10)-C(9)	58.5(4)	C(23)-C(19)-C(20)-Co(2)	58.4(4)
C(7)-C(6)-C(10)-C(11)	179.9(5)	C(19)-C(20)-C(21)-C(22)	0.6(7)
Co(1)-C(6)-C(10)-C(11)	-120.6(5)	Co(2)-C(20)-C(21)-C(22)	-58.9(5)
C(7)-C(6)-C(10)-Co(1)	-59.5(5)	C(19)-C(20)-C(21)-Co(2)	59.5(4)
C(8)-C(9)-C(10)-C(6)	0.9(7)	C(20)-C(21)-C(22)-C(23)	-0.1(7)
Co(1)-C(9)-C(10)-C(6)	-58.5(4)	Co(2)-C(21)-C(22)-C(23)	-59.4(4)
C(8)-C(9)-C(10)-C(11)	179.9(6)	C(20)-C(21)-C(22)-Co(2)	59.4(5)
Co(1)-C(9)-C(10)-C(11)	120.5(6)	C(21)-C(22)-C(23)-C(19)	-0.5(7)
C(8)-C(9)-C(10)-Co(1)	59.3(5)	Co(2)-C(22)-C(23)-C(19)	-59.9(4)
N(2)-N(3)-C(11)-C(12)	1.0(6)	C(21)-C(22)-C(23)-Co(2)	59.4(5)
C(13)-N(3)-C(11)-C(12)	179.7(5)	C(20)-C(19)-C(23)-C(22)	0.9(7)
N(2)-N(3)-C(11)-C(10)	-177.5(5)	Co(2)-C(19)-C(23)-C(22)	59.3(4)
C(13)-N(3)-C(11)-C(10)	1.3(9)	C(20)-C(19)-C(23)-Co(2)	-58.4(4)
C(6)-C(10)-C(11)-C(12)	-7.6(9)	C(35)-C(31)-C(32)-C(33)	-1.9(13)
C(9)-C(10)-C(11)-C(12)	173.5(6)	Co(3)-C(31)-C(32)-C(33)	-62.5(8)
Co(1)-C(10)-C(11)-C(12)	-94.1(7)	C(35)-C(31)-C(32)-Co(3)	60.6(7)
C(6)-C(10)-C(11)-N(3)	170.6(5)	C(31)-C(32)-C(33)-C(34)	2.0(13)
C(9)-C(10)-C(11)-N(3)	-8.3(9)	Co(3)-C(32)-C(33)-C(34)	-59.8(6)
Co(1)-C(10)-C(11)-N(3)	84.0(7)	C(31)-C(32)-C(33)-Co(3)	61.8(9)
N(2)-N(1)-C(12)-C(11)	-0.9(6)	C(32)-C(33)-C(34)-C(35)	-1.4(10)
C(14)-N(1)-C(12)-C(11)	-175.8(5)	Co(3)-C(33)-C(34)-C(35)	-59.7(5)
N(3)-C(11)-C(12)-N(1)	-0.1(5)	C(32)-C(33)-C(34)-Co(3)	58.4(7)
C(10)-C(11)-C(12)-N(1)	178.4(5)	C(33)-C(34)-C(35)-C(31)	0.2(8)
N(2)-N(1)-C(14)-C(18)	-177.2(5)	Co(3)-C(34)-C(35)-C(31)	-59.7(6)
C(12)-N(1)-C(14)-C(18)	-2.4(8)	C(33)-C(34)-C(35)-Co(3)	59.9(5)
N(2)-N(1)-C(14)-C(15)	2.7(7)	C(32)-C(31)-C(35)-C(34)	1.0(11)
C(12)-N(1)-C(14)-C(15)	177.4(5)	Co(3)-C(31)-C(35)-C(34)	60.6(6)
N(2)-N(1)-C(14)-Co(2)	92.8(5)	C(32)-C(31)-C(35)-Co(3)	-59.6(8)
C(12)-N(1)-C(14)-Co(2)	-92.4(6)	C(40)-C(36)-C(37)-C(38)	-0.9(6)
C(18)-C(14)-C(15)-C(16)	1.1(6)	Co(3)-C(36)-C(37)-C(38)	58.8(4)
N(1)-C(14)-C(15)-C(16)	-178.8(5)	C(40)-C(36)-C(37)-Co(3)	-59.7(4)
Co(2)-C(14)-C(15)-C(16)	59.7(4)	C(36)-C(37)-C(38)-C(39)	-0.2(7)
C(18)-C(14)-C(15)-Co(2)	-58.5(4)	Co(3)-C(37)-C(38)-C(39)	58.4(4)
N(1)-C(14)-C(15)-Co(2)	121.6(5)	C(36)-C(37)-C(38)-Co(3)	-58.6(4)
C(14)-C(15)-C(16)-C(17)	-0.6(7)	C(37)-C(38)-C(39)-C(40)	1.2(6)
Co(2)-C(15)-C(16)-C(17)	58.6(4)	Co(3)-C(38)-C(39)-C(40)	60.2(4)
C(14)-C(15)-C(16)-Co(2)	-59.2(4)	C(37)-C(38)-C(39)-Co(3)	-59.0(4)
C(15)-C(16)-C(17)-C(18)	0.0(7)	C(37)-C(36)-C(40)-C(39)	1.7(6)
Co(2)-C(16)-C(17)-C(18)	59.1(4)	Co(3)-C(36)-C(40)-C(39)	-58.2(4)
C(15)-C(16)-C(17)-Co(2)	-59.1(4)	C(37)-C(36)-C(40)-N(4A)	-178.9(5)
C(15)-C(14)-C(18)-C(17)	-1.1(6)	Co(3)-C(36)-C(40)-N(4A)	121.3(5)
N(1)-C(14)-C(18)-C(17)	178.8(5)	C(37)-C(36)-C(40)-C(41)	-178.9(5)
Co(2)-C(14)-C(18)-C(17)	-60.0(4)	Co(3)-C(36)-C(40)-C(41)	121.3(5)

C(37)-C(36)-C(40)-Co(3)	59.9(4)	F(3)-C(24)-S(1)-O(2)	65.2(8)
C(38)-C(39)-C(40)-C(36)	-1.8(6)	F(1)-C(24)-S(1)-O(2)	-50.4(8)
Co(3)-C(39)-C(40)-C(36)	58.4(4)	F(2)-C(24)-S(1)-O(3)	-49.7(9)
C(38)-C(39)-C(40)-N(4A)	178.8(5)	F(3)-C(24)-S(1)-O(3)	-175.5(6)
Co(3)-C(39)-C(40)-N(4A)	-121.1(5)	F(1)-C(24)-S(1)-O(3)	68.9(7)
C(38)-C(39)-C(40)-C(41)	178.8(5)	F(6)-C(25)-S(2)-O(5)	-55.7(13)
Co(3)-C(39)-C(40)-C(41)	-121.1(5)	F(5)-C(25)-S(2)-O(5)	174.4(11)
C(38)-C(39)-C(40)-Co(3)	-60.1(4)	F(4)-C(25)-S(2)-O(5)	64.0(11)
C(48)-C(44)-C(45)-C(46)	-1.7(7)	F(6)-C(25)-S(2)-O(6)	172.3(10)
C(41A)-C(44)-C(45)-C(46)	176.7(5)	F(5)-C(25)-S(2)-O(6)	42.4(12)
N(4)-C(44)-C(45)-C(46)	176.7(5)	F(4)-C(25)-S(2)-O(6)	-68.0(10)
Co(4)-C(44)-C(45)-C(46)	-59.3(4)	F(6)-C(25)-S(2)-O(4)	62.4(12)
C(48)-C(44)-C(45)-Co(4)	57.7(4)	F(5)-C(25)-S(2)-O(4)	-67.5(11)
C(41A)-C(44)-C(45)-Co(4)	-124.0(6)	F(4)-C(25)-S(2)-O(4)	-177.8(9)
N(4)-C(44)-C(45)-Co(4)	-124.0(6)	F(7)-C(26)-S(3)-O(7)	-179.1(5)
C(44)-C(45)-C(46)-C(47)	1.3(7)	F(9)-C(26)-S(3)-O(7)	60.6(7)
Co(4)-C(45)-C(46)-C(47)	-58.8(4)	F(8)-C(26)-S(3)-O(7)	-58.7(6)
C(44)-C(45)-C(46)-Co(4)	60.1(4)	F(7)-C(26)-S(3)-O(8)	60.8(6)
C(45)-C(46)-C(47)-C(48)	-0.4(7)	F(9)-C(26)-S(3)-O(8)	-59.5(7)
Co(4)-C(46)-C(47)-C(48)	-59.9(4)	F(8)-C(26)-S(3)-O(8)	-178.8(5)
C(45)-C(46)-C(47)-Co(4)	59.5(4)	F(7)-C(26)-S(3)-O(9)	-58.9(6)
C(46)-C(47)-C(48)-C(44)	-0.6(6)	F(9)-C(26)-S(3)-O(9)	-179.3(6)
Co(4)-C(47)-C(48)-C(44)	-60.3(4)	F(8)-C(26)-S(3)-O(9)	61.4(6)
C(46)-C(47)-C(48)-Co(4)	59.7(4)	F(10)-C(27)-S(4)-O(11)	-60.2(7)
C(45)-C(44)-C(48)-C(47)	1.4(6)	F(11)-C(27)-S(4)-O(11)	177.7(7)
C(41A)-C(44)-C(48)-C(47)	-177.0(5)	F(12)-C(27)-S(4)-O(11)	59.7(7)
N(4)-C(44)-C(48)-C(47)	-177.0(5)	F(10)-C(27)-S(4)-O(10)	178.8(7)
Co(4)-C(44)-C(48)-C(47)	59.5(4)	F(11)-C(27)-S(4)-O(10)	56.7(8)
C(45)-C(44)-C(48)-Co(4)	-58.1(4)	F(12)-C(27)-S(4)-O(10)	-61.3(7)
C(41A)-C(44)-C(48)-Co(4)	123.5(5)	F(10)-C(27)-S(4)-O(12)	59.5(7)
N(4)-C(44)-C(48)-Co(4)	123.5(5)	F(11)-C(27)-S(4)-O(12)	-62.6(8)
C(53)-C(49)-C(50)-C(51)	0.9(8)	F(12)-C(27)-S(4)-O(12)	179.4(6)
Co(4)-C(49)-C(50)-C(51)	60.0(5)	F(13)-C(28)-S(5)-O(15)	-58.0(6)
C(53)-C(49)-C(50)-Co(4)	-59.1(4)	F(14)-C(28)-S(5)-O(15)	61.1(6)
C(49)-C(50)-C(51)-C(52)	-0.5(7)	F(15)-C(28)-S(5)-O(15)	-178.6(5)
Co(4)-C(50)-C(51)-C(52)	59.2(4)	F(13)-C(28)-S(5)-O(13)	-178.9(5)
C(49)-C(50)-C(51)-Co(4)	-59.7(5)	F(14)-C(28)-S(5)-O(13)	-59.7(6)
C(50)-C(51)-C(52)-C(53)	-0.1(7)	F(15)-C(28)-S(5)-O(13)	60.5(6)
Co(4)-C(51)-C(52)-C(53)	59.1(4)	F(13)-C(28)-S(5)-O(14)	62.3(6)
C(50)-C(51)-C(52)-Co(4)	-59.2(5)	F(14)-C(28)-S(5)-O(14)	-178.5(5)
C(50)-C(49)-C(53)-C(52)	-0.9(7)	F(15)-C(28)-S(5)-O(14)	-58.2(6)
Co(4)-C(49)-C(53)-C(52)	-59.8(4)	F(18)-C(29)-S(6)-O(17)	61.2(11)
C(50)-C(49)-C(53)-Co(4)	58.8(5)	F(16)-C(29)-S(6)-O(17)	-65.0(13)
C(51)-C(52)-C(53)-C(49)	0.6(7)	F(17)-C(29)-S(6)-O(17)	179.4(9)
Co(4)-C(52)-C(53)-C(49)	60.0(4)	F(18)-C(29)-S(6)-O(16)	-55.1(12)
C(51)-C(52)-C(53)-Co(4)	-59.3(4)	F(16)-C(29)-S(6)-O(16)	178.7(12)
F(2)-C(24)-S(1)-O(1)	69.5(9)	F(17)-C(29)-S(6)-O(16)	63.1(10)
F(3)-C(24)-S(1)-O(1)	-56.3(8)	F(18)-C(29)-S(6)-O(18)	-179.6(9)
F(1)-C(24)-S(1)-O(1)	-171.9(7)	F(16)-C(29)-S(6)-O(18)	54.2(13)
F(2)-C(24)-S(1)-O(2)	-169.0(9)	F(17)-C(29)-S(6)-O(18)	-61.4(9)

N(6)-N(5)-N(4)-C(42)	1.9(7)	N(5)-N(6)-N(4A)-C(42)	1.6(7)
N(6)-N(5)-N(4)-C(44)	-174.2(5)	N(5)-N(6)-N(4A)-C(40)	-179.8(5)
C(41)-C(42)-N(4)-N(5)	-0.9(6)	C(41A)-C(42)-N(4A)-N(6)	-0.4(6)
C(41)-C(42)-N(4)-C(44)	174.8(5)	C(41A)-C(42)-N(4A)-C(40)	-178.8(5)
C(45)-C(44)-N(4)-N(5)	-5.4(9)	C(36)-C(40)-N(4A)-N(6)	-167.8(5)
C(48)-C(44)-N(4)-N(5)	172.7(5)	C(39)-C(40)-N(4A)-N(6)	11.5(9)
Co(4)-C(44)-N(4)-N(5)	-98.0(6)	Co(3)-C(40)-N(4A)-N(6)	-78.9(7)
C(45)-C(44)-N(4)-C(42)	179.5(6)	C(36)-C(40)-N(4A)-C(42)	10.3(9)
C(48)-C(44)-N(4)-C(42)	-2.4(9)	C(39)-C(40)-N(4A)-C(42)	-170.3(5)
Co(4)-C(44)-N(4)-C(42)	86.9(7)	Co(3)-C(40)-N(4A)-C(42)	99.2(6)
N(5)-N(6)-C(41)-C(42)	1.6(7)	N(6)-N(5)-C(41A)-C(42)	1.9(7)
C(43)-N(6)-C(41)-C(42)	-171.2(11)	C(43A)-N(5)-C(41A)-C(42)	-169.6(16)
N(5)-N(6)-C(41)-C(40)	-179.8(5)	N(6)-N(5)-C(41A)-C(44)	-174.2(5)
C(43)-N(6)-C(41)-C(40)	7.3(15)	C(43A)-N(5)-C(41A)-C(44)	14.4(19)
N(4)-C(42)-C(41)-N(6)	-0.4(6)	N(4A)-C(42)-C(41A)-N(5)	-0.9(6)
N(4)-C(42)-C(41)-C(40)	-178.8(5)	N(4A)-C(42)-C(41A)-C(44)	174.8(5)
C(36)-C(40)-C(41)-N(6)	-167.8(5)	C(45)-C(44)-C(41A)-N(5)	-5.4(9)
C(39)-C(40)-C(41)-N(6)	11.5(9)	C(48)-C(44)-C(41A)-N(5)	172.7(5)
Co(3)-C(40)-C(41)-N(6)	-78.9(7)	Co(4)-C(44)-C(41A)-N(5)	-98.0(6)
C(36)-C(40)-C(41)-C(42)	10.3(9)	C(45)-C(44)-C(41A)-C(42)	179.5(6)
C(39)-C(40)-C(41)-C(42)	-170.3(5)	C(48)-C(44)-C(41A)-C(42)	-2.4(9)
Co(3)-C(40)-C(41)-C(42)	99.2(6)	Co(4)-C(44)-C(41A)-C(42)	86.9(7)

Triazolium salt (10b) – “1,4-dicobaltoceniumyl-3-methyltriazolium tris(hexafluorido-phosphate)“

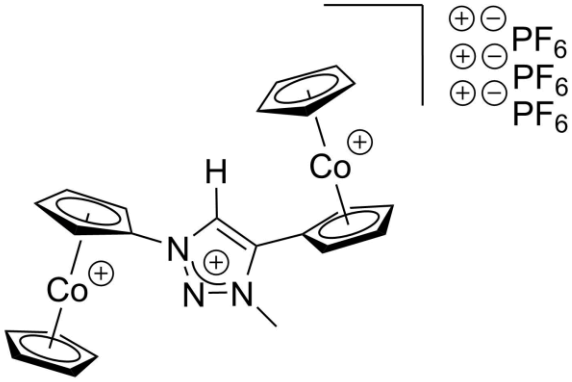

[893.20]

$C_{23}H_{22}N_3Co_2(PF_6)_3$

Analytical data:

IR (ATR [cm^{-1}]): 3127 (ν_{C-H}), 2921, 2851, 1611, 1529, 1469, 1418 ($\nu_{C=C}$), 817 (ν_{P-F}), 554 (ν_{P-F}), 499, 473, 437.

^{1}H-NMR (300 MHz, C_3D_6O, [ppm]): δ 4.71 (s, 3H, CH_3 of triazolium), 6.12 (s, 5H, 4-Cc-Cp), 6.18 (s, 5H, 1-Cc-Cp), 6.29 (pseudo-t, 2H, *J* = 2.3 Hz, C3/C4 of substituted 1-Cc-Cp), 6.36 (pseudo-t, 2H, *J* = 2.3 Hz, C3/C4 of substituted 4-Cc-Cp), 6.61 (pseudo-t, 2H, *J* = 2.3 Hz, C2/C5 of substituted 4-Cc-Cp), 6.83 (pseudo-t, 2H, J = 2.4 Hz, C2/C5 of substituted 1-Cc-Cp), 9.86 (s, 1H, CH of triazolium).

^{13}C-NMR (75 MHz, C_3D_6O, [ppm]): δ 41.5 (CH_3 of triazolium), 80.0 (C3/C4 of substituted 4-Cc-Cp), 85.2 (C3/C4 of substituted 1-Cc-Cp), 85.5 (quart. carbon of substituted 4-Cc-Cp), 86.1 (C2/C5 of substituted 4-Cc-Cp), 87.8 (C2/C5 of substituted 1-Cc-Cp), 88.4 (4-Cc-Cp), 88.8 (1-Cc-Cp), 104.9 (quart. carbon of substituted 1-Cc-Cp), 132.5 (CH of triazolium), 138.6 (quart. carbon of triazolium).

MS (ESI pos, [m/z]): 747.97 ($[M(PF_6)_2]^+$).

Melting point [°C]: 218.

Spectra

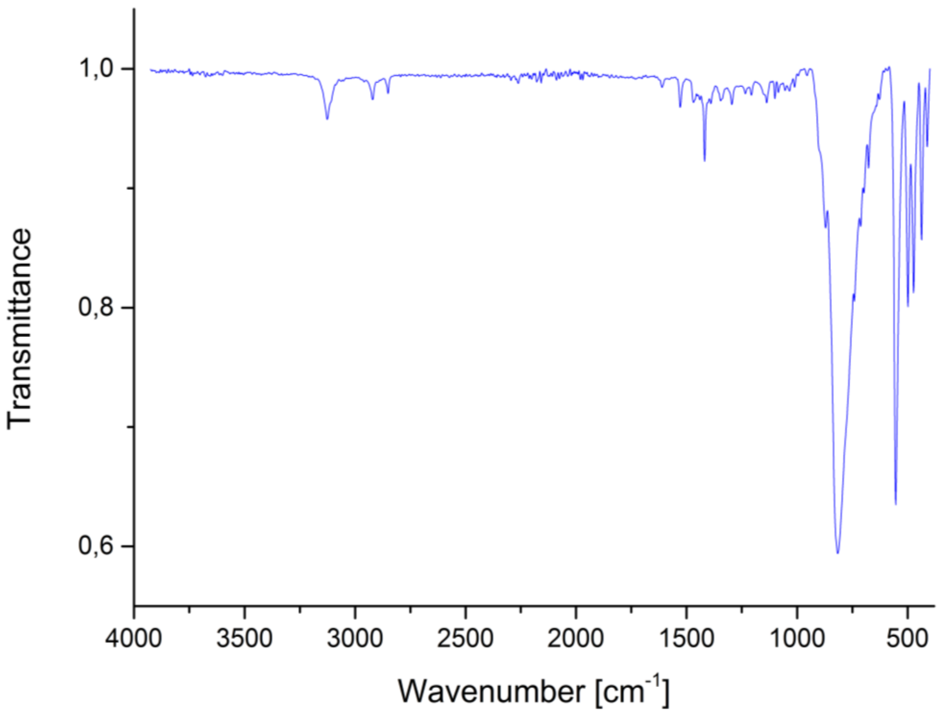

Figure S39. IR-spectrum (ATR, [cm^{-1}]) of “1,4-dicobaltoceniumyl-3-methyltriazolium tris(hexafluoridophosphate)”.

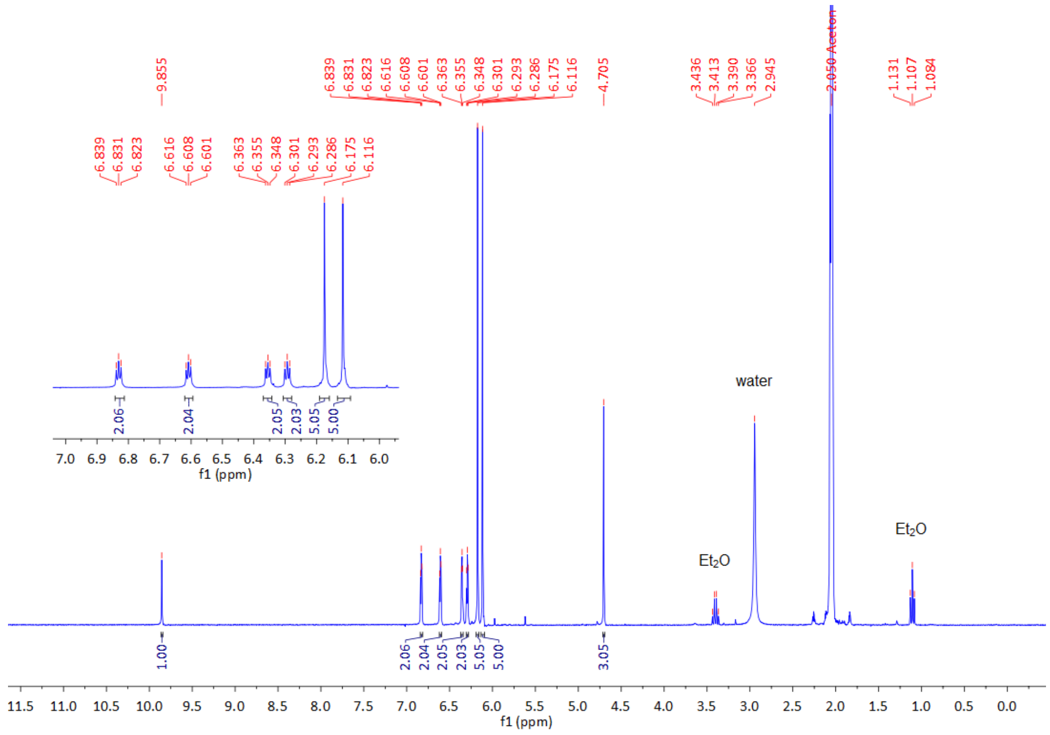

Figure S40. ^{1}H-NMR (300 MHz, C_3D_6O, [ppm]) of “1,4-dicobaltoceniumyl-3-methyltriazolium tris(hexafluoridophosphate)“.

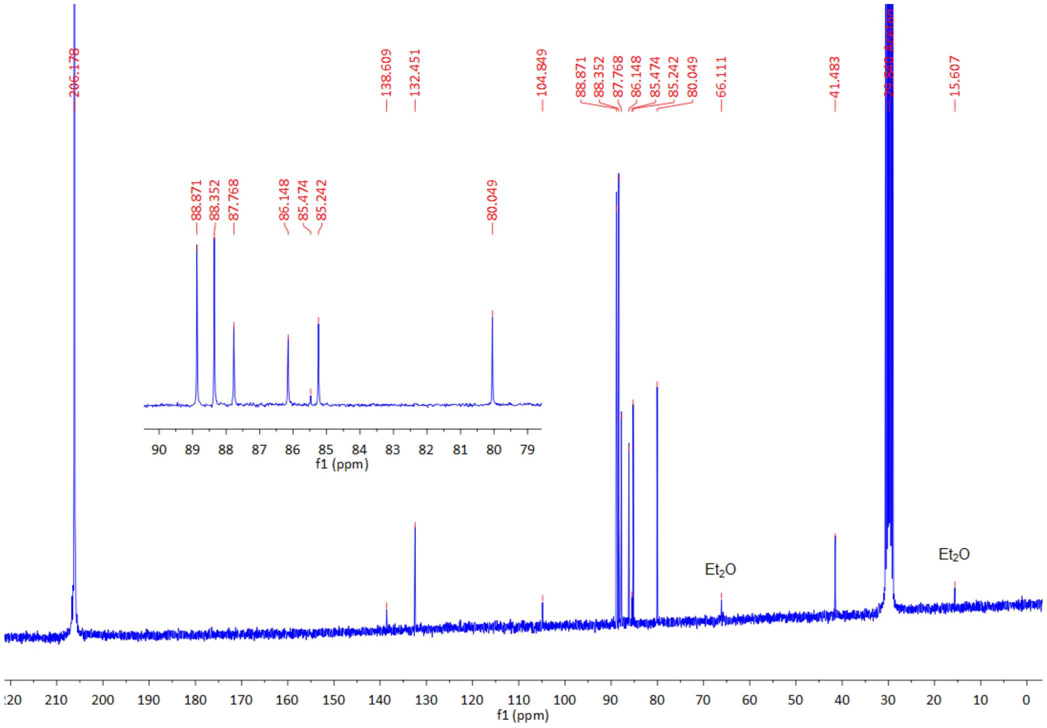

Figure S41. ^{13}C-NMR (75 MHz, C_3D_6O, [ppm]) of “1,4-dicobaltoceniumyl-3-methyltriazolium tris(hexafluoridophosphate)“.

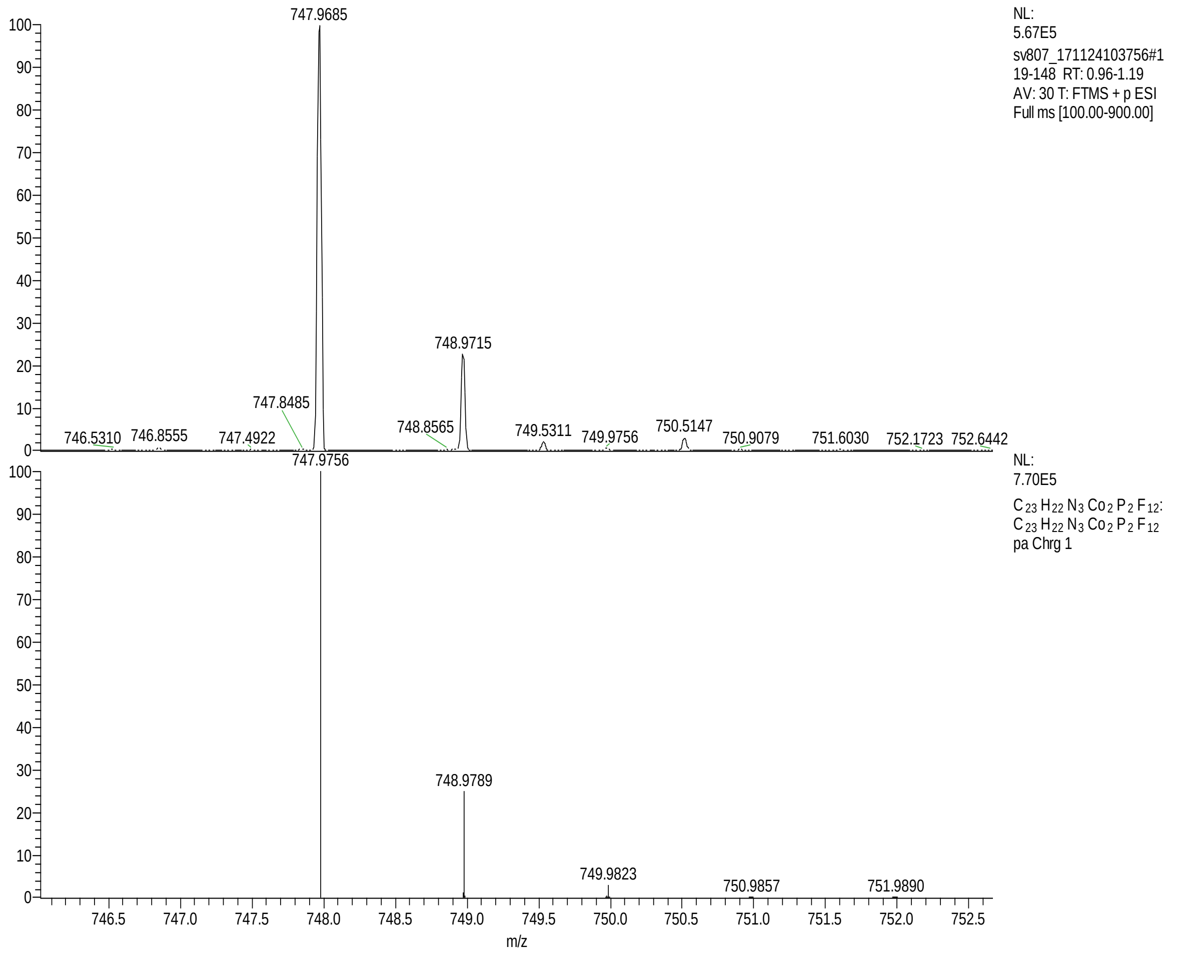

Figure S42. MS (MALDI pos, [m/z]; *top*: experimental, *bottom*: simulated) of "1,4-dicobaltoceniumyl-3-methyltriazolium tris(hexafluoridophosphate)".

Triazolylidene complex (11) – "chlorido (4-cobaltoceniumyl-1-ferrocenyl-3-methyltriazolylidene) silver(I) triflate"

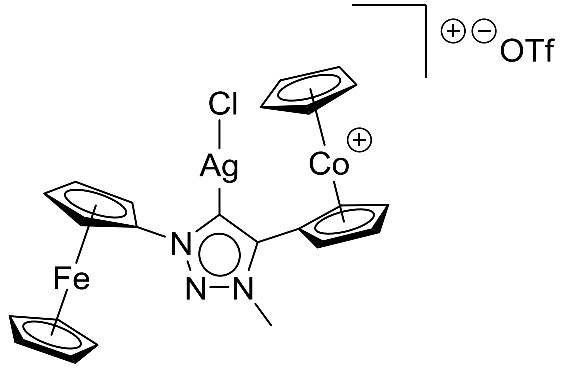

[746.61]

$C_{23}H_{21}N_3CoFeAgCl(CF_3SO_3)$

Analytical data:

IR (ATR [cm^{-1}]): 3114 (ν_{C-H}), 1625, 1451, 1418 ($\nu_{C=C}$), 1253 (ν_{SO3}), 1228 (ν_{CF3}), 1163 (ν_{CF3}), 1030 (ν_{SO3}), 840 (ν_{P-F}), 639, 575 (ν_{P-F}), 515, 485, 448.

^{1}H-NMR (300 MHz, CD_3CN, [ppm]): δ 4.42 (s, 5H, Fc-Cp), 4.46 (s, 3H, CH_3 of triazolium), 4.49 (pseudo-t, 2H, *J* = 2.0 Hz, C3/C4 of substituted Fc-Cp), 5.25 (pseudo-t, 2H, *J* = 2.0 Hz, C2/C5 of substituted Fc-Cp), 6.07 (s, 5H, Cc-Cp), 6.22 (pseudo-t, 2H, *J* = 1.7 Hz, C3/C4 of substituted Cc-Cp), 6.72 (pseudo-t, 2H, J = 2.0 Hz, C2/C5 of substituted Cc-Cp).

^{13}C-NMR (75 MHz, CD_3CN, [ppm]): δ 39.4 (CH_3 of triazolium), 64.7 (C3/C4 of substituted Fc-Cp), 68.8 (C2/C5 of substituted Fc-Cp), 71.9 (Fc-Cp), 72.3 (quart. carbon of substituted Fc-Cp), 85.0 (C3/C4 of substituted Cc-Cp), 86.1 (C2/C5 of substituted Cc-Cp), 87.9 (Cc-Cp), 88.2 (quart. carbon of substituted Fc-Cp), 104.2 (quart. carbon of substituted Cc-Cp), 131.0 (quart. carbon of triazolium), 166.4 (carbene carbon of triazolium).

MS (ESI pos, [m/z]): 597.91 (M^+ - $CF_3SO_3^-$).

Melting point [°C]: 150.

Spectra

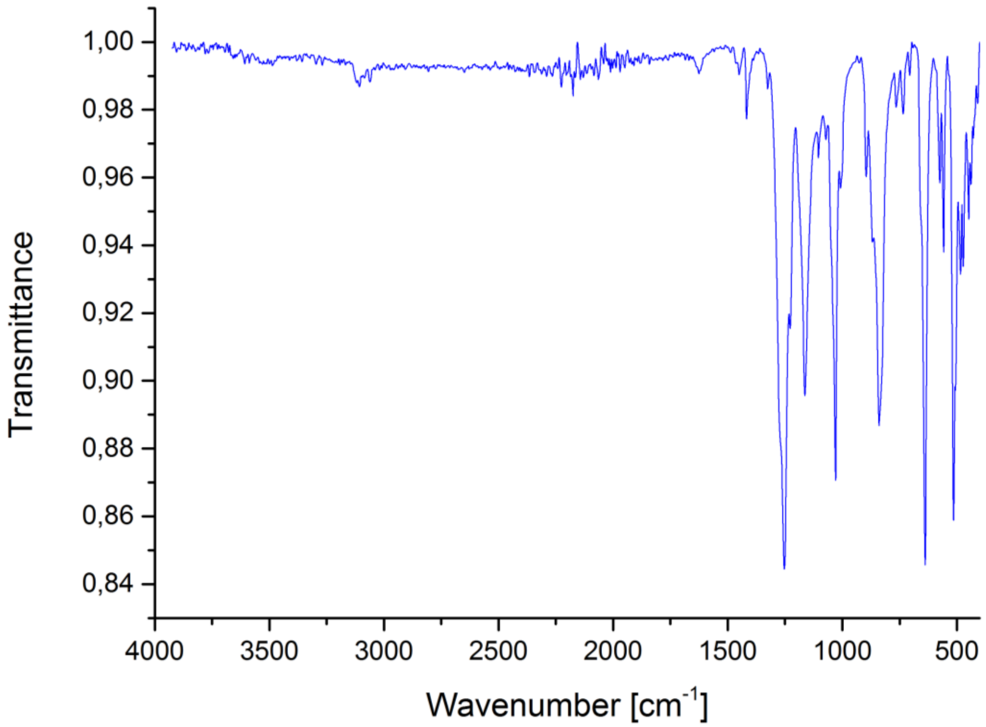

Figure S43. IR-spectrum (ATR, [cm^{-1}]) of “chlorido (4-cobaltoceniumyl-1-ferrocenyl-3-methyltriazolylidene) silver(I) triflate“.

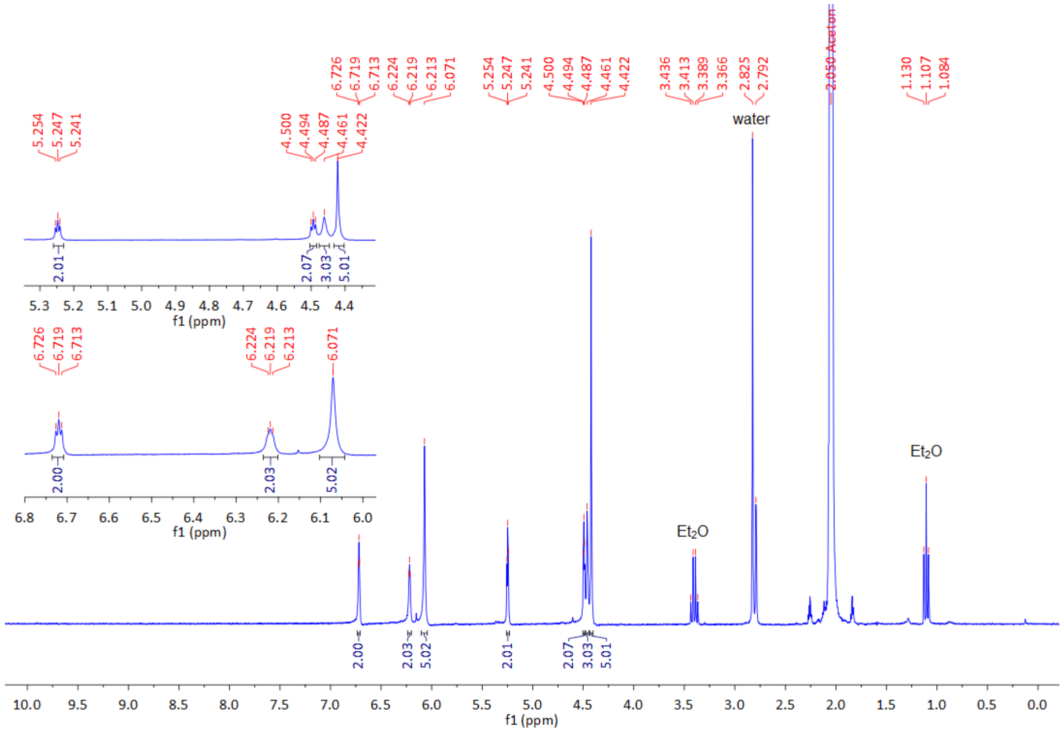

Figure S44. ^{1}H-NMR (300 MHz, CD_3CN, [ppm]) of "chlorido (4-cobaltoceniumyl-1-ferrocenyl-3-methyltriazolylidene) silver(I) triflate".

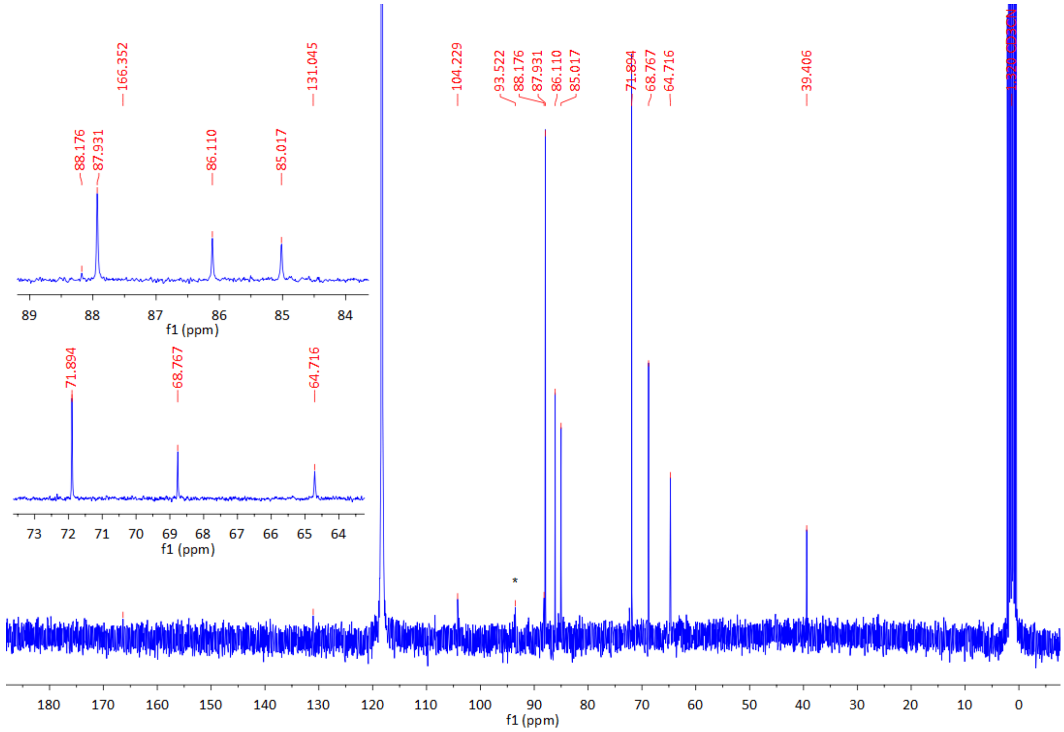

Figure S45. ^{13}C-NMR (75 MHz, CD3CN, [ppm]) of "chlorido (4-cobaltoceniumyl-1-ferrocenyl-3-methyltriazolylidene) silver(I) triflate".

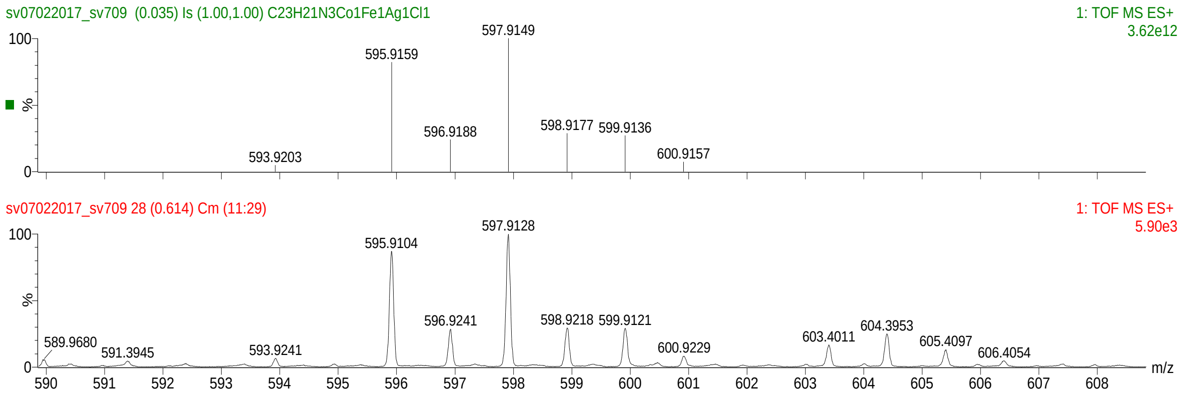

Figure S46. MS (ESI pos, [m/z]; *top*: simulated, *bottom*: experimental) of "chlorido (4-cobaltoceniumyl-1-ferrocenyl-3-methyltriazolylidene) silver(I) triflate".

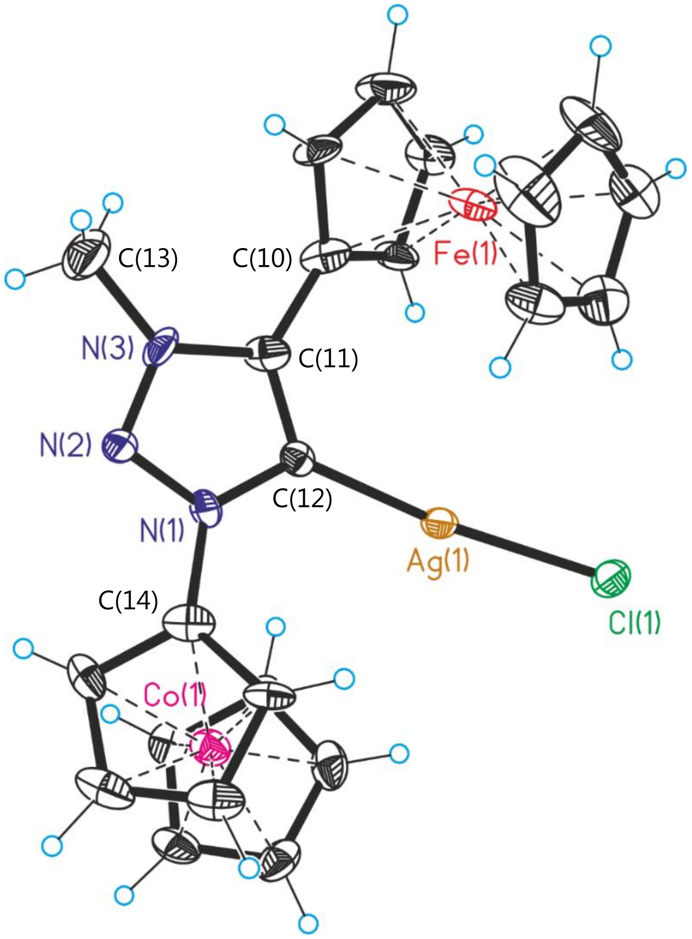

Figure S47. X-ray single crystal structure analysis of "chlorido (4-cobaltoceniumyl-1-ferrocenyl-3-methyltriazolylidene) silver(I) triflate" (counterions omitted for clarity).

X-Ray single crystal structure analysis data

Table S37. Crystal data and structure refinement for **11**.

Empirical formula	C24 H21 Ag Cl Co F3 Fe N3 O3 S x 0.33 C3H6O	
Formula weight	765.76	
Temperature	211(2) K	
Wavelength	0.71073 Å	
Crystal system	Triclinic	
Space group	P-1 (no. 2)	
Unit cell dimensions	a = 10.1622(12) Å	*α* = 78.913(4)°.
	b = 15.916(2) Å	*ß* = 89.366(4)°.
	c = 18.048(2) Å	*γ* = 80.065(4)°.
Volume	2820.9(6) $Å^3$	
Z	4	
Density (calculated)	1.803 Mg/m^3	
Absorption coefficient	1.998 mm^{-1}	
F(000)	1522	
Crystal size	0.180 x 0.040 x 0.040 mm^3	
Theta range for data collection	2.227 to 24.000°.	
Index ranges	-11<h<11, -17<k<18, 0<l<20	
Reflections collected	8543	
Independent reflections	8543 [R(int) = ?]	
Completeness to theta = 24.000°	97.5 %	
Absorption correction	Semi-empirical from equivalents	
Max. and min. transmission	0.901 and 0.663	
Refinement method	Full-matrix least-squares on F^2	
Data / restraints / parameters	8543 / 8 / 714	
Goodness-of-fit on F^2	1.108	
Final R indices [I>2sigma(I)]	R1 = 0.0681, wR2 = 0.1313	
R indices (all data)	R1 = 0.0988, wR2 = 0.1431	
Extinction coefficient	n/a	
Largest diff. peak and hole	1.152 and -0.776 e.$Å^{-3}$	

Table S38. Atomic coordinates (x 10^4) and equivalent isotropic displacement parameters ($Å^2$x 10^3) for **11**. U(eq) is defined as one third of the trace of the orthogonalized U^{ij} tensor.

	x	y	z	U(eq)
Ag(1)	1065(1)	5880(1)	5067(1)	37(1)
Ag(2)	6042(1)	5950(1)	9614(1)	41(1)
Co(1)	4145(2)	4922(1)	6583(1)	40(1)
Co(2)	9056(2)	5054(1)	8414(1)	38(1)
Fe(1)	-1821(2)	8045(1)	4588(1)	41(1)
Fe(2)	3063(2)	8037(1)	9469(1)	34(1)
Cl(1)	972(3)	5208(2)	4025(2)	44(1)
Cl(2)	6098(3)	5172(2)	10862(2)	44(1)
N(1)	2123(9)	6707(6)	6322(6)	35(2)
N(2)	1881(10)	7281(7)	6772(6)	44(3)
N(3)	646(10)	7675(7)	6559(6)	48(3)
N(4)	6965(10)	6813(6)	8046(6)	36(2)
N(5)	6677(10)	7383(7)	7409(6)	40(3)
N(6)	5446(10)	7742(6)	7518(6)	38(3)
C(1)	-1047(16)	7495(10)	3724(9)	61(4)
C(2)	-2313(16)	8024(11)	3516(8)	64(4)
C(3)	-2220(18)	8857(11)	3592(10)	80(6)
C(4)	-925(18)	8879(12)	3847(11)	88(6)
C(5)	-193(13)	8025(9)	3939(9)	54(4)
C(6)	-2092(13)	7107(8)	5475(8)	49(4)
C(7)	-3307(12)	7625(8)	5235(8)	50(4)
C(8)	-3235(14)	8483(10)	5277(10)	73(5)
C(9)	-1933(12)	8520(8)	5565(9)	51(4)
C(10)	-1233(12)	7647(8)	5686(8)	44(4)
C(11)	153(12)	7330(8)	5999(8)	42(3)
C(12)	1118(11)	6678(8)	5838(6)	33(3)
C(13)	-24(17)	8318(14)	6974(12)	109(8)
C(14)	3465(11)	6222(7)	6384(8)	38(3)
C(15)	4218(12)	5998(8)	5767(8)	45(3)
C(16)	5508(13)	5614(9)	6055(9)	50(4)
C(17)	5540(13)	5610(8)	6834(10)	58(5)
C(18)	4255(14)	5982(7)	7072(8)	45(4)
C(19)	2583(13)	4253(8)	6700(8)	43(3)
C(20)	3401(15)	4022(9)	6125(9)	54(4)
C(21)	4697(17)	3669(9)	6421(8)	56(4)
C(22)	4695(14)	3692(8)	7193(9)	51(4)
C(23)	3389(13)	4045(8)	7355(7)	41(3)
C(101)	3880(13)	7433(9)	10469(8)	48(3)
C(102)	2611(15)	7907(10)	10574(9)	60(4)
C(103)	2606(13)	8780(9)	10259(8)	50(4)
C(104)	3909(15)	8825(8)	9992(8)	50(4)
C(105)	4685(14)	8006(8)	10126(7)	43(3)
C(106)	2781(12)	7137(8)	8846(8)	45(4)

C(107)	1542(12)	7677(9)	8958(8)	45(3)
C(108)	1643(13)	8564(9)	8664(8)	52(4)
C(109)	2917(13)	8574(9)	8357(8)	46(3)
C(110)	3622(12)	7708(8)	8443(7)	36(3)
C(111)	5004(12)	7420(8)	8201(7)	34(3)
C(112)	5993(12)	6788(7)	8581(7)	33(3)
C(113)	4719(15)	8337(10)	6902(9)	68(5)
C(114)	8317(11)	6340(8)	8124(7)	38(3)
C(115)	9127(12)	6193(8)	8781(8)	47(4)
C(116)	10430(12)	5788(9)	8581(10)	56(4)
C(117)	10369(13)	5730(8)	7828(9)	48(4)
C(118)	9075(13)	6049(8)	7524(8)	41(3)
C(119)	7544(14)	4344(8)	8591(9)	49(4)
C(120)	8362(18)	4194(9)	9242(9)	63(5)
C(121)	9714(15)	3852(8)	9049(9)	55(4)
C(122)	9649(14)	3800(8)	8285(7)	46(4)
C(123)	8363(14)	4087(8)	8016(8)	48(4)
C(27)	3280(30)	9137(14)	3978(14)	106(8)
F(1)	3000(30)	9941(11)	3650(11)	228(11)
F(2)	4520(14)	8851(12)	3902(10)	175(7)
F(3)	2687(15)	8655(14)	3603(14)	238(12)
S(1)	2777(4)	8922(3)	4890(3)	75(1)
O(1)	3230(20)	8033(7)	5155(12)	178(9)
O(2)	1447(11)	9240(14)	4843(12)	191(11)
O(3)	3484(15)	9450(8)	5262(8)	107(5)
O(4)	8181(18)	7937(9)	9552(16)	196(10)
O(5)	9119(12)	9227(11)	9524(10)	135(6)
O(6)	6919(10)	9191(7)	9850(7)	81(4)
S(2)	7947(6)	8881(4)	9347(6)	102(3)
C(28)	7280(60)	9450(40)	8510(20)	200(20)
F(5)	6299(19)	9019(16)	8409(18)	217(14)
F(4)	7090(30)	10190(20)	8295(18)	230(14)
F(6)	8240(40)	9020(30)	8100(20)	280(17)
S(2A)	8230(20)	8690(15)	10091(12)	88(6)
C(28A)	8320(40)	8580(30)	11026(14)	110(30)
F(4A)	7960(40)	9350(20)	11240(30)	122(17)
F(5A)	9510(30)	8280(30)	11380(30)	106(15)
F(6A)	7510(40)	8080(20)	11420(30)	89(14)
O(7)	7230(40)	9430(20)	11890(20)	188(13)
C(29)	7980(30)	8850(20)	11685(18)	91(9)
C(30)	9300(50)	8780(30)	11560(30)	158(17)
C(31)	6910(60)	8250(30)	11530(30)	155(19)

Table S39. Bond lengths [Å] and angles [°] for **11**.

Ag(1)-C(12)	2.064(12)	N(2)-N(3)	1.328(14)
Ag(1)-Cl(1)	2.345(3)	N(3)-C(11)	1.375(16)
Ag(2)-C(112)	2.068(12)	N(3)-C(13)	1.454(17)
Ag(2)-Cl(2)	2.349(3)	N(4)-N(5)	1.320(14)
Co(1)-C(23)	2.020(12)	N(4)-C(112)	1.375(15)
Co(1)-C(14)	2.028(11)	N(4)-C(114)	1.442(15)
Co(1)-C(16)	2.033(13)	N(5)-N(6)	1.312(13)
Co(1)-C(20)	2.036(13)	N(6)-C(111)	1.346(15)
Co(1)-C(17)	2.039(13)	N(6)-C(113)	1.432(17)
Co(1)-C(22)	2.040(13)	C(1)-C(5)	1.42(2)
Co(1)-C(15)	2.045(13)	C(1)-C(2)	1.42(2)
Co(1)-C(19)	2.046(13)	C(1)-H(1)	0.9400
Co(1)-C(21)	2.051(13)	C(2)-C(3)	1.38(2)
Co(1)-C(18)	2.070(12)	C(2)-H(2)	0.9400
Co(2)-C(117)	2.020(13)	C(3)-C(4)	1.41(2)
Co(2)-C(114)	2.023(12)	C(3)-H(3)	0.9400
Co(2)-C(116)	2.028(12)	C(4)-C(5)	1.41(2)
Co(2)-C(118)	2.030(13)	C(4)-H(4)	0.9400
Co(2)-C(120)	2.031(14)	C(5)-H(5)	0.9400
Co(2)-C(121)	2.038(13)	C(6)-C(7)	1.383(17)
Co(2)-C(122)	2.039(13)	C(6)-C(10)	1.428(17)
Co(2)-C(119)	2.047(13)	C(6)-H(6)	0.9400
Co(2)-C(123)	2.048(14)	C(7)-C(8)	1.397(19)
Co(2)-C(115)	2.059(13)	C(7)-H(7)	0.9400
Fe(1)-C(3)	2.002(15)	C(8)-C(9)	1.442(19)
Fe(1)-C(8)	2.011(15)	C(8)-H(8)	0.9400
Fe(1)-C(2)	2.011(16)	C(9)-C(10)	1.425(17)
Fe(1)-C(5)	2.016(14)	C(9)-H(9)	0.9400
Fe(1)-C(1)	2.020(16)	C(10)-C(11)	1.490(18)
Fe(1)-C(6)	2.020(14)	C(11)-C(12)	1.372(17)
Fe(1)-C(4)	2.021(16)	C(13)-H(13A)	0.9700
Fe(1)-C(10)	2.024(15)	C(13)-H(13B)	0.9700
Fe(1)-C(7)	2.025(13)	C(13)-H(13C)	0.9700
Fe(1)-C(9)	2.042(13)	C(14)-C(15)	1.412(17)
Fe(2)-C(101)	1.992(15)	C(14)-C(18)	1.437(18)
Fe(2)-C(104)	2.011(13)	C(15)-C(16)	1.407(18)
Fe(2)-C(108)	2.013(13)	C(15)-H(15)	0.9400
Fe(2)-C(109)	2.020(14)	C(16)-C(17)	1.41(2)
Fe(2)-C(102)	2.021(14)	C(16)-H(16)	0.9400
Fe(2)-C(103)	2.022(13)	C(17)-C(18)	1.43(2)
Fe(2)-C(107)	2.028(13)	C(17)-H(17)	0.9400
Fe(2)-C(105)	2.029(13)	C(18)-H(18)	0.9400
Fe(2)-C(106)	2.042(11)	C(19)-C(20)	1.388(19)
Fe(2)-C(110)	2.067(12)	C(19)-C(23)	1.398(18)
N(1)-N(2)	1.324(13)	C(19)-H(19)	0.9400
N(1)-C(12)	1.364(14)	C(20)-C(21)	1.41(2)
N(1)-C(14)	1.441(15)	C(20)-H(20)	0.9400

C(21)-C(22)	1.40(2)	C(117)-H(117)	0.9400
C(21)-H(21)	0.9400	C(118)-H(118)	0.9400
C(22)-C(23)	1.399(18)	C(119)-C(123)	1.402(19)
C(22)-H(22)	0.9400	C(119)-C(120)	1.41(2)
C(23)-H(23)	0.9400	C(119)-H(119)	0.9400
C(101)-C(105)	1.384(18)	C(120)-C(121)	1.45(2)
C(101)-C(102)	1.409(19)	C(120)-H(120)	0.9400
C(101)-H(101)	0.9400	C(121)-C(122)	1.40(2)
C(102)-C(103)	1.396(19)	C(121)-H(121)	0.9400
C(102)-H(102)	0.9400	C(122)-C(123)	1.370(19)
C(103)-C(104)	1.412(19)	C(122)-H(122)	0.9400
C(103)-H(103)	0.9400	C(123)-H(123)	0.9400
C(104)-C(105)	1.381(18)	C(27)-F(2)	1.28(2)
C(104)-H(104)	0.9400	C(27)-F(1)	1.29(2)
C(105)-H(105)	0.9400	C(27)-F(3)	1.34(2)
C(106)-C(107)	1.433(17)	C(27)-S(1)	1.71(3)
C(106)-C(110)	1.444(18)	S(1)-O(2)	1.357(12)
C(106)-H(106)	0.9400	S(1)-O(1)	1.400(12)
C(107)-C(108)	1.431(18)	S(1)-O(3)	1.453(12)
C(107)-H(107)	0.9400	O(4)-S(2)	1.455(16)
C(108)-C(109)	1.404(19)	O(4)-S(2A)	1.69(3)
C(108)-H(108)	0.9400	O(5)-S(2)	1.458(15)
C(109)-C(110)	1.421(16)	O(5)-S(2A)	1.58(3)
C(109)-H(109)	0.9400	O(6)-S(2A)	1.46(2)
C(110)-C(111)	1.487(16)	O(6)-S(2)	1.461(12)
C(111)-C(112)	1.376(16)	S(2)-C(28)	1.68(2)
C(113)-H(11A)	0.9700	C(28)-F(4)	1.15(5)
C(113)-H(11B)	0.9700	C(28)-F(5)	1.34(6)
C(113)-H(11C)	0.9700	C(28)-F(6)	1.39(6)
C(114)-C(115)	1.409(18)	S(2A)-C(28A)	1.666(19)
C(114)-C(118)	1.425(16)	C(28A)-F(5A)	1.33(2)
C(115)-C(116)	1.444(17)	C(28A)-F(6A)	1.35(2)
C(115)-H(115)	0.9400	C(28A)-F(4A)	1.35(2)
C(116)-C(117)	1.38(2)	O(7)-C(29)	1.21(4)
C(116)-H(116)	0.9400	C(29)-C(30)	1.35(5)
C(117)-C(118)	1.403(18)	C(29)-C(31)	1.61(6)

C(12)-Ag(1)-Cl(1)	169.6(3)	C(16)-Co(1)-C(17)	40.4(6)
C(112)-Ag(2)-Cl(2)	171.9(3)	C(20)-Co(1)-C(17)	157.1(6)
C(23)-Co(1)-C(14)	124.5(5)	C(23)-Co(1)-C(22)	40.3(5)
C(23)-Co(1)-C(16)	158.8(6)	C(14)-Co(1)-C(22)	157.9(6)
C(14)-Co(1)-C(16)	67.5(5)	C(16)-Co(1)-C(22)	121.9(6)
C(23)-Co(1)-C(20)	66.6(6)	C(20)-Co(1)-C(22)	67.6(6)
C(14)-Co(1)-C(20)	126.6(6)	C(17)-Co(1)-C(22)	106.0(6)
C(16)-Co(1)-C(20)	122.8(7)	C(23)-Co(1)-C(15)	159.9(5)
C(23)-Co(1)-C(17)	123.6(6)	C(14)-Co(1)-C(15)	40.6(5)
C(14)-Co(1)-C(17)	67.3(5)	C(16)-Co(1)-C(15)	40.4(5)

C(20)-Co(1)-C(15)	109.5(6)
C(17)-Co(1)-C(15)	68.0(6)
C(22)-Co(1)-C(15)	158.6(5)
C(23)-Co(1)-C(19)	40.2(5)
C(14)-Co(1)-C(19)	110.6(5)
C(16)-Co(1)-C(19)	158.5(6)
C(20)-Co(1)-C(19)	39.8(5)
C(17)-Co(1)-C(19)	160.5(6)
C(22)-Co(1)-C(19)	68.4(6)
C(15)-Co(1)-C(19)	124.0(5)
C(23)-Co(1)-C(21)	66.8(6)
C(14)-Co(1)-C(21)	161.7(6)
C(16)-Co(1)-C(21)	107.0(6)
C(20)-Co(1)-C(21)	40.3(6)
C(17)-Co(1)-C(21)	120.8(6)
C(22)-Co(1)-C(21)	40.0(6)
C(15)-Co(1)-C(21)	124.0(6)
C(19)-Co(1)-C(21)	67.6(6)
C(23)-Co(1)-C(18)	107.4(6)
C(14)-Co(1)-C(18)	41.0(5)
C(16)-Co(1)-C(18)	69.3(6)
C(20)-Co(1)-C(18)	161.6(6)
C(17)-Co(1)-C(18)	40.9(6)
C(22)-Co(1)-C(18)	120.0(6)
C(15)-Co(1)-C(18)	69.7(5)
C(19)-Co(1)-C(18)	124.4(5)
C(21)-Co(1)-C(18)	155.5(6)
C(117)-Co(2)-C(114)	67.5(5)
C(117)-Co(2)-C(116)	39.9(6)
C(114)-Co(2)-C(116)	68.3(5)
C(117)-Co(2)-C(118)	40.5(5)
C(114)-Co(2)-C(118)	41.2(5)
C(116)-Co(2)-C(118)	68.8(6)
C(117)-Co(2)-C(120)	158.6(7)
C(114)-Co(2)-C(120)	125.8(6)
C(116)-Co(2)-C(120)	124.4(7)
C(118)-Co(2)-C(120)	160.5(6)
C(117)-Co(2)-C(121)	120.0(6)
C(114)-Co(2)-C(121)	161.2(6)
C(116)-Co(2)-C(121)	105.7(6)
C(118)-Co(2)-C(121)	155.2(5)
C(120)-Co(2)-C(121)	41.8(6)
C(117)-Co(2)-C(122)	105.4(5)
C(114)-Co(2)-C(122)	158.4(5)
C(116)-Co(2)-C(122)	120.3(5)
C(118)-Co(2)-C(122)	120.2(5)
C(120)-Co(2)-C(122)	67.9(6)
C(121)-Co(2)-C(122)	40.1(5)
C(117)-Co(2)-C(119)	157.8(6)
C(114)-Co(2)-C(119)	110.8(5)
C(116)-Co(2)-C(119)	161.9(6)
C(118)-Co(2)-C(119)	123.3(6)
C(120)-Co(2)-C(119)	40.3(6)
C(121)-Co(2)-C(119)	69.0(6)
C(122)-Co(2)-C(119)	67.5(5)
C(117)-Co(2)-C(123)	121.5(6)
C(114)-Co(2)-C(123)	125.6(5)
C(116)-Co(2)-C(123)	155.4(6)
C(118)-Co(2)-C(123)	107.2(6)
C(120)-Co(2)-C(123)	67.0(6)
C(121)-Co(2)-C(123)	67.2(6)
C(122)-Co(2)-C(123)	39.2(5)
C(119)-Co(2)-C(123)	40.0(5)
C(117)-Co(2)-C(115)	68.0(5)
C(114)-Co(2)-C(115)	40.4(5)
C(116)-Co(2)-C(115)	41.4(5)
C(118)-Co(2)-C(115)	69.3(6)
C(120)-Co(2)-C(115)	110.1(6)
C(121)-Co(2)-C(115)	123.5(6)
C(122)-Co(2)-C(115)	158.1(5)
C(119)-Co(2)-C(115)	126.2(5)
C(123)-Co(2)-C(115)	162.0(5)
C(3)-Fe(1)-C(8)	104.8(8)
C(3)-Fe(1)-C(2)	40.2(7)
C(8)-Fe(1)-C(2)	119.7(7)
C(3)-Fe(1)-C(5)	68.9(7)
C(8)-Fe(1)-C(5)	158.5(7)
C(2)-Fe(1)-C(5)	69.5(6)
C(3)-Fe(1)-C(1)	68.3(7)
C(8)-Fe(1)-C(1)	157.4(7)
C(2)-Fe(1)-C(1)	41.3(6)
C(5)-Fe(1)-C(1)	41.1(6)
C(3)-Fe(1)-C(6)	157.6(6)
C(8)-Fe(1)-C(6)	68.2(6)
C(2)-Fe(1)-C(6)	123.3(6)
C(5)-Fe(1)-C(6)	125.2(6)
C(1)-Fe(1)-C(6)	109.6(6)
C(3)-Fe(1)-C(4)	40.9(7)
C(8)-Fe(1)-C(4)	121.0(8)
C(2)-Fe(1)-C(4)	68.8(7)
C(5)-Fe(1)-C(4)	41.0(7)
C(1)-Fe(1)-C(4)	68.7(7)
C(6)-Fe(1)-C(4)	160.9(6)
C(3)-Fe(1)-C(10)	157.9(6)
C(8)-Fe(1)-C(10)	68.7(6)
C(2)-Fe(1)-C(10)	161.4(6)
C(5)-Fe(1)-C(10)	109.1(6)
C(1)-Fe(1)-C(10)	125.5(6)
C(6)-Fe(1)-C(10)	41.4(5)
C(4)-Fe(1)-C(10)	123.0(6)

C(3)-Fe(1)-C(7)	121.1(7)
C(8)-Fe(1)-C(7)	40.5(6)
C(2)-Fe(1)-C(7)	106.4(6)
C(5)-Fe(1)-C(7)	160.3(6)
C(1)-Fe(1)-C(7)	123.3(6)
C(6)-Fe(1)-C(7)	40.0(5)
C(4)-Fe(1)-C(7)	157.1(7)
C(10)-Fe(1)-C(7)	68.4(5)
C(3)-Fe(1)-C(9)	120.2(7)
C(8)-Fe(1)-C(9)	41.7(6)
C(2)-Fe(1)-C(9)	155.7(6)
C(5)-Fe(1)-C(9)	122.4(6)
C(1)-Fe(1)-C(9)	160.5(6)
C(6)-Fe(1)-C(9)	69.6(5)
C(4)-Fe(1)-C(9)	105.4(7)
C(10)-Fe(1)-C(9)	41.0(5)
C(7)-Fe(1)-C(9)	69.4(5)
C(101)-Fe(2)-C(104)	67.5(5)
C(101)-Fe(2)-C(108)	159.2(6)
C(104)-Fe(2)-C(108)	119.2(6)
C(101)-Fe(2)-C(109)	159.8(5)
C(104)-Fe(2)-C(109)	107.3(6)
C(108)-Fe(2)-C(109)	40.7(5)
C(101)-Fe(2)-C(102)	41.1(6)
C(104)-Fe(2)-C(102)	67.5(6)
C(108)-Fe(2)-C(102)	120.5(6)
C(109)-Fe(2)-C(102)	157.0(6)
C(101)-Fe(2)-C(103)	68.9(6)
C(104)-Fe(2)-C(103)	41.0(5)
C(108)-Fe(2)-C(103)	102.6(6)
C(109)-Fe(2)-C(103)	120.8(5)
C(102)-Fe(2)-C(103)	40.4(5)
C(101)-Fe(2)-C(107)	124.5(6)
C(104)-Fe(2)-C(107)	154.8(5)
C(108)-Fe(2)-C(107)	41.5(5)
C(109)-Fe(2)-C(107)	68.8(6)
C(102)-Fe(2)-C(107)	105.9(6)
C(103)-Fe(2)-C(107)	118.1(5)
C(101)-Fe(2)-C(105)	40.3(5)
C(104)-Fe(2)-C(105)	40.0(5)
C(108)-Fe(2)-C(105)	156.0(6)
C(109)-Fe(2)-C(105)	123.3(6)
C(102)-Fe(2)-C(105)	68.2(6)
C(103)-Fe(2)-C(105)	68.8(5)
C(107)-Fe(2)-C(105)	162.4(5)
C(101)-Fe(2)-C(106)	109.4(6)
C(104)-Fe(2)-C(106)	162.8(5)
C(108)-Fe(2)-C(106)	70.1(6)
C(109)-Fe(2)-C(106)	69.5(6)
C(102)-Fe(2)-C(106)	122.1(6)
C(103)-Fe(2)-C(106)	155.4(5)
C(107)-Fe(2)-C(106)	41.2(5)
C(105)-Fe(2)-C(106)	126.8(5)
C(101)-Fe(2)-C(110)	125.3(5)
C(104)-Fe(2)-C(110)	125.7(5)
C(108)-Fe(2)-C(110)	68.7(5)
C(109)-Fe(2)-C(110)	40.7(5)
C(102)-Fe(2)-C(110)	160.1(5)
C(103)-Fe(2)-C(110)	159.4(5)
C(107)-Fe(2)-C(110)	68.5(5)
C(105)-Fe(2)-C(110)	111.2(5)
C(106)-Fe(2)-C(110)	41.1(5)
N(2)-N(1)-C(12)	117.2(10)
N(2)-N(1)-C(14)	114.4(9)
C(12)-N(1)-C(14)	128.2(10)
N(1)-N(2)-N(3)	102.0(9)
N(2)-N(3)-C(11)	111.6(9)
N(2)-N(3)-C(13)	118.5(11)
C(11)-N(3)-C(13)	129.6(12)
N(5)-N(4)-C(112)	117.0(10)
N(5)-N(4)-C(114)	115.6(10)
C(112)-N(4)-C(114)	127.2(10)
N(6)-N(5)-N(4)	102.0(9)
N(5)-N(6)-C(111)	112.5(10)
N(5)-N(6)-C(113)	118.6(11)
C(111)-N(6)-C(113)	128.6(11)
C(5)-C(1)-C(2)	108.0(13)
C(5)-C(1)-Fe(1)	69.3(9)
C(2)-C(1)-Fe(1)	69.0(9)
C(5)-C(1)-H(1)	126.0
C(2)-C(1)-H(1)	126.0
Fe(1)-C(1)-H(1)	127.2
C(3)-C(2)-C(1)	107.5(14)
C(3)-C(2)-Fe(1)	69.6(10)
C(1)-C(2)-Fe(1)	69.7(9)
C(3)-C(2)-H(2)	126.3
C(1)-C(2)-H(2)	126.3
Fe(1)-C(2)-H(2)	126.1
C(2)-C(3)-C(4)	109.7(15)
C(2)-C(3)-Fe(1)	70.3(9)
C(4)-C(3)-Fe(1)	70.3(9)
C(2)-C(3)-H(3)	125.1
C(4)-C(3)-H(3)	125.1
Fe(1)-C(3)-H(3)	125.9
C(3)-C(4)-C(5)	107.4(14)
C(3)-C(4)-Fe(1)	68.8(9)
C(5)-C(4)-Fe(1)	69.3(8)
C(3)-C(4)-H(4)	126.3
C(5)-C(4)-H(4)	126.3
Fe(1)-C(4)-H(4)	127.2

C(4)-C(5)-C(1)	107.5(13)
C(4)-C(5)-Fe(1)	69.7(9)
C(1)-C(5)-Fe(1)	69.6(8)
C(4)-C(5)-H(5)	126.3
C(1)-C(5)-H(5)	126.3
Fe(1)-C(5)-H(5)	126.0
C(7)-C(6)-C(10)	108.1(11)
C(7)-C(6)-Fe(1)	70.2(8)
C(10)-C(6)-Fe(1)	69.5(8)
C(7)-C(6)-H(6)	126.0
C(10)-C(6)-H(6)	126.0
Fe(1)-C(6)-H(6)	126.0
C(6)-C(7)-C(8)	108.8(12)
C(6)-C(7)-Fe(1)	69.8(8)
C(8)-C(7)-Fe(1)	69.2(9)
C(6)-C(7)-H(7)	125.6
C(8)-C(7)-H(7)	125.6
Fe(1)-C(7)-H(7)	126.9
C(7)-C(8)-C(9)	109.3(12)
C(7)-C(8)-Fe(1)	70.3(8)
C(9)-C(8)-Fe(1)	70.3(8)
C(7)-C(8)-H(8)	125.3
C(9)-C(8)-H(8)	125.3
Fe(1)-C(8)-H(8)	125.6
C(10)-C(9)-C(8)	105.1(11)
C(10)-C(9)-Fe(1)	68.8(8)
C(8)-C(9)-Fe(1)	68.0(8)
C(10)-C(9)-H(9)	127.4
C(8)-C(9)-H(9)	127.4
Fe(1)-C(9)-H(9)	127.2
C(9)-C(10)-C(6)	108.8(11)
C(9)-C(10)-C(11)	126.4(11)
C(6)-C(10)-C(11)	124.8(11)
C(9)-C(10)-Fe(1)	70.2(8)
C(6)-C(10)-Fe(1)	69.2(8)
C(11)-C(10)-Fe(1)	127.8(9)
C(12)-C(11)-N(3)	108.3(11)
C(12)-C(11)-C(10)	130.1(12)
N(3)-C(11)-C(10)	121.5(11)
N(1)-C(12)-C(11)	100.9(11)
N(1)-C(12)-Ag(1)	129.7(8)
C(11)-C(12)-Ag(1)	129.5(9)
N(3)-C(13)-H(13A)	109.5
N(3)-C(13)-H(13B)	109.5
H(13A)-C(13)-H(13B)	109.5
N(3)-C(13)-H(13C)	109.5
H(13A)-C(13)-H(13C)	109.5
H(13B)-C(13)-H(13C)	109.5
C(15)-C(14)-C(18)	111.3(11)
C(15)-C(14)-N(1)	124.4(12)
C(18)-C(14)-N(1)	124.0(12)
C(15)-C(14)-Co(1)	70.3(7)
C(18)-C(14)-Co(1)	71.0(7)
N(1)-C(14)-Co(1)	130.8(7)
C(16)-C(15)-C(14)	106.5(13)
C(16)-C(15)-Co(1)	69.4(7)
C(14)-C(15)-Co(1)	69.1(7)
C(16)-C(15)-H(15)	126.8
C(14)-C(15)-H(15)	126.8
Co(1)-C(15)-H(15)	126.3
C(17)-C(16)-C(15)	108.5(13)
C(17)-C(16)-Co(1)	70.0(8)
C(15)-C(16)-Co(1)	70.3(7)
C(17)-C(16)-H(16)	125.7
C(15)-C(16)-H(16)	125.7
Co(1)-C(16)-H(16)	125.5
C(16)-C(17)-C(18)	110.4(12)
C(16)-C(17)-Co(1)	69.6(8)
C(18)-C(17)-Co(1)	70.7(7)
C(16)-C(17)-H(17)	124.8
C(18)-C(17)-H(17)	124.8
Co(1)-C(17)-H(17)	126.5
C(17)-C(18)-C(14)	103.3(13)
C(17)-C(18)-Co(1)	68.4(8)
C(14)-C(18)-Co(1)	67.9(7)
C(17)-C(18)-H(18)	128.3
C(14)-C(18)-H(18)	128.3
Co(1)-C(18)-H(18)	126.9
C(20)-C(19)-C(23)	106.1(12)
C(20)-C(19)-Co(1)	69.7(8)
C(23)-C(19)-Co(1)	68.9(7)
C(20)-C(19)-H(19)	126.9
C(23)-C(19)-H(19)	126.9
Co(1)-C(19)-H(19)	126.0
C(19)-C(20)-C(21)	109.3(13)
C(19)-C(20)-Co(1)	70.5(8)
C(21)-C(20)-Co(1)	70.4(8)
C(19)-C(20)-H(20)	125.4
C(21)-C(20)-H(20)	125.4
Co(1)-C(20)-H(20)	125.3
C(22)-C(21)-C(20)	107.8(13)
C(22)-C(21)-Co(1)	69.6(8)
C(20)-C(21)-Co(1)	69.3(7)
C(22)-C(21)-H(21)	126.1
C(20)-C(21)-H(21)	126.1
Co(1)-C(21)-H(21)	126.6
C(23)-C(22)-C(21)	106.5(13)
C(23)-C(22)-Co(1)	69.1(7)
C(21)-C(22)-Co(1)	70.4(8)
C(23)-C(22)-H(22)	126.8

C(21)-C(22)-H(22)	126.8	C(109)-C(108)-C(107)	107.5(11)
Co(1)-C(22)-H(22)	125.3	C(109)-C(108)-Fe(2)	69.9(8)
C(19)-C(23)-C(22)	110.3(12)	C(107)-C(108)-Fe(2)	69.8(7)
C(19)-C(23)-Co(1)	70.9(7)	C(109)-C(108)-H(108)	126.2
C(22)-C(23)-Co(1)	70.6(8)	C(107)-C(108)-H(108)	126.2
C(19)-C(23)-H(23)	124.9	Fe(2)-C(108)-H(108)	125.6
C(22)-C(23)-H(23)	124.9	C(108)-C(109)-C(110)	109.3(13)
Co(1)-C(23)-H(23)	125.2	C(108)-C(109)-Fe(2)	69.4(8)
C(105)-C(101)-C(102)	108.7(12)	C(110)-C(109)-Fe(2)	71.4(8)
C(105)-C(101)-Fe(2)	71.3(8)	C(108)-C(109)-H(109)	125.3
C(102)-C(101)-Fe(2)	70.6(9)	C(110)-C(109)-H(109)	125.3
C(105)-C(101)-H(101)	125.6	Fe(2)-C(109)-H(109)	125.4
C(102)-C(101)-H(101)	125.6	C(109)-C(110)-C(106)	107.8(11)
Fe(2)-C(101)-H(101)	124.1	C(109)-C(110)-C(111)	127.3(12)
C(103)-C(102)-C(101)	108.2(12)	C(106)-C(110)-C(111)	124.9(11)
C(103)-C(102)-Fe(2)	69.8(8)	C(109)-C(110)-Fe(2)	67.9(7)
C(101)-C(102)-Fe(2)	68.3(8)	C(106)-C(110)-Fe(2)	68.5(7)
C(103)-C(102)-H(102)	125.9	C(111)-C(110)-Fe(2)	126.9(9)
C(101)-C(102)-H(102)	125.9	N(6)-C(111)-C(112)	109.0(10)
Fe(2)-C(102)-H(102)	127.5	N(6)-C(111)-C(110)	122.2(11)
C(102)-C(103)-C(104)	105.9(11)	C(112)-C(111)-C(110)	128.7(11)
C(102)-C(103)-Fe(2)	69.8(8)	N(4)-C(112)-C(111)	99.6(10)
C(104)-C(103)-Fe(2)	69.1(7)	N(4)-C(112)-Ag(2)	128.0(8)
C(102)-C(103)-H(103)	127.1	C(111)-C(112)-Ag(2)	132.3(9)
C(104)-C(103)-H(103)	127.1	N(6)-C(113)-H(11A)	109.5
Fe(2)-C(103)-H(103)	125.7	N(6)-C(113)-H(11B)	109.5
C(105)-C(104)-C(103)	110.1(12)	H(11A)-C(113)-H(11B)	109.5
C(105)-C(104)-Fe(2)	70.7(7)	N(6)-C(113)-H(11C)	109.5
C(103)-C(104)-Fe(2)	69.9(8)	H(11A)-C(113)-H(11C)	109.5
C(105)-C(104)-H(104)	124.9	H(11B)-C(113)-H(11C)	109.5
C(103)-C(104)-H(104)	124.9	C(115)-C(114)-C(118)	110.2(11)
Fe(2)-C(104)-H(104)	126.0	C(115)-C(114)-N(4)	124.6(11)
C(104)-C(105)-C(101)	107.0(13)	C(118)-C(114)-N(4)	124.8(11)
C(104)-C(105)-Fe(2)	69.3(8)	C(115)-C(114)-Co(2)	71.2(7)
C(101)-C(105)-Fe(2)	68.4(8)	C(118)-C(114)-Co(2)	69.7(7)
C(104)-C(105)-H(105)	126.5	N(4)-C(114)-Co(2)	131.8(8)
C(101)-C(105)-H(105)	126.5	C(114)-C(115)-C(116)	105.8(12)
Fe(2)-C(105)-H(105)	127.3	C(114)-C(115)-Co(2)	68.5(7)
C(107)-C(106)-C(110)	106.5(11)	C(116)-C(115)-Co(2)	68.2(7)
C(107)-C(106)-Fe(2)	68.9(7)	C(114)-C(115)-H(115)	127.1
C(110)-C(106)-Fe(2)	70.4(6)	C(116)-C(115)-H(115)	127.1
C(107)-C(106)-H(106)	126.8	Co(2)-C(115)-H(115)	127.7
C(110)-C(106)-H(106)	126.8	C(117)-C(116)-C(115)	107.7(13)
Fe(2)-C(106)-H(106)	125.6	C(117)-C(116)-Co(2)	69.7(7)
C(108)-C(107)-C(106)	108.8(11)	C(115)-C(116)-Co(2)	70.4(7)
C(108)-C(107)-Fe(2)	68.7(7)	C(117)-C(116)-H(116)	126.1
C(106)-C(107)-Fe(2)	69.9(7)	C(115)-C(116)-H(116)	126.1
C(108)-C(107)-H(107)	125.6	Co(2)-C(116)-H(116)	125.3
C(106)-C(107)-H(107)	125.6	C(116)-C(117)-C(118)	110.9(11)
Fe(2)-C(107)-H(107)	127.3	C(116)-C(117)-Co(2)	70.4(8)

C(118)-C(117)-Co(2)	70.1(7)
C(116)-C(117)-H(117)	124.5
C(118)-C(117)-H(117)	124.5
Co(2)-C(117)-H(117)	126.6
C(117)-C(118)-C(114)	105.3(12)
C(117)-C(118)-Co(2)	69.4(8)
C(114)-C(118)-Co(2)	69.2(7)
C(117)-C(118)-H(118)	127.3
C(114)-C(118)-H(118)	127.3
Co(2)-C(118)-H(118)	125.7
C(123)-C(119)-C(120)	106.7(13)
C(123)-C(119)-Co(2)	70.0(8)
C(120)-C(119)-Co(2)	69.3(8)
C(123)-C(119)-H(119)	126.6
C(120)-C(119)-H(119)	126.6
Co(2)-C(119)-H(119)	125.7
C(119)-C(120)-C(121)	108.1(13)
C(119)-C(120)-Co(2)	70.4(8)
C(121)-C(120)-Co(2)	69.3(8)
C(119)-C(120)-H(120)	125.9
C(121)-C(120)-H(120)	125.9
Co(2)-C(120)-H(120)	125.9
C(122)-C(121)-C(120)	105.7(13)
C(122)-C(121)-Co(2)	70.0(8)
C(120)-C(121)-Co(2)	68.8(7)
C(122)-C(121)-H(121)	127.1
C(120)-C(121)-H(121)	127.1
Co(2)-C(121)-H(121)	125.6
C(123)-C(122)-C(121)	109.5(13)
C(123)-C(122)-Co(2)	70.7(8)
C(121)-C(122)-Co(2)	69.9(8)
C(123)-C(122)-H(122)	125.3
C(121)-C(122)-H(122)	125.3
Co(2)-C(122)-H(122)	125.7
C(122)-C(123)-C(119)	109.9(13)
C(122)-C(123)-Co(2)	70.1(8)
C(119)-C(123)-Co(2)	69.9(8)
C(122)-C(123)-H(123)	125.0
C(119)-C(123)-H(123)	125.0
Co(2)-C(123)-H(123)	126.5
F(2)-C(27)-F(1)	110(3)
F(2)-C(27)-F(3)	102.4(18)
F(1)-C(27)-F(3)	110(2)
F(2)-C(27)-S(1)	113.3(18)
F(1)-C(27)-S(1)	114.8(17)
F(3)-C(27)-S(1)	106(2)
O(2)-S(1)-O(1)	119.8(13)
O(2)-S(1)-O(3)	110.6(10)
O(1)-S(1)-O(3)	111.6(12)
O(2)-S(1)-C(27)	103.9(13)
O(1)-S(1)-C(27)	106.0(11)
O(3)-S(1)-C(27)	103.0(10)
O(4)-S(2)-O(5)	111.0(12)
O(4)-S(2)-O(6)	106.2(12)
O(5)-S(2)-O(6)	104.8(9)
O(4)-S(2)-C(28)	124(3)
O(5)-S(2)-C(28)	109(3)
O(6)-S(2)-C(28)	99(2)
F(4)-C(28)-F(5)	117(4)
F(4)-C(28)-F(6)	112(5)
F(5)-C(28)-F(6)	97(5)
F(4)-C(28)-S(2)	129(5)
F(5)-C(28)-S(2)	101(3)
F(6)-C(28)-S(2)	93(3)
O(6)-S(2A)-O(5)	99.2(15)
O(6)-S(2A)-C(28A)	107(2)
O(5)-S(2A)-C(28A)	124(2)
O(6)-S(2A)-O(4)	95.3(14)
O(5)-S(2A)-O(4)	94.4(15)
C(28A)-S(2A)-O(4)	130(2)
F(5A)-C(28A)-F(6A)	103.8(19)
F(5A)-C(28A)-F(4A)	101.8(19)
F(6A)-C(28A)-F(4A)	103.3(19)
F(5A)-C(28A)-S(2A)	119(3)
F(6A)-C(28A)-S(2A)	116(3)
F(4A)-C(28A)-S(2A)	112(4)
O(7)-C(29)-C(30)	128(4)
O(7)-C(29)-C(31)	100(4)
C(30)-C(29)-C(31)	132(4)

Table S40. Anisotropic displacement parameters ($Å^2 x 10^3$) for **11**. The anisotropic displacement factor exponent takes the form: $-2p^2 [h^2 a^{*2}U^{11} + ... + 2 h k a^* b^* U^{12}]$.

	U^{11}	U^{22}	U^{33}	U^{23}	U^{13}	U^{12}
Ag(1)	29(1)	35(1)	48(1)	-17(1)	-7(1)	2(1)
Ag(2)	35(1)	46(1)	39(1)	-7(1)	4(1)	3(1)
Co(1)	34(1)	37(1)	50(1)	-8(1)	-4(1)	-7(1)
Co(2)	31(1)	38(1)	47(1)	-13(1)	1(1)	-4(1)
Fe(1)	27(1)	26(1)	67(1)	-4(1)	-13(1)	-2(1)
Fe(2)	24(1)	27(1)	55(1)	-21(1)	7(1)	-7(1)
Cl(1)	37(2)	49(2)	49(2)	-20(2)	4(2)	-6(2)
Cl(2)	38(2)	51(2)	42(2)	-8(2)	-3(2)	-4(1)
N(1)	28(6)	36(6)	44(6)	-14(5)	-11(5)	-11(4)
N(2)	30(6)	56(7)	52(7)	-29(6)	-8(5)	2(5)
N(3)	42(7)	53(7)	63(8)	-45(6)	-1(6)	-4(5)
N(4)	36(6)	24(6)	48(7)	-10(5)	-5(5)	-3(4)
N(5)	35(6)	36(6)	48(7)	-8(5)	0(5)	-1(5)
N(6)	40(6)	34(6)	36(6)	-5(5)	-2(5)	4(5)
C(1)	76(11)	41(9)	65(10)	-2(8)	6(9)	-13(8)
C(2)	59(10)	75(12)	55(10)	1(8)	-9(8)	-15(9)
C(3)	83(13)	51(11)	87(13)	33(9)	-40(10)	-6(9)
C(4)	80(13)	70(13)	116(16)	12(11)	-28(11)	-56(11)
C(5)	30(7)	52(9)	74(11)	5(8)	4(7)	-11(7)
C(6)	43(8)	29(7)	72(10)	-16(7)	-21(7)	9(6)
C(7)	25(7)	47(8)	82(11)	-14(7)	-5(7)	-16(6)
C(8)	35(8)	58(10)	122(16)	-29(10)	-2(9)	18(7)
C(9)	28(7)	38(8)	96(12)	-45(8)	-8(7)	2(6)
C(10)	28(7)	32(7)	80(11)	-23(7)	11(7)	-12(6)
C(11)	25(7)	37(8)	68(10)	-18(7)	-4(6)	-12(6)
C(12)	32(7)	38(7)	28(7)	-1(5)	1(6)	-9(6)
C(13)	59(11)	140(19)	152(19)	-117(17)	-20(12)	16(11)
C(14)	26(7)	23(6)	66(9)	-1(6)	9(6)	-16(5)
C(15)	23(7)	42(8)	64(10)	-7(7)	2(6)	5(6)
C(16)	30(7)	44(8)	72(11)	0(8)	10(7)	-14(6)
C(17)	31(8)	26(8)	113(15)	4(8)	-20(8)	-11(6)
C(18)	60(9)	20(7)	56(9)	-4(6)	-30(7)	-12(6)
C(19)	46(8)	37(8)	55(9)	-26(7)	7(7)	-9(6)
C(20)	67(10)	49(9)	54(10)	-19(8)	-11(8)	-21(8)
C(21)	81(12)	33(8)	51(10)	-9(7)	13(8)	4(7)
C(22)	45(8)	27(7)	74(11)	4(7)	-16(8)	2(6)
C(23)	55(9)	35(7)	31(8)	3(6)	6(6)	-16(6)
C(101)	47(8)	46(8)	58(9)	-17(7)	8(7)	-21(7)
C(102)	64(10)	61(10)	72(11)	-38(9)	44(9)	-32(8)
C(103)	29(7)	47(9)	75(11)	-25(8)	5(7)	4(6)
C(104)	73(11)	26(7)	56(9)	-15(6)	-4(8)	-15(7)
C(105)	51(8)	44(8)	43(8)	-31(6)	-5(6)	-12(7)
C(106)	32(7)	32(7)	84(10)	-36(7)	8(7)	-15(6)

C(107)	27(7)	55(9)	62(9)	-28(7)	9(6)	-11(6)
C(108)	35(8)	50(9)	68(11)	-18(8)	-13(7)	9(7)
C(109)	48(9)	44(8)	49(9)	-23(7)	-3(7)	-1(7)
C(110)	34(7)	35(7)	44(8)	-20(6)	-1(6)	-5(6)
C(111)	37(7)	34(7)	36(8)	-13(6)	1(6)	-7(6)
C(112)	36(7)	31(7)	33(7)	-14(6)	5(6)	-1(5)
C(113)	48(9)	67(11)	71(11)	12(9)	9(8)	11(8)
C(114)	29(7)	41(8)	48(8)	-12(6)	-5(6)	-13(6)
C(115)	23(7)	53(9)	74(10)	-34(7)	7(7)	-5(6)
C(116)	25(7)	51(9)	106(14)	-45(9)	-7(8)	-13(6)
C(117)	35(8)	28(7)	88(12)	-27(7)	18(7)	-6(6)
C(118)	41(8)	37(8)	47(8)	-21(6)	14(6)	0(6)
C(119)	46(9)	36(8)	68(11)	-16(7)	7(8)	-9(6)
C(120)	99(13)	46(9)	41(9)	7(7)	10(9)	-23(9)
C(121)	55(9)	34(8)	63(11)	5(7)	-3(8)	13(7)
C(122)	47(9)	36(8)	40(9)	13(6)	0(6)	14(6)
C(123)	55(9)	33(8)	57(9)	-4(7)	3(7)	-14(7)
C(27)	112(19)	62(14)	140(20)	-46(14)	-36(16)	14(13)
F(1)	350(30)	99(12)	211(19)	-54(12)	-69(19)	75(14)
F(2)	69(9)	212(17)	222(18)	-5(14)	-10(10)	-5(10)
F(3)	107(12)	300(20)	400(30)	-270(20)	53(15)	-67(14)
S(1)	47(2)	53(3)	123(4)	-5(3)	-6(3)	-17(2)
O(1)	219(19)	22(6)	260(20)	40(9)	99(17)	8(8)
O(2)	25(7)	330(30)	270(20)	-240(20)	-28(10)	26(10)
O(3)	139(12)	76(8)	113(11)	3(7)	-29(9)	-60(8)
O(4)	156(16)	65(10)	390(30)	-127(16)	-20(19)	11(10)
O(5)	55(8)	187(15)	191(16)	-88(14)	55(10)	-45(9)
O(6)	44(6)	65(7)	140(11)	-42(7)	35(6)	-4(5)
S(2)	56(4)	54(4)	205(10)	-47(5)	44(5)	-12(3)
F(5)	111(15)	230(30)	390(40)	-240(30)	32(19)	-66(16)
F(4)	240(30)	190(30)	270(30)	0(20)	60(30)	-110(20)

Table S41. Hydrogen coordinates (x 10^4) and isotropic displacement parameters (Å^{2}x 10^3) for **11**.

	x	y	z	U(eq)
H(1)	-818	6898	3719	74
H(2)	-3075	7839	3357	77
H(3)	-2917	9338	3489	96
H(4)	-606	9371	3938	105
H(5)	699	7842	4113	64
H(6)	-1871	6504	5495	59
H(7)	-4056	7431	5072	60
H(8)	-3927	8962	5138	88

H(9)	-1614	9013	5654	61
H(13A)	315	8178	7491	163
H(13B)	-978	8317	6969	163
H(13C)	145	8889	6737	163
H(15)	3917	6088	5262	54
H(16)	6227	5397	5774	59
H(17)	6296	5394	7154	70
H(18)	3993	6053	7561	54
H(19)	1671	4500	6658	52
H(20)	3132	4091	5617	65
H(21)	5433	3456	6150	68
H(22)	5426	3508	7534	61
H(23)	3096	4129	7836	49
H(101)	4138	6827	10610	57
H(102)	1890	7676	10814	72
H(103)	1885	9243	10230	60
H(104)	4205	9337	9758	60
H(105)	5589	7865	10006	51
H(106)	3002	6531	9004	54
H(107)	786	7480	9186	54
H(108)	978	9051	8675	63
H(109)	3250	9075	8130	55
H(11A)	4773	8928	6945	102
H(11B)	3791	8263	6914	102
H(11C)	5100	8222	6428	102
H(115)	8872	6330	9251	56
H(116)	11186	5598	8905	67
H(117)	11097	5506	7556	58
H(118)	8772	6068	7030	49
H(119)	6625	4573	8549	59
H(120)	8083	4297	9720	75
H(121)	10475	3697	9370	66
H(122)	10376	3600	8001	56
H(123)	8072	4108	7519	58

Table S42. Torsion angles [°] for **11**.

C(12)-N(1)-N(2)-N(3)	-1.7(14)	C(5)-C(1)-C(2)-C(3)	1.0(18)
C(14)-N(1)-N(2)-N(3)	175.1(10)	Fe(1)-C(1)-C(2)-C(3)	59.5(12)
N(1)-N(2)-N(3)-C(11)	1.0(14)	C(5)-C(1)-C(2)-Fe(1)	-58.5(11)
N(1)-N(2)-N(3)-C(13)	175.3(15)	C(1)-C(2)-C(3)-C(4)	0(2)
C(112)-N(4)-N(5)-N(6)	-1.3(13)	Fe(1)-C(2)-C(3)-C(4)	59.4(13)
C(114)-N(4)-N(5)-N(6)	-176.1(9)	C(1)-C(2)-C(3)-Fe(1)	-59.6(11)
N(4)-N(5)-N(6)-C(111)	1.4(13)	C(2)-C(3)-C(4)-C(5)	-1(2)
N(4)-N(5)-N(6)-C(113)	-172.7(12)	Fe(1)-C(3)-C(4)-C(5)	58.8(13)

C(2)-C(3)-C(4)-Fe(1)	-59.4(13)	C(18)-C(14)-C(15)-C(16)	-0.2(14)
C(3)-C(4)-C(5)-C(1)	1(2)	N(1)-C(14)-C(15)-C(16)	173.8(10)
Fe(1)-C(4)-C(5)-C(1)	59.6(11)	Co(1)-C(14)-C(15)-C(16)	-59.6(9)
C(3)-C(4)-C(5)-Fe(1)	-58.4(13)	C(18)-C(14)-C(15)-Co(1)	59.4(8)
C(2)-C(1)-C(5)-C(4)	-1.4(18)	N(1)-C(14)-C(15)-Co(1)	-126.6(11)
Fe(1)-C(1)-C(5)-C(4)	-59.7(12)	C(14)-C(15)-C(16)-C(17)	-0.4(15)
C(2)-C(1)-C(5)-Fe(1)	58.3(11)	Co(1)-C(15)-C(16)-C(17)	-59.8(9)
C(10)-C(6)-C(7)-C(8)	1.0(18)	C(14)-C(15)-C(16)-Co(1)	59.4(9)
Fe(1)-C(6)-C(7)-C(8)	-58.4(12)	C(15)-C(16)-C(17)-C(18)	0.9(15)
C(10)-C(6)-C(7)-Fe(1)	59.4(10)	Co(1)-C(16)-C(17)-C(18)	-59.1(9)
C(6)-C(7)-C(8)-C(9)	-1(2)	C(15)-C(16)-C(17)-Co(1)	60.0(9)
Fe(1)-C(7)-C(8)-C(9)	-59.7(12)	C(16)-C(17)-C(18)-C(14)	-1.0(14)
C(6)-C(7)-C(8)-Fe(1)	58.8(11)	Co(1)-C(17)-C(18)-C(14)	-59.4(7)
C(7)-C(8)-C(9)-C(10)	0.6(18)	C(16)-C(17)-C(18)-Co(1)	58.4(9)
Fe(1)-C(8)-C(9)-C(10)	-59.2(10)	C(15)-C(14)-C(18)-C(17)	0.8(13)
C(7)-C(8)-C(9)-Fe(1)	59.7(12)	N(1)-C(14)-C(18)-C(17)	-173.3(10)
C(8)-C(9)-C(10)-C(6)	0.1(17)	Co(1)-C(14)-C(18)-C(17)	59.7(8)
Fe(1)-C(9)-C(10)-C(6)	-58.6(10)	C(15)-C(14)-C(18)-Co(1)	-58.9(9)
C(8)-C(9)-C(10)-C(11)	-178.4(14)	N(1)-C(14)-C(18)-Co(1)	127.0(10)
Fe(1)-C(9)-C(10)-C(11)	123.0(14)	C(23)-C(19)-C(20)-C(21)	0.4(15)
C(8)-C(9)-C(10)-Fe(1)	58.6(11)	Co(1)-C(19)-C(20)-C(21)	60.0(10)
C(7)-C(6)-C(10)-C(9)	-0.6(17)	C(23)-C(19)-C(20)-Co(1)	-59.6(9)
Fe(1)-C(6)-C(10)-C(9)	59.2(10)	C(19)-C(20)-C(21)-C(22)	-0.9(16)
C(7)-C(6)-C(10)-C(11)	177.9(13)	Co(1)-C(20)-C(21)-C(22)	59.1(10)
Fe(1)-C(6)-C(10)-C(11)	-122.3(14)	C(19)-C(20)-C(21)-Co(1)	-60.0(9)
C(7)-C(6)-C(10)-Fe(1)	-59.8(11)	C(20)-C(21)-C(22)-C(23)	1.1(15)
N(2)-N(3)-C(11)-C(12)	0.0(16)	Co(1)-C(21)-C(22)-C(23)	60.0(9)
C(13)-N(3)-C(11)-C(12)	-173.6(17)	C(20)-C(21)-C(22)-Co(1)	-58.9(9)
N(2)-N(3)-C(11)-C(10)	177.9(12)	C(20)-C(19)-C(23)-C(22)	0.3(15)
C(13)-N(3)-C(11)-C(10)	4(2)	Co(1)-C(19)-C(23)-C(22)	-59.8(9)
C(9)-C(10)-C(11)-C(12)	-146.3(15)	C(20)-C(19)-C(23)-Co(1)	60.1(9)
C(6)-C(10)-C(11)-C(12)	35(2)	C(21)-C(22)-C(23)-C(19)	-0.9(15)
Fe(1)-C(10)-C(11)-C(12)	-54.0(19)	Co(1)-C(22)-C(23)-C(19)	60.0(9)
C(9)-C(10)-C(11)-N(3)	36(2)	C(21)-C(22)-C(23)-Co(1)	-60.9(9)
C(6)-C(10)-C(11)-N(3)	-142.0(14)	C(105)-C(101)-C(102)-C(103)	2.8(17)
Fe(1)-C(10)-C(11)-N(3)	128.5(12)	Fe(2)-C(101)-C(102)-C(103)	-58.6(11)
N(2)-N(1)-C(12)-C(11)	1.7(14)	C(105)-C(101)-C(102)-Fe(2)	61.4(10)
C(14)-N(1)-C(12)-C(11)	-174.6(11)	C(101)-C(102)-C(103)-C(104)	-2.3(17)
N(2)-N(1)-C(12)-Ag(1)	-178.9(9)	Fe(2)-C(102)-C(103)-C(104)	-60.0(10)
C(14)-N(1)-C(12)-Ag(1)	4.9(18)	C(101)-C(102)-C(103)-Fe(2)	57.7(10)
N(3)-C(11)-C(12)-N(1)	-0.9(14)	C(102)-C(103)-C(104)-C(105)	1.0(16)
C(10)-C(11)-C(12)-N(1)	-178.6(13)	Fe(2)-C(103)-C(104)-C(105)	-59.4(9)
N(3)-C(11)-C(12)-Ag(1)	179.6(9)	C(102)-C(103)-C(104)-Fe(2)	60.4(10)
C(10)-C(11)-C(12)-Ag(1)	2(2)	C(103)-C(104)-C(105)-C(101)	0.7(15)
N(2)-N(1)-C(14)-C(15)	-140.6(12)	Fe(2)-C(104)-C(105)-C(101)	-58.2(9)
C(12)-N(1)-C(14)-C(15)	35.8(18)	C(103)-C(104)-C(105)-Fe(2)	58.9(10)
N(2)-N(1)-C(14)-C(18)	32.7(15)	C(102)-C(101)-C(105)-C(104)	-2.2(16)
C(12)-N(1)-C(14)-C(18)	-151.0(12)	Fe(2)-C(101)-C(105)-C(104)	58.8(9)
N(2)-N(1)-C(14)-Co(1)	126.4(11)	C(102)-C(101)-C(105)-Fe(2)	-61.0(10)
C(12)-N(1)-C(14)-Co(1)	-57.2(17)	C(110)-C(106)-C(107)-C(108)	-2.8(15)

Fe(2)-C(106)-C(107)-C(108)	57.8(9)	C(115)-C(116)-C(117)-C(118)	-2.0(15)
C(110)-C(106)-C(107)-Fe(2)	-60.7(9)	Co(2)-C(116)-C(117)-C(118)	58.4(9)
C(106)-C(107)-C(108)-C(109)	1.4(15)	C(115)-C(116)-C(117)-Co(2)	-60.5(9)
Fe(2)-C(107)-C(108)-C(109)	60.0(9)	C(116)-C(117)-C(118)-C(114)	1.6(15)
C(106)-C(107)-C(108)-Fe(2)	-58.5(10)	Co(2)-C(117)-C(118)-C(114)	60.2(8)
C(107)-C(108)-C(109)-C(110)	0.6(15)	C(116)-C(117)-C(118)-Co(2)	-58.6(10)
Fe(2)-C(108)-C(109)-C(110)	60.5(9)	C(115)-C(114)-C(118)-C(117)	-0.5(14)
C(107)-C(108)-C(109)-Fe(2)	-59.9(9)	N(4)-C(114)-C(118)-C(117)	172.2(11)
C(108)-C(109)-C(110)-C(106)	-2.4(15)	Co(2)-C(114)-C(118)-C(117)	-60.3(8)
Fe(2)-C(109)-C(110)-C(106)	56.9(9)	C(115)-C(114)-C(118)-Co(2)	59.8(9)
C(108)-C(109)-C(110)-C(111)	-179.7(12)	N(4)-C(114)-C(118)-Co(2)	-127.4(12)
Fe(2)-C(109)-C(110)-C(111)	-120.4(13)	C(123)-C(119)-C(120)-C(121)	1.0(15)
C(108)-C(109)-C(110)-Fe(2)	-59.3(9)	Co(2)-C(119)-C(120)-C(121)	-59.3(10)
C(107)-C(106)-C(110)-C(109)	3.2(15)	C(123)-C(119)-C(120)-Co(2)	60.3(9)
Fe(2)-C(106)-C(110)-C(109)	-56.5(9)	C(119)-C(120)-C(121)-C(122)	-0.7(16)
C(107)-C(106)-C(110)-C(111)	-179.4(12)	Co(2)-C(120)-C(121)-C(122)	-60.6(10)
Fe(2)-C(106)-C(110)-C(111)	120.9(12)	C(119)-C(120)-C(121)-Co(2)	60.0(10)
C(107)-C(106)-C(110)-Fe(2)	59.7(9)	C(120)-C(121)-C(122)-C(123)	0.0(16)
N(5)-N(6)-C(111)-C(112)	-1.0(14)	Co(2)-C(121)-C(122)-C(123)	-59.9(10)
C(113)-N(6)-C(111)-C(112)	172.3(13)	C(120)-C(121)-C(122)-Co(2)	59.9(9)
N(5)-N(6)-C(111)-C(110)	-178.0(10)	C(121)-C(122)-C(123)-C(119)	0.6(16)
C(113)-N(6)-C(111)-C(110)	-5(2)	Co(2)-C(122)-C(123)-C(119)	-58.7(9)
C(109)-C(110)-C(111)-N(6)	-45.4(18)	C(121)-C(122)-C(123)-Co(2)	59.3(10)
C(106)-C(110)-C(111)-N(6)	137.7(13)	C(120)-C(119)-C(123)-C(122)	-1.0(15)
Fe(2)-C(110)-C(111)-N(6)	-134.3(11)	Co(2)-C(119)-C(123)-C(122)	58.8(10)
C(109)-C(110)-C(111)-C(112)	138.3(14)	C(120)-C(119)-C(123)-Co(2)	-59.8(9)
C(106)-C(110)-C(111)-C(112)	-39(2)	F(2)-C(27)-S(1)-O(2)	-177.5(18)
Fe(2)-C(110)-C(111)-C(112)	49.4(17)	F(1)-C(27)-S(1)-O(2)	56(2)
N(5)-N(4)-C(112)-C(111)	0.7(13)	F(3)-C(27)-S(1)-O(2)	-65.7(18)
C(114)-N(4)-C(112)-C(111)	174.8(11)	F(2)-C(27)-S(1)-O(1)	-50(2)
N(5)-N(4)-C(112)-Ag(2)	176.7(8)	F(1)-C(27)-S(1)-O(1)	-177(2)
C(114)-N(4)-C(112)-Ag(2)	-9.3(17)	F(3)-C(27)-S(1)-O(1)	61.5(19)
N(6)-C(111)-C(112)-N(4)	0.2(12)	F(2)-C(27)-S(1)-O(3)	67.1(19)
C(110)-C(111)-C(112)-N(4)	176.9(11)	F(1)-C(27)-S(1)-O(3)	-60(2)
N(6)-C(111)-C(112)-Ag(2)	-175.5(9)	F(3)-C(27)-S(1)-O(3)	178.8(15)
C(110)-C(111)-C(112)-Ag(2)	1(2)	O(4)-S(2)-C(28)-F(4)	-178(5)
N(5)-N(4)-C(114)-C(115)	134.8(13)	O(5)-S(2)-C(28)-F(4)	-44(7)
C(112)-N(4)-C(114)-C(115)	-39.3(18)	O(6)-S(2)-C(28)-F(4)	65(7)
N(5)-N(4)-C(114)-C(118)	-37.0(16)	O(4)-S(2)-C(28)-F(5)	42(5)
C(112)-N(4)-C(114)-C(118)	148.9(12)	O(5)-S(2)-C(28)-F(5)	176(3)
N(5)-N(4)-C(114)-Co(2)	-130.1(11)	O(6)-S(2)-C(28)-F(5)	-75(4)
C(112)-N(4)-C(114)-Co(2)	55.8(17)	O(4)-S(2)-C(28)-F(6)	-56(5)
C(118)-C(114)-C(115)-C(116)	-0.7(15)	O(5)-S(2)-C(28)-F(6)	78(4)
N(4)-C(114)-C(115)-C(116)	-173.4(11)	O(6)-S(2)-C(28)-F(6)	-173(3)
Co(2)-C(114)-C(115)-C(116)	58.2(9)	O(6)-S(2A)-C(28A)-F(5A)	-168(3)
C(118)-C(114)-C(115)-Co(2)	-58.9(9)	O(5)-S(2A)-C(28A)-F(5A)	-54(4)
N(4)-C(114)-C(115)-Co(2)	128.3(12)	O(4)-S(2A)-C(28A)-F(5A)	80(4)
C(114)-C(115)-C(116)-C(117)	1.6(15)	O(6)-S(2A)-C(28A)-F(6A)	68(3)
Co(2)-C(115)-C(116)-C(117)	60.0(9)	O(5)-S(2A)-C(28A)-F(6A)	-179(2)
C(114)-C(115)-C(116)-Co(2)	-58.4(9)	O(4)-S(2A)-C(28A)-F(6A)	-44(4)

O(6)-S(2A)-C(28A)-F(4A)	-50(3)	O(4)-S(2A)-C(28A)-F(4A)	-162(3)
O(5)-S(2A)-C(28A)-F(4A)	64(3)		

Triazolylidene complex (12) – "chlorido (1,4-dicobaltoceniumyl-3-methyltriazol-ylidene) copper(I) dimer bis(hexafluoridophosphate) ditriflate"

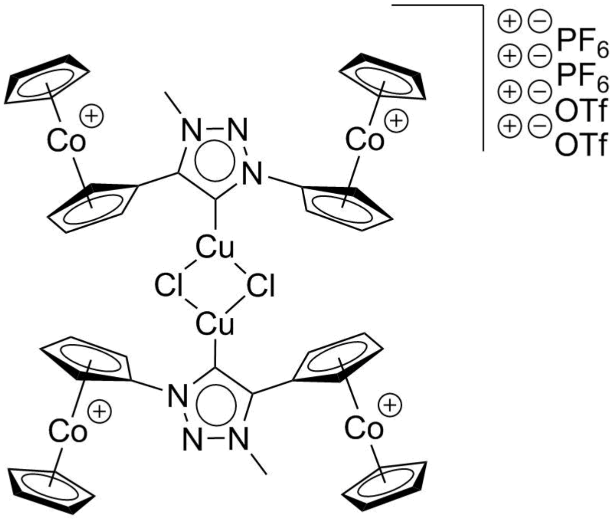

[1,700.67]

$[C_{23}H_{21}N_3Co_2CuCl]_2(PF_6)_2(CF_3SO_3)_2$

Analytical data:

IR (ATR [cm^{-1}]): 3119 (ν_{C-H}), 2961, 2925, 2854, 1705, 1668, 1620, 1554, 1419 ($\nu_{C=C}$), 1248 (ν_{SO3}), 1228 (ν_{CF3}), 1161 (ν_{CF3}), 1030 (ν_{SO3}), 841 (ν_{P-F}), 766, 635, 577 (ν_{P-F}), 559, 518, 476.

^{1}H-NMR (300 MHz, C_3D_6O, [ppm]): δ 4.49 (s, 3H, CH_3 of triazolium), 6.13 (s, 5H, 4-Cc-Cp), 6.17 (s, 5H, 1-Cc-Cp), 6.19 (shoulder, 2H, C3/C4 of substituted 1-Cc-Cp), 6.23 (shoulder, 2H, C3/C4 of substituted 4-Cc-Cp), 6.64 (pseudo-t, 2H, J = 1.8 Hz, C2/C5 of substituted 4-Cc-Cp), 6.87 (pseudo-t, 2H, J = 1.8 Hz, C2/C5 of substituted 1-Cc-Cp).

^{13}C-NMR (75 MHz, C_3D_6O, [ppm]): δ 41.4 (CH_3 of triazolium), 76.3 (C3/C4 of substituted 4-Cc-Cp), 80.7 (C3/C4 of substituted 1-Cc-Cp), 83.0 (C2/C5 of substituted 4-Cc-Cp), 85.1 (C2/C5 of substituted 1-Cc-Cp), 88.3 (4-Cc-Cp), 88.6 (1-Cc-Cp), *not observed* (quart. carbon of substituted 4-Cc-Cp), 105.3 (quart. carbon of substituted 1-Cc-Cp), 120.0 (CF_3 of triflate), 124.3 (CF_3 of triflate), 138.9 (quart. carbon of triazolium), 160.3 (carbene carbon of triazolium).

MS (ESI pos, [m/z]): 703.88 ($[M(CF_3SO_3)]^+$ of the monomer).

Melting point [°C]: 214 (dec).

Spectra

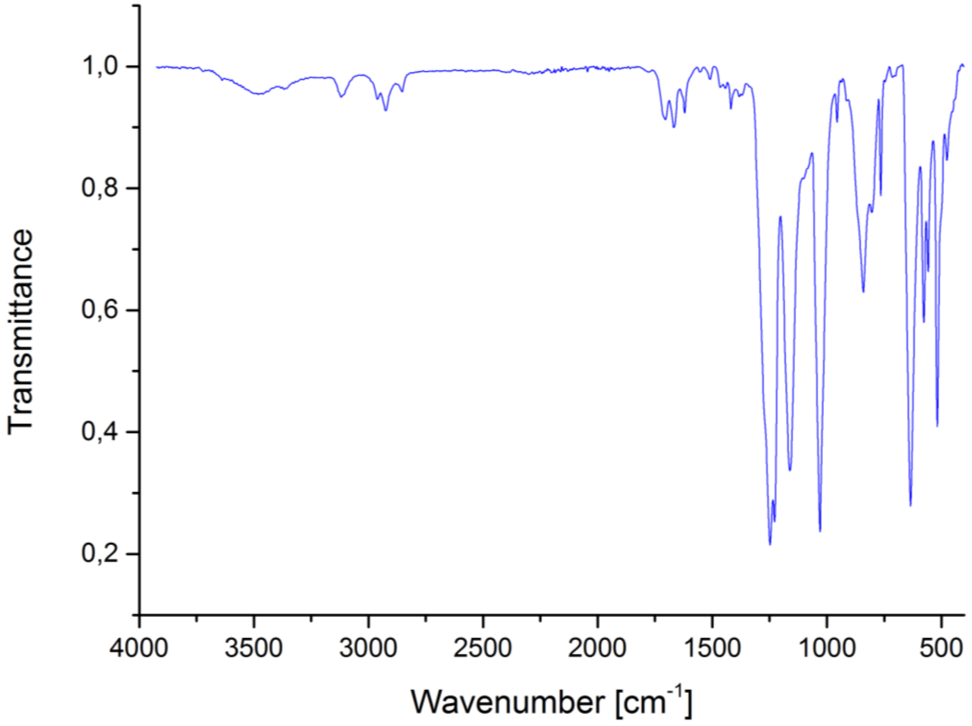

Figure S48. IR-spectrum (ATR, [cm^{-1}]) of “chlorido (1,4-dicobaltoceniumyl-3-methyltriazolylidene) copper(I) dimer bis(hexafluoridophosphate) ditriflate“.

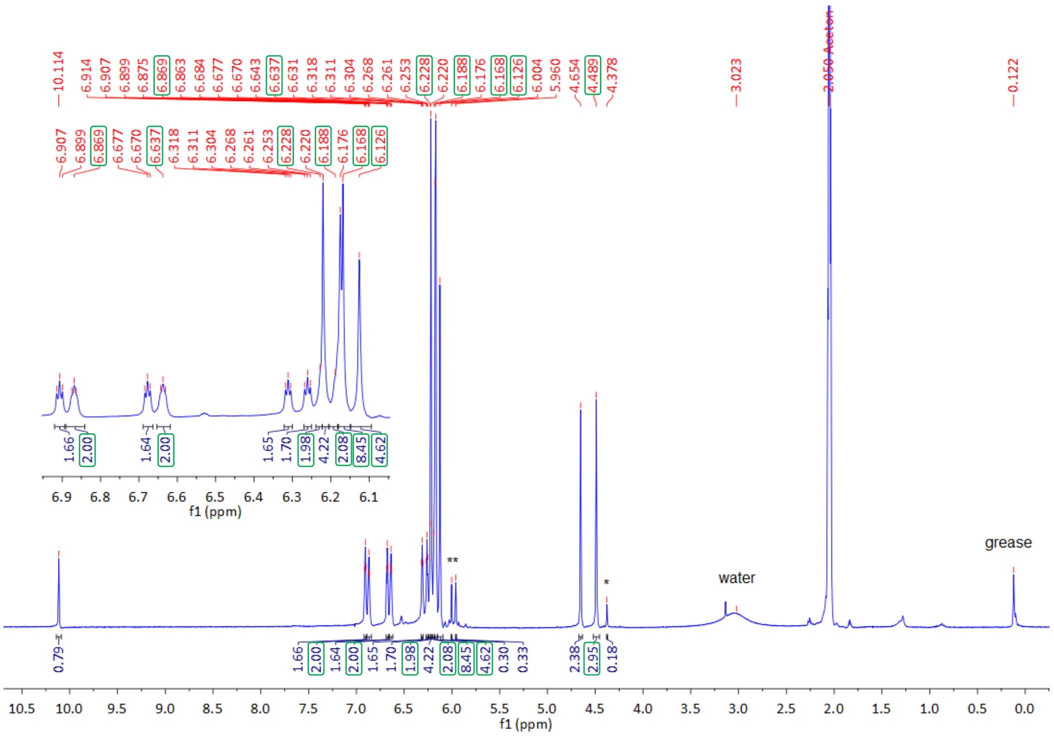

Figure S49. ^{1}H-NMR (300 MHz, C_3D_6O, [ppm]) of “chlorido (1,4-dicobaltoceniumyl-3-methyltriazolylidene) copper(I) dimer bis(hexafluoridophosphate) ditriflate“ (product peaks marked green; educt peaks unmarked).

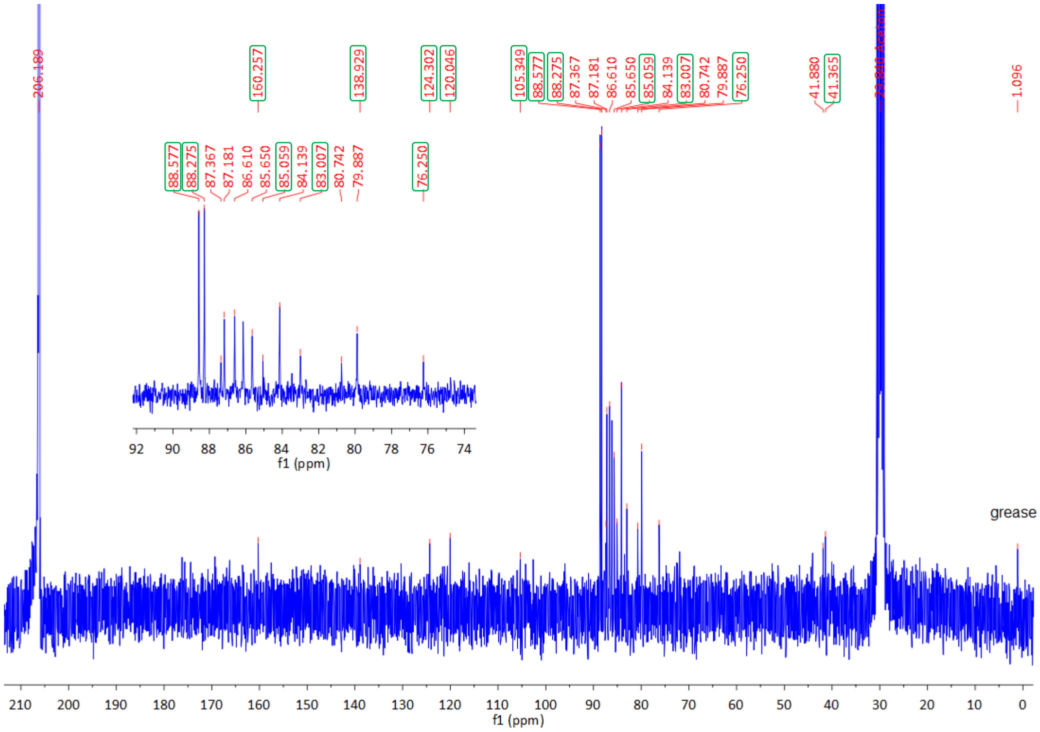

Figure S50. ^{13}C-NMR (75 MHz, C_3D_6O, [ppm]) of “chlorido (1,4-dicobaltoceniumyl-3-methyltriazolylidene) copper(I) dimer bis(hexafluoridophosphate) ditriflate“ (product peaks marked green; educt peaks unmarked).

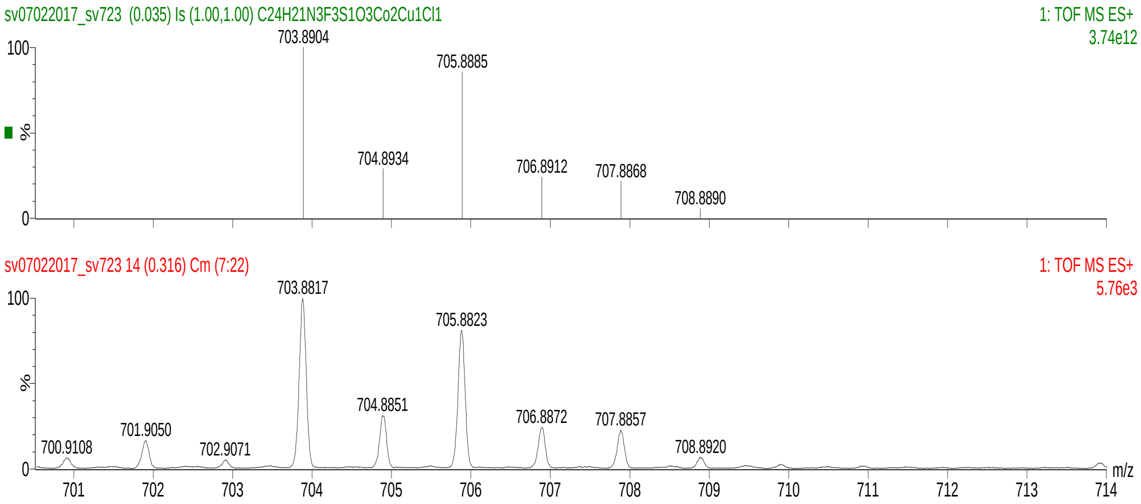

Figure S51. MS (ESI pos, [m/z]; *top*: simulated, *bottom*: experimental) of "chlorido (1,4-dicobaltoceniumyl-3-methyltriazolylidene) copper(I) dimer bis(hexafluoridophosphate) ditriflate".

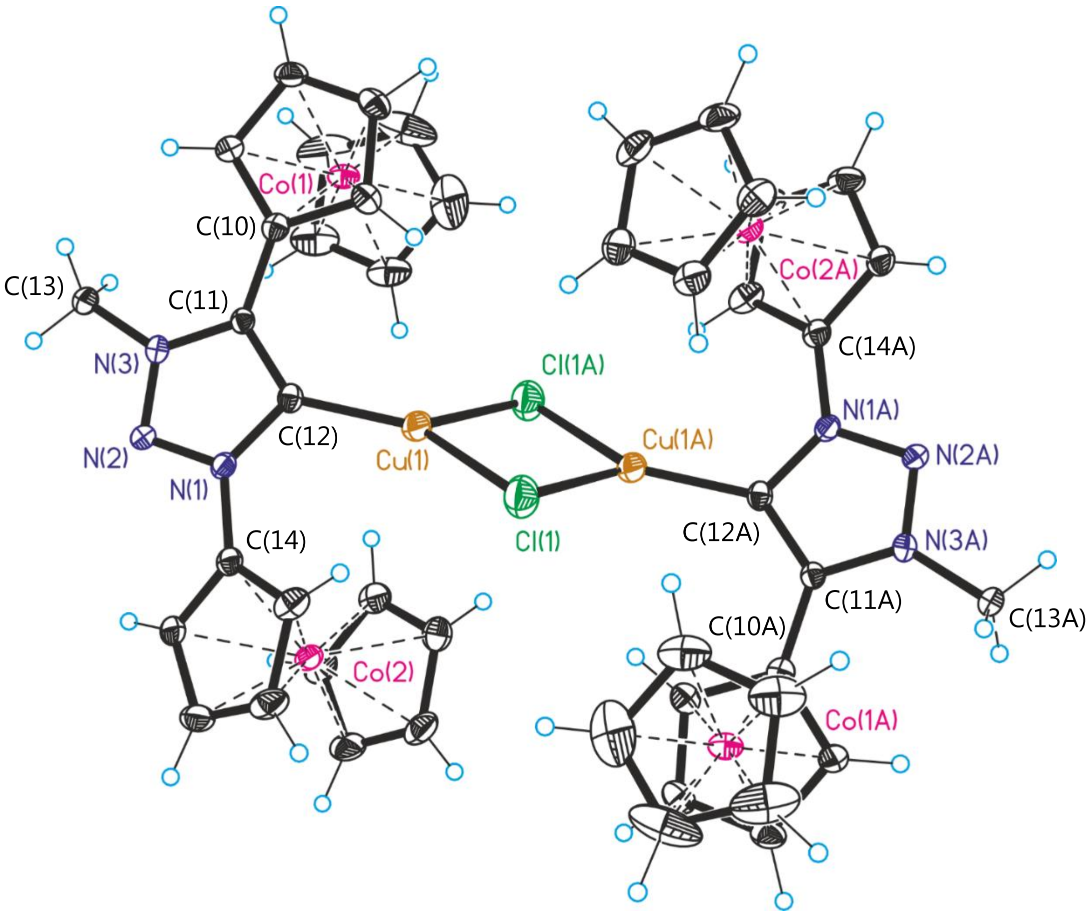

Figure S52. X-ray single crystal structure analysis of "chlorido (1,4-dicobaltoceniumyl-3-methyltriazolylidene) copper(I) dimer bis(hexafluoridophosphate) ditriflate" (counterions omitted for clarity).

X-Ray single crystal structure analysis data

Table S43. Crystal data and structure refinement for **12**.

Empirical formula	0.7 (C23 H21 Cl Co2 Cu) 0.3 (C23 H22 Cl Co2) 1.8 (CF3SO3) 0.2 (PF6)	
Formula weight	834.84	
Temperature	163(2) K	
Wavelength	0.71073 Å	
Crystal system	Monoclinic	
Space group	$P2_1/c$ (no. 14)	
Unit cell dimensions	a = 8.9008(6) Å	α = 90°.
	b = 12.5213(7) Å	ß = 97.103(2)°.
	c = 25.9743(16) Å	γ = 90°.
Volume	2872.6(3) $Å^3$	
Z	4	
Density (calculated)	1.930 Mg/m^3	
Absorption coefficient	1.982 mm^{-1}	
F(000)	1667	
Crystal size	0.180 x 0.140 x 0.040 mm^3	
Theta range for data collection	2.268 to 24.998°.	
Index ranges	-10<h<10, -14<k<14, -30<l<30	
Reflections collected	42549	
Independent reflections	5058 [R(int) = 0.0389]	
Completeness to theta = 24.998°	99.8 %	
Absorption correction	Semi-empirical from equivalents	
Max. and min. transmission	0.914 and 0.777	
Refinement method	Full-matrix least-squares on F^2	
Data / restraints / parameters	5058 / 0 / 424	
Goodness-of-fit on F^2	1.088	
Final R indices [I>2sigma(I)]	R1 = 0.0583, wR2 = 0.1401	
R indices (all data)	R1 = 0.0670, wR2 = 0.1448	
Extinction coefficient	n/a	
Largest diff. peak and hole	1.379 and -0.745 e.$Å^{-3}$	

Table S44. Atomic coordinates (x 10^4) and equivalent isotropic displacement parameters ($Å^2$x 10^3) for **12**. U(eq) is defined as one third of the trace of the orthogonalized U^{ij} tensor.

	x	y	z	U(eq)
Co(1)	8460(1)	-257(1)	6855(1)	27(1)
Co(2)	2502(1)	2953(1)	5373(1)	28(1)
Cu(1)	4446(1)	195(1)	5551(1)	43(1)
N(1)	3303(4)	1478(3)	6337(2)	25(1)
N(2)	3465(5)	1821(3)	6831(2)	26(1)
N(3)	4519(5)	1188(3)	7060(2)	27(1)
C(1)	9902(10)	53(7)	6339(4)	77(3)
C(2)	10711(8)	-184(7)	6826(5)	84(3)
C(3)	10318(9)	545(7)	7176(4)	76(3)
C(4)	9267(8)	1264(5)	6928(3)	58(2)
C(5)	8990(7)	977(5)	6412(3)	50(2)
C(6)	6663(6)	-1107(4)	6526(2)	30(1)
C(7)	7741(6)	-1789(4)	6803(2)	34(1)
C(8)	7949(6)	-1463(4)	7321(2)	36(1)
C(9)	6992(6)	-573(5)	7381(2)	36(1)
C(10)	6168(5)	-351(4)	6881(2)	26(1)
C(11)	5031(5)	464(4)	6730(2)	22(1)
C(13)	5059(7)	1419(5)	7607(2)	36(1)
C(14)	2199(5)	2007(4)	5989(2)	27(1)
C(15)	1428(6)	1569(5)	5523(2)	36(1)
C(16)	352(6)	2355(5)	5317(2)	42(1)
C(17)	493(6)	3251(5)	5641(2)	41(1)
C(18)	1644(6)	3056(5)	6065(2)	32(1)
C(19)	4024(7)	2632(5)	4875(2)	43(1)
C(20)	2901(7)	3344(6)	4642(2)	45(2)
C(21)	2929(7)	4261(5)	4960(2)	47(2)
C(22)	4083(7)	4128(5)	5387(2)	42(1)
C(23)	4750(6)	3115(5)	5329(2)	38(1)
S(2)	1283(2)	6426(1)	6177(1)	47(1)
O(4)	1670(8)	5897(9)	6615(3)	174(5)
O(5)	1034(8)	5789(5)	5706(3)	102(2)
O(6)	169(6)	7259(5)	6132(3)	87(2)
C(25)	2954(8)	7174(6)	6076(3)	51(2)
F(4)	2869(6)	7635(7)	5643(2)	137(3)
F(5)	3311(5)	7867(4)	6457(2)	92(2)
F(6)	4189(5)	6542(5)	6114(2)	91(2)
C(12)	4245(5)	653(4)	6237(2)	25(1)
Cl(1)	3182(2)	-427(1)	4800(1)	53(1)
S(1)	6265(2)	3708(2)	6802(1)	38(1)
O(1)	6459(5)	2704(3)	6579(2)	58(1)
O(2)	6667(5)	3827(5)	7331(2)	68(1)
O(3)	4948(5)	4253(3)	6596(2)	54(1)
C(24)	7695(9)	4484(6)	6529(3)	38(2)

F(1)	7615(5)	5460(3)	6669(2)	71(1)
F(2)	7484(5)	4369(4)	6025(2)	68(1)
F(3)	9070(5)	4086(4)	6698(2)	49(1)
C(12A)	4245(5)	653(4)	6237(2)	25(1)
P(1)	7053(9)	4027(6)	6665(3)	25(1)
F(1A)	6459(5)	2704(3)	6579(2)	58(1)
F(2A)	6667(5)	3827(5)	7331(2)	68(1)
F(3A)	4948(5)	4253(3)	6596(2)	54(1)
F(4A)	8700(20)	3687(17)	6891(8)	53(5)

Table S45. Bond lengths [Å] and angles [°] for **12**.

Co(1)-C(1)	2.004(7)	C(1)-H(1)	0.9500
Co(1)-C(2)	2.016(7)	C(2)-C(3)	1.365(13)
Co(1)-C(5)	2.016(6)	C(2)-H(2)	0.9500
Co(1)-C(6)	2.020(5)	C(3)-C(4)	1.397(10)
Co(1)-C(7)	2.021(5)	C(3)-H(3)	0.9500
Co(1)-C(8)	2.022(5)	C(4)-C(5)	1.379(10)
Co(1)-C(3)	2.025(7)	C(4)-H(4)	0.9500
Co(1)-C(4)	2.036(6)	C(5)-H(5)	0.9500
Co(1)-C(9)	2.042(6)	C(6)-C(7)	1.412(7)
Co(1)-C(10)	2.053(5)	C(6)-C(10)	1.428(7)
Co(2)-C(21)	2.021(6)	C(6)-H(6)	0.9500
Co(2)-C(19)	2.027(6)	C(7)-C(8)	1.397(8)
Co(2)-C(23)	2.028(5)	C(7)-H(7)	0.9500
Co(2)-C(17)	2.032(6)	C(8)-C(9)	1.423(7)
Co(2)-C(14)	2.033(5)	C(8)-H(8)	0.9500
Co(2)-C(22)	2.033(6)	C(9)-C(10)	1.436(7)
Co(2)-C(20)	2.035(6)	C(9)-H(9)	0.9500
Co(2)-C(15)	2.039(6)	C(10)-C(11)	1.457(7)
Co(2)-C(18)	2.040(5)	C(11)-C(12A)	1.402(7)
Co(2)-C(16)	2.043(5)	C(11)-C(12)	1.402(7)
Cu(1)-C(12A)	1.900(5)	C(13)-H(13A)	0.9800
Cu(1)-C(12)	1.900(5)	C(13)-H(13B)	0.9800
Cu(1)-Cl(1)	2.2670(19)	C(13)-H(13C)	0.9800
Cu(1)-Cl(1)#1	2.418(2)	C(14)-C(15)	1.425(7)
N(1)-N(2)	1.343(5)	C(14)-C(18)	1.425(8)
N(1)-C(12A)	1.376(6)	C(15)-C(16)	1.430(8)
N(1)-C(12)	1.376(6)	C(15)-H(15)	0.9500
N(1)-C(14)	1.416(6)	C(16)-C(17)	1.400(9)
N(2)-N(3)	1.314(6)	C(16)-H(16)	0.9500
N(3)-C(11)	1.364(6)	C(17)-C(18)	1.429(8)
N(3)-C(13)	1.470(6)	C(17)-H(17)	0.9500
C(1)-C(2)	1.407(13)	C(18)-H(18)	0.9500
C(1)-C(5)	1.439(11)	C(19)-C(23)	1.409(8)

C(19)-C(20)	1.418(9)	C(25)-F(6)	1.348(8)
C(19)-H(19)	0.9500	Cl(1)-Cu(1)#1	2.418(2)
C(20)-C(21)	1.412(9)	S(1)-O(2)	1.384(5)
C(20)-H(20)	0.9500	S(1)-O(1)	1.403(5)
C(21)-C(22)	1.425(8)	S(1)-O(3)	1.405(4)
C(21)-H(21)	0.9500	S(1)-C(24)	1.814(7)
C(22)-C(23)	1.417(9)	C(24)-F(1)	1.280(9)
C(22)-H(22)	0.9500	C(24)-F(2)	1.308(8)
C(23)-H(23)	0.9500	C(24)-F(3)	1.344(8)
S(2)-O(4)	1.324(6)	C(12A)-H(12A)	0.9500
S(2)-O(6)	1.434(6)	P(1)-F(4A)	1.57(2)
S(2)-O(5)	1.455(6)	P(1)-F(1A)	1.744(9)
S(2)-C(25)	1.805(7)	P(1)-F(2A)	1.820(10)
C(25)-F(4)	1.258(8)	P(1)-F(3A)	1.881(9)
C(25)-F(5)	1.326(8)		

C(1)-Co(1)-C(2)	41.0(4)	C(6)-Co(1)-C(9)	69.0(2)
C(1)-Co(1)-C(5)	42.0(3)	C(7)-Co(1)-C(9)	68.8(2)
C(2)-Co(1)-C(5)	68.9(3)	C(8)-Co(1)-C(9)	41.0(2)
C(1)-Co(1)-C(6)	111.0(3)	C(3)-Co(1)-C(9)	112.2(3)
C(2)-Co(1)-C(6)	138.3(4)	C(4)-Co(1)-C(9)	111.5(3)
C(5)-Co(1)-C(6)	113.1(2)	C(1)-Co(1)-C(10)	139.0(3)
C(1)-Co(1)-C(7)	111.3(3)	C(2)-Co(1)-C(10)	179.3(3)
C(2)-Co(1)-C(7)	110.4(3)	C(5)-Co(1)-C(10)	111.5(2)
C(5)-Co(1)-C(7)	141.6(3)	C(6)-Co(1)-C(10)	41.0(2)
C(6)-Co(1)-C(7)	40.9(2)	C(7)-Co(1)-C(10)	68.9(2)
C(1)-Co(1)-C(8)	139.0(3)	C(8)-Co(1)-C(10)	68.8(2)
C(2)-Co(1)-C(8)	110.8(3)	C(3)-Co(1)-C(10)	141.2(4)
C(5)-Co(1)-C(8)	178.0(3)	C(4)-Co(1)-C(10)	113.0(3)
C(6)-Co(1)-C(8)	68.6(2)	C(9)-Co(1)-C(10)	41.1(2)
C(7)-Co(1)-C(8)	40.4(2)	C(21)-Co(2)-C(19)	68.7(3)
C(1)-Co(1)-C(3)	67.7(4)	C(21)-Co(2)-C(23)	68.7(2)
C(2)-Co(1)-C(3)	39.5(4)	C(19)-Co(2)-C(23)	40.7(2)
C(5)-Co(1)-C(3)	67.5(3)	C(21)-Co(2)-C(17)	105.3(2)
C(6)-Co(1)-C(3)	177.6(3)	C(19)-Co(2)-C(17)	160.3(2)
C(7)-Co(1)-C(3)	137.3(3)	C(23)-Co(2)-C(17)	156.5(3)
C(8)-Co(1)-C(3)	110.9(3)	C(21)-Co(2)-C(14)	160.4(2)
C(1)-Co(1)-C(4)	68.5(3)	C(19)-Co(2)-C(14)	123.4(2)
C(2)-Co(1)-C(4)	67.7(3)	C(23)-Co(2)-C(14)	109.3(2)
C(5)-Co(1)-C(4)	39.8(3)	C(17)-Co(2)-C(14)	68.3(2)
C(6)-Co(1)-C(4)	141.6(2)	C(21)-Co(2)-C(22)	41.2(2)
C(7)-Co(1)-C(4)	177.5(3)	C(19)-Co(2)-C(22)	68.8(3)
C(8)-Co(1)-C(4)	138.2(3)	C(23)-Co(2)-C(22)	40.8(2)
C(3)-Co(1)-C(4)	40.2(3)	C(17)-Co(2)-C(22)	119.8(3)
C(1)-Co(1)-C(9)	180.0(4)	C(14)-Co(2)-C(22)	124.5(2)
C(2)-Co(1)-C(9)	139.0(4)	C(21)-Co(2)-C(20)	40.7(3)
C(5)-Co(1)-C(9)	138.0(3)	C(19)-Co(2)-C(20)	40.9(2)

C(23)-Co(2)-C(20)	68.6(2)
C(17)-Co(2)-C(20)	122.5(2)
C(14)-Co(2)-C(20)	158.3(3)
C(22)-Co(2)-C(20)	69.0(3)
C(21)-Co(2)-C(15)	156.0(2)
C(19)-Co(2)-C(15)	108.6(3)
C(23)-Co(2)-C(15)	125.8(2)
C(17)-Co(2)-C(15)	68.8(3)
C(14)-Co(2)-C(15)	41.0(2)
C(22)-Co(2)-C(15)	162.1(2)
C(20)-Co(2)-C(15)	121.5(3)
C(21)-Co(2)-C(18)	122.2(3)
C(19)-Co(2)-C(18)	158.0(2)
C(23)-Co(2)-C(18)	121.5(2)
C(17)-Co(2)-C(18)	41.1(2)
C(14)-Co(2)-C(18)	41.0(2)
C(22)-Co(2)-C(18)	105.8(2)
C(20)-Co(2)-C(18)	159.0(2)
C(15)-Co(2)-C(18)	69.6(2)
C(21)-Co(2)-C(16)	119.6(2)
C(19)-Co(2)-C(16)	125.1(3)
C(23)-Co(2)-C(16)	162.6(3)
C(17)-Co(2)-C(16)	40.2(3)
C(14)-Co(2)-C(16)	68.3(2)
C(22)-Co(2)-C(16)	155.0(3)
C(20)-Co(2)-C(16)	106.9(2)
C(15)-Co(2)-C(16)	41.0(2)
C(18)-Co(2)-C(16)	68.8(2)
C(12)-Cu(1)-Cl(1)	144.75(16)
C(12)-Cu(1)-Cl(1)#1	120.30(16)
Cl(1)-Cu(1)-Cl(1)#1	94.59(7)
N(2)-N(1)-C(12A)	115.2(4)
N(2)-N(1)-C(12)	115.2(4)
N(2)-N(1)-C(14)	116.4(4)
C(12A)-N(1)-C(14)	128.4(4)
C(12)-N(1)-C(14)	128.4(4)
N(3)-N(2)-N(1)	103.0(4)
N(2)-N(3)-C(11)	113.1(4)
N(2)-N(3)-C(13)	116.7(4)
C(11)-N(3)-C(13)	130.0(4)
C(2)-C(1)-C(5)	106.6(8)
C(2)-C(1)-Co(1)	70.0(4)
C(5)-C(1)-Co(1)	69.5(4)
C(2)-C(1)-H(1)	126.7
C(5)-C(1)-H(1)	126.7
Co(1)-C(1)-H(1)	125.4
C(3)-C(2)-C(1)	108.2(7)
C(3)-C(2)-Co(1)	70.6(4)
C(1)-C(2)-Co(1)	69.0(4)
C(3)-C(2)-H(2)	125.9
C(1)-C(2)-H(2)	125.9
Co(1)-C(2)-H(2)	126.0
C(2)-C(3)-C(4)	109.6(8)
C(2)-C(3)-Co(1)	69.9(4)
C(4)-C(3)-Co(1)	70.3(4)
C(2)-C(3)-H(3)	125.2
C(4)-C(3)-H(3)	125.2
Co(1)-C(3)-H(3)	126.2
C(5)-C(4)-C(3)	108.0(8)
C(5)-C(4)-Co(1)	69.3(4)
C(3)-C(4)-Co(1)	69.4(4)
C(5)-C(4)-H(4)	126.0
C(3)-C(4)-H(4)	126.0
Co(1)-C(4)-H(4)	126.8
C(4)-C(5)-C(1)	107.5(7)
C(4)-C(5)-Co(1)	70.9(4)
C(1)-C(5)-Co(1)	68.6(4)
C(4)-C(5)-H(5)	126.2
C(1)-C(5)-H(5)	126.2
Co(1)-C(5)-H(5)	125.9
C(7)-C(6)-C(10)	108.5(5)
C(7)-C(6)-Co(1)	69.6(3)
C(10)-C(6)-Co(1)	70.7(3)
C(7)-C(6)-H(6)	125.8
C(10)-C(6)-H(6)	125.8
Co(1)-C(6)-H(6)	125.6
C(8)-C(7)-C(6)	108.3(5)
C(8)-C(7)-Co(1)	69.8(3)
C(6)-C(7)-Co(1)	69.5(3)
C(8)-C(7)-H(7)	125.9
C(6)-C(7)-H(7)	125.9
Co(1)-C(7)-H(7)	126.4
C(7)-C(8)-C(9)	108.9(5)
C(7)-C(8)-Co(1)	69.7(3)
C(9)-C(8)-Co(1)	70.3(3)
C(7)-C(8)-H(8)	125.6
C(9)-C(8)-H(8)	125.6
Co(1)-C(8)-H(8)	126.0
C(8)-C(9)-C(10)	107.4(5)
C(8)-C(9)-Co(1)	68.8(3)
C(10)-C(9)-Co(1)	69.9(3)
C(8)-C(9)-H(9)	126.3
C(10)-C(9)-H(9)	126.3
Co(1)-C(9)-H(9)	126.6
C(6)-C(10)-C(9)	106.9(4)
C(6)-C(10)-C(11)	123.3(4)
C(9)-C(10)-C(11)	129.7(5)
C(6)-C(10)-Co(1)	68.3(3)
C(9)-C(10)-Co(1)	69.1(3)
C(11)-C(10)-Co(1)	127.7(3)

N(3)-C(11)-C(12A)	107.1(4)	C(21)-C(20)-H(20)	126.2
N(3)-C(11)-C(12)	107.1(4)	C(19)-C(20)-H(20)	126.2
N(3)-C(11)-C(10)	124.8(4)	Co(2)-C(20)-H(20)	127.0
C(12A)-C(11)-C(10)	128.0(4)	C(20)-C(21)-C(22)	108.5(5)
C(12)-C(11)-C(10)	128.0(4)	C(20)-C(21)-Co(2)	70.1(3)
N(3)-C(13)-H(13A)	109.5	C(22)-C(21)-Co(2)	69.9(3)
N(3)-C(13)-H(13B)	109.5	C(20)-C(21)-H(21)	125.7
H(13A)-C(13)-H(13B)	109.5	C(22)-C(21)-H(21)	125.7
N(3)-C(13)-H(13C)	109.5	Co(2)-C(21)-H(21)	125.8
H(13A)-C(13)-H(13C)	109.5	C(23)-C(22)-C(21)	107.1(6)
H(13B)-C(13)-H(13C)	109.5	C(23)-C(22)-Co(2)	69.4(3)
N(1)-C(14)-C(15)	125.9(5)	C(21)-C(22)-Co(2)	69.0(3)
N(1)-C(14)-C(18)	124.6(5)	C(23)-C(22)-H(22)	126.5
C(15)-C(14)-C(18)	109.5(5)	C(21)-C(22)-H(22)	126.5
N(1)-C(14)-Co(2)	128.8(3)	Co(2)-C(22)-H(22)	126.7
C(15)-C(14)-Co(2)	69.7(3)	C(19)-C(23)-C(22)	108.5(5)
C(18)-C(14)-Co(2)	69.8(3)	C(19)-C(23)-Co(2)	69.6(3)
C(14)-C(15)-C(16)	106.4(5)	C(22)-C(23)-Co(2)	69.8(3)
C(14)-C(15)-Co(2)	69.3(3)	C(19)-C(23)-H(23)	125.7
C(16)-C(15)-Co(2)	69.6(3)	C(22)-C(23)-H(23)	125.7
C(14)-C(15)-H(15)	126.8	Co(2)-C(23)-H(23)	126.5
C(16)-C(15)-H(15)	126.8	O(4)-S(2)-O(6)	122.6(6)
Co(2)-C(15)-H(15)	125.9	O(4)-S(2)-O(5)	116.4(6)
C(17)-C(16)-C(15)	108.7(5)	O(6)-S(2)-O(5)	107.4(4)
C(17)-C(16)-Co(2)	69.5(3)	O(4)-S(2)-C(25)	104.6(3)
C(15)-C(16)-Co(2)	69.4(3)	O(6)-S(2)-C(25)	100.6(4)
C(17)-C(16)-H(16)	125.7	O(5)-S(2)-C(25)	101.8(4)
C(15)-C(16)-H(16)	125.7	F(4)-C(25)-F(5)	110.5(7)
Co(2)-C(16)-H(16)	127.1	F(4)-C(25)-F(6)	107.1(6)
C(16)-C(17)-C(18)	109.2(5)	F(5)-C(25)-F(6)	102.2(6)
C(16)-C(17)-Co(2)	70.3(3)	F(4)-C(25)-S(2)	114.1(5)
C(18)-C(17)-Co(2)	69.8(3)	F(5)-C(25)-S(2)	110.9(5)
C(16)-C(17)-H(17)	125.4	F(6)-C(25)-S(2)	111.3(5)
C(18)-C(17)-H(17)	125.4	N(1)-C(12)-C(11)	101.5(4)
Co(2)-C(17)-H(17)	126.1	N(1)-C(12)-Cu(1)	122.3(3)
C(14)-C(18)-C(17)	106.2(5)	C(11)-C(12)-Cu(1)	135.4(4)
C(14)-C(18)-Co(2)	69.2(3)	Cu(1)-Cl(1)-Cu(1)#1	85.41(7)
C(17)-C(18)-Co(2)	69.2(3)	O(2)-S(1)-O(1)	118.5(3)
C(14)-C(18)-H(18)	126.9	O(2)-S(1)-O(3)	115.3(3)
C(17)-C(18)-H(18)	126.9	O(1)-S(1)-O(3)	114.7(3)
Co(2)-C(18)-H(18)	126.2	O(2)-S(1)-C(24)	102.8(3)
C(23)-C(19)-C(20)	108.2(6)	O(1)-S(1)-C(24)	101.0(3)
C(23)-C(19)-Co(2)	69.7(3)	O(3)-S(1)-C(24)	100.6(3)
C(20)-C(19)-Co(2)	69.9(3)	F(1)-C(24)-F(2)	112.6(6)
C(23)-C(19)-H(19)	125.9	F(1)-C(24)-F(3)	110.0(6)
C(20)-C(19)-H(19)	125.9	F(2)-C(24)-F(3)	107.3(6)
Co(2)-C(19)-H(19)	126.1	F(1)-C(24)-S(1)	109.5(5)
C(21)-C(20)-C(19)	107.6(5)	F(2)-C(24)-S(1)	108.1(5)
C(21)-C(20)-Co(2)	69.1(3)	F(3)-C(24)-S(1)	109.2(5)
C(19)-C(20)-Co(2)	69.3(3)	N(1)-C(12A)-C(11)	101.5(4)

N(1)-C(12A)-Cu(1)	122.3(3)	F(4A)-P(1)-F(2A)	83.5(9)
C(11)-C(12A)-Cu(1)	135.4(4)	F(1A)-P(1)-F(2A)	84.4(5)
N(1)-C(12A)-H(12A)	129.2	F(4A)-P(1)-F(3A)	161.9(11)
C(11)-C(12A)-H(12A)	129.2	F(1A)-P(1)-F(3A)	81.3(4)
Cu(1)-C(12A)-H(12A)	9.8	F(2A)-P(1)-F(3A)	79.0(4)
F(4A)-P(1)-F(1A)	92.6(9)		

Symmetry transformations used to generate equivalent atoms: #1 -x+1,-y,-z+1

Table S46. Anisotropic displacement parameters ($Å^2$x 10^3) for **12**. The anisotropic displacement factor exponent takes the form: $-2p^2$ [h^2 $a^{*2}U^{11}$ + ... + 2 h k a* b* U^{12}].

	U^{11}	U^{22}	U^{33}	U^{23}	U^{13}	U^{12}
Co(1)	23(1)	24(1)	35(1)	6(1)	0(1)	-2(1)
Co(2)	24(1)	38(1)	23(1)	7(1)	2(1)	2(1)
Cu(1)	67(1)	32(1)	28(1)	-2(1)	2(1)	13(1)
N(1)	25(2)	30(2)	19(2)	1(2)	2(2)	0(2)
N(2)	30(2)	30(2)	21(2)	3(2)	4(2)	6(2)
N(3)	30(2)	30(2)	21(2)	-1(2)	7(2)	3(2)
C(1)	75(6)	73(6)	97(6)	-6(5)	60(5)	-35(5)
C(2)	28(4)	54(5)	171(10)	41(6)	17(5)	2(3)
C(3)	54(4)	62(5)	101(7)	24(5)	-35(4)	-29(4)
C(4)	53(4)	33(3)	83(5)	12(3)	-11(4)	-18(3)
C(5)	33(3)	46(4)	70(5)	31(3)	7(3)	-10(3)
C(6)	27(3)	26(3)	35(3)	-5(2)	2(2)	-4(2)
C(7)	33(3)	20(3)	48(3)	-2(2)	10(2)	-2(2)
C(8)	37(3)	31(3)	41(3)	14(2)	9(2)	7(2)
C(9)	45(3)	37(3)	28(3)	7(2)	8(2)	13(3)
C(10)	27(2)	23(2)	27(2)	2(2)	6(2)	-6(2)
C(11)	25(2)	20(2)	22(2)	0(2)	6(2)	-2(2)
C(13)	47(3)	40(3)	20(3)	-2(2)	1(2)	14(3)
C(14)	22(2)	35(3)	25(2)	5(2)	8(2)	0(2)
C(15)	29(3)	46(3)	32(3)	7(2)	-1(2)	-9(2)
C(16)	23(3)	65(4)	37(3)	15(3)	-4(2)	-3(3)
C(17)	31(3)	51(4)	43(3)	18(3)	8(2)	12(3)
C(18)	29(3)	44(3)	26(3)	4(2)	9(2)	7(2)
C(19)	42(3)	51(4)	38(3)	7(3)	18(3)	3(3)
C(20)	42(3)	66(4)	28(3)	14(3)	6(2)	-2(3)
C(21)	50(4)	47(4)	44(3)	26(3)	13(3)	8(3)
C(22)	43(3)	43(3)	40(3)	9(3)	10(3)	-11(3)
C(23)	27(3)	51(4)	39(3)	11(3)	9(2)	-1(2)
S(2)	29(1)	52(1)	60(1)	5(1)	3(1)	-2(1)
O(4)	101(5)	283(12)	120(6)	138(7)	-54(5)	-115(7)

O(5)	94(5)	87(5)	119(5)	-53(4)	-5(4)	-7(4)
O(6)	41(3)	103(5)	118(5)	-40(4)	6(3)	12(3)
C(25)	56(4)	54(4)	46(4)	14(3)	10(3)	-7(3)
F(4)	76(3)	244(8)	90(4)	96(5)	4(3)	-25(4)
F(5)	60(3)	86(3)	126(4)	-16(3)	-5(3)	-25(3)
F(6)	42(2)	107(4)	127(4)	15(3)	21(3)	13(2)
C(12)	27(2)	24(2)	24(2)	-3(2)	5(2)	-3(2)
Cl(1)	55(1)	60(1)	44(1)	-10(1)	7(1)	1(1)
S(1)	44(1)	34(1)	38(1)	8(1)	12(1)	2(1)
O(1)	54(3)	31(2)	91(4)	0(2)	17(2)	1(2)
O(2)	58(3)	100(4)	44(3)	14(3)	-3(2)	-18(3)
O(3)	51(2)	50(3)	59(3)	1(2)	-8(2)	6(2)
C(24)	44(4)	37(4)	34(4)	16(3)	7(3)	-13(4)
F(1)	75(3)	47(2)	88(3)	-4(2)	5(2)	4(2)
F(2)	62(2)	101(3)	43(2)	6(2)	7(2)	4(2)
F(3)	28(2)	66(3)	52(3)	7(3)	4(2)	8(2)
C(12A)	27(2)	24(2)	24(2)	-3(2)	5(2)	-3(2)
F(1A)	54(3)	31(2)	91(4)	0(2)	17(2)	1(2)
F(2A)	58(3)	100(4)	44(3)	14(3)	-3(2)	-18(3)
F(3A)	51(2)	50(3)	59(3)	1(2)	-8(2)	6(2)

Table S47. Hydrogen coordinates (x 10^4) and isotropic displacement parameters (Å^{2}x 10^3) for **12**.

	x	y	z	U(eq)
H(1)	9948	-323	6024	93
H(2)	11411	-754	6898	101
H(3)	10701	561	7534	91
H(4)	8822	1848	7087	70
H(5)	8319	1326	6153	60
H(6)	6323	-1145	6165	35
H(7)	8239	-2369	6660	40
H(8)	8620	-1784	7590	43
H(9)	6913	-194	7693	43
H(13A)	4452	1996	7730	54
H(13B)	6124	1637	7638	54
H(13C)	4962	777	7816	54
H(15)	1595	890	5378	43
H(16)	-342	2282	5010	51
H(17)	-86	3888	5589	49
H(18)	1974	3529	6342	39
H(19)	4249	1948	4747	51
H(20)	2248	3226	4330	54
H(21)	2286	4866	4899	56

H(22)	4353	4625	5659	50
H(23)	5552	2812	5558	46
H(12A)	4339	303	5918	30

Table S48. Torsion angles [°] for **12**.

C(12A)-N(1)-N(2)-N(3)	-1.4(5)	N(2)-N(3)-C(11)-C(12A)	0.7(6)
C(12)-N(1)-N(2)-N(3)	-1.4(5)	C(13)-N(3)-C(11)-C(12A)	-173.1(5)
C(14)-N(1)-N(2)-N(3)	178.9(4)	N(2)-N(3)-C(11)-C(12)	0.7(6)
N(1)-N(2)-N(3)-C(11)	0.3(5)	C(13)-N(3)-C(11)-C(12)	-173.1(5)
N(1)-N(2)-N(3)-C(13)	175.0(4)	N(2)-N(3)-C(11)-C(10)	-178.4(4)
C(5)-C(1)-C(2)-C(3)	0.2(8)	C(13)-N(3)-C(11)-C(10)	7.8(8)
Co(1)-C(1)-C(2)-C(3)	-60.0(5)	C(6)-C(10)-C(11)-N(3)	179.1(5)
C(5)-C(1)-C(2)-Co(1)	60.1(4)	C(9)-C(10)-C(11)-N(3)	-0.8(8)
C(1)-C(2)-C(3)-C(4)	-0.3(9)	Co(1)-C(10)-C(11)-N(3)	-93.9(5)
Co(1)-C(2)-C(3)-C(4)	-59.3(5)	C(6)-C(10)-C(11)-C(12A)	0.1(8)
C(1)-C(2)-C(3)-Co(1)	59.0(5)	C(9)-C(10)-C(11)-C(12A)	-179.7(5)
C(2)-C(3)-C(4)-C(5)	0.3(8)	Co(1)-C(10)-C(11)-C(12A)	87.2(6)
Co(1)-C(3)-C(4)-C(5)	-58.8(5)	C(6)-C(10)-C(11)-C(12)	0.1(8)
C(2)-C(3)-C(4)-Co(1)	59.0(5)	C(9)-C(10)-C(11)-C(12)	-179.7(5)
C(3)-C(4)-C(5)-C(1)	-0.1(7)	Co(1)-C(10)-C(11)-C(12)	87.2(6)
Co(1)-C(4)-C(5)-C(1)	-59.0(4)	N(2)-N(1)-C(14)-C(15)	-154.9(5)
C(3)-C(4)-C(5)-Co(1)	58.8(5)	C(12A)-N(1)-C(14)-C(15)	25.5(8)
C(2)-C(1)-C(5)-C(4)	0.0(7)	C(12)-N(1)-C(14)-C(15)	25.5(8)
Co(1)-C(1)-C(5)-C(4)	60.4(4)	N(2)-N(1)-C(14)-C(18)	22.3(7)
C(2)-C(1)-C(5)-Co(1)	-60.5(5)	C(12A)-N(1)-C(14)-C(18)	-157.3(5)
C(10)-C(6)-C(7)-C(8)	-1.0(6)	C(12)-N(1)-C(14)-C(18)	-157.3(5)
Co(1)-C(6)-C(7)-C(8)	59.3(4)	N(2)-N(1)-C(14)-Co(2)	113.2(5)
C(10)-C(6)-C(7)-Co(1)	-60.2(3)	C(12A)-N(1)-C(14)-Co(2)	-66.4(7)
C(6)-C(7)-C(8)-C(9)	0.5(6)	C(12)-N(1)-C(14)-Co(2)	-66.4(7)
Co(1)-C(7)-C(8)-C(9)	59.5(4)	N(1)-C(14)-C(15)-C(16)	176.1(5)
C(6)-C(7)-C(8)-Co(1)	-59.1(4)	C(18)-C(14)-C(15)-C(16)	-1.4(6)
C(7)-C(8)-C(9)-C(10)	0.2(6)	Co(2)-C(14)-C(15)-C(16)	-60.0(4)
Co(1)-C(8)-C(9)-C(10)	59.4(4)	N(1)-C(14)-C(15)-Co(2)	-123.9(5)
C(7)-C(8)-C(9)-Co(1)	-59.2(4)	C(18)-C(14)-C(15)-Co(2)	58.6(4)
C(7)-C(6)-C(10)-C(9)	1.1(6)	C(14)-C(15)-C(16)-C(17)	1.3(6)
Co(1)-C(6)-C(10)-C(9)	-58.4(4)	Co(2)-C(15)-C(16)-C(17)	-58.5(4)
C(7)-C(6)-C(10)-C(11)	-178.8(4)	C(14)-C(15)-C(16)-Co(2)	59.8(4)
Co(1)-C(6)-C(10)-C(11)	121.7(4)	C(15)-C(16)-C(17)-C(18)	-0.7(6)
C(7)-C(6)-C(10)-Co(1)	59.5(3)	Co(2)-C(16)-C(17)-C(18)	-59.1(4)
C(8)-C(9)-C(10)-C(6)	-0.8(6)	C(15)-C(16)-C(17)-Co(2)	58.4(4)
Co(1)-C(9)-C(10)-C(6)	57.9(3)	N(1)-C(14)-C(18)-C(17)	-176.5(5)
C(8)-C(9)-C(10)-C(11)	179.1(5)	C(15)-C(14)-C(18)-C(17)	1.0(6)
Co(1)-C(9)-C(10)-C(11)	-122.2(5)	Co(2)-C(14)-C(18)-C(17)	59.6(4)
C(8)-C(9)-C(10)-Co(1)	-58.7(4)	N(1)-C(14)-C(18)-Co(2)	123.9(5)

C(15)-C(14)-C(18)-Co(2)	-58.6(4)	O(5)-S(2)-C(25)-F(6)	-71.0(6)
C(16)-C(17)-C(18)-C(14)	-0.2(6)	N(2)-N(1)-C(12)-C(11)	1.7(5)
Co(2)-C(17)-C(18)-C(14)	-59.6(3)	C(14)-N(1)-C(12)-C(11)	-178.6(4)
C(16)-C(17)-C(18)-Co(2)	59.4(4)	N(2)-N(1)-C(12)-Cu(1)	-169.6(3)
C(23)-C(19)-C(20)-C(21)	-0.7(7)	C(14)-N(1)-C(12)-Cu(1)	10.1(7)
Co(2)-C(19)-C(20)-C(21)	58.7(4)	N(3)-C(11)-C(12)-N(1)	-1.4(5)
C(23)-C(19)-C(20)-Co(2)	-59.4(4)	C(10)-C(11)-C(12)-N(1)	177.7(4)
C(19)-C(20)-C(21)-C(22)	0.8(7)	N(3)-C(11)-C(12)-Cu(1)	168.2(4)
Co(2)-C(20)-C(21)-C(22)	59.5(4)	C(10)-C(11)-C(12)-Cu(1)	-12.7(8)
C(19)-C(20)-C(21)-Co(2)	-58.8(4)	O(2)-S(1)-C(24)-F(1)	-61.2(6)
C(20)-C(21)-C(22)-C(23)	-0.5(6)	O(1)-S(1)-C(24)-F(1)	176.0(5)
Co(2)-C(21)-C(22)-C(23)	59.2(4)	O(3)-S(1)-C(24)-F(1)	58.0(5)
C(20)-C(21)-C(22)-Co(2)	-59.7(4)	O(2)-S(1)-C(24)-F(2)	175.8(5)
C(20)-C(19)-C(23)-C(22)	0.4(6)	O(1)-S(1)-C(24)-F(2)	53.0(6)
Co(2)-C(19)-C(23)-C(22)	-59.1(4)	O(3)-S(1)-C(24)-F(2)	-65.0(6)
C(20)-C(19)-C(23)-Co(2)	59.5(4)	O(2)-S(1)-C(24)-F(3)	59.3(6)
C(21)-C(22)-C(23)-C(19)	0.1(6)	O(1)-S(1)-C(24)-F(3)	-63.5(5)
Co(2)-C(22)-C(23)-C(19)	59.0(4)	O(3)-S(1)-C(24)-F(3)	178.5(5)
C(21)-C(22)-C(23)-Co(2)	-58.9(4)	N(2)-N(1)-C(12A)-C(11)	1.7(5)
O(4)-S(2)-C(25)-F(4)	171.9(9)	C(14)-N(1)-C(12A)-C(11)	-178.6(4)
O(6)-S(2)-C(25)-F(4)	-60.1(7)	N(2)-N(1)-C(12A)-Cu(1)	-169.6(3)
O(5)-S(2)-C(25)-F(4)	50.3(8)	C(14)-N(1)-C(12A)-Cu(1)	10.1(7)
O(4)-S(2)-C(25)-F(5)	-62.5(8)	N(3)-C(11)-C(12A)-N(1)	-1.4(5)
O(6)-S(2)-C(25)-F(5)	65.4(6)	C(10)-C(11)-C(12A)-N(1)	177.7(4)
O(5)-S(2)-C(25)-F(5)	175.9(5)	N(3)-C(11)-C(12A)-Cu(1)	168.2(4)
O(4)-S(2)-C(25)-F(6)	50.6(8)	C(10)-C(11)-C(12A)-Cu(1)	-12.7(8)
O(6)-S(2)-C(25)-F(6)	178.5(5)		

Symmetry transformations used to generate equivalent atoms: #1 -x+1,-y,-z+1

Triazolylidene complexe (13) – “chlorido (1,4-dicobaltoceniumyl-3-methyltriazol-ylidene) gold(I) hexafluoridophosphate triflate“

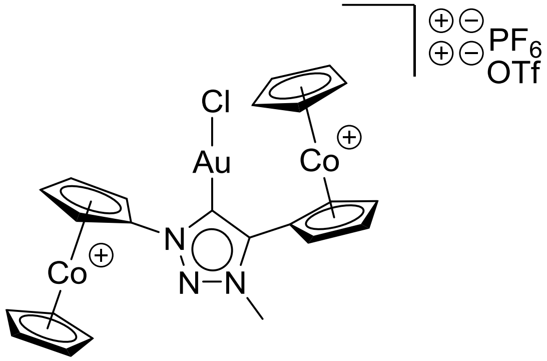

[983.76]

$C_{23}H_{21}N_3Co_2AuCl(PF_6)(CF_3SO_3)$

Analytical data:

IR (ATR [cm^{-1}]): 3099 ($\nu_{C–H}$), 1706, 1415 ($\nu_{C=C}$), 1362, 1255 (ν_{SO3}), 1224 (ν_{CF3}), 1149 (ν_{CF3}), 1027 (ν_{SO3}), 865 ($\nu_{P–F}$), 757, 635, 573 ($\nu_{P–F}$), 506, 480, 434.

^{1}H-NMR (300 MHz, C_3D_6O, [ppm]): δ 4.50 (s, 3H, CH_3 of triazolium), 6.12 (s, 5H, 4-Cc-Cp), 6.18 (pseudo-t, 2H, *J* = 2.1 Hz, C3/C4 of substituted 1-Cc-Cp), 6.19 (s, 5H, 1-Cc-Cp), 6.22 (pseudo-t, 2H, *J* = 1.8 Hz, C3/C4 of substituted 4-Cc-Cp), 6.66 (pseudo-t, 2H, *J* = 2.0 Hz, C2/C5 of substituted 4-Cc-Cp), 6.91 (pseudo-t, 2H, J = 2.3 Hz, C2/C5 of substituted 1-Cc-Cp).

^{13}C-NMR (75 MHz, C_3D_6O, [ppm]): δ 40.7 (CH_3 of triazolium), 81.0 (C3/C4 of substituted 4-Cc-Cp), 84.1 (C3/C4 of substituted 1-Cc-Cp), 86.0 (C2/C5 of substituted 4-Cc-Cp), 86.0 (C2/C5 of substituted 1-Cc-Cp), 88.4 (4-Cc-Cp), 88.7 (1-Cc-Cp), 92.2 (quart. carbon of substituted 4-Cc-Cp), 109.5 (quart. carbon of substituted 1-Cc-Cp), 120.0 (CF_3 of triflate), 124.3 (CF_3 of triflate), 141.0 (quart. carbon of triazolium), 162.2 (carbene carbon of triazolium).

MS (ESI pos, [m/z]): 837.93 ([$M(CF_3SO_3)]^+$).

Melting point [°C]: 161.

Spectra

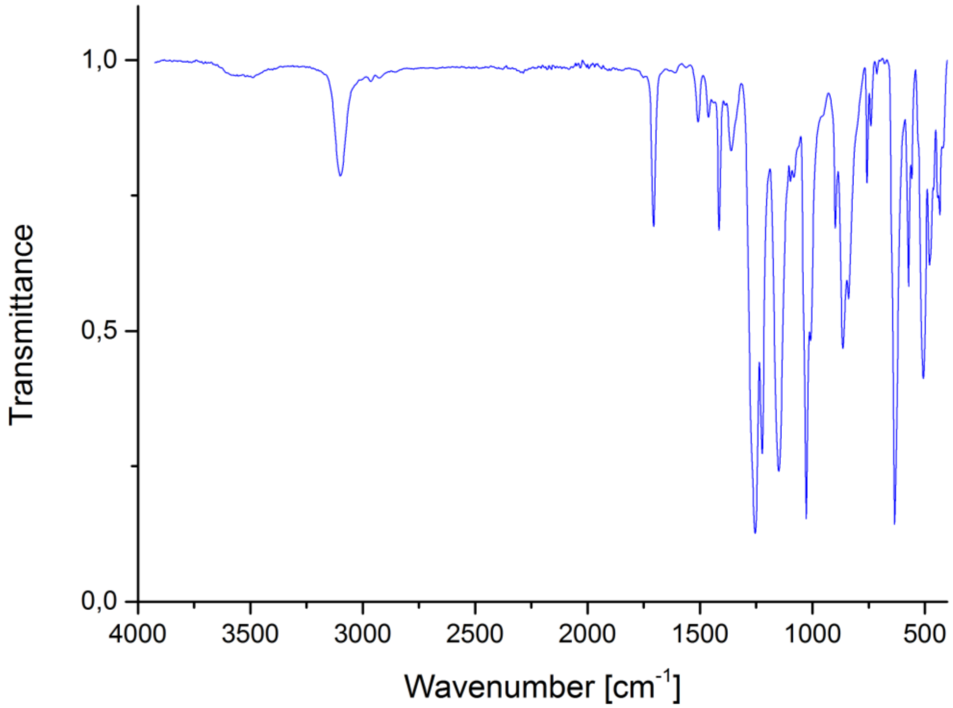

Figure S53. IR-spectrum (ATR, [cm^{-1}]) of “chlorido (1,4-dicobaltoceniumyl-3-methyltriazolylidene) gold(I) hexafluoridophosphate triflate“.

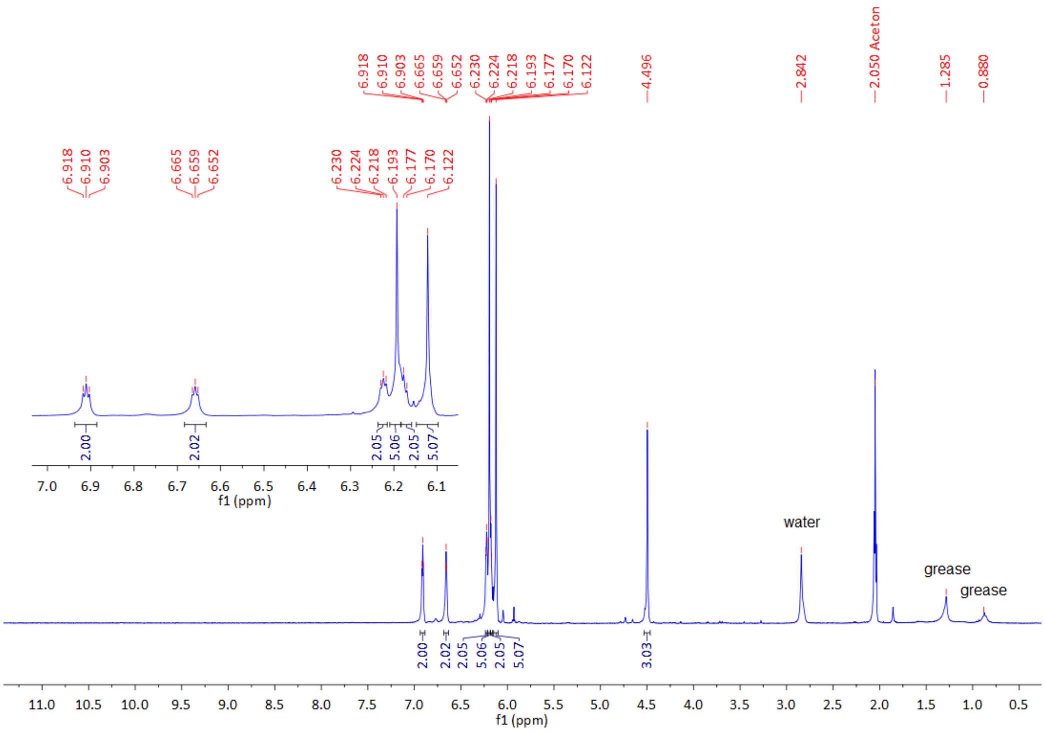

Figure S54. ^{1}H-NMR (300 MHz, C_3D_6O, [ppm]) of “chlorido (1,4-dicobaltoceniumyl-3-methyltriazolylidene) gold(I) hexafluoridophosphate triflate“.

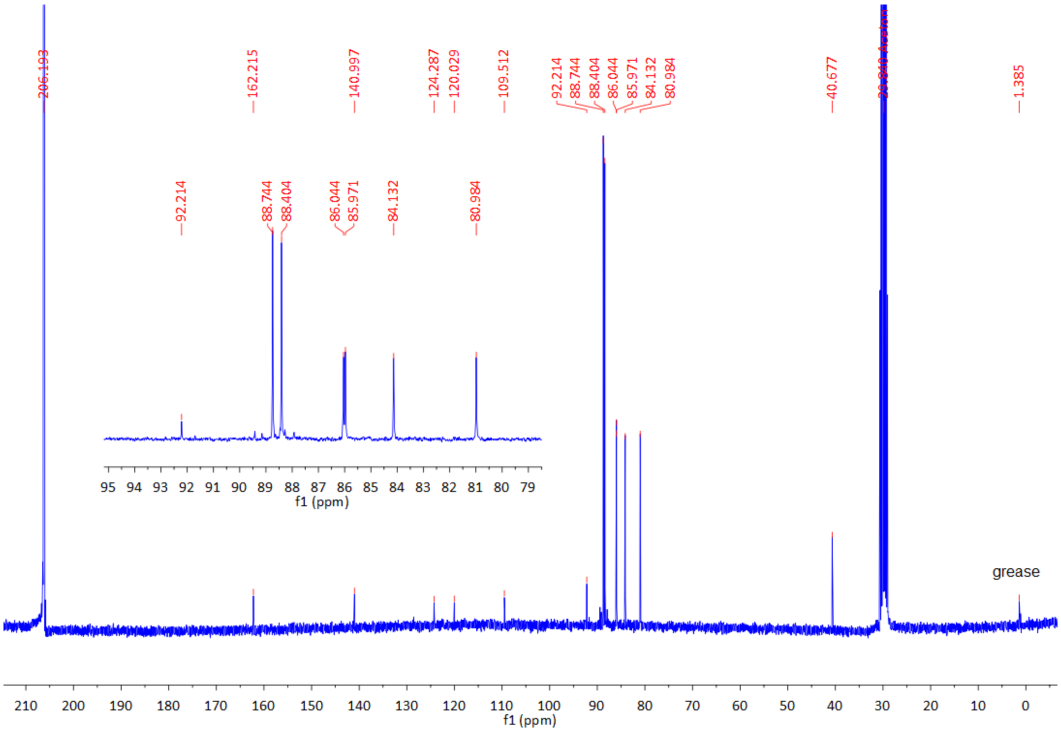

Figure S55. ^{13}C-NMR (75 MHz, C_3D_6O, [ppm]) of “chlorido (1,4-dicobaltoceniumyl-3-methyltriazolylidene) gold(I) hexafluoridophosphate triflate“.

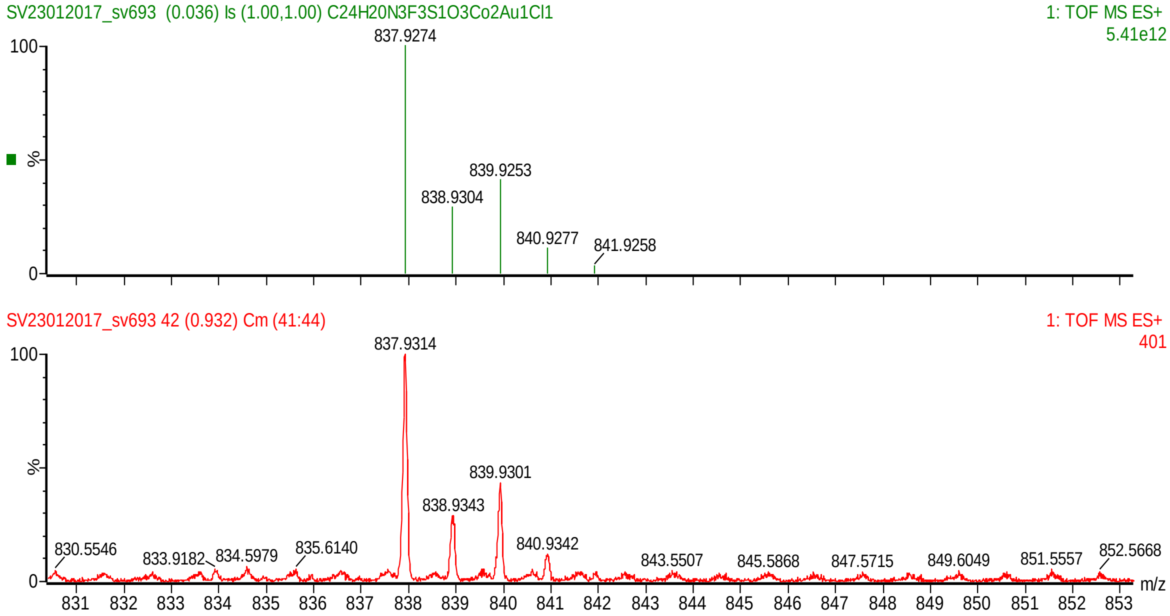

Figure S56. MS (ESI pos, [m/z]; *top*: simulated, *bottom*: experimental) of “chlorido (1,4-dicobaltoceniumyl-3-methyltriazolylidene) gold(I) hexafluoridophosphate triflate“.

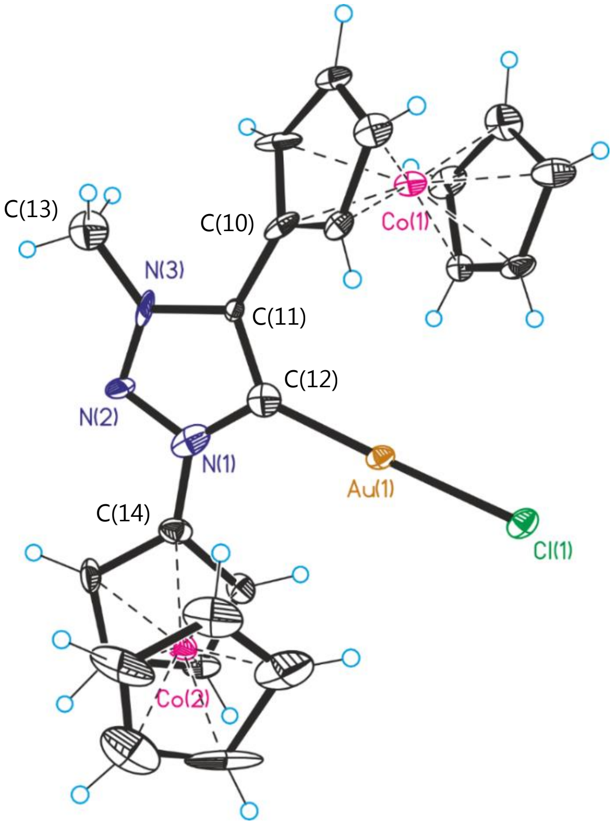

Figure S57. X-ray single crystal structure analysis of “chlorido (1,4-dicobaltoceniumyl-3-methyltriazolylidene) gold(I) hexafluoridophosphate triflate“ (counterions omitted for clarity).

X-Ray single crystal structure analysis data

Table S49. Crystal data and structure refinement for **13**.

Empirical formula	C25 H21 Au Cl Co2 F6 N3 O6 S2 x C3H6O	
Formula weight	1045.92	
Temperature	300(2) K	
Wavelength	0.71073 Å	
Crystal system	Triclinic	
Space group	P-1 (no. 2)	
Unit cell dimensions	a = 7.7345(4) Å	α = 90.009(2)°.
	b = 15.2167(7) Å	ß = 101.942(2)°.
	c = 15.7961(8) Å	γ = 104.396(2)°.
Volume	1759.14(15) $Å^3$	
Z	2	
Density (calculated)	1.975 Mg/m^3	
Absorption coefficient	5.366 mm^{-1}	
F(000)	1016	
Crystal size	0.190 x 0.180 x 0.090 mm^3	
Theta range for data collection	2.640 to 25.499°.	
Index ranges	-9<h<9, -18<k<18, -19<l<19	
Reflections collected	33895	
Independent reflections	6563 [R(int) = 0.0440]	
Completeness to theta = 25.242°	99.9 %	
Absorption correction	Semi-empirical from equivalents	
Max. and min. transmission	0.646 and 0.337	
Refinement method	Full-matrix least-squares on F^2	
Data / restraints / parameters	6563 / 0 / 454	
Goodness-of-fit on F^2	1.059	
Final R indices [I>2sigma(I)]	R1 = 0.0282, wR2 = 0.0603	
R indices (all data)	R1 = 0.0382, wR2 = 0.0633	
Extinction coefficient	n/a	
Largest diff. peak and hole	0.523 and -0.840 e.$Å^{-3}$	

Table S50. Atomic coordinates (x 10^4) and equivalent isotropic displacement parameters ($Å^2$x 10^3) for **13**. U(eq) is defined as one third of the trace of the orthogonalized U^{ij} tensor.

	x	y	z	U(eq)
Au(1)	7293(1)	5270(1)	24(1)	40(1)
Co(2)	9758(1)	5409(1)	3221(1)	40(1)
Co(1)	7668(1)	7764(1)	-950(1)	67(1)
Cl(1)	7620(2)	4278(1)	-981(1)	63(1)
N(1)	7180(4)	6068(2)	1752(2)	41(1)
N(2)	6815(5)	6734(3)	2174(2)	52(1)
N(3)	6467(5)	7278(3)	1563(3)	51(1)
C(1)	9037(14)	6942(5)	-1360(6)	121(3)
C(2)	8774(14)	7591(6)	-1964(5)	128(3)
C(3)	9624(13)	8450(5)	-1556(5)	113(3)
C(4)	10399(11)	8322(6)	-704(5)	102(2)
C(5)	10022(11)	7390(6)	-596(6)	108(3)
C(6)	5168(8)	7014(4)	-824(4)	78(2)
C(7)	4990(10)	7690(4)	-1427(4)	104(3)
C(8)	5860(9)	8537(4)	-997(4)	86(2)
C(9)	6614(7)	8405(3)	-135(4)	64(1)
C(10)	6160(6)	7445(3)	-19(3)	54(1)
C(11)	6588(5)	6983(3)	772(3)	42(1)
C(12)	7058(5)	6163(3)	879(3)	38(1)
C(13)	5868(9)	8074(4)	1811(4)	82(2)
C(14)	7570(5)	5333(3)	2241(3)	41(1)
C(15)	8614(5)	4750(3)	2048(3)	42(1)
C(16)	8658(6)	4112(3)	2706(3)	52(1)
C(17)	7654(6)	4309(4)	3293(3)	59(1)
C(18)	6974(6)	5068(3)	3024(3)	50(1)
C(19)	10941(8)	6730(4)	3606(4)	72(2)
C(20)	11889(9)	6422(5)	3098(4)	92(2)
C(21)	12504(7)	5692(6)	3489(5)	93(2)
C(22)	11857(7)	5575(4)	4263(4)	63(1)
C(23)	10900(6)	6210(4)	4319(3)	59(1)
S(1)	3023(2)	3256(1)	4565(1)	62(1)
S(2)	8672(2)	1078(1)	8250(1)	63(1)
O(1)	4346(8)	3343(4)	4071(4)	135(2)
O(2)	1200(6)	3130(3)	4095(3)	100(2)
O(3)	3474(6)	3886(3)	5295(3)	92(1)
O(4)	7125(7)	731(3)	8621(3)	102(2)
O(5)	10091(6)	635(3)	8510(3)	98(1)
O(6)	9236(6)	2036(3)	8265(3)	95(1)
C(24)	2997(9)	2186(5)	5078(5)	90(2)
C(25)	7836(10)	738(4)	7131(5)	83(2)
F(1)	1766(8)	2000(4)	5540(4)	158(2)
F(2)	4606(7)	2203(3)	5556(4)	153(2)

F(3)	2600(8)	1523(3)	4485(4)	158(2)
F(4)	9121(8)	1014(4)	6686(3)	142(2)
F(5)	6484(7)	1097(3)	6780(3)	134(2)
F(6)	7263(7)	-156(3)	7010(3)	133(2)
O(7)	2681(12)	-1575(8)	7087(6)	242(5)
C(26)	2555(9)	-1154(6)	6484(6)	104(3)
C(27)	2902(15)	-1410(10)	5683(9)	227(8)
C(28)	1938(16)	-348(8)	6497(11)	227(7)

Table S51. Bond lengths [Å] and angles [°] for **13**.

Au(1)-C(12)	1.982(4)	C(3)-C(4)	1.391(10)
Au(1)-Cl(1)	2.2836(12)	C(3)-H(3)	0.9300
Co(2)-C(20)	2.000(5)	C(4)-C(5)	1.394(10)
Co(2)-C(21)	2.012(5)	C(4)-H(4)	0.9300
Co(2)-C(14)	2.023(4)	C(5)-H(5)	0.9300
Co(2)-C(15)	2.024(4)	C(6)-C(10)	1.408(7)
Co(2)-C(22)	2.025(5)	C(6)-C(7)	1.416(7)
Co(2)-C(19)	2.026(5)	C(6)-H(6)	0.9300
Co(2)-C(23)	2.027(5)	C(7)-C(8)	1.398(9)
Co(2)-C(16)	2.038(5)	C(7)-H(7)	0.9300
Co(2)-C(18)	2.043(4)	C(8)-C(9)	1.402(8)
Co(2)-C(17)	2.046(5)	C(8)-H(8)	0.9300
Co(1)-C(5)	2.012(8)	C(9)-C(10)	1.436(6)
Co(1)-C(2)	2.012(7)	C(9)-H(9)	0.9300
Co(1)-C(1)	2.013(7)	C(10)-C(11)	1.452(6)
Co(1)-C(9)	2.026(6)	C(11)-C(12)	1.385(6)
Co(1)-C(4)	2.026(8)	C(13)-H(13A)	0.9600
Co(1)-C(3)	2.027(7)	C(13)-H(13B)	0.9600
Co(1)-C(7)	2.027(7)	C(13)-H(13C)	0.9600
Co(1)-C(8)	2.029(6)	C(14)-C(15)	1.411(6)
Co(1)-C(6)	2.036(6)	C(14)-C(18)	1.431(6)
Co(1)-C(10)	2.047(5)	C(15)-C(16)	1.422(6)
N(1)-N(2)	1.332(5)	C(15)-H(15)	0.9300
N(1)-C(12)	1.373(5)	C(16)-C(17)	1.402(7)
N(1)-C(14)	1.418(5)	C(16)-H(16)	0.9300
N(2)-N(3)	1.301(5)	C(17)-C(18)	1.414(7)
N(3)-C(11)	1.357(6)	C(17)-H(17)	0.9300
N(3)-C(13)	1.480(6)	C(18)-H(18)	0.9300
C(1)-C(5)	1.365(11)	C(19)-C(20)	1.347(9)
C(1)-C(2)	1.396(10)	C(19)-C(23)	1.377(7)
C(1)-H(1)	0.9300	C(19)-H(19)	0.9300
C(2)-C(3)	1.398(11)	C(20)-C(21)	1.408(10)
C(2)-H(2)	0.9300	C(20)-H(20)	0.9300

C(21)-C(22)	1.409(9)	C(24)-F(3)	1.310(8)
C(21)-H(21)	0.9300	C(24)-F(2)	1.310(7)
C(22)-C(23)	1.368(7)	C(25)-F(4)	1.320(8)
C(22)-H(22)	0.9300	C(25)-F(5)	1.320(7)
C(23)-H(23)	0.9300	C(25)-F(6)	1.323(7)
S(1)-O(1)	1.392(5)	O(7)-C(26)	1.150(9)
S(1)-O(2)	1.417(4)	C(26)-C(27)	1.421(13)
S(1)-O(3)	1.427(4)	C(26)-C(28)	1.423(12)
S(1)-C(24)	1.816(7)	C(27)-H(27A)	0.9600
S(2)-O(6)	1.414(4)	C(27)-H(27B)	0.9600
S(2)-O(5)	1.417(4)	C(27)-H(27C)	0.9600
S(2)-O(4)	1.424(4)	C(28)-H(28A)	0.9600
S(2)-C(25)	1.780(7)	C(28)-H(28B)	0.9600
C(24)-F(1)	1.294(8)	C(28)-H(28C)	0.9600

C(12)-Au(1)-Cl(1)	178.18(12)	C(15)-Co(2)-C(18)	69.20(18)
C(20)-Co(2)-C(21)	41.1(3)	C(22)-Co(2)-C(18)	135.6(2)
C(20)-Co(2)-C(14)	111.9(2)	C(19)-Co(2)-C(18)	113.9(2)
C(21)-Co(2)-C(14)	142.5(3)	C(23)-Co(2)-C(18)	110.9(2)
C(20)-Co(2)-C(15)	108.7(2)	C(16)-Co(2)-C(18)	68.5(2)
C(21)-Co(2)-C(15)	112.9(2)	C(20)-Co(2)-C(17)	175.6(3)
C(14)-Co(2)-C(15)	40.79(17)	C(21)-Co(2)-C(17)	136.4(3)
C(20)-Co(2)-C(22)	67.9(2)	C(14)-Co(2)-C(17)	68.17(17)
C(21)-Co(2)-C(22)	40.9(3)	C(15)-Co(2)-C(17)	68.33(18)
C(14)-Co(2)-C(22)	174.2(2)	C(22)-Co(2)-C(17)	112.5(2)
C(15)-Co(2)-C(22)	145.0(2)	C(19)-Co(2)-C(17)	145.2(3)
C(20)-Co(2)-C(19)	39.1(3)	C(23)-Co(2)-C(17)	116.5(2)
C(21)-Co(2)-C(19)	67.5(3)	C(16)-Co(2)-C(17)	40.2(2)
C(14)-Co(2)-C(19)	109.16(19)	C(18)-Co(2)-C(17)	40.5(2)
C(15)-Co(2)-C(19)	133.5(2)	C(5)-Co(1)-C(2)	67.4(4)
C(22)-Co(2)-C(19)	66.9(2)	C(5)-Co(1)-C(1)	39.7(3)
C(20)-Co(2)-C(23)	66.7(2)	C(2)-Co(1)-C(1)	40.6(3)
C(21)-Co(2)-C(23)	67.5(2)	C(5)-Co(1)-C(9)	123.8(3)
C(14)-Co(2)-C(23)	134.9(2)	C(2)-Co(1)-C(9)	158.0(3)
C(15)-Co(2)-C(23)	173.04(19)	C(1)-Co(1)-C(9)	159.5(3)
C(22)-Co(2)-C(23)	39.5(2)	C(5)-Co(1)-C(4)	40.4(3)
C(19)-Co(2)-C(23)	39.7(2)	C(2)-Co(1)-C(4)	67.7(4)
C(20)-Co(2)-C(16)	135.5(3)	C(1)-Co(1)-C(4)	67.6(4)
C(21)-Co(2)-C(16)	110.6(2)	C(9)-Co(1)-C(4)	107.5(3)
C(14)-Co(2)-C(16)	68.44(18)	C(5)-Co(1)-C(3)	67.6(3)
C(15)-Co(2)-C(16)	40.98(16)	C(2)-Co(1)-C(3)	40.5(3)
C(22)-Co(2)-C(16)	116.0(2)	C(1)-Co(1)-C(3)	68.0(3)
C(19)-Co(2)-C(16)	173.6(2)	C(9)-Co(1)-C(3)	121.9(3)
C(23)-Co(2)-C(16)	145.9(2)	C(4)-Co(1)-C(3)	40.1(3)
C(20)-Co(2)-C(18)	142.3(3)	C(5)-Co(1)-C(7)	160.7(4)
C(21)-Co(2)-C(18)	175.9(3)	C(2)-Co(1)-C(7)	107.1(4)
C(14)-Co(2)-C(18)	41.23(16)	C(1)-Co(1)-C(7)	124.2(4)

C(9)-Co(1)-C(7)	68.4(3)	C(2)-C(3)-H(3)	126.3
C(4)-Co(1)-C(7)	156.7(3)	Co(1)-C(3)-H(3)	126.2
C(3)-Co(1)-C(7)	121.1(3)	C(3)-C(4)-C(5)	107.6(7)
C(5)-Co(1)-C(8)	158.5(4)	C(3)-C(4)-Co(1)	70.0(5)
C(2)-Co(1)-C(8)	122.3(3)	C(5)-C(4)-Co(1)	69.3(5)
C(1)-Co(1)-C(8)	159.3(4)	C(3)-C(4)-H(4)	126.2
C(9)-Co(1)-C(8)	40.5(2)	C(5)-C(4)-H(4)	126.2
C(4)-Co(1)-C(8)	121.7(3)	Co(1)-C(4)-H(4)	126.1
C(3)-Co(1)-C(8)	106.3(3)	C(1)-C(5)-C(4)	109.0(7)
C(7)-Co(1)-C(8)	40.3(3)	C(1)-C(5)-Co(1)	70.2(5)
C(5)-Co(1)-C(6)	125.3(3)	C(4)-C(5)-Co(1)	70.3(4)
C(2)-Co(1)-C(6)	123.0(3)	C(1)-C(5)-H(5)	125.5
C(1)-Co(1)-C(6)	109.3(3)	C(4)-C(5)-H(5)	125.5
C(9)-Co(1)-C(6)	68.6(2)	Co(1)-C(5)-H(5)	125.5
C(4)-Co(1)-C(6)	161.1(3)	C(10)-C(6)-C(7)	108.4(5)
C(3)-Co(1)-C(6)	157.9(3)	C(10)-C(6)-Co(1)	70.3(3)
C(7)-Co(1)-C(6)	40.8(2)	C(7)-C(6)-Co(1)	69.3(4)
C(8)-Co(1)-C(6)	68.1(2)	C(10)-C(6)-H(6)	125.8
C(5)-Co(1)-C(10)	109.7(2)	C(7)-C(6)-H(6)	125.8
C(2)-Co(1)-C(10)	159.1(3)	Co(1)-C(6)-H(6)	126.2
C(1)-Co(1)-C(10)	123.8(2)	C(8)-C(7)-C(6)	107.9(5)
C(9)-Co(1)-C(10)	41.28(19)	C(8)-C(7)-Co(1)	69.9(4)
C(4)-Co(1)-C(10)	124.6(3)	C(6)-C(7)-Co(1)	69.9(4)
C(3)-Co(1)-C(10)	159.5(3)	C(8)-C(7)-H(7)	126.0
C(7)-Co(1)-C(10)	68.4(2)	C(6)-C(7)-H(7)	126.0
C(8)-Co(1)-C(10)	68.4(2)	Co(1)-C(7)-H(7)	125.7
C(6)-Co(1)-C(10)	40.3(2)	C(7)-C(8)-C(9)	108.9(5)
N(2)-N(1)-C(12)	115.2(3)	C(7)-C(8)-Co(1)	69.8(4)
N(2)-N(1)-C(14)	117.0(3)	C(9)-C(8)-Co(1)	69.6(3)
C(12)-N(1)-C(14)	127.8(4)	C(7)-C(8)-H(8)	125.6
N(3)-N(2)-N(1)	102.6(3)	C(9)-C(8)-H(8)	125.6
N(2)-N(3)-C(11)	113.8(4)	Co(1)-C(8)-H(8)	126.6
N(2)-N(3)-C(13)	116.3(4)	C(8)-C(9)-C(10)	107.6(5)
C(11)-N(3)-C(13)	129.7(4)	C(8)-C(9)-Co(1)	69.9(4)
C(5)-C(1)-C(2)	107.9(7)	C(10)-C(9)-Co(1)	70.2(3)
C(5)-C(1)-Co(1)	70.1(4)	C(8)-C(9)-H(9)	126.2
C(2)-C(1)-Co(1)	69.7(5)	C(10)-C(9)-H(9)	126.2
C(5)-C(1)-H(1)	126.0	Co(1)-C(9)-H(9)	125.3
C(2)-C(1)-H(1)	126.0	C(6)-C(10)-C(9)	107.1(4)
Co(1)-C(1)-H(1)	125.7	C(6)-C(10)-C(11)	125.1(4)
C(1)-C(2)-C(3)	108.0(8)	C(9)-C(10)-C(11)	127.8(5)
C(1)-C(2)-Co(1)	69.7(4)	C(6)-C(10)-Co(1)	69.4(3)
C(3)-C(2)-Co(1)	70.3(4)	C(9)-C(10)-Co(1)	68.6(3)
C(1)-C(2)-H(2)	126.0	C(11)-C(10)-Co(1)	128.0(3)
C(3)-C(2)-H(2)	126.0	N(3)-C(11)-C(12)	106.8(4)
Co(1)-C(2)-H(2)	125.5	N(3)-C(11)-C(10)	123.9(4)
C(4)-C(3)-C(2)	107.4(6)	C(12)-C(11)-C(10)	129.2(4)
C(4)-C(3)-Co(1)	69.9(4)	N(1)-C(12)-C(11)	101.7(4)
C(2)-C(3)-Co(1)	69.2(4)	N(1)-C(12)-Au(1)	127.0(3)
C(4)-C(3)-H(3)	126.3	C(11)-C(12)-Au(1)	131.2(3)

N(3)-C(13)-H(13A)	109.5	C(22)-C(21)-Co(2)	70.1(3)
N(3)-C(13)-H(13B)	109.5	C(20)-C(21)-H(21)	127.1
H(13A)-C(13)-H(13B)	109.5	C(22)-C(21)-H(21)	127.1
N(3)-C(13)-H(13C)	109.5	Co(2)-C(21)-H(21)	125.4
H(13A)-C(13)-H(13C)	109.5	C(23)-C(22)-C(21)	107.8(5)
H(13B)-C(13)-H(13C)	109.5	C(23)-C(22)-Co(2)	70.3(3)
C(15)-C(14)-N(1)	126.2(4)	C(21)-C(22)-Co(2)	69.0(3)
C(15)-C(14)-C(18)	108.7(4)	C(23)-C(22)-H(22)	126.1
N(1)-C(14)-C(18)	125.1(4)	C(21)-C(22)-H(22)	126.1
C(15)-C(14)-Co(2)	69.7(2)	Co(2)-C(22)-H(22)	126.1
N(1)-C(14)-Co(2)	125.7(3)	C(22)-C(23)-C(19)	108.8(5)
C(18)-C(14)-Co(2)	70.1(2)	C(22)-C(23)-Co(2)	70.2(3)
C(14)-C(15)-C(16)	107.5(4)	C(19)-C(23)-Co(2)	70.1(3)
C(14)-C(15)-Co(2)	69.5(2)	C(22)-C(23)-H(23)	125.6
C(16)-C(15)-Co(2)	70.0(2)	C(19)-C(23)-H(23)	125.6
C(14)-C(15)-H(15)	126.3	Co(2)-C(23)-H(23)	125.7
C(16)-C(15)-H(15)	126.3	O(1)-S(1)-O(2)	116.0(4)
Co(2)-C(15)-H(15)	125.7	O(1)-S(1)-O(3)	115.6(3)
C(17)-C(16)-C(15)	108.1(4)	O(2)-S(1)-O(3)	113.0(3)
C(17)-C(16)-Co(2)	70.2(3)	O(1)-S(1)-C(24)	104.4(4)
C(15)-C(16)-Co(2)	69.0(3)	O(2)-S(1)-C(24)	103.5(3)
C(17)-C(16)-H(16)	126.0	O(3)-S(1)-C(24)	102.0(3)
C(15)-C(16)-H(16)	126.0	O(6)-S(2)-O(5)	115.5(3)
Co(2)-C(16)-H(16)	126.4	O(6)-S(2)-O(4)	114.4(3)
C(16)-C(17)-C(18)	109.3(4)	O(5)-S(2)-O(4)	113.5(3)
C(16)-C(17)-Co(2)	69.6(3)	O(6)-S(2)-C(25)	104.3(3)
C(18)-C(17)-Co(2)	69.6(3)	O(5)-S(2)-C(25)	103.7(3)
C(16)-C(17)-H(17)	125.4	O(4)-S(2)-C(25)	103.4(3)
C(18)-C(17)-H(17)	125.4	F(1)-C(24)-F(3)	107.3(7)
Co(2)-C(17)-H(17)	126.9	F(1)-C(24)-F(2)	110.2(7)
C(17)-C(18)-C(14)	106.5(4)	F(3)-C(24)-F(2)	107.4(6)
C(17)-C(18)-Co(2)	69.9(3)	F(1)-C(24)-S(1)	111.1(5)
C(14)-C(18)-Co(2)	68.6(2)	F(3)-C(24)-S(1)	109.9(6)
C(17)-C(18)-H(18)	126.8	F(2)-C(24)-S(1)	110.8(5)
C(14)-C(18)-H(18)	126.8	F(4)-C(25)-F(5)	106.1(6)
Co(2)-C(18)-H(18)	126.3	F(4)-C(25)-F(6)	107.3(6)
C(20)-C(19)-C(23)	108.6(6)	F(5)-C(25)-F(6)	108.7(6)
C(20)-C(19)-Co(2)	69.4(3)	F(4)-C(25)-S(2)	111.2(5)
C(23)-C(19)-Co(2)	70.2(3)	F(5)-C(25)-S(2)	111.8(5)
C(20)-C(19)-H(19)	125.7	F(6)-C(25)-S(2)	111.5(5)
C(23)-C(19)-H(19)	125.7	O(7)-C(26)-C(27)	123.6(12)
Co(2)-C(19)-H(19)	126.3	O(7)-C(26)-C(28)	120.3(13)
C(19)-C(20)-C(21)	108.9(6)	C(27)-C(26)-C(28)	116.0(11)
C(19)-C(20)-Co(2)	71.5(3)	C(26)-C(27)-H(27A)	109.5
C(21)-C(20)-Co(2)	69.9(3)	C(26)-C(27)-H(27B)	109.5
C(19)-C(20)-H(20)	125.5	H(27A)-C(27)-H(27B)	109.5
C(21)-C(20)-H(20)	125.5	C(26)-C(27)-H(27C)	109.5
Co(2)-C(20)-H(20)	124.7	H(27A)-C(27)-H(27C)	109.5
C(20)-C(21)-C(22)	105.9(5)	H(27B)-C(27)-H(27C)	109.5
C(20)-C(21)-Co(2)	69.0(3)	C(26)-C(28)-H(28A)	109.5

C(26)-C(28)-H(28B)	109.5	H(28A)-C(28)-H(28C)	109.5
H(28A)-C(28)-H(28B)	109.5	H(28B)-C(28)-H(28C)	109.5
C(26)-C(28)-H(28C)	109.5		

Table S52. Anisotropic displacement parameters ($Å^2$x 10^3) for **13**. The anisotropic displacement factor exponent takes the form: $-2p^2 [h^2 a^{*2}U^{11} + ... + 2 h k a^* b^* U^{12}]$.

	U^{11}	U^{22}	U^{33}	U^{23}	U^{13}	U^{12}
Au(1)	43(1)	40(1)	33(1)	2(1)	6(1)	7(1)
Co(2)	33(1)	50(1)	30(1)	5(1)	4(1)	3(1)
Co(1)	107(1)	42(1)	47(1)	10(1)	11(1)	16(1)
Cl(1)	58(1)	65(1)	61(1)	-21(1)	23(1)	0(1)
N(1)	33(2)	49(2)	40(2)	-3(2)	5(1)	12(2)
N(2)	46(2)	62(3)	48(2)	-7(2)	6(2)	19(2)
N(3)	43(2)	49(2)	62(3)	-10(2)	8(2)	15(2)
C(1)	226(10)	62(4)	115(6)	23(5)	112(7)	49(6)
C(2)	230(10)	105(6)	63(4)	10(4)	68(6)	38(7)
C(3)	196(9)	66(4)	107(6)	40(4)	93(6)	37(5)
C(4)	116(6)	96(6)	98(6)	-2(4)	60(5)	8(4)
C(5)	131(6)	124(7)	111(6)	60(6)	83(5)	64(6)
C(6)	89(4)	52(3)	65(4)	10(3)	-27(3)	2(3)
C(7)	120(6)	75(4)	80(4)	28(4)	-41(4)	7(4)
C(8)	100(5)	55(3)	95(5)	28(3)	-6(4)	25(3)
C(9)	63(3)	43(3)	82(4)	8(3)	6(3)	13(2)
C(10)	50(3)	42(3)	63(3)	7(2)	-3(2)	11(2)
C(11)	32(2)	42(2)	49(3)	3(2)	0(2)	10(2)
C(12)	30(2)	46(2)	35(2)	5(2)	1(2)	6(2)
C(13)	92(4)	72(4)	96(5)	-8(3)	27(4)	41(4)
C(14)	33(2)	51(3)	33(2)	6(2)	5(2)	3(2)
C(15)	42(2)	48(2)	31(2)	3(2)	3(2)	9(2)
C(16)	54(3)	46(3)	43(3)	5(2)	-2(2)	3(2)
C(17)	50(3)	70(3)	42(3)	19(2)	4(2)	-7(2)
C(18)	35(2)	72(3)	38(2)	-1(2)	10(2)	2(2)
C(19)	73(4)	43(3)	77(4)	4(3)	-19(3)	1(3)
C(20)	74(4)	109(6)	55(4)	-3(4)	14(3)	-47(4)
C(21)	28(3)	121(6)	119(6)	-63(5)	9(3)	4(3)
C(22)	54(3)	59(3)	63(3)	0(3)	-17(3)	12(3)
C(23)	49(3)	77(4)	43(3)	-12(3)	3(2)	7(3)
S(1)	58(1)	68(1)	62(1)	2(1)	16(1)	15(1)
S(2)	63(1)	47(1)	75(1)	3(1)	6(1)	12(1)
O(1)	137(5)	143(5)	141(5)	5(4)	100(4)	9(4)
O(2)	82(3)	122(4)	89(3)	5(3)	-14(2)	39(3)
O(3)	103(3)	82(3)	89(3)	-25(2)	7(2)	31(3)

O(4)	107(3)	89(3)	123(4)	26(3)	56(3)	22(3)
O(5)	81(3)	88(3)	115(4)	-4(3)	-19(2)	37(2)
O(6)	109(3)	50(2)	111(4)	-5(2)	7(3)	4(2)
C(24)	72(4)	80(5)	118(6)	-1(4)	17(4)	20(4)
C(25)	91(5)	61(4)	91(5)	-2(3)	4(4)	22(3)
F(1)	160(5)	147(5)	189(5)	80(4)	91(4)	38(4)
F(2)	133(4)	137(4)	182(5)	18(4)	-33(4)	74(3)
F(3)	158(5)	73(3)	231(7)	-40(4)	24(4)	22(3)
F(4)	194(5)	147(4)	112(4)	14(3)	77(4)	55(4)
F(5)	134(4)	132(4)	122(4)	10(3)	-37(3)	59(3)
F(6)	170(4)	74(3)	125(4)	-33(2)	-22(3)	19(3)
O(7)	168(7)	323(13)	196(8)	119(9)	-34(6)	49(8)
C(26)	58(4)	122(7)	113(6)	37(5)	-11(4)	10(4)
C(27)	136(9)	283(18)	252(16)	-154(14)	66(10)	14(10)
C(28)	160(11)	127(9)	410(20)	-37(12)	79(13)	48(9)

Table S53. Hydrogen coordinates (x 10^4) and isotropic displacement parameters (Å^{2}x 10^3) for **13**.

	x	y	z	U(eq)
H(1)	8616	6315	-1460	145
H(2)	8144	7472	-2538	154
H(3)	9664	9005	-1808	136
H(4)	11052	8777	-282	122
H(5)	10383	7117	-85	129
H(6)	4708	6390	-940	94
H(7)	4398	7589	-2008	125
H(8)	5928	9097	-1243	104
H(9)	7287	8857	286	76
H(13A)	5632	8014	2384	123
H(13B)	4773	8107	1409	123
H(13C)	6810	8618	1800	123
H(15)	9172	4777	1577	50
H(16)	9248	3647	2740	62
H(17)	7466	3991	3783	70
H(18)	6276	5341	3301	60
H(19)	10404	7212	3494	87
H(20)	12103	6654	2574	110
H(21)	13195	5357	3280	112
H(22)	12047	5144	4665	76
H(23)	10318	6279	4765	71
H(27A)	3419	-1924	5756	341
H(27B)	3744	-912	5495	341
H(27C)	1779	-1568	5256	341

H(28A)	633	-507	6425	340
H(28B)	2278	18	6034	340
H(28C)	2487	-13	7042	340

Table S54. Torsion angles [°] for **13**.

C(12)-N(1)-N(2)-N(3)	-0.6(5)	C(13)-N(3)-C(11)-C(12)	-174.5(5)
C(14)-N(1)-N(2)-N(3)	-178.1(3)	N(2)-N(3)-C(11)-C(10)	176.3(4)
N(1)-N(2)-N(3)-C(11)	0.3(5)	C(13)-N(3)-C(11)-C(10)	1.6(7)
N(1)-N(2)-N(3)-C(13)	175.7(4)	C(6)-C(10)-C(11)-N(3)	-137.4(6)
C(5)-C(1)-C(2)-C(3)	0.2(10)	C(9)-C(10)-C(11)-N(3)	41.4(7)
Co(1)-C(1)-C(2)-C(3)	60.2(6)	Co(1)-C(10)-C(11)-N(3)	132.4(4)
C(5)-C(1)-C(2)-Co(1)	-60.0(6)	C(6)-C(10)-C(11)-C(12)	37.8(8)
C(1)-C(2)-C(3)-C(4)	-0.1(10)	C(9)-C(10)-C(11)-C(12)	-143.4(5)
Co(1)-C(2)-C(3)-C(4)	59.7(6)	Co(1)-C(10)-C(11)-C(12)	-52.3(6)
C(1)-C(2)-C(3)-Co(1)	-59.8(6)	N(2)-N(1)-C(12)-C(11)	0.7(4)
C(2)-C(3)-C(4)-C(5)	0.0(9)	C(14)-N(1)-C(12)-C(11)	177.9(4)
Co(1)-C(3)-C(4)-C(5)	59.3(5)	N(2)-N(1)-C(12)-Au(1)	-174.4(3)
C(2)-C(3)-C(4)-Co(1)	-59.2(6)	C(14)-N(1)-C(12)-Au(1)	2.7(6)
C(2)-C(1)-C(5)-C(4)	-0.1(10)	N(3)-C(11)-C(12)-N(1)	-0.5(4)
Co(1)-C(1)-C(5)-C(4)	-59.9(5)	C(10)-C(11)-C(12)-N(1)	-176.4(4)
C(2)-C(1)-C(5)-Co(1)	59.7(6)	N(3)-C(11)-C(12)-Au(1)	174.4(3)
C(3)-C(4)-C(5)-C(1)	0.1(9)	C(10)-C(11)-C(12)-Au(1)	-1.5(7)
Co(1)-C(4)-C(5)-C(1)	59.8(6)	N(2)-N(1)-C(14)-C(15)	-154.9(4)
C(3)-C(4)-C(5)-Co(1)	-59.7(5)	C(12)-N(1)-C(14)-C(15)	28.0(6)
C(10)-C(6)-C(7)-C(8)	-0.3(9)	N(2)-N(1)-C(14)-C(18)	24.4(6)
Co(1)-C(6)-C(7)-C(8)	-59.8(6)	C(12)-N(1)-C(14)-C(18)	-152.7(4)
C(10)-C(6)-C(7)-Co(1)	59.6(5)	N(2)-N(1)-C(14)-Co(2)	-65.1(4)
C(6)-C(7)-C(8)-C(9)	1.1(9)	C(12)-N(1)-C(14)-Co(2)	117.7(4)
Co(1)-C(7)-C(8)-C(9)	-58.8(5)	N(1)-C(14)-C(15)-C(16)	180.0(4)
C(6)-C(7)-C(8)-Co(1)	59.8(5)	C(18)-C(14)-C(15)-C(16)	0.6(5)
C(7)-C(8)-C(9)-C(10)	-1.4(8)	Co(2)-C(14)-C(15)-C(16)	60.0(3)
Co(1)-C(8)-C(9)-C(10)	-60.3(4)	N(1)-C(14)-C(15)-Co(2)	119.9(4)
C(7)-C(8)-C(9)-Co(1)	58.9(6)	C(18)-C(14)-C(15)-Co(2)	-59.4(3)
C(7)-C(6)-C(10)-C(9)	-0.6(7)	C(14)-C(15)-C(16)-C(17)	-0.2(5)
Co(1)-C(6)-C(10)-C(9)	58.4(4)	Co(2)-C(15)-C(16)-C(17)	59.5(3)
C(7)-C(6)-C(10)-C(11)	178.4(6)	C(14)-C(15)-C(16)-Co(2)	-59.7(3)
Co(1)-C(6)-C(10)-C(11)	-122.6(5)	C(15)-C(16)-C(17)-C(18)	-0.3(5)
C(7)-C(6)-C(10)-Co(1)	-59.0(5)	Co(2)-C(16)-C(17)-C(18)	58.5(3)
C(8)-C(9)-C(10)-C(6)	1.3(7)	C(15)-C(16)-C(17)-Co(2)	-58.8(3)
Co(1)-C(9)-C(10)-C(6)	-58.9(4)	C(16)-C(17)-C(18)-C(14)	0.6(5)
C(8)-C(9)-C(10)-C(11)	-177.7(5)	Co(2)-C(17)-C(18)-C(14)	59.1(3)
Co(1)-C(9)-C(10)-C(11)	122.1(5)	C(16)-C(17)-C(18)-Co(2)	-58.5(3)
C(8)-C(9)-C(10)-Co(1)	60.1(4)	C(15)-C(14)-C(18)-C(17)	-0.8(5)
N(2)-N(3)-C(11)-C(12)	0.1(5)	N(1)-C(14)-C(18)-C(17)	179.8(4)

Co(2)-C(14)-C(18)-C(17)	-59.9(3)	O(1)-S(1)-C(24)-F(1)	-177.7(6)
C(15)-C(14)-C(18)-Co(2)	59.2(3)	O(2)-S(1)-C(24)-F(1)	-55.9(6)
N(1)-C(14)-C(18)-Co(2)	-120.2(4)	O(3)-S(1)-C(24)-F(1)	61.5(6)
C(23)-C(19)-C(20)-C(21)	-0.7(6)	O(1)-S(1)-C(24)-F(3)	-59.1(6)
Co(2)-C(19)-C(20)-C(21)	-60.1(4)	O(2)-S(1)-C(24)-F(3)	62.6(6)
C(23)-C(19)-C(20)-Co(2)	59.4(4)	O(3)-S(1)-C(24)-F(3)	-179.9(5)
C(19)-C(20)-C(21)-C(22)	0.3(6)	O(1)-S(1)-C(24)-F(2)	59.4(7)
Co(2)-C(20)-C(21)-C(22)	-60.8(4)	O(2)-S(1)-C(24)-F(2)	-178.9(6)
C(19)-C(20)-C(21)-Co(2)	61.1(4)	O(3)-S(1)-C(24)-F(2)	-61.4(6)
C(20)-C(21)-C(22)-C(23)	0.2(6)	O(6)-S(2)-C(25)-F(4)	59.8(5)
Co(2)-C(21)-C(22)-C(23)	-59.9(4)	O(5)-S(2)-C(25)-F(4)	-61.5(5)
C(20)-C(21)-C(22)-Co(2)	60.1(4)	O(4)-S(2)-C(25)-F(4)	179.8(5)
C(21)-C(22)-C(23)-C(19)	-0.6(6)	O(6)-S(2)-C(25)-F(5)	-58.6(6)
Co(2)-C(22)-C(23)-C(19)	-59.7(4)	O(5)-S(2)-C(25)-F(5)	-179.9(5)
C(21)-C(22)-C(23)-Co(2)	59.1(4)	O(4)-S(2)-C(25)-F(5)	61.4(6)
C(20)-C(19)-C(23)-C(22)	0.8(6)	O(6)-S(2)-C(25)-F(6)	179.5(5)
Co(2)-C(19)-C(23)-C(22)	59.8(4)	O(5)-S(2)-C(25)-F(6)	58.2(6)
C(20)-C(19)-C(23)-Co(2)	-58.9(4)	O(4)-S(2)-C(25)-F(6)	-60.5(6)

Computational Section

All calculations were performed with the program suite Turbomole.[1] The only exception is the calculation of Tolman Electronic Parameters, for which Gaussian09 was used.[2] Structures and molecular electrostatic potentials (MEPs) were visualized with PyMol.[3]

Tolman Electronic Parameters (TEP) are determined in experiment to characterize the electronic properties of carbenes as ligands in organic complexes. Quantum chemical TEP calculations of these complexes, typically depend amongst other on the employed exchange-correlation functional. Thus, transferability of the results may not be straight forward. However recently, a parametrization scheme has been published by Mathew and Sureh allowing for a quantum chemical calculation of the Tolman Electronic Parameter of the ligand alone, while requiring only the molecular electrostatic potential at the carbene (V_c).[4] They examined 28 common NHCs and showed a linear correlation between the experimentally obtained TEP value and the molecular electrostatic potential V_C at the carbene carbon atom to be

$$\mathrm{TEP} = 0.4335\mathrm{V_C} + 6072.9 \qquad \text{(Eq.1)}$$

when V_C is calculated with B3LYP/6-311++G**.

For sake of comparison, calculation of TEPs in this study were performed with Gausian09[2] using the density functionals B3LYP,[5] in combination with the Pople type basis set 6-311++G(d,p) basis set as implemented in Gaussian09.[6,7] For the calculation of TEP, the B3LYP/6-311++G(d,p) single point were performed on the BP86/def2-TZVP optimized structures.

To calculate pK_a values in solution, the solvent was modeled implicitly by a COSMO approach.[8] Due to the solubility of **8-10** in water, a dielectric constant of ε = 80 was chosen.

pK_a values were obtained from the Gibbs free energy of the solvation process $\Delta G^{\mathrm{Rxn}}{}_{\mathrm{(solv)}}$ according to

$$\mathrm{p}K_a = \frac{\Delta G^{\mathrm{Rxn}}{}_{\mathrm{(solv)}}}{\mathrm{RTln(10)}} \qquad (1)$$

R denotes here the universal gas constant and T the temperature. While $\Delta G^{\mathrm{Rxn}}{}_{\mathrm{(solv)}}$ is difficult to calculate directly, it can conveniently be obtained obtain *via* the thermodynamic cycle depicted in Scheme S1: By calculation of the gas phase reaction free energy $\Delta G^{\mathrm{Rxn}}{}_{\mathrm{(g)}}$ and the free energies of solvation for each species, $\Delta G_{\mathrm{(solv)}}$ (HA), $\Delta G_{\mathrm{(solv)}}$ (H^+), and $\Delta G_{\mathrm{(solv)}}$ (A^-), $\Delta G^{\mathrm{Rxn}}{}_{\mathrm{(solv)}}$ was retrieved. Here, $\Delta G_{\mathrm{(solv)}}$ (HA) is $\Delta G_{\mathrm{(solv)}}$ of **8-10**, whereas $\Delta G_{\mathrm{(solv)}}$ (A^-) corresponds to $\Delta G_{\mathrm{(solv)}}$ of the deprotonated species **8'-10'**. The literature value of -259.80 kcal/mol was used for the free energy of solvation, $\Delta G_{\mathrm{(solv)}}$ (H^+),[9] whereas the phase free energy of H^+, $G_{\mathrm{(g)}}$ (H^+) = -6.29 kcal/mol, was obtained from the Sackur-Tetrode equation and translational energy at 298 K.[10]

pK_a values were calculated for both density functionals (BP86 and PBE0) in combination with and without dispersion corrections.

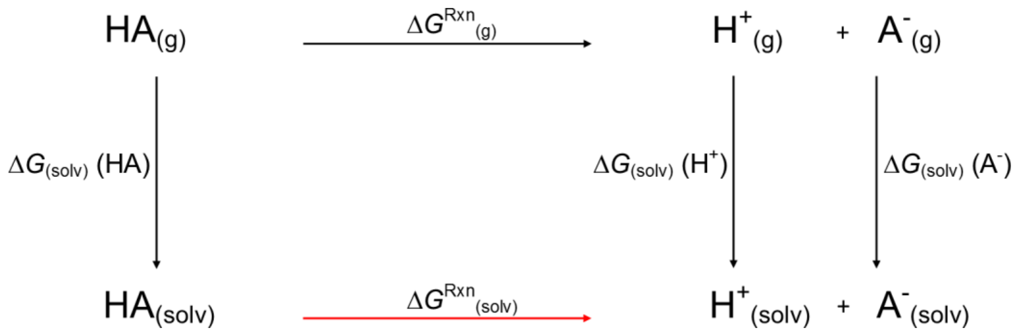

Scheme S1. Thermodynamic cycle used for the calculation of p*K*a values. While $\Delta G^{Rxn}{}_{(solv)}$ (depicted by the red arrow) is the property of interest, it has to be calculated via the gas phase reaction free energy and the solvation free energies of each species.

*Structural Parameters and Relative Energies of **8-10***

The X-ray crystal structures of the three triazolium salts **8-10** show variably twisted ferrocenyl and cobaltoceniumyl ligands. While the two substituents point in different directions (*anti* conformation) in **8** and **10**, the point in the same direction in **9** (*syn* orientation). These different orientations are likely to arise due to packing effects and their low energy conformations may differ in the gas phase. Therefore, all four possible conformations are energy minimized, exemplary shown for **8** in Figure S58. In all calculations the *anti* conformers, where the two substituents point in opposite directions, are ca. 4 kJ/mol more stable (see Table S55) but structural parameters such as bond lengths are very similar (see Table S56).

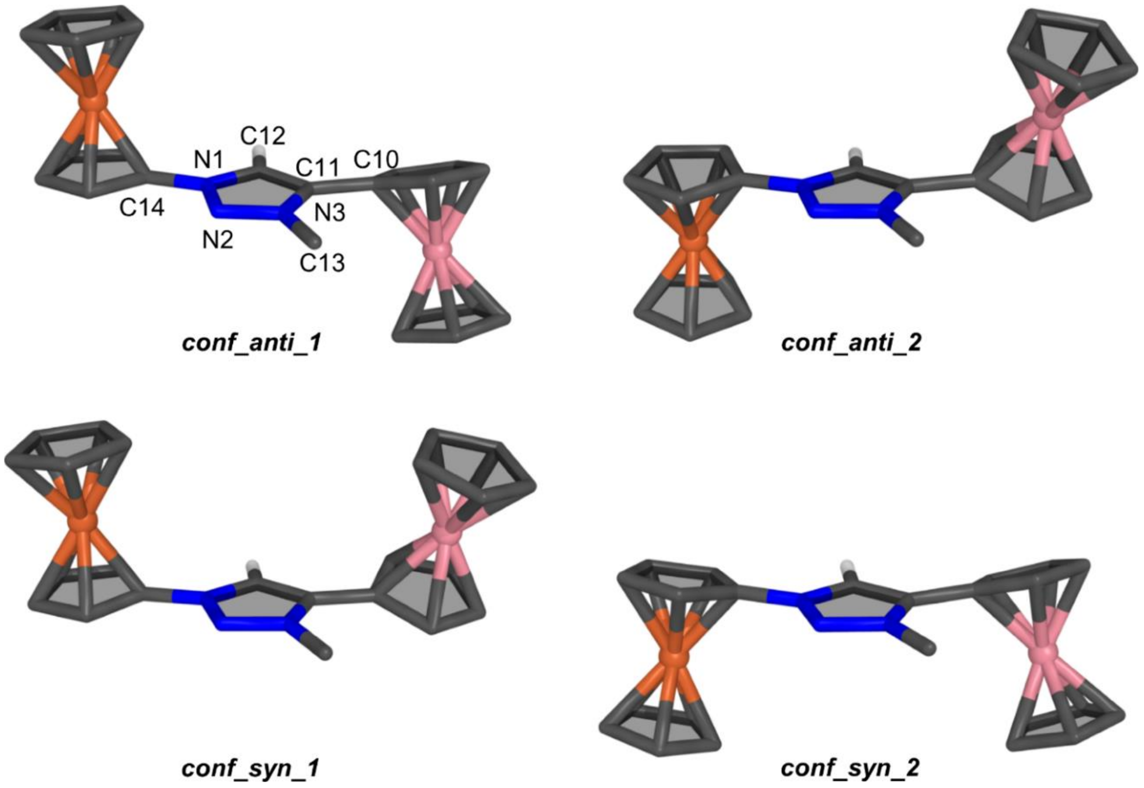

Figure S58. Four conformations of **8**.

Table S55. Relative energies in kJ/mol for the four conformers of **8**.

**8**	**BP86/def2-SV(P)**	**BP86/def2-TZVP**	**BP86 /def2-TZVP/BJ**	**PBE0/def2-TZVP**	**PBE0/def2-TZVP/BJ**
*conf_anti_1**	0.04	0.09	0.00	0.88	0.00
conf_anti_2	0.00	0.00	0.18	0.00	1.00
conf_syn_1	3.81	4.32	4.47	4.47	3.46
conf_syn_2	5.56	4.26	4.12	4.13	3.98

Table S56. Structural parameters of **8-10**. Bond length are given in Å.

	**N1-N2**	**N2-N3**	**N3-C11**	**C11-C12**	**N1-C12**	**N1-C14**	**C10-C11**
**8**							
Conf_anti_1							
BP86/def2-TZVP	1.333	1.335	1.378	1.389	1.366	1.414	1.455
BP86/def2-TZVP/BJ	1.333	1.336	1.376	1.386	1.368	1.410	1.451
PBE0/def2-TZVP	1.304	1.310	1.361	1.375	1.352	1.409	1.455
Conf_anti_2							
BP86/def2-TZVP	1.333	1.334	1.378	1.389	1.366	1.414	1.456
BP86/def2-TZVP/BJ	1.332	1.336	1.376	1.386	1.368	1.410	1.451
PBE0/def2-TZVP	1.304	1.308	1.361	1.374	1.352	1.410	1.457
PBE0/def2-TZVP/BJ	1.303	1.311	1.361	1.373	1.353	1.407	1.452
Conf_syn_1							
BP86/def2-TZVP	1.333	1.333	1.378	1.389	1.366	1.415	1.456
BP86/def2-TZVP/BJ	1.333	1.335	1.376	1.387	1.367	1.410	1.451
Conf_syn_2							
BP86/def2-TZVP	1.333	1.334	1.378	1.389	1.366	1.415	1.456
BP86/def2-TZVP/BJ	1.333	1.334	1.375	1.387	1.367	1.411	1.451
Exp.	1.326(2)	1.320(2)	1.367(2)	1.374(3)	1.350(2)	1.425(2)	1.455(3)

	**N1-N2**	**N2-N3**	**N3-C11**	**C11-C12**	**N1-C12**	**N1-C14**	**C10-C11**
**9**							
Conf_anti_1							
BP86/def2-TZVP	1.333	1.331	1.386	1.395	1.367	1.415	1.439
BP86/def2-TZVP/BJ	1.333	1.329	1.385	1.394	1.367	1.411	1.435
Exp.	1.323(3)	1.322(3)	1.366(3)	1.369(4)	1.353(3)	1.417(3)	1.456(4)

	N1-N2	N2-N3	N3-C11	C11-C12	N1-C12	N1-C14	C10-C11
**10**							
Conf_anti_1							
BP86/def2-TZVP	1.331	1.324	1.383	1.390	1.366	1.430	1.461
BP86/def2-TZVP/BJ	1.331	1.324	1.381	1.389	1.366	1.425	1.456
Conf_anti_2							
BP86/def2-TZVP	1.331	1.324	1.383	1.390	1.366	1.429	1.461
BP86/def2-TZVP/BJ	1.331	1.325	1.381	1.389	1.366	1.425	1.456
Exp.	1.323(6)	1.294(7)	1.374(7)	1.360(7)	1.353(7)	1.415(7)	1.445(8)

*Structural Parameters of NHCs **A** and **B***

As for the presented systems **8'** to **10'** no experimental Tolman electronic parameters could be obtained, the reference systems **A**[11] and **B**[12] as depicted in Figure S59 were also investigated. For sake of comparison, structural parameters are listed in Table S57. These systems were chosen as their experimental TEP values belong to the most electron poor NHCs. For compound **A**, two low energy conformers were found, which differ in the orientation of the phenyl ring. The planar structure (Conf_2) is found to be more stable by 13.1 kJ/mol (BP86/def2-TZVP).

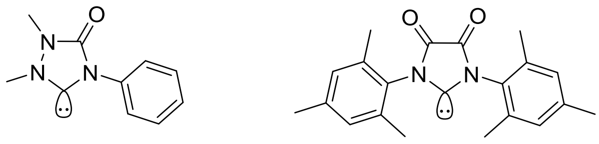

A **B**

Figure S59. Molecular structures of the reference systems **A**[11] and **B**[12].

Table S57. Structural parameters of **A** and **B**. Bond length are given in Å, angle in °.

	N1-N2	N2-C2	C2-N3	C1-N3	C1-N1	C1-N3-C2-C3	
**A**							
Conf_1							
BP86/def2-TZVP	1.411	1.381	1.422	1.380	1.338	90.1	
Conf_2							
BP86/def2-TZVP	1.406	1.380	1.426	1.386	1.332	-0.1	
	**C1-N1**	**N1-C2**	**C2-C3**	**N2-C3**	**C1-N2**	**N1-C4**	**N2-C5**
**B**							
BP86/def2-TZVP	1.384	1.406	1.553	1.406	1.443	1.443	1.443

Tolman Electronic Parameters

Mathew and Sureh's parametrization scheme allow for an *in-silico* Tolman electronic parameter (TEP) calculation when experimental TEP values are not accessible. However, for their initial linear regression, data for electron poor NHCs with high TEP values is scarce. To ensure reliability and validity of Eq. 1, we calculated TEP values for two additional electron poor NHCs, **A** and **B**, for which experimental values were also known. As seen from Table S58, we found an excellent agreement between calculated and measured TEP values, for **A** 2060/2061 cm^{-1} (calculated) vs. 2060 cm^{-1} (measured) and for **B** 2070 cm^{-1} (calculated) vs. 2069 cm^{-1} (measured). (see Figure S60 for an overview of the investigated systems). Thus, we are confident to obtain reliable calculated TEPs for NHCs that are even more electron deficient.

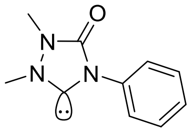

A

TEP_{cal} = 2060 cm^{-1}

TEP_{exp} = 2060 cm^{-1}

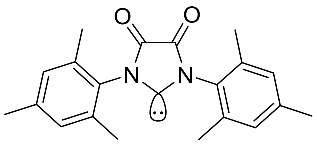

B

TEP_{cal} = 2070 cm^{-1}

TEP_{exp} = 2069 cm^{-1}

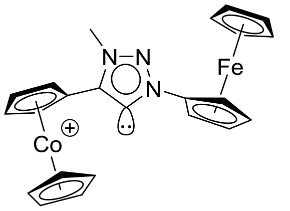

8'

TEP_{cal} = 2078 cm^{-1}

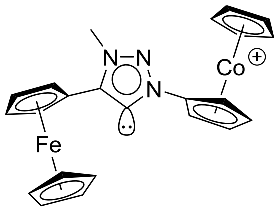

9'

TEP_{cal} = 2077 cm^{-1}

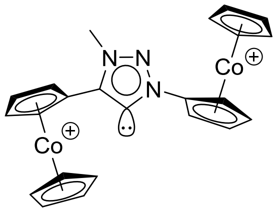

10'

TEP_{cal} = 2109 cm^{-1}

Figure S60. Molecular structures with corresponding TEP values of the reference systems **A** and **B** (top) compared with the novel carbenes **8'** to **10'** (bottom), exhibited in this work.

To test the dependence of the Tolman electronic parameter (TEP) on the conformation for the presented compounds **8'** to **10'**, TEP parameters for all four conformers of **8'**, two with a *syn* conformation of the ferrocenyl and cobaltoceniumyl substituents and two, where they are in *anti* conformation, were tested. TEP values listed in Table S58 show little dependence on the ferrocenyl-cobaltoceniumyl conformation and all conformers have similar TEP values of around ca. 2078 cm^{-1}. Thus, TEP values of **9'** and **10'** are only depicted for the most stable *anti* conformer.

The TEP values of **9'** is very similar to **8'** because both molecules have a ferrocenyl and a cobaltocenyl substituent and both are still in the interpolation range of Mathew and Suresh's correlation between experimental TEP and V_C. **10'** with two cobaltocenyl moieties – on the other hand – is significantly more electron poor and its TEP value is extrapolated by employing Eq. 1 to be 2108.7 cm^{-1}. Although no experimental TEP data in this regime is available, the calculated value of 2108.7 cm^{-1} is reasonable

because the cobaltocenyl substituents are more electron withdrawing, thus depleting electron density at the carbene carbon even more compared to **8'** and **9'**.

The TEP value of **10'** is remarkably similar to that of PF_3 (2110.0 cm^{-1}), which is the most *π*-acidic phosphine[11] indicating that it represents one of the electrophilic NHCs.

Table S58. Tolman electronic parameter (TEP) for **8'**-**10'** and the NHC reference systems **A** and **B**, for which experimental TEP values are available. The electrostatic potential V_C is calculated at the carbene atom as B3LYP/6-311++G(d,p) on the BP86/def2-TZVP optimized structures.

	V_c [kcal/mol]	TEP [cm^{-1}]
**8'**		
*conf_anti_1**	-9215.76	2077.9
conf_anti_2	-9215.74	2077.9
conf_syn_1	-9215.68	2077.9
conf_syn_2	-9215.55	2078.0
**9'**		
conf_anti_1	-9217.53	2077.1
**10'**		
conf_anti_1	-9144.75	2108.7
**A**		
calc. conf_1	-9257.21	2060
calc. conf_2	-9254.45	2061
Exp.		2060
**B**		
calc.	-9234.03	2070
Exp.		2069

*Conformation of X-ray crystal structure.

Calculation of pK_a

pK_a values were calculated for the deprotonation of the unsubstituted triazole carbon in **8** to **10** to yield the triazolylidene ligands **8'** to **10'** according to the thermodynamic cycle depicted in Scheme S1. As exemplary shown for **8**, calculated pK_a – listed in Table S59 – show little dependence on the conformation of the ferrocenyl and cobaltoceniumyl substituents but all yield very similar results. However, differences up to 3 pK_a units were found for the *conf_anti_2* conformer of **8**, when different density functionals in combination with or without empirical dispersion corrections were used. This results in an average pK_a of **8** of about 24 with a standard deviation of around ± 1. However, we are less interested in the accurate number but more in the acidities of **8** to **10**, relative to each other, thus, only BP86 values are discussed in the main manuscript. While the pK_a values of **8** and **9** are very similar, **10** is found to be considerably more acidic with pK_a values being significantly smaller than those of **8** and **9**.

Table S59. Calculated pK_a values for **8-10**.

	**BP86/def2-TZVP**	**BP86/def2-TZVP/BJ**	**PBE0/def2-TZVP**	**PBE0/def2-TZVP/BJ**
**8**				
*conf_anti_1**	22.3	23.6		
conf_anti_2	22.4	23.7	24.6	24.4
conf_syn_1	22.4	24.3		
conf_syn_2	22.7	23.1		
**9**				
conf_anti_1	23.5	22.9		
**10**				
*conf_anti_1**	17.1	17.3		
conf_anti_2	18.3	16.7	19.9	

*Conformation of X-ray crystal structure.

Electrochemical Section

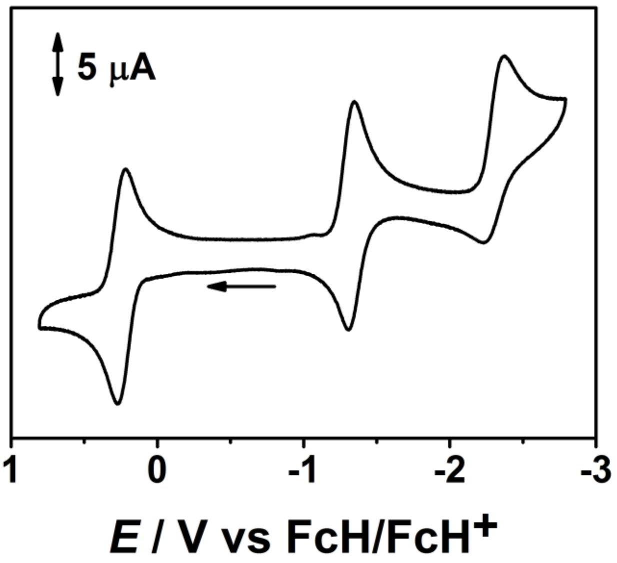

Figure S61. Cyclic voltammogram of **5** in CH_3CN / 0.1 M Bu_4NPF_6.

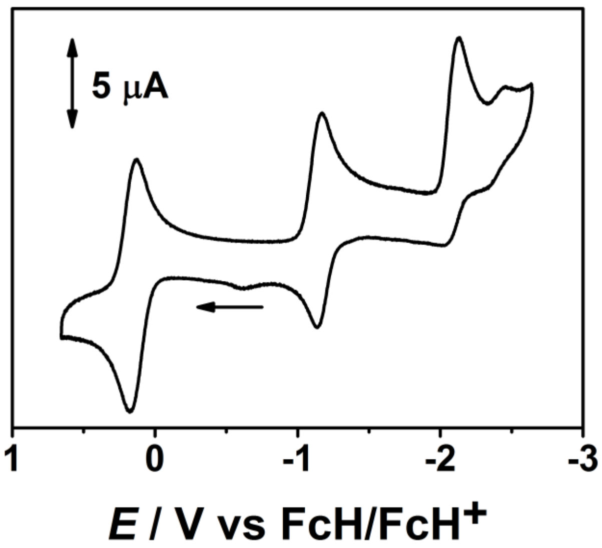

Figure S62. Cyclic voltammogram of **6** in CH_3CN / 0.1 M Bu_4NPF_6.

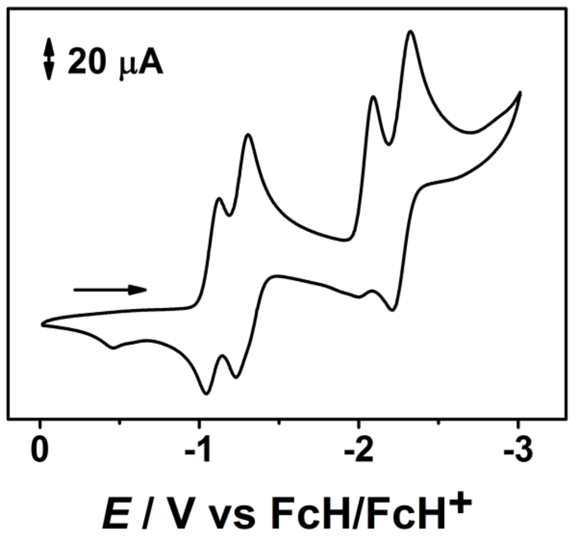

Figure S63. Cyclic voltammogram of **7** in CH_3CN / 0.1 M Bu_4NPF_6.

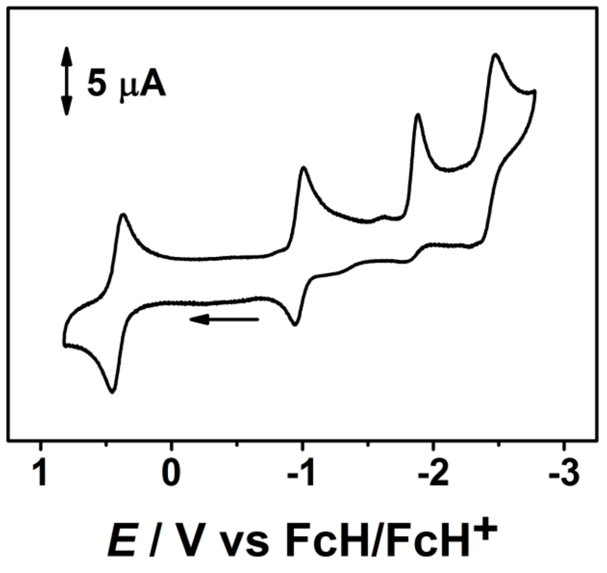

Figure S64. Cyclic voltammogram of **8a** in CH_3CN / 0.1 M Bu_4NPF_6.

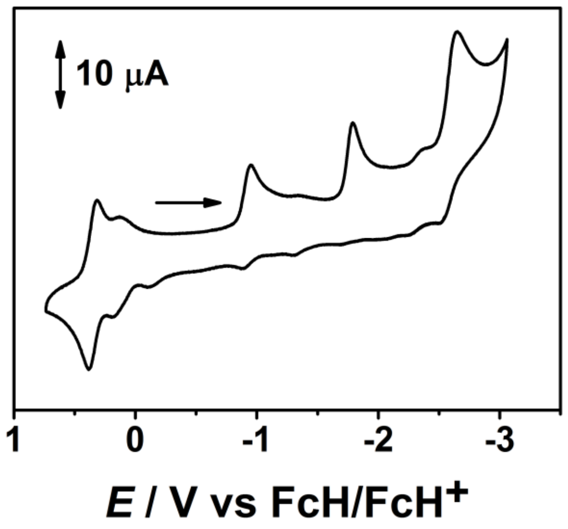

Figure S65. Cyclic voltammogram of **9a** in CH_3CN / 0.1 M Bu_4NPF_6.

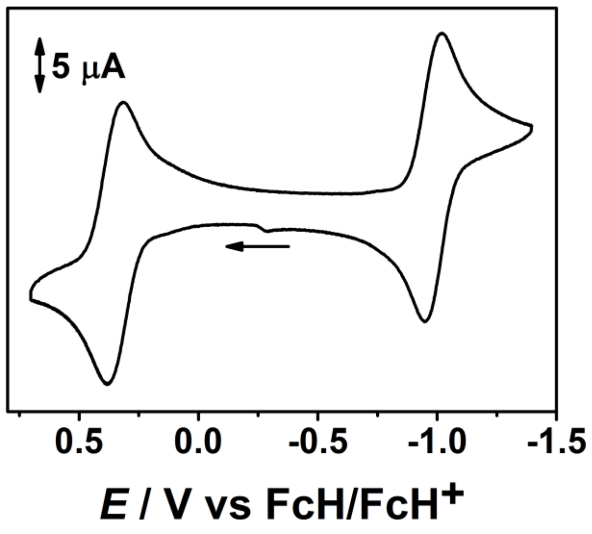

Figure S66. Cyclic voltammogram of **9a** in CH_3CN / 0.1 M Bu_4NPF_6 displaying the first oxidation and first reduction.

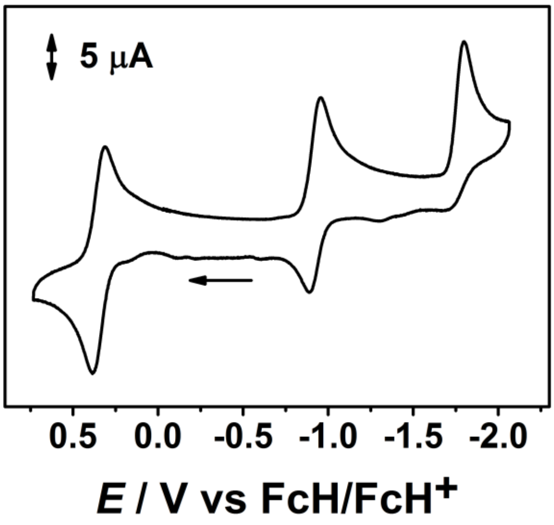

Figure S67. Cyclic voltammogram of **9a** in CH_3CN / 0.1 M Bu_4NPF_6 displaying the first oxidation and the first two reductions.

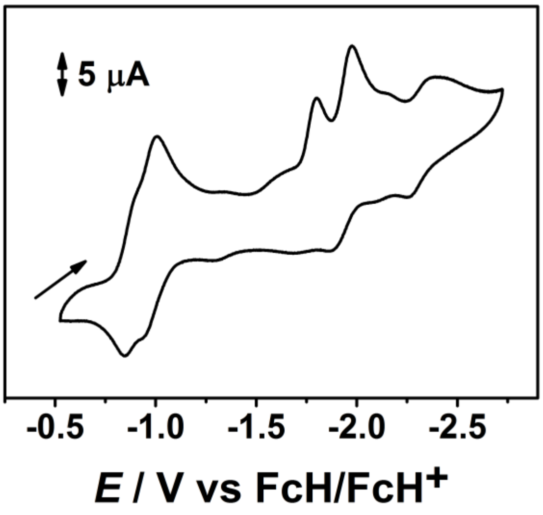

Figure S68. Cyclic voltammogram of **10a** in CH_3CN / 0.1 M Bu_4NPF_6.

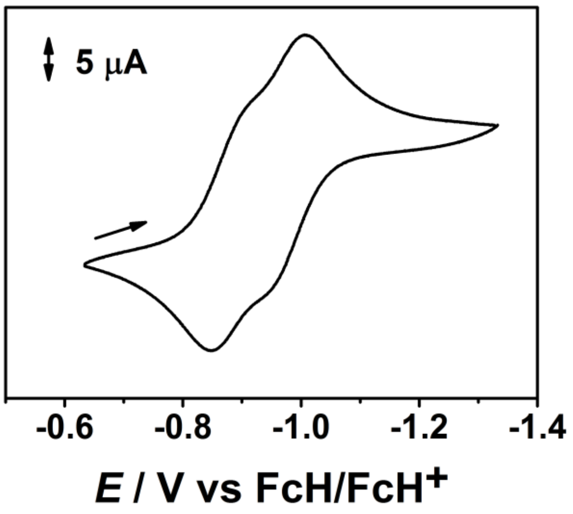

Figure S69. Cyclic voltammogram of **10a** in CH_3CN / 0.1 M Bu_4NPF_6 displaying the first two reductions.

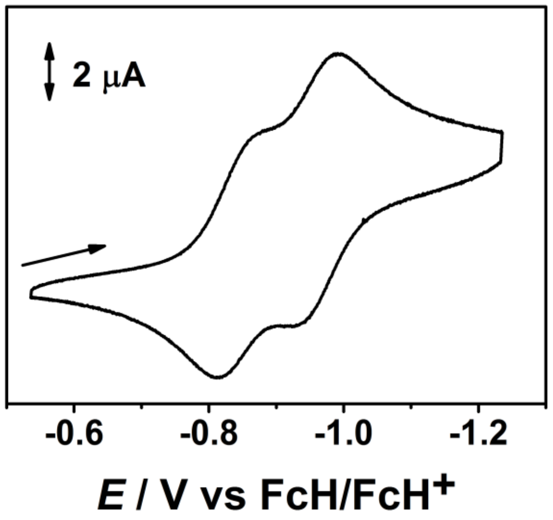

Figure S70. Cyclic voltammogram of **10a** in CH_3CN / 0.1 M Bu_4NBArF displaying the first two reductions.

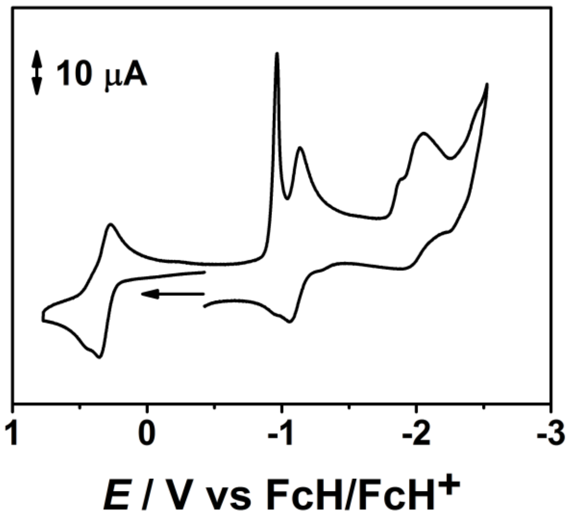

Figure S71. Cyclic voltammogram of **11** in CH_3CN / 0.1 M Bu_4NPF_6.

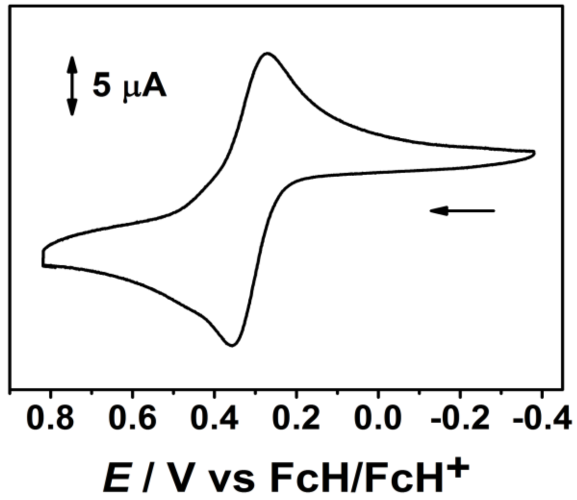

Figure S72. Cyclic voltammogram of **11** in CH_3CN / 0.1 M Bu_4NPF_6 displaying the first oxidation.

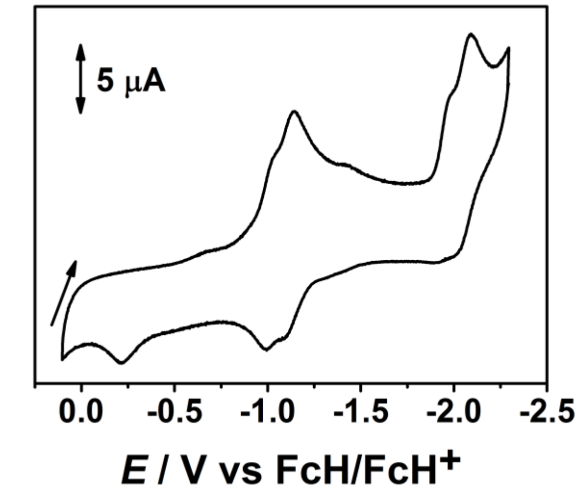

Figure S73. Cyclic voltammogram of **13** in CH_3CN / 0.1 M Bu_4NPF_6.

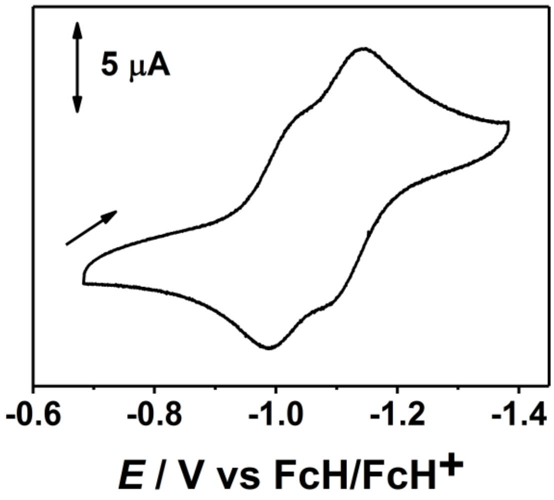

Figure S74. Cyclic voltammogram of **13** in CH_3CN / 0.1 M Bu_4NPF_6 displaying the first two reductions.

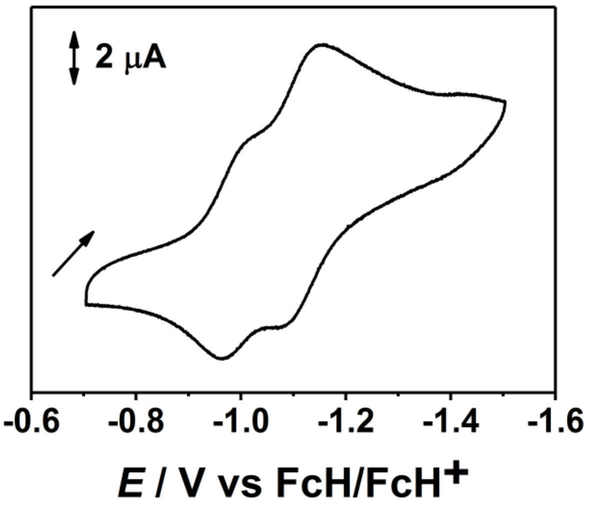

Figure S75. Cyclic voltammogram of **13** in CH_3CN / 0.1 M Bu_4NBArF displaying the first two reductions.

Table S59. Standard potentials in V of the compounds obtained from cyclic voltammetry measurements in CH_3CN / 0.1 M Bu_4NPF_6.

Compound	$E_{1/2}$(ox1)	$E_{1/2}$(red1)	$E_{1/2}$(red2)	$E_{1/2}$(red3)	$E_{1/2}$(red4)	Epc
**5**	0.25	−1.33	−2.30[a]			
**6**	0.15	−1.15	−2.08[a]	−2.45[b]		
**7**		−1.08	−1.26	−2.04[a]	−2.27[a]	
**8**	0.41	−0.98	−1.83[a]	−2.38[a]		
**9**	0.35	−0.98	−1.74[a]	−2.58[a]		
**10**		−0.93	−0.93	−1.80[b]	−1.98[b]	−2.40
**10**[c]		−0.84	−0.96	−1.81[b]	−1.97[b]	−2.44
**11**	0.31	−1.10	−1.97[a]			
**13**		−1.01	−1.12	−2.09[b]		
**13**[c]		−0.99	−1.12	−2.10[b]		

[a] half-wave potential for irreversible processes
[b] peak-forward potential for irreversible processes
[c] with Bu_4NBArF as electrolyte

Catalytical Section

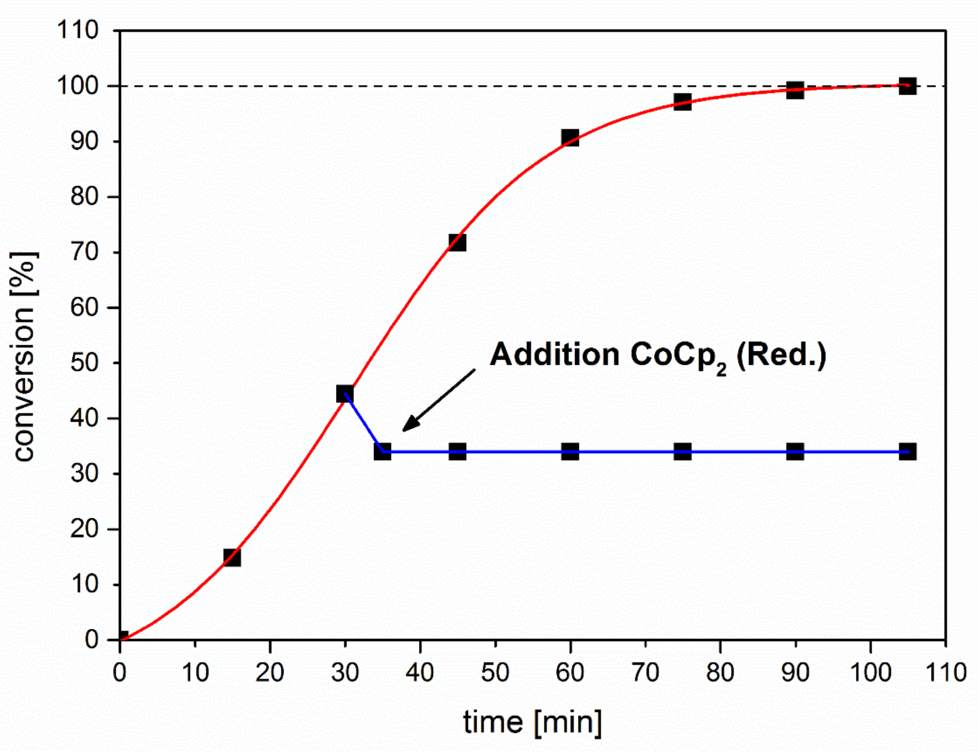

Figure S76. Time versus conversion plot for the synthesis of oxazoline catalyzed by complex **13** (red), compared to the reaction process after adding $CoCp_2$ as reducing agent (blue).

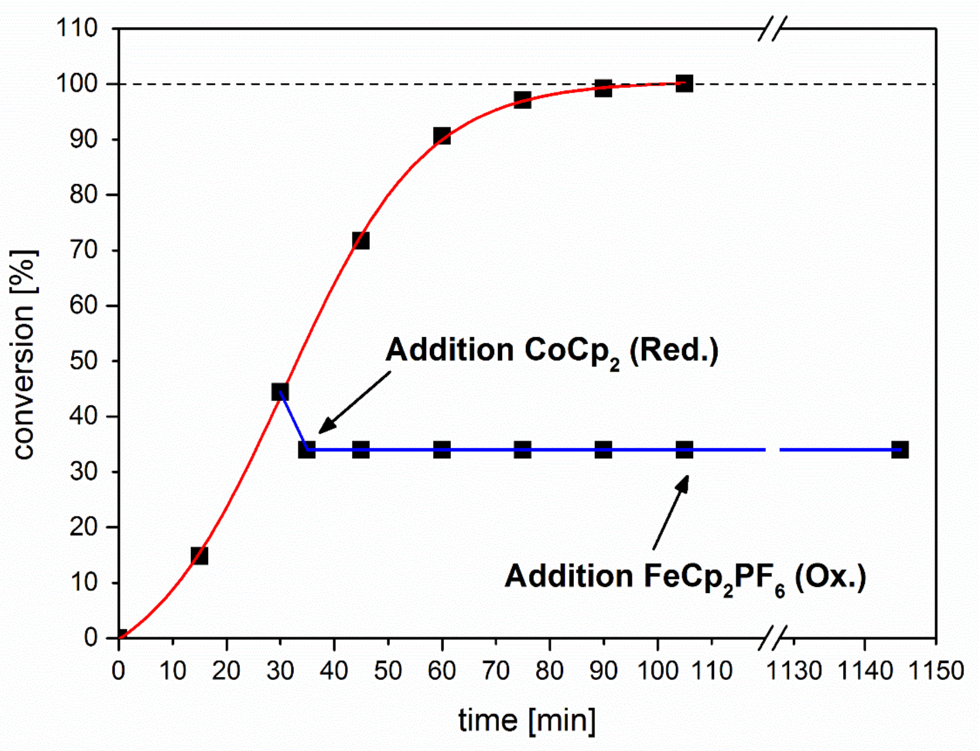

Figure S77. Time versus conversion plot for the synthesis of oxazoline catalyzed by complex **13** (red), compared to the reaction process after adding $CoCp_2$ as reducing agent (blue). Further addition of $FeCp_2PF_6$ as oxidizing agent lead to no regeneration of the catalyst (blue).

References

(1) TURBOMOLE V7.0 2015, a development of University of Karlsruhe and Forschungszentrum Karlsruhe GmbH, 1989-2007, TURBOMOLE GmbH, since 2007; available from http://www.turbomole.com.

(2) Gaussian 09, Revision D.01, M. J. Frisch, G. W. Trucks, H. B. Schlegel, G. E. Scuseria, M. A. Robb, J. R. Cheeseman, G. Scalmani, V. Barone, G. A. Petersson, H. Nakatsuji, X. Li, M. Caricato, A. Marenich, J. Bloino, B. G. Janesko, R. Gomperts, B. Mennucci, H. P. Hratchian, J. V. Ortiz, A. F. Izmaylov, J. L. Sonnenberg, D. Williams-Young, F. Ding, F. Lipparini, F. Egidi, J. Goings, B. Peng, A. Petrone, T. Henderson, D. Ranasinghe, V. G. Zakrzewski, J. Gao, N. Rega, G. Zheng, W. Liang, M. Hada, M. Ehara, K. Toyota, R. Fukuda, J. Hasegawa, M. Ishida, T. Nakajima, Y. Honda, O. Kitao, H. Nakai, T. Vreven, K. Throssell, J. A. Montgomery, Jr., J. E. Peralta, F. Ogliaro, M. Bearpark, J. J. Heyd, E. Brothers, K. N. Kudin, V. N. Staroverov, T. Keith, R. Kobayashi, J. Normand, K. Raghavachari, A. Rendell, J. C. Burant, S. S. Iyengar, J. Tomasi, M. Cossi, J. M. Millam, M. Klene, C. Adamo, R. Cammi, J. W. Ochterski, R. L. Martin, K. Morokuma, O. Farkas, J. B. Foresman, and D. J. Fox, Gaussian, Inc., Wallingford CT, 2009.

(3) The PyMOL Molecular Graphics System, Version 1.8 Schrödinger, LLC.

(4) J. Mathew, C. H. Suresh, *Inorg. Chem.* **2010**, *49*, 4665-4669.

(5) A. D. Becke, *J. Chem. Phys.* **1993**, *98 (2)*, 1372-1377.

(6) (a) K. Raghavachari, G. W. Trucks, *J. Chem. Phys.* **1989**, *91*, 1062-1065. (b) R. Krishnan, J. S. Binkley, R. Seeger, J. A. Pople, *J. Phys. Chem.* **1980**, *72*, 650-654.

(7) T. Clark, J. Chandrasekhar, G. W. Spitznagel, P. v. R. Schleyer, *J. Comp. Chem.* **1983**, *4*, 294-301.

(8) A. Klamt, G. Schüürmann, *J. Chem. Soc. Perkin Trans. 2*, **1993**, *5*, 799-805.

(9) Single-Ion Solvation. Experimental and Theoretical Approaches to Elusive Thermodynamic Quantities. P. Hünenberger, M. Reif, Royal Society of Chemistry - Theoretical and Computational Chemistry Series London, UK, ISBN: 978-1-84755-187-0 (2011).

(10) See e.g., J. Ho, M. L. Coote, *Wiley Interdiscip. Rev. Comput. Mol. Sci.* **2011**, *1*, 649-660, and references therein.

(11) M. Jonek, J. Diekmann, C. Ganter, *Chem. Eur. J.* **2015**, *21*, 15759-15768.

(12) M. Braun, W. Frank, G. J. Reiss, C. Ganter, *Organometallics* **2010**, *29*, 4418-4420.

(13) T. Kruck, *Angew. Chem. Int. Ed*. **1967**, *6*, 53-67.
